# Discovery of benzochromene derivatives first example with dual cytotoxic activity against the resistant cancer cell MCF-7/ADR and inhibitory effect of the *P*-glycoprotein expression levels

**DOI:** 10.1080/14756366.2022.2155814

**Published:** 2023-01-20

**Authors:** Lali M. Al‑Harbi, Eman A. Al-Harbi, Rawda M. Okasha, R. A. El-Eisawy, Mohammed A. A. El-Nassag, Hany M. Mohamed, Ahmed M. Fouda, Ahmed A. Elhenawy, Ahmed Mora, Ahmed M. El-Agrody, Heba K. A. El-Mawgoud

**Affiliations:** a Chemistry Department, Faculty of Science, King Abdul-AzizUniversity, Jeddah, Saudi Arabia; b Chemistry Department, Faculty of Science, Taibah University, Medina, Saudi Arabia; c Chemistry Department, Faculty of Science, Al-Azhar University, Cairo, Egypt; d Chemistry Department, Faculty of Science and Art, Al-Baha University, Al-Baha, Saudi Arabia; e Chemistry Department, Faculty of Science, Jazan University, Jazan, Saudi Arabia; f Chemistry Department, Faculty of Science, King Khalid University, Abha, Saudi Arabia; g Chemistry Department, Faculty of Science and Art, AlBaha University, Al Bahah, Saudi Arabia; h Chemistry Department, Faculty of Women for Arts, Science, and Education, Ain Shams University, Cairo, Egypt

**Keywords:** Ultrasound synthesis, 1H-Benzo[f]chromenes, antitumor activity, MCF-7/ADR, *P-*glycoprotein, western blot, cell cycle arrest, SAR

## Abstract

A series of 1*H*-benzo[*f*]chromene moieties (**4a–z**) were synthesised under Ultrasonic irradiation and confirmed with spectral analyses. Derivative **4i** solely possessed an X-ray single crystal. The anti-proliferative efficacy of the desired molecules has been explored against three cancer cells: MCF-7, HCT-116, and HepG-2 with the cytotoxically active derivatives screened against MCF-7/ADR and normal cells HFL-1 and WI-38. Furthermore, compounds **4b–d**, **4k**, **4n**, **4q**, and **4w**, which possessed good potency against MCF-7/ADR, were tested as permeability glycoprotein (*P-*glycoprotein [*P*-gp]) expression inhibitors. The attained data confirmed that **4b–d**, **4q**, and **4w** exhibited strong expression inhibition against the *P-*gp alongside its cytotoxic effect on MCF-7/ADR. The western blot results and Rho123 accumulation assays showed that compounds **4b–d, 4q**, and **4w** effectively inhibited the *P*-gp expression and efflux function. Meanwhile, **4b–d**, **4q**, and **4w** induced apoptosis and accumulation of the treated MCF-7/ADR cells in the G1 phase and **4k** and **4n** in the S phase of the cell cycle.

## Graphical Abstract

**Figure:**
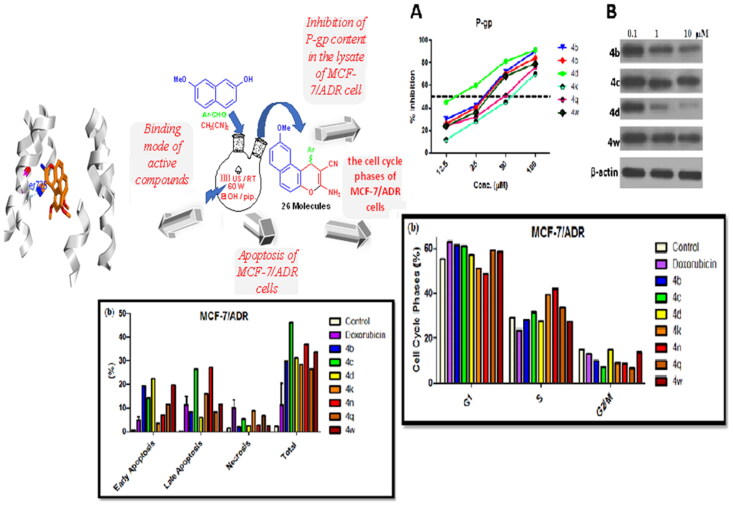

## Introduction

An efficient and green tool for organic reactions is to use the Ultrasound technique as a non-conventional energy source, which can provide a range of benefits, such as shorter reaction times, easier operation, and improved yields of pure products^1–4^. Benzochromene is one of the most attractive oxygen-incorporating heterocyclic scaffolds in the light of their unique natural characteristics and their biomedical performances^5^. Benzochromene derivatives are the most valuable pharmacological compounds, possessing a range of significant biological properties, such as antimicrobial^6–9^, anti-inflammatory and analgesic^10^, antiviral and central nerve system activities^11^^,^^12^, antioxidant^6^^,^^13^, hypolipidemic^14^, anti-proliferative^15^, anticancer^16^, anti-rheumatic^17^, anti-tubercular^18^^,^^19^, Alzheimer’s preventative^20^, anti-obesity^21^ effects, and agents. Similarly, various studies have reported their targeting of signalling pathways, which is critical for the inhibition of tumour cell proliferation and enables them as prospective primary candidates in the manufacture of antitumor agents^22–27^. For example, some benzochromene derivatives displayed inhibitory behaviour of the *c*-Src kinase with their antitumor ability and apoptotic effects^28–31^. Other benzochromene templates exhibited antitumor activities, trigger cell cycle arrest at M, S, and G2 stages, enhance the formation of caspases 3/7, and initiate apoptosis with diminished toxicity in tumour cells through the dual inhibition of topoisomerase I/II and in breast cancer xenografts^27^^,^^31–35^. Alongside its anti-proliferative features, chromene derivatives exhibited a DNA binding ability, the inhibition of the Bcl-2 protein^26^ and a high potency for the hAChE^36^. One of the main molecular mechanisms shared in failure of chemotherapy is multi-drug resistance (MDR) ^37^^,^^38^. The main cause of MDR is overexpression of the ABC transporters, such as permeability glycoprotein (*P*-glycoprotein [*P*-gp)), multidrug resistance protein 1 (MDR1), and ATP-binding cassette sub-family B member 1 (ABCB1), (DGAT-1), and (hCA XII) inhibitors^38–40^. In addition, *P*-gp/(ABCB1) plays a main role in the multidrug-resistant phenotype in cancer mediating MDR^41^. Our continuous endeavours to develop powerful, novel oxygen-integrating heterocyclic molecules antitumor and antimicrobial agents are expanded upon by this current report^42–51^.

## Aim of the work and rationale

**Chart 1.**
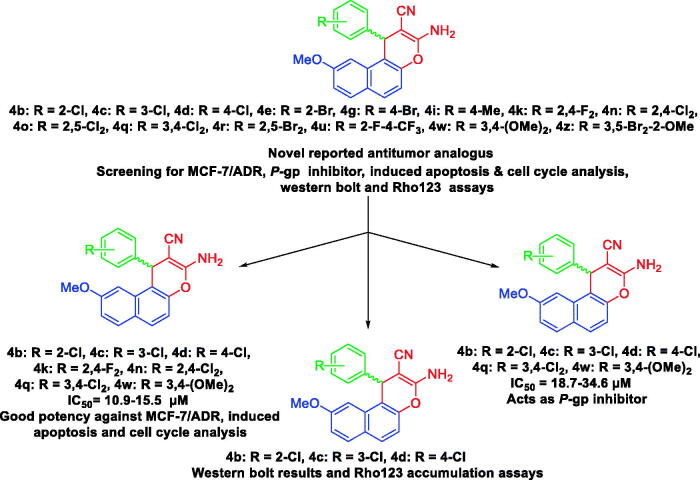
Study rationale analysis of antitumor activities, MCF-7/ADR, P-gp inhibitor, apoptosis, and cell cycle analysis western blot and Rh123 results.

Herein, we report the synthesis of anti-proliferative 1*H*-benzo[*f*]chromene derivatives **(a–z)**. In this work, we explore the potent and anti-proliferative effects of the substituents on the C-1 position of the phenyl-bearing moieties of the 1*H*-benzo[*f*]chromene derivatives. To achieve this goal, three tumour cell lines MCF-7 (breast cancer), HCT-116 (human colon cancer), and HepG-2 (hepatocellular carcinoma) were utilised to investigate the anti-proliferative activity of the desired molecules. Subsequently, the highly cytotoxically-active derivatives **4b–e**, **4g**, **4i**, **4k**, **4n**, **4o**, **4q**, **4r**, **4u**, **4w**, and **4z** were subjected to further screenings against Adriamycin (ADR)-resistant human breast cancer cells (MCF-7/ADR) and the normal cell lines human foetal lung (HFL-1) alongside WI-38 human (diploid fibroblasts). Furthermore, compounds **4b–d**, **4k**, **4n**, **4q**, and **4w** revealed good potency towards MCF-7/ADR cells and were examined for the inhibition of *P*-gp expression; meanwhile, **4b–d**, **4q**, and **4w** displayed high potency against *P*-gp expression MDR in MCF-7/ADR and they are able to inhibit *P*-gp. Besides, western blot results and Rho123 accumulation assays showed that compounds **4b–d**, **4q**, and **4w** effectively inhibited *P*-gp expression and efflux function, while compounds **4b–d**, **4q**, and **4w** induced accumulation of the treated MCF-7/ADR cells in the G1 phase, and compounds **4k** and **4n** in the S phase of the cell cycle, as illustrated in Chart 1.

**Chart 2.**
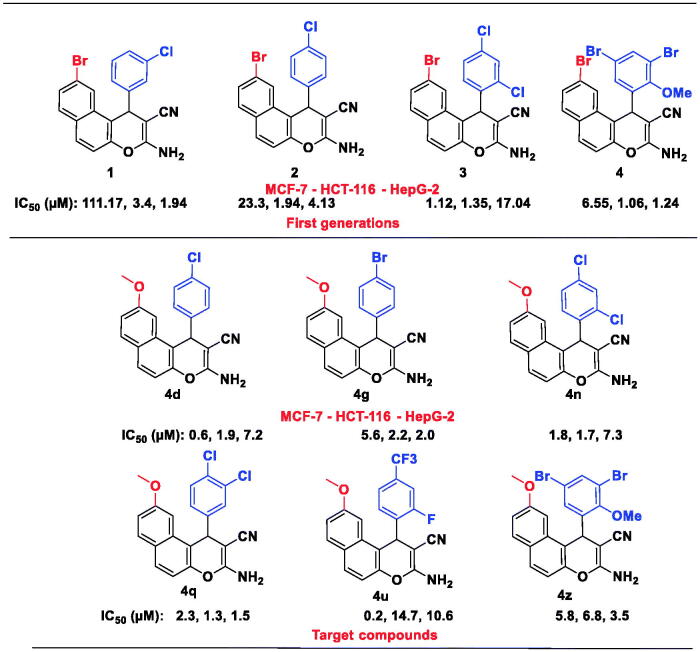
Rationale for designing target compounds.

The rational design of the 9-methoxy-1*H*-benzo[*f*]chromene derivatives was based on the following considerations: (i) the 1*H*-benzo[*f*]chromene template, (ii) the existence of the efficacious 9-position substituent, (iii) the varying sorts of substituents located on the aryl moieties bound to the 9-methoxy-1*H*-benzo[*f*]chromene framework at the 1-position which emerges as a critical component in cytotoxic behaviour, and (iv) comparative analyses regarding the performances of the freshly prepared molecules and the formerly prepared molecules with a bromine atom at the 9-position^24^ as illustrated in Chart 2.

The final feature in this rationale study revealed that the recent molecules **4b**, **4g**, **4n**, **4q**, **4u**, and **4z** possessed a remarkable influence regarding their behaviours against tumour cells, which had an elevated potency in comparison with the molecules **1–4**^24^.

## Results and discussion

### Chemistry

**Scheme 1.**
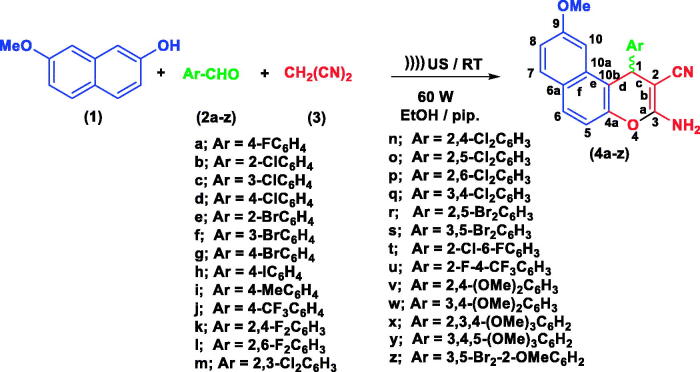
Synthesis of 1H-benzo[f]chromene derivatives (**4a–z).**

A series of substituted fused heterocyclic derivatives **(4a–z)** were synthesised using 7-methoxynaphthalen-2-ol **(1)** as a starting material. The attained molecules **4e, 4f, 4j,** and **4r–u** are described herein for the first time, while previous reported compounds **4a–d, 4g–i, 4k–q,** and **4v–z** have been acquired employing an ultrasound as a new synthetic strategy. The interaction of **1** with a number of aldehydes derivatives **(2a–z)** in the presence of and malononitrile **(3)** and absolute ethanol/piperidine solution has been achieved utilising 60 W of ultrasonic irradiation conditions at ambient temperature and allowed for the formation of *β*-enamionitriles containing 9-methoxy-1*H*-benzo[*f*]chromene motifs **(4a–z)** as shown in Scheme 1.

The 1-position of compounds **4a–z** is a chiral centre and all the reactions were controlled using TLC technique. The optical execution of **4a–z** was detected utilising using a Carl Zeiss polarimeter and displayed zero rotation (i.e. optically inactive) due to their occurrence as a racemic (±) mixture^52^^,^^53^, as shown in Scheme 1.

The structure’s identity of the novel molecules **4e, 4f, 4j,** and **4r–u** was substantiated *via* their IR, ^1^H NMR, ^13^C NMR, ^13^C NMR-APT, and MS data. Supporting evidences for the suggested structures comes from their infra-red spectra which exhibited at *υ* 3448–3402, 3338–3291, 3261–3200 cm^−1^ for an NH_2_ group and at *υ* 2204–2167 cm^−1^ for the CN group of compounds **4e, 4f, 4j,** and **4r–u**. The ^1^H NMR data of **4e, 4f, 4j,** and** 4r–u** demonstrated singlet signals at *δ* 7.17–6.93 ppm that is corresponding to the amino protons, while signals at *δ* 6.06–5.26 are attributable for the methine protons. Moreover, the ^13^C NMR-APT of compound **4s** as well as the MS spectrum, the single crystal X-ray analysis of compounds **4d, 4 g,**^9^^,^^54^ and **4i** provided a conclusive verification for the desired molecules (see Supplementary Materials 1S-100S).

### Crystal data

**Figure 1.**
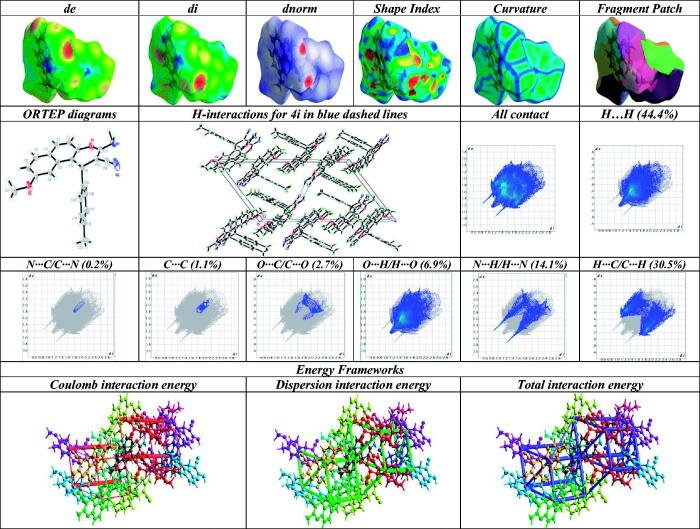
2D, 3D, and energy framework molecular packing of compound **4i**.

Table 1 illustrates the crystallographic analysis and the refinement information of molecule **4i** with the chemical formula of C_22_H_18_N_2_O_2_. The asymmetric unit of compound **4i** is containing one molecule as shown in Figure 1. The length of all bonds and the angles are in normal ranges^55^. In the crystal packing, Figure 1, molecules of compound **4i** were linked via two intermolecular hydrogen bonds (Table 2).

**Table 1.** X-Ray experimental details for compounds **4i**.

Crystal data	4i
Chemical formula	C_22_H_18_N_2_O_2_
Mr	342.38
Crystal system, space group	Monoclinic, *P*2_1_/*c*
Temperature (K)	293
*a*, *b*, *c* (Å)	14.5119 (11), 6.7076 (4), 20.8913 (12)
*α, β, γ* (°)	90.0, 119.746 (4), 90.0
V (Å^3^)	1765.6 (2)
*Z*	4
Radiation type	Mo *K*α
µ (mm^−1^)	0.08
Crystal size (mm)	0.53 × 0.31 × 0.09
Data collection	
Diffractometre	Bruker APEX-II D8 venture diffractometre
Absorption correction	Multi-scan SADABS Bruker 2018
T_min_, T_max_	0.887, 0.903
No. of measured, independent, and observed [*I* > 2σ(I)] reflections	20,263, 4057, 1645
R_int_	0.212
Refinement	
R[*F^2^* > 2σ( *F^2^*)], wR( *F^2^*), S	0.107, 0.306, 0.97
No. of reflections	4057
No. of parameters	246
Δ*ρ*_max_, Δ*ρ*_min_ (e Å^−3^)	0.44, −0.38
CCDC No.	2,132,096

**Table 2.** Hydrogen-bond geometry (Å, °) for compound **4i**.

Compound **4i**				
*D*—H···*A*	*D*—H	H···*A*	*D*···*A*	*D*—H···*A*
N1—H2N1···N2^i^	0.90 (6)	2.60 (6)	3.389 (6)	147 (7)
N1—H1N1···N2^ii^	1.00 (6)	2.14 (6)	3.114 (7)	166 (4)
Symmetry codes: (i) *x*, *y* + 1, *z*; (ii) −*x* + 3, −*y* − 2, −*z* + 2.				

The central pyran ring (O1–C1–C10–C11–C12–C13) is almost planar with the largest deviation from the mean plan of −0.013(2) at C10 atom, which connected to naphthalene ring and to a tolyl ring (Figure 1). The dihedral angle between the main core ring and the tolyl is 73.51(7)°. The mean plan through the naphthalene ring (C8—C13) is nearly orthogonal to that of the tolyl ring as signified by the dihedral angle between them of 144.44(4)°. Also, there are two intermolecular hydrogen bonds interactions, N2—H2N1•••N1, given in Table 2.

Crystal explorer17^56^ was used to investigate the Hirschfield-surface “HS” with their 2D-fingerprint surfaces “2D-FS” analysis, which displayed the intermolecular interactions in the crystal packing. HS was mapped over *d_norm_* for **4i** (Figure 1). The HS-volume “HSV” and the surface area “HSA” are 433.89 Å^3^ and 382.56 Å^2^, respectively, which represented in red(−*d_norm_*), blue(+*d_norm_*), and white (normal *d_norm_*) colour schemes. Negative, positive, and normal *d_norm_*, are shorter, longer and equal contacts than van-der-Waals-radii, respectively. The decomposed 2D-HS in (Figure 1) showed the chief contribution in C–H and H–H contacts, which contribute the most (30.9%) to the total HSA. The lowest contributions are from C–C, C–N contacts, while C–O, O–H, and N–H displayed contributions at 2.7, 6.9, and 14.1 contacts. The strong intermolecular interactions appear as distinct spikes in the fingerprint plots.

Shape index (SI) and curvedness (CS) were used to study the curvature of the surface (Figure 1). The SI sheds light on the stacking arrangement for **4i**, which was used to identify the complementary cavities (red) and bumps (blue) where two molecular HS close to each other. The blue and red coloured triangular-shaped patterns represented the particular stacking arrangement of **4i**. Molecule **4i** displayed no significant triangular-patterns on SI map, which suggested the absence of the π–π interaction. The CS was represented by the function of the r.m.s. curvature. CS mapped was found to be in range of (−4.0–4.0) which categorised by bulky green-coloured regions and detached by deep blue boundaries. Since there is no flat surface seen on the CS plot, there is no planar stacking between the molecules.

The B3LYP/6-311G(d,p) function was employed for simulating the intermolecular interaction energies in the crystal to examine their stabilisation degree of the crystal lattice (Table 3). Figure 1 represents their energy framework as a cylinder-shaped, which displayed the relative-strength for the interaction energies and also give a clear view for their role in the stabilisation of the crystal packing. According to Table 3, it is clear that the dispersion force (−233.3 kJ/mol) plays a central role, which having a maximum energy of −233.3 kJ/mol, among the total interaction energy which is −244.7 kJ/mol.

**Table 3.** The energetic interaction in kJ/mol of the **4b** at B3LYP/6-311G(d,p), *R*: Centroid distance; Etot: The total interaction energy; Eele: sum classical electrostatic and coulomb energy; Epol: polarisation energy, Edisp: dispersion energy; Erep; exchange repulsion energy.

	*N*	Symop	*R*	E_ele	E_pol	E_dis	E_rep	E_tot
	2	−*x*, *y* + 1/2, −*z* + 1/2	7.06	−7.4	−1.6	−43	21.1	−33.3
	1	−*x*, −*y*, −*z*	7.40	−14.8	−4.5	−72	40.9	−56.2
	2	*x*, *y*, *z*	6.71	−16.7	−3.7	−44	27	−42.2
	1	−*x*, −*y*, −*z*	10.29	−8.4	−2.1	−21	10.9	−21.9
	2	−*x*, *y* + 1/2, −*z* + 1/2	9.34	−5.2	−2.8	−30	14.7	−25
	2	*x*, −*y* + 1/2, *z* + 1/2	11.1	−1.5	−1.7	−4.7	3	−5
	1	−*x*, −*y*, −*z*	11.32	−58.2	−13.5	−11	39.8	−56.9
	2	*x*, −*y* + 1/2, *z* + 1/2	10.8	0.9	−0.6	−5	1.2	−3.1
	1	−*x*, −*y*, −*z*	12.92	1.1	−0.6	−2.1	0	−1.1

## Biological activity

### Cell viability assay

**Figure 2.**
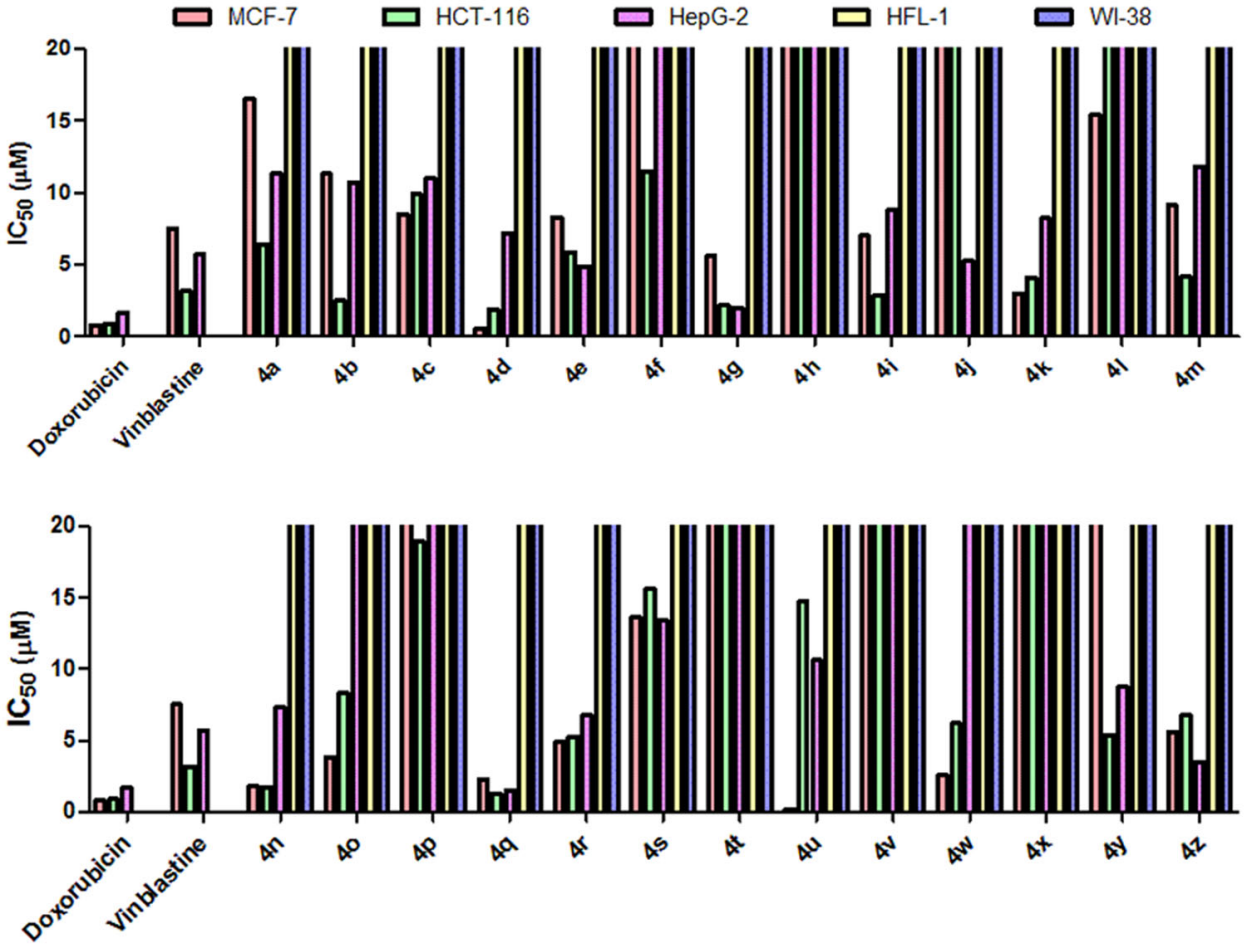
IC_50_ values expressed in (µM) of 1H-benzo[f]chromene derivatives (**4a–z**) against MCF-7, HCT, and HepG-2 tumour cells.

**Figure 3.**
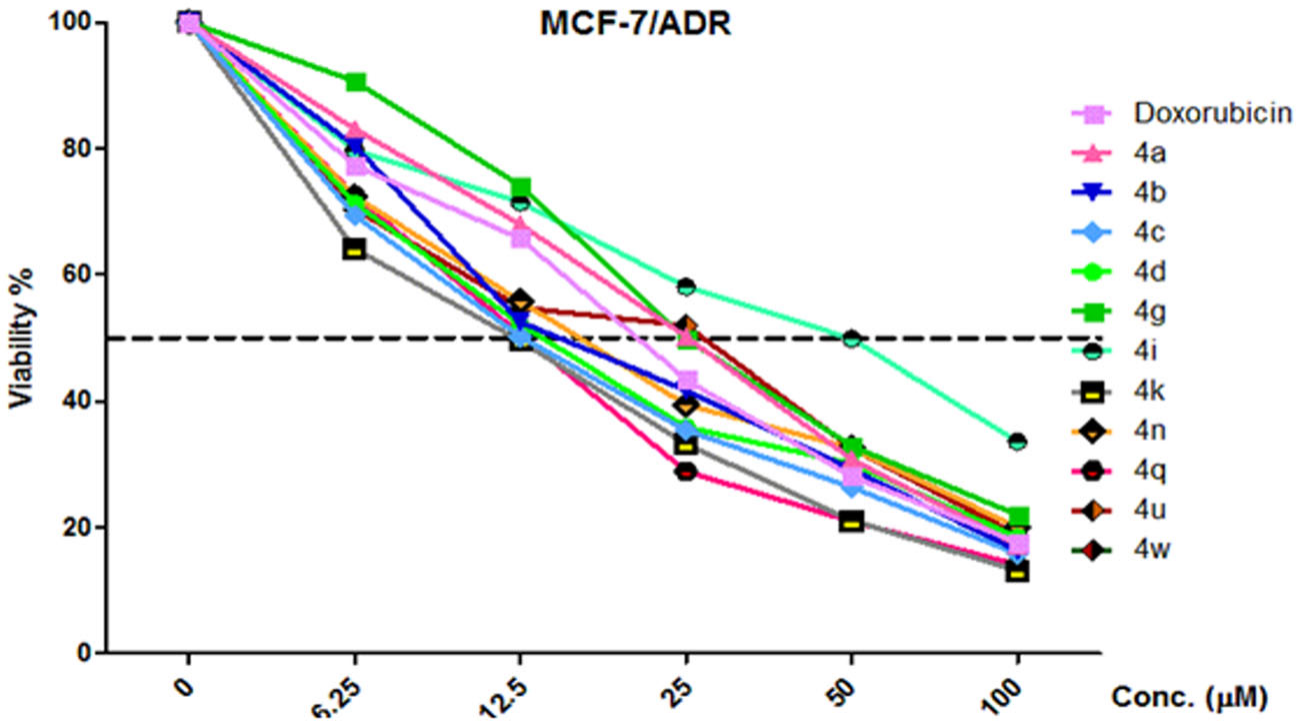
Dose-dependent cytotoxicity in MCF-7/ADR cells and the effect of varying concentrations of tested compounds **4a–4d, dg, 4i, 4k, 4n, 4q, 4u,** and **4w** on cell growth of MCF-7/ADR cells following exposure 24 h.

According to previous reports regarding the cytotoxic features of a numerous array of benzochromene scaffolds, compounds **4a − z** have been elected to explore their cytotoxic ability against three tumour cell lines, including HCT-116 (colon cancer), MCF-7 (breast cancer), and HepG-2 (hepatocellular cancer), and employing the 3–(4,5-dimethylthiazol-2-yl)-2,5-diphenyl tetrazolium bromide (MTT) colorimetric assay as described in literature^57^. Judges choice of such cell lines has been stimulated by the acknowledged antitumor performance of a substantial number of 1*H*-benzo[*f*]chromene and 4*H*-benzo[*h*]chromene molecules towards the asserted cell lines^18–35^. The *in-vitro* assay has been demonstrated utilising different concentrations (0–50 µM), where Doxorubicin and Vinblastine have been selected to be the reference drugs. Moreover, benzochromene derivatives with highly cytotoxic behaviour, **4a − d, 4g, 4i, 4k, 4n, 4q, 4 u,** and **4w**, are subjected to further inspection against ADR-resistant human breast cancer cells (MCF-7/ADR) and the two normal cell lines, HFL-1 and human diploid fibroblasts (WI-38). The attained data were declared as growth inhibitory concentration (IC_50_) values, which represent the compounds concentrations required to produce a 50% inhibition of cell growth after 24 h of incubation, compared to the untreated controls. The results revealed potent growth inhibitory activity against MCF-7, HCT-116, and HepG-2 cancer cells as shown in Figure 2. The results revealed potent growth inhibitory activity against MCF-7, HCT-116, and HepG-2 cancer cells; HFL-1 and WI-38 normal cell lines as shown in Figure 2, and MCF-7/ADR cancer cell as shown in Figure 3. Also, the results are illustrated in Table 4.

**Table 4.** Cytotoxic activity of the target compounds against MCF-7, HCT-116, HepG-2, MCF-7/ADR cancer cell lines and normal cell lines, HFL-1, WI-38.

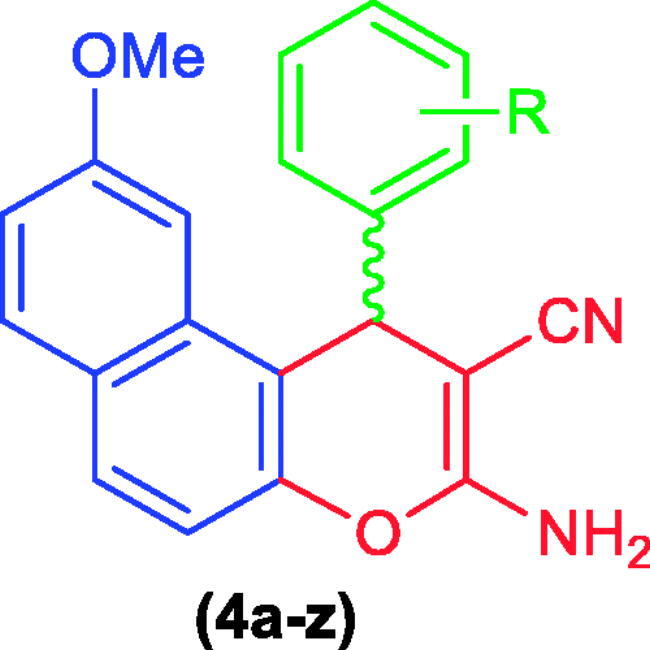							
IC_50_ µM^a^							
		Cancerotoxicity			Normotoxicity		
Cpd.	*R*	MCF-7	HCT-116	HepG-2	MCF-7/ADR	HFL-1	WI-38
**4a**	4-F	16.5 ± 0.1	6.4 ± 0.1	11.3 ± 0.1	24.4 ± 1.2	45.1 ± 0.2	56.7 ± 1.1
**4b**	2-Cl	11.3 ± 0.1	2.5 ± 0.2	10.7 ± 0.5	13.3 ± 0.5	71.9 ± 0.1	66.7 ± 0.2
**4c**	3-Cl	8.5 ± 0.6	9.9 ± 0.1	11.0 ± 0.2	10.9 ± 0.2	59.5 ± 1.1	61.5 ± 1.2
**4d**	4-Cl	0.6 ± 0.2	1.9 ± 0.4	7.2 ± 0.1	12.5 ± 0.3	77.1 ± 1.1	83.0 ± 1.1
**4e**	2-Br	8.3 ± 0.6	5.9 ± 0.4	4.9 ± 0.2	–	49.5 ± 1.1	59.1 ± 1.1
**4f**	3-Br	69.2 ± 0.3	11.5 ± 1.1	63.6 ± 3.2	–	–	–
**4g**	4-Br	5.6 ± 0.1	2.2 ± 0.1	2.0 ± 0.1	19.6 ± 1.0	65.3 ± 0.1	69.0 ± 1.1
**4h**	4-I	48.4 ± 0.1	27.7 ± 0.3	39.2 ± 0.2	–	–	–
**4i**	4-Me	7.0 ± 0.1	2.9 ± 0.1	8.8 ± 0.2	42.8 ± 2.4	71.6 ± 0.3	61.7 ± 0.1
**4j**	4-CF_3_	26.8 ± 1.8	20.2 ± 1.1	5.3 ± 0.1	–	–	–
**4k**	2,4-F_2_	3.0 ± 0.3	4.1 ± 0.2	8.2 ± 0.6	11.5 ± 0.6	59.6 ± 0.1	47.3 ± 0.2
**4l**	2,6-F_2_	15.4 ± 0.2	77.7 ± 0.3	28.5 ± 0.4	–	–	–
**4m**	2,3-Cl_2_	9.1 ± 0.2	42.0 ± 0.2	11.8 ± 0.2	–	–	–
**4n**	2,4-Cl_2_	1.8 ± 0.1	1.7 ± 0.2	7.3 ± 0.2	15.5 ± 0.2	71.7 ± 0.1	61.9 ± 0.3
**4o**	2,5-Cl_2_	3.8 ± 0.3	8.3 ± 0.2	46.6 ± 0.1	–	–	–
**4p**	2,6-Cl_2_	41.5 ± 0.1	18.9 ± 0.2	25.9 ± 0.3	–	–	–
**4q**	3,4-Cl_2_	2.3 ± 0.1	1.3 ± 0.1	1.5 ± 0.1	13.4 ± 0.4	75.8 ± 0.2	72.8 ± 0.4
**4r**	2,5-Br_2_	4.9 ± 0.5	5.3 ± 0.4	6.8 ± 0.5	–	38.1 ± 0.2	33.9 ± 0.4
**4s**	3,5-Br_2_	13.6 ± 0.2	15.0 ± 2.6	13.4 ± 1.1	–	–	–
**4t**	2-Cl-6-F	100.0 ± 1.3	96.5 ± 2.0	48.1 ± 1.3	–	–	–
**4u**	2-F-4-CF_3_	0.2 ± 1.3	14.7 ± 2.3	10.6 ± 0.9	19.7 ± 0.8	55.3 ± 0.1	60.3 ± 0.1
**4v**	2,4-(OMe)_2_	100.0 ± 0.2	49.9 ± 0.5	93.2 ± 0.2	–	–	–
**4w**	3,4-(OMe)_2_	2.6 ± 0.5	6.2 ± 0.3	52.3 ± 0.4	11.5 ± 0.6	47.1 ± 0.2	52.9 ± 0.3
**4x**	2,3,4-(OMe)_3_	96.1 ± 0.6	36.6 ± 0.1	59.7 ± 0.3	–	–	–
**4y**	3,4,5-(OMe)_3_	26.1 ± 0.1	15.4 ± 0.1	8.8 ± 0.2	–	34.1 ± 0.2	29.9 ± 0.4
**4z**	3,5-Br_2_-2-OMe	5.8 ± 0.04	6.8 ± 0.1	3.5 ± 0.2	–	33.3 ± 0.1	39.6 ± 0.4
**A**	–	7.5 ± 0.03	3.2 ± 0.1	5.7 ± 0.1	–	–	–
**B**	–	0.8 ± 0.01	0.9 ± 0.02	1.7 ± 0.4	18.6 ± 0.2	–	–

^a^IC_50_ values expressed in µM as the mean values of triplicate wells from at least three experiments and are reported as the mean ± standard error, A = Vinblastine and B = Doxorubicin.

Table 4 divulges that the 1*H*-benzo[*f*]chromene molecules **4d, 4 g, 4i, 4k, 4n, 4o, 4q, 4r, 4u, 4w,** and **4z** exhibited potent anti-proliferative character towards MCF-7 cell line with IC_50_ in the range of 0.2–7.0 µM. Molecules **4b, 4d, 4g, 4i, 4n,** and **4q** displayed superior activity against HCT-116 with IC_50_ in the range of 1.3–2.9 µM. Meanwhile, compounds **4e, 4 g, 4q,** and **4z** possessed growth inhibitory ability towards HepG-2 cell line with IC_50_ 1.5–4.9 µM. The remaining derivatives illustrated good, moderate, or inactive cytotoxic activity. Additionally, compounds **4a–e, 4 g, 4i, 4k, 4n, 4q, 4r, 4 u, 4w, 4y,** and **4z** demonstrated week growth inhibitory impact towards two normal cell lines, HFL-1 and WI-38 with IC_50_ values between 29.9 and 83.0 µg/mL. Furthermore, derivatives **4b–d, 4k, 4n, 4q,** and **4w** revealed good potency against MCF-7/ADR cell with IC_50_ values between 10.9 and 15.5 μM, as compared with Doxorubicin (IC_50_ = 18.6 μM), while compounds **4a, 4 g, 4i,** and **4u** are inactive against MCF-7/ADR cell with IC_50_ ranging from 19.6 to 42.8 μM. Finally, the remaining compounds exhibited fair cytotoxic behaviour towards the examined tumour cell types in comparison Doxorubicin.

### P-glycoprotein-mediated multidrug resistance in MCF-7/ADR cell

**Figure 4.**
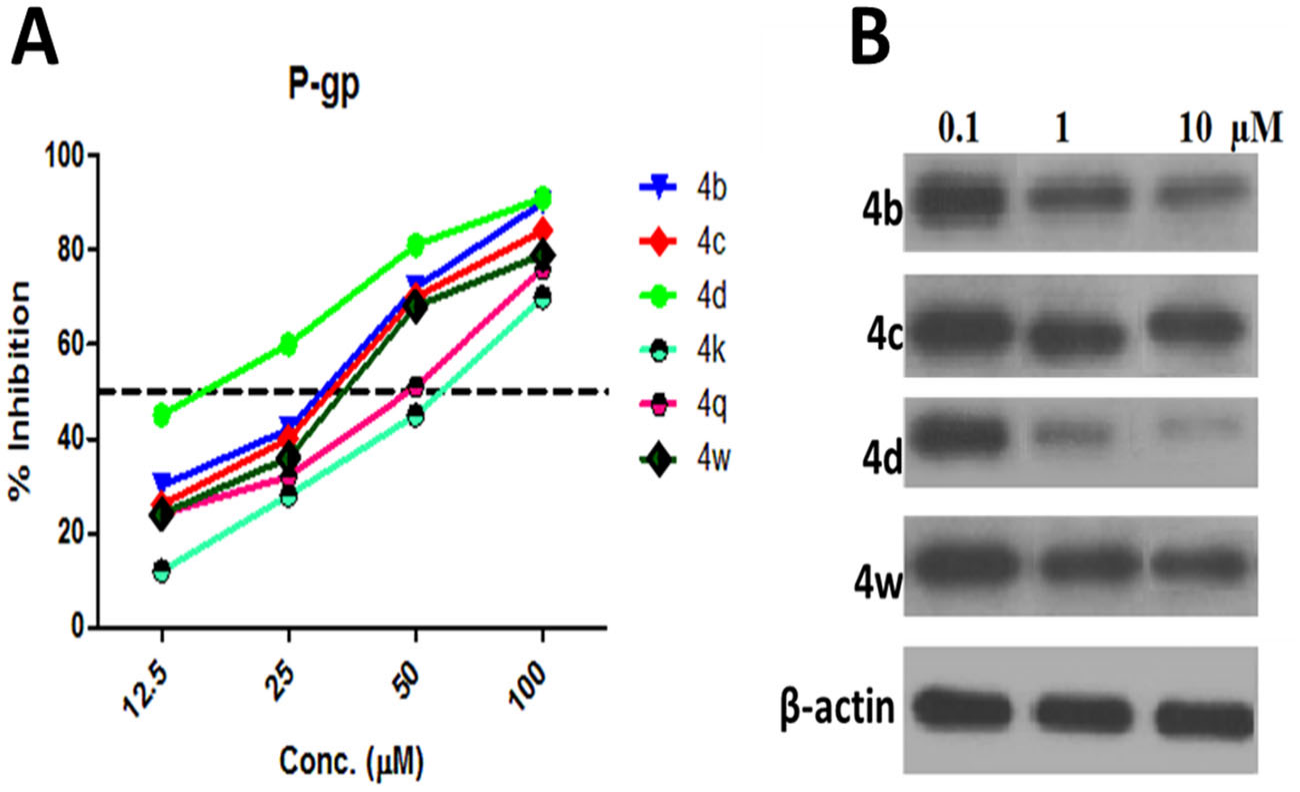
A. Inhibition of P-gp content in the lysate of MCF-7/ADR cell using varying conc. (12.5–100 µM) of tested compounds **4b–4d, 4k, 4q,** and **4w** follows exposure 48 h as determined by ELISA. B. Western blot analysis of P-gp expression in MCF-7/ADR cells after treatment with 0.1, 1.0, and 10.0 µM of compounds **4b–4d,** and **4w** for 48 h, the β-actin was used as a control.

*P*-gp pumps multiple types of drugs out of the cell, using energy generated from adenosine triphosphate (ATP), and confers MDR on cancer cells^58^. It is one of the noteworthy problems in malignant tumour clinical therapeutics^59^. The active compounds **4b–4d, 4k, 4q,** and **4w** against MCF-7/ADR lines were tested as possible inhibitory effect on *P*-gp inhibitors and the acquired results revealed that compounds **4b–4d, 4q,** and **4w** having 2-Cl, 3-Cl, 4-Cl, 3,4-Cl_2_, and 3,4-(OMe)_2_ substituents have good inhibitory potency against *P*-gp expression MDR in MCF-7/ADR with IC_50_ ranging from 13.5 to 45.3 μM as compared with Doxorubicin (IC_50_ = 50.9 μM), as shown in Figure 4(A) and Table 5.

**Table 5.** IC_50_ values of the active compounds **4b, 4c, 4d, 4q,** and **4w** against P-gp mediated MDR in MCF-7/ADR cells.

	%Reduction of *P-*gp expression				IC_50_ µM
Conc. (µM)	100	50	25	12.5	
4b	90	72	42	30	26.7
4c	84	70	40	26	28.4
4d	91	81	60	45	13.5
4k	70	45	28	12	61.4
4q	76	51	32	19	45.3
4w	79	68	36	24	32.4
Doxorubicin	–	–	–	–	50.9

The effect of compounds **4b–d, 4k,** and **4w** on the inhibition of *P*-gp expression in MCF-7/ADR was also confirmed using western blot analysis as shown in Figure 4(B). Compounds **4b–d, 4k, 4q,** and **4w** have strong cytotoxic effects on MCF-7/ADR cells; they also displayed excellent inhibitory performance on the *P*-gp content (Figure 4(A)) with the exception of compound **4k**. These results demonstrated that only compounds **4b–d** and **4w** possessed high inhibitory effect on the expression of *P*-gp which subsequently have a great potency in reversal the MDR in MCF-7/ADR, similar results have been reported previously^60^^,^^61^. Furthermore, the reversion of *P*-gp mediated multidrug resistance may be achieved by down-regulation of *P*-gp expression and or inhibition of *P*-gp efflux function^62^. For this reason, the effect of our synthesised drugs was tested for inhibitory potential of *P*-gp activity using a Rhodamine 123 Accumulation Assay (Rhodamine Competitive ELISA Kit) (Table 6).

**Table 6.** P-glycoprotein inhibitory potential (IC_50_ values) based on P-glycoprotein content in MCF-7/ADR cell lysate and rhodamine 123 accumulation assay.

Cpd.	MCF-7/ADR IC_50_ (µM)	*P*-gp IC_50_ (µM)	Rho123 IC_50_ (µM)
**4b**	13.3 ± 0.5	26.7	20.5
**4c**	10.9 ± 0.2	28. 4	23.0
**4d**	12.5 ± 0.3	13.5	10.6
**4k**	11.5 ± 0.6	61.4	57.8
**4q**	13.4 ± 0.4	45.3	39.1
**4w**	11.5 ± 0.6	32.4	25.6
**Doxorubicin**	18.6 ± 0.2	50.9	–
**Verapamil**	–	–	14.3

The data in Table 6 portrayed the (IC_50_) of Rhodamine 123 for evaluating the *P*-gp functional inhibition of compounds **4b–d,** and **4w** which ranging from 10.6 to 25.6 µM compared to 14.3 µM of the reference drug Verapamil. Compounds **4b–d** and **4w** had an impact on the restoration of the sensitivity to MCF-7/ADR cells by reducing not only the *P*-gp expression but also its function. On the other hand, compounds **4k** and **4q** exhibited cytotoxic activity against MDR which may be associated with different types of mechanisms such as genetic factors, growth factors, increased DNA repair capacity^63^.

### Cell cycle arrest in treated MCF-7/ADR cancer cells

**Figure 5.**
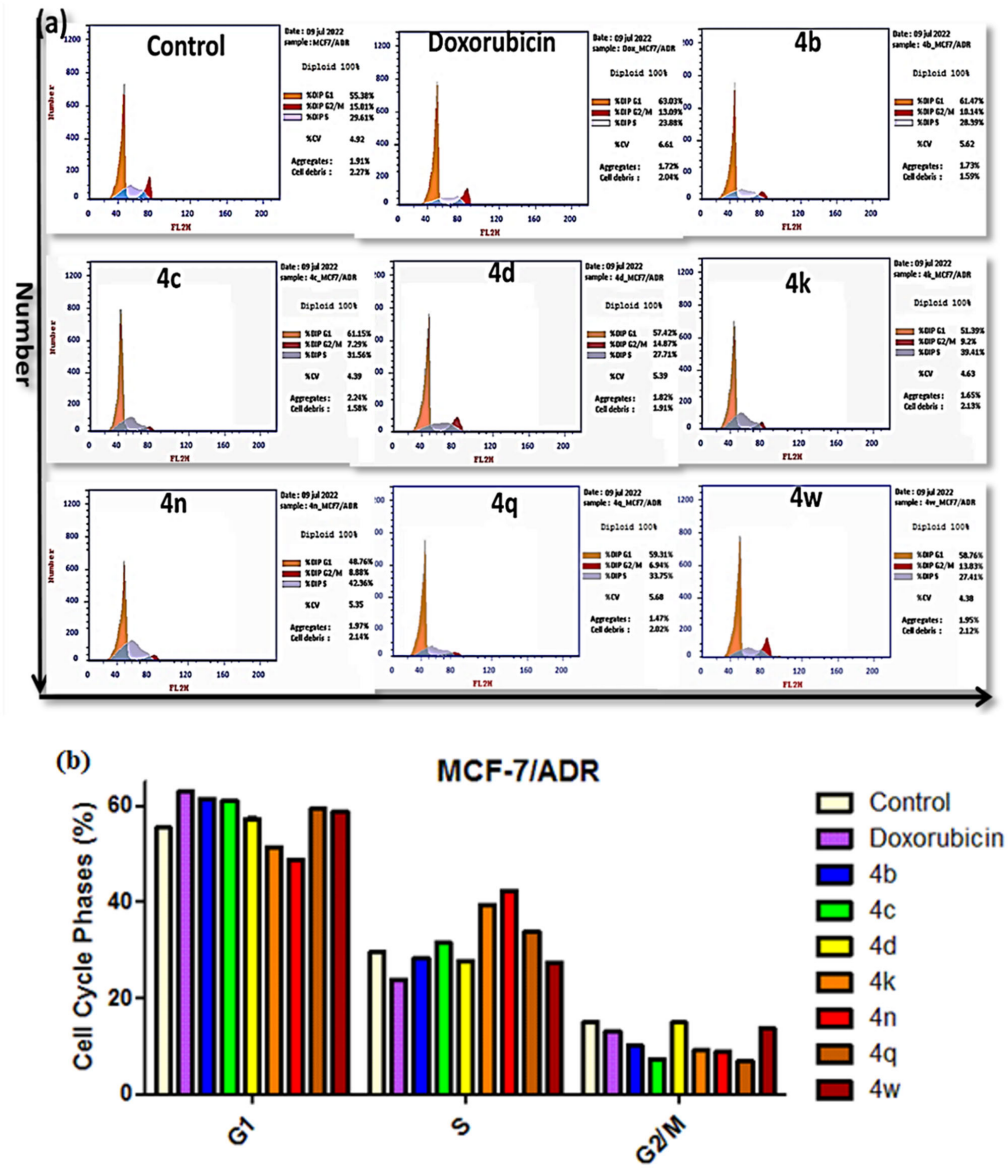
Effects of compounds **4 b–d, 4k, 4n, 4q,** and **4w** on the cell cycle phases of MCF-7/ADR cells. (a) Representative histograms of the DNA content distribution of cells were incubated with IC_50_ values for 24 h and stained with propidium iodide (PI). Their DNA content was analysed by the fluorescence flow cytometry. (b) The percentage of MCF-7/ADR cells in the G1, S, and G2/M phases after incubation with tested compounds (IC_50_ value) for 24 h. The data are expressed as the mean ± *SD* of three independent experiments in triplicate.

Development of anticancer agents targeting cell cycle arrest represents an important therapeutic intervention in treating diseases like cancer. Cancer cells undergo unscheduled cell divisions by the down regulation of the four cell cycle stages (G1, S, G2, and M) ^64^^,^^65^. *P*-gp breast cancer resistant proteins (BCRP) normally prevent intercellular drug accumulation, affecting the anticancer agent’s impact on the cell cycle arrest^66^. Therefore, the effect of the most potent newly synthesised compounds **4b–d, 4k, 4n, 4q,** and **4w** on regulating cell cycle progression of MCF-7/ADR cancer cells cycle was analysed by the flow cytometry, exploiting the FACS Calibres (Becton Dickinson). The distribution of cells along the G1 (2*n*), G2/M (4*n*), and S (2*n*–4*n*) phases of the cycle was exhibited in the representative cell cycle distribution histogram of the stained DNA in Figure 5(a). The MCF-7/ADR cancer cells were remedied with each derivative at its IC_50_ values for 24h, a controlled experiment with no treatment done.

The outcomes of the cell cycle progression showed that all the tested compounds have expressed a significant increased percentage of 10–15% (60–65%) of cells at the G1 phase in comparison to the (55%) control cells expect for compounds **4k** and **4n** which show increased percentages 40% and 43%, respectively, in S phase compared to the control (30%). In addition, these results were accompanied by a considerable decrease in percentage at the G2/M phases compared to the untreated control cells (Figure 5(b)). The cell cycle evaluation presented that the tested derivatives significantly arrested the cells’ progression by restricting the G1 and S phases.

### Apoptosis induction in MCF-7/ADR cancer cells

**Figure 6.**
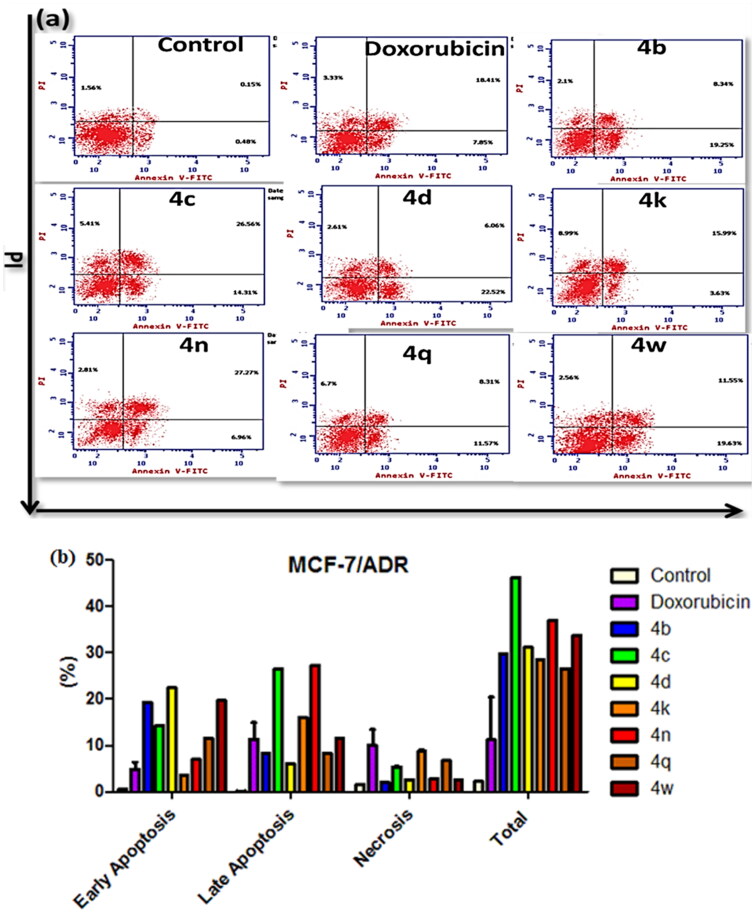
Apoptosis of MCF-7/ADR cells treated with compounds **4b–d, 4k, 4n, 4q,** and **4w**. (a) The dot plot of the Annexin V/PI stained cells, treated with the indicated drugs. (b) The apoptosis percentage of MCF-7/ADR cells after incubation with tested compounds (IC_50_ value) for 24 h. The data are expressed as the mean ± *SD* of three independent experiments in triplicate.

Several lines of evidence indicate that many anticancer compounds exerted their effects by blocking the cell cycle progression, by inducing apoptosis, or the combined effect of both^67^. Also, *P*-gp inhibits apoptosis by preventing the release of cytochrome c which is mediated by the intrinsic mitochondrial pathway^68^. To further assess the pivotal relation of the newly synthesised MCF-7/ADR anticancer compounds and apoptosis, phosphatidylserine (PS) translocation to the cell membrane as a marker for apoptosis was measured by the means of the Annexin V/PI double staining flow cytometric assay^69^. The representative dot plots of the double-stained MCF-7/ADR cells after treatment with the diverse examined compounds were displayed in Figure 6(a).

Unlike necrosis, which was not observed in all the results, all treated cells illustrated up to 40% in total apoptosis in evaluation against 30% and 2% of the Doxorubicin and untreated cells respectively. Moreover, all the tested compounds showed early (Annexin V positive, PI negative) as well as late (Annexin V positive, PI positive) apoptosis for all the treated cells (Figure 6(b)). Our results proposed that the induction of MCF-7/ADR cytotoxicity occurs *via* mechanisms associated with apoptosis with no obvious negative effects of the *P*-gp.

### Structure–activity relationship SAR

**Scheme 2.**
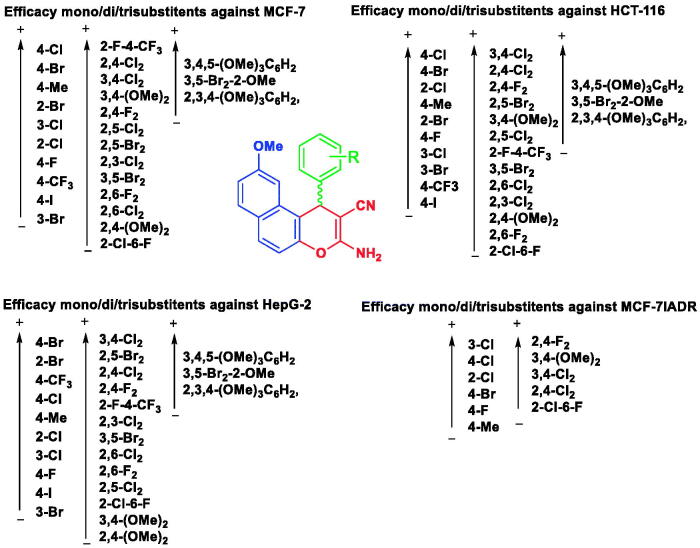
Schematic representation of structure–activity relationship.

The initial SAR investigations performed were centred on the impact of substituting hydrogen atoms on the phenyl group at 1-position of the 1*H*-benzo[*f*]chromene platform by halogen atoms, methyl or methoxy groups and methoxy group at 9-position as illustrate in Scheme 2. The implementation of the halogen or methoxy monosubstituted for the first series (**4a–j**) reduced their activities against MCF-7 cell line (IC_50_ in the range of 0.6–69.2 µM) as compared with Vinblastine. This behaviour could be attributed to the effect of the grafting of a lipophilic electron withdrawing or electron donating groups on the phenyl group at 1-position of the 1*H*-benzo[*f*]chromene moiety. Meanwhile, the second series with disubstituted halogens atoms or methoxy groups (**4k–w**) revealed a fluctuation in their antiproliferative activities with a simultaneous variation in the position and size of the disubstituted halogens or methoxy. Among the third series with the trisubstituents, compounds (**4x–z**), only compound (**4z**) possessed adequate activity in assessment with Vinblastine.

Regarding the influence of the substituent’s groups of the desired molecules from the first series on their potency against HCT-116 cell lines, the halogenated or methylated monosubstituent (**4a–j**) exhibited diminished IC_50_ in the range of 1.9–27.7 µM values, while for compounds (**4k–w**) the activities were in the range of 1.3–96.5 µM. Furthermore, through the trisubstituents of the third series (**4x–z**), only compound (**4z**) exhibited activity approximate to Vinblastine, while the other derivatives were inactive, intimating that the disubstituent demonstrated superior potency in evaluation with the mono- and tri-substituent analogues.

In addition, selected derivatives of the three series demonstrated superior potency against HepG-2 cell lines. For instance, compounds **4q, 4g, 4z, 4e,** and **4j** had IC_50_ values in the range of 1.5–5.3 µM in appraisal with Vinblastine and Doxorubicin. Also, compounds **4b–d, 4k, 4n, 4q,** and **4w** possessed good potency against resistant cell strains (MCF-7/ADR) with IC_50_ in the range of 10.9–15.5 μM. Such results tentatively suggested that grafting a lipophilic electron withdrawing with moderate size (difluoro or dichloro substituents) is more beneficial than other substituents for the activity. Furthermore, compounds **4a–e, 4g, 4i, 4k, 4n, 4q, 4r, 4u, 4w, 4 y,** and **4z** have been screened against two normal cell lines, HFL-1 and WI-38 and displayed IC_50_ ranging from 29.9 to 83.0 µM, which confirm their inadequate performance against these control cell lines. Finally, we can deduce that the position and the type of the substituent on the phenyl group at the 1-position of the 1H-benzo[f]chromene moiety played a vital role in its antitumor activity.

### Molecular docking

To account for the most profiling compounds **4b–d, 4q,** and **4w** against *P*-gp, molecular docking was conducted to investigate their possible interactions (PDB code 3G60)^70^ and theoretical model was utilised in all docking experiment. I-TASSER^71^ was used to generate Human *P*-gp, and then used AMBER force field to optimise their model. Ramachandran plots were obtained which showed that model is similar to that one gained from the experimental mouse structure, which reported in the protein data bank. We also predicted the crystal structure of mouse *P*-gp (code 3G60) by I-TASSER. In this docking analysis we used the *P*-gp structure and translocation of the substrate pore as a rigid object in the docking procedure. However, since the ligand-binding pose of *P*-gp (a facing inward closed contact to apo conformation) was employed, the docking data give insight in the suitable complex geometry to ATP hydrolysis. To validate the accuracy of the docking analysis we redocked the reference cyclic peptide “QZ59-RRR” bound (PDB code 3G60), and compared with original geometry for QZ59-RRR. The original QZ59-RRR was docked into the experimentally determined structure of mouse *P*-gp with a high accuracy with a RMSD value of 1.78 Å. To gain further validation for the docking experiment, the inhibition constant (Ki) and bioactivity factor also examined as ligand efficiency (LE) were calculated (Table 7). All investigated compounds and reference molecules appeared in acceptable range as listed in Table 7.

**Table 7.** The binding affinity for compounds **4a–z** and QZ59-RRR in (kcal/mol) against P-gp.

	ΔG	rmsd	H.B.	Int.	E_ele		ΔG	rmsd	H.B.	Int.	E_ele
**4a**	−9.032	1.087	−23.942	−20.046	−9.396	**4n**	−10.287	2.627	−40.442	−20.503	−9.315
**4b**	−11.056	1.187	−19.279	−24.376	−10.742	**4o**	−8.712	1.602	−29.282	−18.190	−9.297
**4c**	−11.090	0.725	−29.261	−23.533	−9.599	**4p**	−10.227	2.502	−30.100	−17.257	−9.302
**4d**	−11.313	1.462	4.162	−24.106	−9.757	**4q**	−11.147	1.163	4.366	−18.866	−9.821
**4e**	−8.489	1.769	−21.211	−17.199	−10.033	**4r**	−8.697	2.367	−45.575	−19.625	−9.838
**4f**	−9.150	2.239	−26.141	−17.131	−9.178	**4s**	−10.943	1.541	−23.512	−20.074	−9.571
**4g**	−8.288	1.055	−16.842	−16.464	−9.755	**4t**	−10.616	1.408	−31.202	−22.709	−10.503
**4h**	−7.997	3.358	−26.703	−15.968	−9.284	**4u**	−10.347	2.552	−28.926	−16.892	−8.573
**4i**	−8.408	2.069	−17.976	−19.505	−10.067	**4v**	−9.002	1.733	−42.449	−19.098	−8.492
**4j**	−10.111	1.580	−28.479	−18.606	−9.498	**4w**	−11.011	2.460	−33.716	−14.732	−8.412
**4k**	−10.796	2.390	−37.913	−16.912	−9.138	**4x**	−8.590	1.413	−23.069	−23.269	−9.656
**4l**	−10.397	1.068	−23.996	−21.129	−9.055	**4y**	−10.619	1.856	−1.805	−23.168	−9.866
**4m**	−10.699	2.583	−19.317	−19.348	−9.643	**4z**	−9.652	2.332	−27.569	−15.237	−8.609
**Dox.**	−7.448	1.559	131.239	−15.918	−10.203	**QZ59**	−8.373	1.768	89.393	−27.475	−8.461

Dox.: Doxorubicin, a reference molecule and QZ59: reference inhibitor for *P*-glycoprotein.

**Figure 7.**
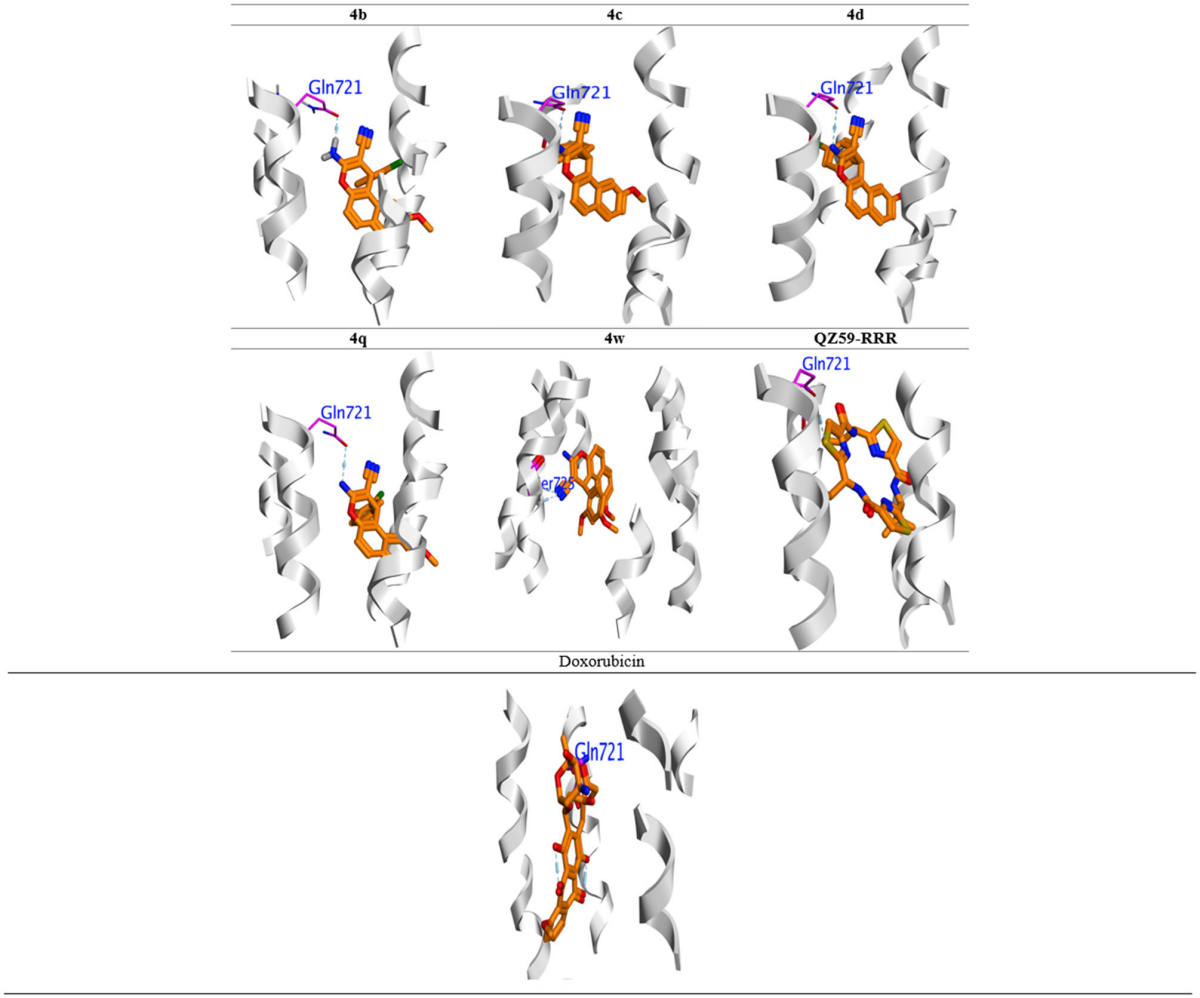
Binding mode of most active compounds, reference molecule (Doxorubicin), and QZ59-RRR into P-glycoprotein (PDB: 3G60). H-bonding represented in blue lines.

The docking experiment has been accomplished by Glide’s module^®^. The binding free energies ΔG are listed in Table 7. The original-inhibitors have been suitably fitted into their own binding sites for their crystal structures. The QZ59-RRR capped the 3G60 binding pocket through bounded with and formed the same H-bond with Gn721 and Ser725. All compounds **4b-d, 4q,** and **4w** and reference molecule (Doxorubicin) docked fruitfully into active sites in the same manner as original inhibitor through formation H-bond with Gln721 (Figure 7).

From Table 7, the most potential antiproliferative compounds **4b–d, 4k, 4q**, and **4w** showed high promising binding affinity compared to QZ59-RRR for the investigated *P*-gp (PDB: 3G60).

Compound **4d** showed the highest binding affinity Δ*G* = −11.313 kcal/mol against all investigated compounds and reference inhibitor, which explain the highest *P*-gp-inhibition activity (IC_50_ = 13.5 μM), through blocked active site by the formation of the H-bond with vital Gln 721. Besides, the other compounds **4b, 4c, 4k, 4q,** and **4w** showed higher ΔG than QZ59-RRR, which arranged in decreasing order **4c< 4b < 4q < 4w< 4k <** QZ59-RRR (Table 7). Compounds **4b, 4c, 4k,** and **4q** were occupied the binding pocket with the same behaviour of QZ59-RRR through engaged Gln721, only compound **4w** showed high stability in binding pocket through the interaction with important Ser725 (Figure 7).

## Conclusions

In continuation with our preceding endeavours in cultivating novel efficacious molecules, this report explores the design and synthesis of a series of oxygen-incorporating heterocyclic derivatives (**4a–z**) with the integration of a benzochromene moiety. Upon the evaluation of these derivatives for their cytotoxic behaviours against MCF-7, HCT-116, HepG-2, and MCF-7/ADR tumour cell lines in comparison with the standard reference drugs Vinblastine, and Doxorubicin, only molecules exhibiting cytotoxically active characteristics were selected for additional assessment against the representative tumour cell line ADR-resistant human breast cancer cells (MCF-7/ADR)) and two healthy cell lines (HFL-1 and human diploid fibroblasts (WI-38). Additionally, molecules **4b–d, 4k, 4n, 4q,** and **4w** which possessed good potency against MCF-7/ADR cell comparable to that of Doxorubicin were tested to show the possible inhibitory effect on *P*-gp expression and the obtained data concluded that **4b–d, 4q,** and **4w** possessed good inhibitory potency against *P*-gp expression in MCF-7/ADR cells. Furthermore, the western blot results and the Rh123 accumulation assays showed that compounds **4b–d,** and **4w** effectively inhibited *P*-gp expression and efflux function while, compounds **4b–d, 4k, 4n, 4q,** and **4w** induced accumulation of the treated MCF-7/ADR cells in the G1 phase and **4k** and **4n** additionally in the S phase of the cell cycle. Lastly, the SARs investigations of the molecules corroborated that the variation within the substitution on the phenyl ring of the 1*H*-benzo[*f*]chromene scaffold alongside the incidence of the methoxy moiety at the 9-position elevates molecular capabilities against these diverse cell lines.

## Experimental section

### Materials and equipments

All chemicals were purchased from Sigma-Aldrich Chemical Co. (Sigma-Aldrich Corp., St. Louis, MO). All melting points were measured with a Stuart Scientific Co. Ltd apparatus are uncorrected. The IR spectra were recorded on a KBr disc on a Jasco FT/IR 460 plus spectrophotometer. The ^1^H NMR (500 MHz) and ^13^C NMR (125 MHz) spectra were measured on BRUKER AV 500 MHz spectrometer in DMSO-d_6_ as a solvent, using tetramethylsilane (TMS) as an internal standard, and chemical shifts were expressed as *δ* (ppm). ^13^C-NMR spectra were obtained using distortion-free enhancement by polarisation transfer (DEPT) and the attached proton test (APT). The Ultrasonic apparatus used is Fisher Scientific UL TRASONIC CLEANER FS220. The mass spectra were determined on a Shimadzu GC/MS-QP5050A spectrometer. Elemental analysis was carried out at the Regional Centre for Mycology and Biotechnology (RCMP), Al-Azhar University, Cairo, Egypt, and the results were within ± 0.25%. Reaction courses and product mixtures were routinely monitored by thin-layer chromatography (TLC) on silica gel precoated F_254_ Merck plates.

### General procedure for synthesis of 1H-benzo[f]chromene derivatives (4a–z)

A mixture of 7-methoxynaphthalen-2-ol (**1**) (0.01 mol), different aromatic aldehydes (**2a–z**) (0.01 mol), malononitrile (**3**) (0.01 mol), and piperidine (0.5 ml) in ethanol (30 ml) was equipped with Ultrasonic probe under the power of 60 W at room temperature. After completion of the reaction, as indicated by TLC (*n*-hexane/ethyl acetate 1:3), the reaction mixture was cooled to ambient temperature and the precipitated solid was filtered off, washed with methanol, and was recrystallised from ethanol. The physical and spectral data of compounds **4a–z** are as follows:

### 3-Amino-1–(4-fluorophenyl)-9-methoxy-1H-benzo[f]chromene-2-carbonitrile (4a)

Pale yellow crystals; yield 92%; m.p. 267–268 °C (Literature procedure, reflux condition, yield 89%; m.p. 265–266 °C) ^72^.

### 3-Amino-1–(2-chlorophenyl)-9-methoxy-1H-benzo[f]chromene-2-carbonitrile (4b)

Colourless needles; yield 90%; m.p. 266–267 °C (Literature procedure, reflux condition, yield 82%; m.p. 265–266 °C) ^28^.

### 3-Amino-1–(3-chlorophenyl)-9-methoxy-1H-benzo[f]chromene-2-carbonitrile (4c)

Colourless needles; yield 89%; m.p. 262–263 °C (Literature procedure, reflux condition, yield 86%; m.p. 260–261 °C) ^28^.

### 3-Amino-1–(4-chlorophenyl)-9-methoxy-1H-benzo[f]chromene-2-carbonitrile (4d)

Colourless needles; yield 92%; m.p. 257–258 °C (Literature procedure, reflux condition, yield 89%; m.p. 257–258 °C) ^72^.

### 3-Amino-1–(2-bromophenyl)-9-methoxy-1H-benzo[f]chromene-2-carbonitrile (4e)

Colourless crystals; yield 90%; m.p. 273–275 °C; IR (KBr) *υ* (cm^−1^): 3423, 3338, 3202 (NH_2_), 2180 (CN); ^1^H NMR *δ*: 7.89–6.83 (m, 11H, aromatic and NH_2_), 5.54 (s, 1H, H-1), 3.72 (s, 3H, OMe); ^13^C NMR *δ*: 160.14 (C-3), 158.69 (C-9), 148.03 (C-4a), 144.76 (C-1, Ar), 135.99 (C-3, Ar), 134.10 (C-6a), 132.99 (C-6, Ar), 132.10 (C-10a), 130.65 (C-4, Ar), 129.94 (C-5, Ar and C-6), 129.26 (C-10), 126.42 (C-8), 122.54 (C-7), 120.19 (C-2, Ar), 117.49 (C-10b), 114.56 (CN), 56.93 (C-2), 102.79 (C-5), 55.76 (CH_3_), 35.76 (C-1); MS *m/z* (%): 408 (M^+^+2, 16.92), 406 (M^+^, 1754) with a base peak at 203 (100); Anal. Calcd for C_21_H_15_BrN_2_O_2_ (407.26): C, 61.93; H, 3.71; N, 6.88. Found: C, 62.00; H, 3.78; N, 6.95%.

### 3-Amino-1–(3-bromophenyl)-9-methoxy-1H-benzo[f]chromene-2-carbonitrile (4f)

Pale yellow crystals; yield 93%; m.p. 263–265 °C; IR (KBr) *υ* (cm^−1^): 3438, 3322, 3200 (NH_2_), 2176 (CN); ^1^H NMR *δ*: 7.88–6.69 (m, 11H, aromatic and NH_2_), 5.36 (s, 1H, H-1), 3.75 (s, 3H, OMe); ^13^C NMR *δ*: 160.26 (C-3), 158.53 (C-9), 148.90 (C-4a), 147.76 (C-1, Ar), 131.98 (C-10a), 131.38 (C-2, Ar), 130.45 (C-5, Ar), 129.97 (C-4, Ar), 128.78 (C-6, Ar and C-6), 126.81 (C-7), 126.42 (C-6a), 122.24 (C-3, Ar), 120.82 (C-10b), 117.41 (C-5), 114.69 (C-8), 114.50 (CN), 103.53 (C-10), 57.75 (C-2), 55.55 (CH_3_), 37.77 (C-1); MS *m/z* (%): 408 (M^+^+2, 38.57), 406 (M^+^, 39.59) with a base peak at 337 (100); Anal. Calcd for C_21_H_15_BrN_2_O_2_ (407.26): C, 61.93; H, 3.71; N, 6.88. Found: C, 61.83; H, 3.65; N, 6.81%.

### 3-Amino-1–(4-bromophenyl)-9-methoxy-1H-benzo[f]chromene-2-carbonitrile (4g)

Colourless crystals; yield 90%; m.p. 252–253 °C (Literature procedure, reflux condition, yield 87%; m.p. 251–252 °C) ^72^.

### 3-Amino-1–(4-iodophenyl)-9-methoxy-1H-benzo[f]chromene-2-carbonitrile (4h)

Yellow crystals; yield 93%; m.p. 227–228 °C (Literature procedure, reflux condition, yield 89%; m.p. 226–227 °C) ^28^.

### 3-Amino-1–(4-methylphenyl)-9-methoxy-1H-benzo[f]chromene-2-carbonitrile (4i)

Yellow crystals; yield 94%; m.p. 245–246 °C (Literature procedure, reflux condition, yield 88%; m.p. 244–245 °C) ^72^.

### 3-Amino-1–(4-trifluoromethylphenyl)-9-methoxy-1H-benzo[f]chromene-2-carbonitrile (4j)

Yellow crystals; yield 91%; m.p. 212–214 °C; IR (KBr) *υ* (cm^−1^): 3402, 3337, 3252 (NH_2_), 2204 (CN); ^1^H NMR *δ*: 8.96–7.02 (m, 11H, aromatic and NH_2_), 5.17 (s, 1H, H-1), 3.75 (s, 3H, OMe); MS *m/z* (%): 396 (M^+^, 22.79) with a base peak at 70 (100); Anal. Calcd for C_22_H_15_F_3_N_2_O_2_ (396.36): C, 66.67; H, 3.81; N, 7.07. Found: C, 66.75; H, 3.88; N, 7.13%.

### 3-Amino-1–(2,4-difluorophenyl)-9-methoxy-1H-benzo[f]chromene-2-carbonitrile (4k)

Yellow crystals; yield 89%; m.p. 255–256 °C (Literature procedure, reflux condition, yield 87%; m.p. 255–256 °C) ^28^.

### 3-Amino-1–(2,6-difluorophenyl)-9-methoxy-1H-benzo[f]chromene-2-carbonitrile (4l)

Colourless crystals; yield 91%; m.p. 305–306 °C (Literature procedure, reflux condition, yield 88%; m.p. 305–306 °C) ^28^.

### 3-Amino-1–(2,3-dichlorophenyl)-9-methoxy-1H-benzo[f]chromene-2-carbonitrile (4m)

Colourless crystals; yield 93%; m.p. 310–311 °C (Literature procedure, reflux condition, yield 87%; m.p. 310–311 °C) ^28^.

### 3-Amino-1–(2,4-dichlorophenyl)-9-methoxy-1H-benzo[f]chromene-2-carbonitrile (4n)

Colourless crystals; yield 90%; m.p. 261–262 °C (Literature procedure, reflux condition, yield 87%; m.p. 260–261 °C) ^28^.

### 3-Amino-1–(2,5-dichlorophenyl)-9-methoxy-1H-benzo[f]chromene-2-carbonitrile (4o)

Colourless crystals; yield 90%; m.p. 268–269 °C (Literature procedure, reflux condition, yield 82%; m.p. 268–269 °C) ^28^.

### 3-Amino-1–(2,6-dichlorophenyl)-9-methoxy-1H-benzo[f]chromene-2-carbonitrile (4p)

Colourless crystals; yield 88%; m.p. 321–322 °C (Literature procedure, reflux condition, yield 86%; m.p. 320–321 °C) ^28^.

### 3-Amino-1–(3,4-dichlorophenyl)-9-methoxy-1H-benzo[f]chromene-2-carbonitrile (4q)

Colourless crystals; yield 90%; m.p. 255–256 °C (Literature procedure, reflux condition, yield 86%; m.p. 255–256 °C) ^28^.

### 3-Amino-1–(2,5-dibromophenyl)-9-methoxy-1H-benzo[f]chromene-2-carbonitrile (4r)

Pale yellow crystals; yield 89%; m.p. 260–262 °C; IR (KBr) *υ* (cm^−1^): 3410, 3291, 3209 (NH_2_), 2191 (CN); ^1^H NMR *δ*: 7.92–6.79 (m, 10H, aromatic and NH_2_), 5.53 (s, 1H, H-1), 3.72 (s, 3H, OMe); ^13^C NMR *δ*: 160.22 (C-3), 158.82 (C-9), 148.04 (C-4a), 146.94 (C-1, Ar), 135.27 (C-6, Ar), 132.82 (C-10a), 132.30 (C-3, Ar), 131.91 (C-4, Ar), 130.87 (C-6), 130.38 (C-7), 126.42 (C-6a), 122.01 (C-5, Ar), 121.60 (C-10b, C-2, Ar), 117.65 (C-5), 114.60 (CN), 113.54 (C-8), 102.61 (C-10), 56.22 (C-2), 55.77 (CH_3_), 31.91 (C-1); MS *m/z* (%):488 (M^+^+4, 8.13), 486 (M^+^+2, 15.53), 484 (M^+^, 8.85) with a base peak at 89 (100); Anal. Calcd for C_21_H_14_Br_2_N_2_O_2_ (486.16): C, 51.88; H, 2.90; N, 5.76. Found: C, 51.80; H, 2.82; N, 5.70%.

### 3-Amino-1–(3,5-dibromophenyl)-9-methoxy-1H-benzo[f]chromene-2-carbonitrile (4s)

Pale yellow crystals; yield 90%; m.p. 268–270 °C; IR (KBr) *υ* (cm^−1^): 34105, 3295, 3212 (NH_2_), 2196 (CN); ^1^ H NMR *δ*: 7.92–7.03 (m, 10H, aromatic and NH_2_), 5.46 (s, 1H, H-1), 3.81 (s, 3H, OMe); ^13^C NMR *δ*: 160.45 (C-3), 158.72 (C-9), 150.79 (C-4a), 147.91 (C-1, Ar), 132.24 (C-10a), 131.91 (C-2,6, Ar), 130.65 (C-4, Ar), 130.12 (C-6), 129.74 (C-7), 126.47 (C-6a), 123.18 (C-3,5, Ar), 120.67 (C-10b), 117.61 (C-5), 114.59 (CN), 114.08 (C-8), 103.41 (C-10), 57.20 (C-2), 55.65 (CH_3_), 32.24 (C-1). In ^13^C NMR-APT spectrum: CH, CH_3_ [positive (up)] and CH_2_, Cq [negative (down)], revealed the following signals at *δ*: 160.45 (C-3 ↓), 158.72 (C-9 ↓), 150.79 (C-4a ↓), 147.91 (C-1, Ar ↓), 132.24 (C-10a ↓), 131.91 (C-2,6, Ar ↑), 130.65 (C-4, Ar ↓), 130.12 (C-6 ↑), 129.74 (C-7 ↑), 126.47 (C-6a ↓), 123.18 (C-3,5, Ar ↓), 120.67 (C-10b ↓), 117.61 (C-5 ↑), 114.59 (CN ↓), 114.08 (C-8 ↑), 103.41 (C-10 ↑), 57.20 (C-2 ↓), 55.65 (CH_3_ ↑), 32.24 (C-1 ↑); MS *m/z* (%): 488 (M^+^+4, 18.83), 486 (M^+^+2, 37.81), 484 (M^+^, 19.82) with a base peak at 176 (100); Anal. Calcd for C_21_H_14_Br_2_N_2_O_2_ (486.16): C, 51.88; H, 2.90; N, 5.76. Found: C, 51.96; H, 2.97; N, 5.84%.

### 3-Amino-1–(2-chloro-6-fulorophenyl)-9-methoxy-1H-benzo[f]chromene-2-carbonitrile (4t)

Pale yellow crystals; yield 94%; m.p. 292–294 °C; IR (KBr) *υ* (cm^−1^): 3448, 3328, 3256 (NH_2_), 2174 (CN); ^1^H NMR *δ*: 7.96–7.02 (m, 10H, aromatic and NH_2_), 5.17 (s, 1H, H-1); MS *m/z* (%): 382 (M^+^+2, 15.20), 380 (M^+^, 23.99) with a base peak at 102 (100); Anal. Calcd for C_21_H_14_ClFN_2_O_2_ (380.80): C, 66.24; H, 3.71; N, 7.36. Found: C, 66.19; H, 3.65; N, 7.30%.

### 3-Amino-1–(2-fluoro-6-trifuloromethylphenyl)-9-methoxy-1H-benzo[f]chromene-2-carbonitrile (4u)

Pale yellow crystals; yield 90%; m.p. 237–239 °C; IR (KBr) *υ* (cm^−1^): 3443, 3325, 3261 (NH_2_), 2200 (CN); ^1^H NMR *δ*: 7.86–6.96 (m, 10H, aromatic and NH_2_), 5.55 (s, 1H, H-1), 3.70 (s, 3H, OMe); ^13^C NMR *δ*: 160.70 (C-3), 158.65 (C-2, Ar), 153.65 (C-9), 147.98 (C-4a), 137.11 (C-10a), 136.82 (C-6, Ar), 133.82 (C-4, Ar), 131.82 (C-6), 130.64 (C-1, Ar), 129.99 (C-7), 126.28 (C-6a), 122.28 (CF_3_); 120.49 (C-10b, C-3,5, Ar), 117.63 (C-5), 114.45 (CN), 112.43 (C-8), 102.43 (C-10), 58.65 (C-2), 55.40 (CH_3_), 33.20 (C-1); MS *m/z* (%): 414 (M^+^, 31.03) with a base peak at 106 (100); Anal. Calcd for C_22_H_14_F_4_N_2_O_2_ (414.35): C, 63.77; H, 3.41; N, 6.76. Found: C, 63.71; H, 3.03; N, 6.70%.

### 3-Amino-1–(2,4-dimethoxyphenyl)-9-methoxy-1H-benzo[f]chromene-2-carbonitrile (4v)

Pale yellow crystals; yield 89%; m.p. 218–220 °C (Literature procedure, reflux condition, yield 80%; m.p. 205–206 °C)^72^ IR (KBr) *υ* (cm^−1^): 3444, 3328, 3267 (NH_2_), 2199 (CN); ^1^H NMR *δ*: 7.78–6.26 (m, 10H, aromatic and NH_2_), 5.36 (s, 1H, H-1), 3.81 (s, 3H, OMe); ^13^C NMR *δ*: 160.38 (C-3), 159.58 (C-2, Ar), 158.40 (C-4, Ar), 156.90 (C-9), 147.86 (C-4a), 132.18 (C-10a), 130.43 (C-6, Ar), 129.98 (C-6), 129.04 (C-7), 126.35 (C-6a), 126.24 (C-10b), 121.044 (C-5), 117.24 (C-8), 115.77 (CN), 114.49 (C-1, Ar), 106.27 (C-10), 102.64 (C-,5, Ar), 98.53 (C-3, Ar), 57.91 (C-2), 56.37 (CH_3_), 55.49 (CH_3_), 55.29 (CH_3_), 31.23 (C-1); MS *m/z* (%): 388 (M^+^, 37.08) with a base peak at 102 (100); Anal. Calcd for C_23_H_20_N_2_O_4_ (388.42): C, 71.12; H, 5.19; N, 7.21. Found: C, 71.19; H, 5.25; N, 7.27%.

### 3-Amino-1–(3,4-dimethoxyphenyl)-9-methoxy-1H-benzo[f]chromene-2-carbonitrile (4w)

Yellow crystals; yield 88%; m.p. 253–255 °C (Literature procedure, reflux condition, yield 79%; m.p. 195–196 °C)^51^ IR (KBr) *υ* (cm ^−1^): 3365, 3324, 3293 (NH_2_), 2191 (CN); ^1^H NMR *δ*: 7.86–6.56 (m, 10H, aromatic and NH_2_), 5.21 (s, 1H, H-1), 3.74 (s, 3H, OMe), 3.65 (s, 6H, 2OMe); ^13^C NMR *δ*: 159.98 (C-3), 158.36 (C-9), 148.97 (C-4a), 147.84 (C-3, Ar), 147.39 (C-4, Ar), 138.93 (C-10a), 132.23 (C-1, Ar), 130.38 (C-6), 129.37 (C-7), 126.41 (C-6a), 121.09 (C-6, Ar), 119.75 (C-10b), 117.28 (C-5), 115.59 (C-8), 114.48 (CN), 112.62 (C-,5, Ar), 111.80 (C-2, Ar), 103.69 (C-10), 58.63 (C-2), 55.90 (CH_3_), 55.54 (CH_3_), 32.23 (C-1); MS *m/z* (%): 388 (M^+^, 37.08) with a base peak at 106 (100); Anal. Calcd for C_23_H_20_N_2_O_4_ (388.42): C, 71.12; H, 5.19; N, 7.21. Found: C, 71.07; H, 5.13; N, 7.15%.

### 3-Amino-1–(2,3,4-trimethoxyphenyl)-9-methoxy-1H-benzo[f]chromene-2-carbonitrile (4x)

Colourless crystals; yield 89%; m.p. 239–240 °C (Literature procedure, reflux condition, yield 84%; m.p. 239–240 °C) ^28^.

### 3-Amino-1–(3,4,5-trimethoxyphenyl)-9-methoxy-1H-benzo[f]chromene-2-carbonitrile (4y)

Colourless crystals; yield 89%; m.p. 260–261 °C (Literature procedure, reflux condition, yield 84%; m.p. 259–260 °C) ^28^.

### 3-Amino-1–(3,5-dibromo-2-methoxyphenyl)-9-methoxy-1H-benzo[f]chromene-2-carbonitrile (4z)

Colourless crystals; yield 92%; m.p. 263–264 °C (Literature procedure, reflux condition, yield 86%; m.p. 263–264 °C^28^).

### X-ray crystallography analysis

The compounds **4i** was obtained as single crystals by slow evaporation from ethanol solution of the pure compound at room temperature. Data were collected on a Bruker APEX-II D8 Venture area diffractometre, equipped with graphite monochromatic Mo Kα and Cu Kα radiations at 293 (2) K. Cell refinement and data reduction were carried out by Bruker SAINT. SHELXTL-2018/3^73^^,^^74^ was used to solve structure. The final refinement was carried out by full-matrix least-squares techniques with anisotropic thermal data for nonhydrogen atoms on *F*. CCDC 2132096 contain the Supplementary crystallographic data for this compound can be obtained free of charge from the Cambridge Crystallographic Data Centre *via* www.ccdc.cam.ac.uk/data_request/cif.

### Biological screening

#### Cell culture

The tumour cell lines MCF-7, HepG-2, and PC-3 were obtained from the American Type Culture Collection (ATCC, Rockville, MD). The cells were grown on RPMI-1640 medium supplemented with 10% inactivated foetal calf serum and 50 µg/mL gentamycin. The cells were maintained at 37 °C in a humidified atmosphere with 5% CO_2_ and were subculture two to three times a week.

#### Cytotoxicity evaluation using viability assay

The cytotoxic activity was appraised, using the 3–(4,5-dimethylthiazol-2-yl)-2,5-diphenyl-tetrazolium bromide (MTT) colorimetric assay, as reported previously^57^ and ELISA assay for MCF-7/ADR^75^.

#### Analysis of P-glycoprotein

The content of *P*-gp in the MCF-7/ADR cell lysates after incubation with varying conc. (12.5–100 µM) of tested compounds **4b, 4c, 4d, 4q,** and **4w** following exposure for 48 h. was determined using commercial human *P*-gp ELISA Kit (MBS2506188, MyBioSource Inc., San Diego, CA). Absorption was recorded at 450 nm with a Spectramax Gemini fluorescence microplate reader (Molecular Devices, Sunnyvale, CA) ^76^.

#### Rhodamine 123 accumulation assay

*P*-gp activity was determined by measuring intracellular accumulation of Rhodamine 123 in MCF-7/ADR cells in the absence or presence of compounds **4b–4d, 4k,** and **4w**, according to commercial Rhodamine Competitive ELISA Kit (AKR-5142, Cell Biolabs Inc., San Diego, CA) which provides a convenient method for the detection of total Rhodamine in extracts from cells^77^. Briefly, resistant cancer cells were harvested, washed twice, and counted. After dilution to 1 × 10^6^ cells/mL in 6 well plate each well, 1 mL of fresh media containing different concentrations of compounds **4b–4d, 4k,** and **4w** were added and incubated at 37 °C for 4 h in an atmosphere containing 5% CO_2_. Subsequently, 5.25 μM of Rho123 was added to each well and the wells were incubated for another 30 min at 37 °C. Finally, cells were washed, lysed as described before and intracellular quantification levels of rhodamine 123 were further analysed according to ELISA protocol Kit. Absorbance at 450 nm of each well was measured using Spectramax Gemini fluorescence microplate reader (Molecular Devices, Sunnyvale, CA). The total content of Rhodamine in each sample was determined by comparison with a Rhodamine standard curve.

#### Western blot analysis

Cellular protein extracts of cell lysates and western blotting were prepared after treatment of MCF-7/ADR cells with different compounds **4b–4d** and **4w** conc. (0.1, 1.0, and 10 μM) for 48 h as already described^78^. Cells were washed twice with ice-cold phosphate-buffered saline and total cell lysates were collected in sodium dodecyl sulphate (SDS) sample buffer. Cell lysates, containing equal amounts of protein, were separated by SDS–polyacrylamide gel electrophoresis (PAGE) and transferred to Hybond enhanced chemiluminescence nitrocellulose membrane (Amersham Biosciences, NJ). After being blocked in 5% non-fat milk in Tris-buffered saline with 0.1% Tween 20 (pH 7.6), membranes were incubated with the appropriate primary antibodies at 4 °C, overnight, and exposed to the appropriate secondary antibody for 3 h at room temperature. Finally, enzyme-linked chemiluminescence was visualised according to the ECL kit (Thermal Fisher, Waltham, MA) protocol. *β*-Actin was used to confirm equal loading in each lane in the samples prepared from cell lysates.

#### Cell cycle assay

Cell cycle arrest and distribution were done using Propidium Iodide Flow Cytometry Kit (ab139418, Abcam, Cambridge, UK) as previously described^79^. Briefly, MCF-7/ADR cancer cells at 1 × 104 cells were cultured in 60-mm dishes in the presence of various tested compounds with a concentration equal to the IC_50_ value for 24 h. Cells were collected and washed with PBS, fixed with precooled 70% ethanol at 4 °C. Staining went along in PBS containing 40 μg/ml RNase A and 10 μg/mL propidium iodide (PI) in the dark for 15 min. The DNA content in each cell nucleus was determined by a FACS Calibur flow cytometer (BD Biosciences, Franklin Lakes, NJ). Finally, Cell cycle phase distribution was analysed using Cell Quest Pro software (BD Biosciences) showing collected PI fluorescence intensity on FL2.

#### Annexin V-FITC apoptosis assay

Apoptosis assay was performed with an Annexin V-FITC/PI double staining apoptosis detection kit (K101, Biovison, Milpitas, CA) using a flow cytometer^80^. MCF-7/ADR cancer cells treated with different newly synthesised compounds (IC_50_ value) were harvested by trypsinization, washed twice with 4 °C PBS, and re-suspended in binding buffer. Annexin V-FITC and PI solutions were then added to stain the cells before analysis by flow cytometry A minimum of 10,000 cells per sample were acquired. Annexin V-FITC binding (FL1) and PI (FL2) were analysed using Cell Quest Pro software (BD Biosciences).

### Statistics

All data were expressed as the means ± standard deviation (SD), from at least three independent experiments with similar results. Statistical analysis and figures were performed by Graph Pad Prism version 5.01 (Graph Pad software, San Diego, CA).

### Molecule docking

The docking experiment was employed according to previous work^81^, the 3D model of *P*-gp was obtained from Swiss-Model^82^. Finally, the outcomes for docking were achieved by PyMol software^83^.

## Supplementary Material

Supplemental Material
