# Supplementary material for: Discovery of benzochromene derivatives first example with dual cytotoxic activity against the resistant cancer cell MCF-7/ADR and inhibitory effect of the P-glycoprotein expression levels
Source: J Enzyme Inhib Med Chem. 2023 Jan 20;38(1):2155814. doi: 10.1080/14756366.2022.2155814 (PMC9869995; doi:10.1080/14756366.2022.2155814)
Supplement: Supplemental Material [file IENZ_A_2155814_SM3115.pdf]

Supplementary Material

Discovery of Benzochromene Derivatives First Example as dual potent cytotoxic activity against the resistant cancer cell MCF-7/ADR and inhibitory effect of the *P*-Glycoprotein levels

Lali M. Al-Harbi [a], Eman A. Al-Harbi [b], Rawda M. Okasha [b], R.A. El-Eisawy [c,d], Mohammed A.A. El-Nassag [c], Hany M. Mohamed [c,e], Ahmed M. Fouda [f], Ahmed A. Elhenawy [c,g], Ahmed Mora [c], Ahmed M. El-Agrody [c,*], Heba K.A. El-Mawgoudg [h]

[a] *Chemistry Department, Faculty of Science, King Abdul-AzizUniversity, P.O. Box 80203, Jeddah 21589, Saudi Arabia*

[b] *Chemistry Department, Faculty of Science, Taibah University, Medina 30002, Saudi Arabia*

[c] *Chemistry Department, Faculty of Science, Al-Azhar University, Nasr City 11884, Cairo, Egypt*

[d] *Chemistry Department, Faculty of Science and Art, Al-Baha University, Al-Baha, 1988, Saudi Arabia*

[e] *Chemistry Department, Faculty of Science, Jazan University, Jazan 45142, Saudi Arabia*

[f] *Chemistry Department, Faculty of Science, King Khalid University, Abha 61413, Saudi Arabia*

[g] *Chemistry Department, Faculty of Science and Art, AlBaha University, Mukhwah, Al Bahah 65731, Saudi Arabia*

[h] *Chemistry Department, Faculty of Women for Arts, Science, and Education, Ain Shams University, Heliopolis, Cairo, 11757, Egypt*

** Correspondence: elagrody_am@azhar.edu.eg (Ahmed M. El-Agrody)*

Table of Contents:	Page
**Figure S1:** ^{1}H NMR spectrum (DMSO-d_6, 500 MHz) of compound **4a**.	S5
**Figure S2:** ^{1}H NMR spectrum (DMSO-d_6, 500 MHz) of compound **4a**.	S6
**Figure S3:** ^{13}C NMR spectrum (DMSO-d_6, 125 MHz) of compound **4a**.	S7
**Figure S4:** DEPT45 spectrum (DMSO-d_6, 125 MHz) of compound **4a**.	S8
**Figure S5:** DEPT90 spectrum (DMSO-d_6, 125 MHz) of compound **4a**.	S9
**Figure S6:** DEPT135 spectrum (DMSO-d_6, 125 MHz) of compound **4a**.	S10
**Figure S7:** APT spectrum (DMSO-d_6, 125 MHz) of compound **4a**.	S11
**Figure S8:** ^{1}H NMR spectrum (DMSO-d_6, 500 MHz) of compound **4b**.	S12
**Figure S9:** ^{1}H NMR spectrum (DMSO-d_6, 500 MHz) of compound **4b**.	S13
**Figure S10:** ^{13}C NMR spectrum (DMSO-d_6, 125 MHz) of compound **4b**.	S14
**Figure S11:** DEPT45 spectrum (DMSO-d_6, 125 MHz) of compound **4b**.	S15
**Figure S12:** DEPT90 spectrum (DMSO-d_6, 125 MHz) of compound **4b**.	S16
**Figure S13:** DEPT135 spectrum (DMSO-d_6, 125 MHz) of compound **4b**.	S17
**Figure S14:** APT spectrum (DMSO-d_6, 125 MHz) of compound **4b**.	S18
**Figure S15:** ^{1}H NMR spectrum (DMSO-d_6, 500 MHz) of compound **4c**.	S19
**Figure S16:** ^{1}H NMR spectrum (DMSO-d_6, 500 MHz) of compound **4c**.	S20
**Figure S17:** ^{13}C NMR spectrum (DMSO-d_6, 125 MHz) of compound **4c**.	S21
**Figure S18:** ^{1}H NMR spectrum (DMSO-d_6, 500 MHz) of compound **4d**.	S22
**Figure S19:** ^{1}H NMR spectrum (DMSO-d_6, 500 MHz) of compound **4d**.	S23
**Figure S20:** ^{13}C NMR spectrum (DMSO-d_6, 125 MHz) of compound **4d**.	S24
**Figure S21:** ^{1}H NMR spectrum (DMSO-d_6, 500 MHz) of compound **4e**.	S25
**Figure S22:** ^{13}C NMR spectrum (DMSO-d_6, 500 MHz) of compound **4e**.	S26
**Figure S23:** ^{1}H NMR spectrum (DMSO-d_6, 125 MHz) of compound **4f**.	S27
**Figure S24:** ^{13}C NMR spectrum (DMSO-d_6, 500 MHz) of compound **4f**.	S28
**Figure S25:** ^{1}H NMR spectrum (DMSO-d_6, 500 MHz) of compound **4g**.	S29
**Figure S26:** ^{13}C NMR spectrum (DMSO-d_6, 125 MHz) of compound **4g**.	S30
**Figure S27:** ^{1}H NMR spectrum (DMSO-d_6, 500 MHz) of compound **4h**.	S31
**Figure S28:** ^{1}H NMR spectrum (DMSO-d_6, 500 MHz) of compound **4h**	S32
**Figure S29:** ^{13}C NMR spectrum (DMSO-d_6, 125 MHz) of compound **4h**.	S33
**Figure S30:** DEPT45 spectrum (DMSO-d_6, 125 MHz) of compound **4h**.	S34
**Figure S31:** DEPT90 spectrum (DMSO-d_6, 125 MHz) of compound **4h**.	S35
**Figure S32:** DEPT135 spectrum (DMSO-d_6, 125 MHz) of compound **4h**.	S36
**Figure S33:** APT spectrum (DMSO-d_6, 125 MHz) of compound **4h**.	S37
**Figure S34:** ^{1}H NMR spectrum (DMSO-d_6, 500 MHz) of compound **4i**	S38
**Figure S35:** ^{1}H NMR spectrum (DMSO-d_6, 500 MHz) of compound **4i**	S39
**Figure S36:**.^{13}C NMR spectrum (DMSO-d_6, 500 MHz) of compound **4i**	S40
**Figure S37:**. DEPT45 spectrum (DMSO-d_6, 125 MHz) of compound **4i**	S41

Figure S38:. DEPT90 spectrum (DMSO-d_6, 125 MHz) of compound **4i** S42
Figure S39: DEPT135 spectrum (DMSO-d_6, 125 MHz) of compound **4i**. S43
Figure S40:. APT spectrum (DMSO-d_6, 125 MHz) of compound **4i** S44
Figure S41:.^{1}H NMR spectrum (DMSO-d_6, 500 MHz) of compound **4j** S45
Figure S42:.^{13}C NMR spectrum (DMSO-d_6, 125 MHz) of compound **4j** S46
Figure S43: ^{1}H NMR spectrum (DMSO-d_6, 500 MHz) of compound **4k** S47
Figure S44:.^{1}H NMR spectrum (DMSO-d_6, 500 MHz) of compound **4k** S48
Figure S45: ^{13}C NMR spectrum (DMSO-d_6, 500 MHz) of compound **4k** S49
Figure S46: DEPT45 spectrum (DMSO-d_6, 125 MHz) of compound **4k** S50
Figure S47: DEPT90 spectrum (DMSO-d_6, 125 MHz) of compound 4**k** S51
Figure S48: DEPT135 spectrum (DMSO-d_6, 125 MHz) of compound **4k** S52
Figure S49: APT spectrum (DMSO-d_6, 125 MHz) of compound **4k** S53
Figure S50: ^{1}H NMR spectrum (DMSO-d_6, 500 MHz) of compound **4l**. S54
Figure S51: ^{1}H NMR spectrum (DMSO-d_6, 500 MHz) of compound **4l**. S55
Figure S52: ^{13}C NMR spectrum (DMSO-d_6, 125 MHz) of compound **4l** S56
Figure S53: DEPT45 spectrum (DMSO-d_6, 125 MHz) of compound **4l**. S57
Figure S54: DEPT90 spectrum (DMSO-d_6, 125 MHz) of compound **4l** S58
Figure S55: DEPT135 spectrum (DMSO-d_6, 125 MHz) of compound **4l**. S59
Figure S56: APT spectrum (DMSO-d_6, 125 MHz) of compound **4l** S60
Figure S57: ^{1}H NMR spectrum (DMSO-d_6, 500 MHz) of compound **4m**. S61
Figure S58: ^{13}C NMR spectrum (DMSO-d_6, 125 MHz) of compound **4m**. S62
Figure S59: ^{1}H NMR spectrum (DMSO-d_6, 125 MHz) of compound **4n**. S63
Figure S60: ^{1}H NMR spectrum (DMSO-d_6, 500 MHz) of compound **4n**. S64
Figure S61: ^{13}C NMR spectrum (DMSO-d_6, 500 MHz) of compound **4n** S65
Figure S62:.^{1}H NMR spectrum (DMSO-d_6, 500 MHz) of compound **4o** S66
Figure S63: ^{1}H NMR spectrum (DMSO-d_6, 500 MHz) of compound **4o**. S67
Figure S64: ^{13}C NMR spectrum (DMSO-d_6, 500 MHz) of compound **4o**. S68
Figure S65: ^{1}H NMR spectrum (DMSO-d_6, 125 MHz) of compound **4p**. S69
Figure S66: ^{1}H NMR spectrum (DMSO-d_6, 125 MHz) of compound **4p** S70
Figure S67: ^{13}C NMR spectrum (DMSO-d_6, 125 MHz) of compound **4p**. S71
Figure S68: ^{1}H NMR spectrum (DMSO-d_6, 125 MHz) of compound **4q**. S72
Figure S69: ^{1}H NMR spectrum (DMSO-d_6, 500 MHz) of compound **4q**. S73
Figure S70: ^{13}C NMR spectrum (DMSO-d_6, 500 MHz) of compound **4q**. S74
Figure S71: ^{1}H NMR spectrum (DMSO-d_6, 125 MHz) of compound **4r**. S75
Figure S72: ^{13}C NMR spectrum (DMSO-d_6, 125 MHz) of compound **4r** S76
Figure S73: ^{1}H NMR spectrum (DMSO-d_6, 125 MHz) of compound **4s** S77
Figure S74: ^{13}C NMR spectrum (DMSO-d_6, 125 MHz) of compound **4s** S78
Figure S75: APT spectrum (DMSO-d_6, 125 MHz) of compound **4s** S79
Figure S76: ^{1}H NMR spectrum (DMSO-d_6, 500 MHz) of compound **4t**. S80

Figure S77: ^{13}C NMR spectrum (DMSO-d_6, 125 MHz) of compound **4t**. S81
Figure S78: ^{1}H NMR spectrum (DMSO-d_6, 125 MHz) of compound **4u**. S82
Figure S79: ^{13}C NMR spectrum (DMSO-d_6, 125 MHz) of compound **4u** S83
Figure S80: ^{1}H NMR spectrum (DMSO-d_6, 125 MHz) of compound **4v** S84
Figure S81: ^{13}C NMR spectrum (DMSO-d_6, 125 MHz) of compound **4v** S85
Figure S82: ^{1}H NMR spectrum (DMSO-d_6, 125 MHz) of compound **4w** S86
Figure S83: ^{13}C NMR spectrum (DMSO-d_6, 125 MHz) of compound **4w** S87
Figure S84: ^{1}H NMR spectrum (DMSO-d_6, 125 MHz) of compound **4x** S88
Figure S85: ^{1}H NMR spectrum (DMSO-d_6, 125 MHz) of compound **4x** S89
Figure S86: ^{13}C NMR spectrum (DMSO-d_6, 125 MHz) of compound **4x** S90
Figure S87: DEPT45 spectrum (DMSO-d_6, 125 MHz) of compound 4**x** S91
Figure S88: DEPT90 spectrum (DMSO-d_6, 125 MHz) of compound 4**x** S92
Figure S89: DEPT135 spectrum (DMSO-d_6, 125 MHz) of compound 4**x** S93
Figure S90: APT spectrum (DMSO-d_6, 125 MHz) of compound 4**x** S94
Figure S91: ^{1}H NMR spectrum (DMSO-d_6, 125 MHz) of compound **4y** S95
Figure S92: ^{1}H NMR spectrum (DMSO-d_6, 125 MHz) of compound **4y** S96
Figure S93: ^{13}C NMR spectrum (DMSO-d_6, 125 MHz) of compound **4y** S97
Figure S94: DEPT45 spectrum (DMSO-d_6, 125 MHz) of compound 4**y** S98
Figure S95: DEPT90 spectrum (DMSO-d_6, 125 MHz) of compound 4**y** S99
Figure S96: DEPT135 spectrum (DMSO-d_6, 125 MHz) of compound 4**y** S100
Figure S97: APT spectrum (DMSO-d_6, 125 MHz) of compound 4**y** S101
Figure S98: ^{1}H NMR spectrum (DMSO-d_6, 125 MHz) of compound **4z** S102
Figure S99: ^{1}H NMR spectrum (DMSO-d_6, 125 MHz) of compound **4z** S103
Figure S100: ^{1}H NMR spectrum (DMSO-d_6, 125 MHz) of compound **4z** S104

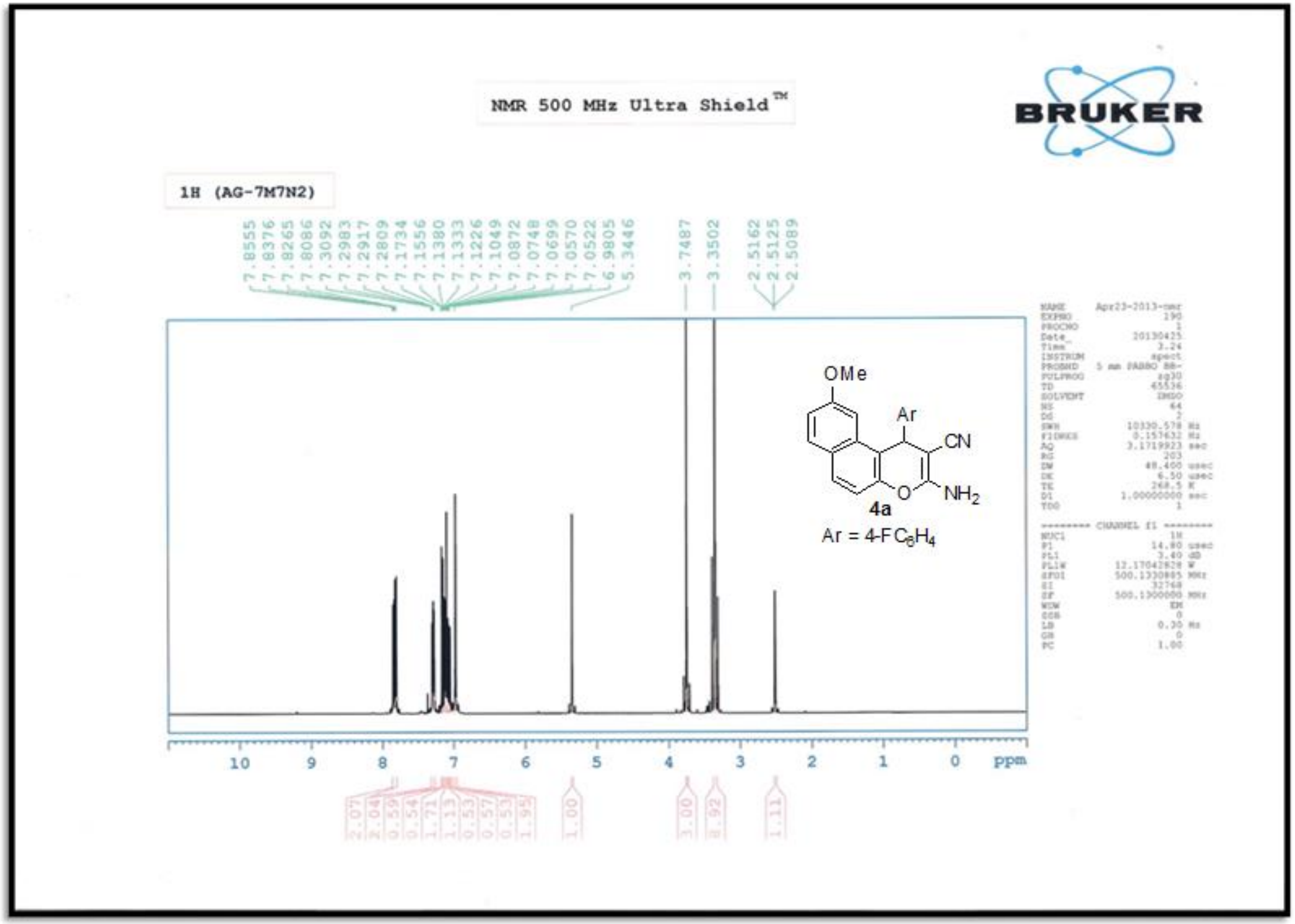

Figure S1: ^{1}H NMR spectrum (DMSO-d_6, 500 MHz) of compound **4a**.

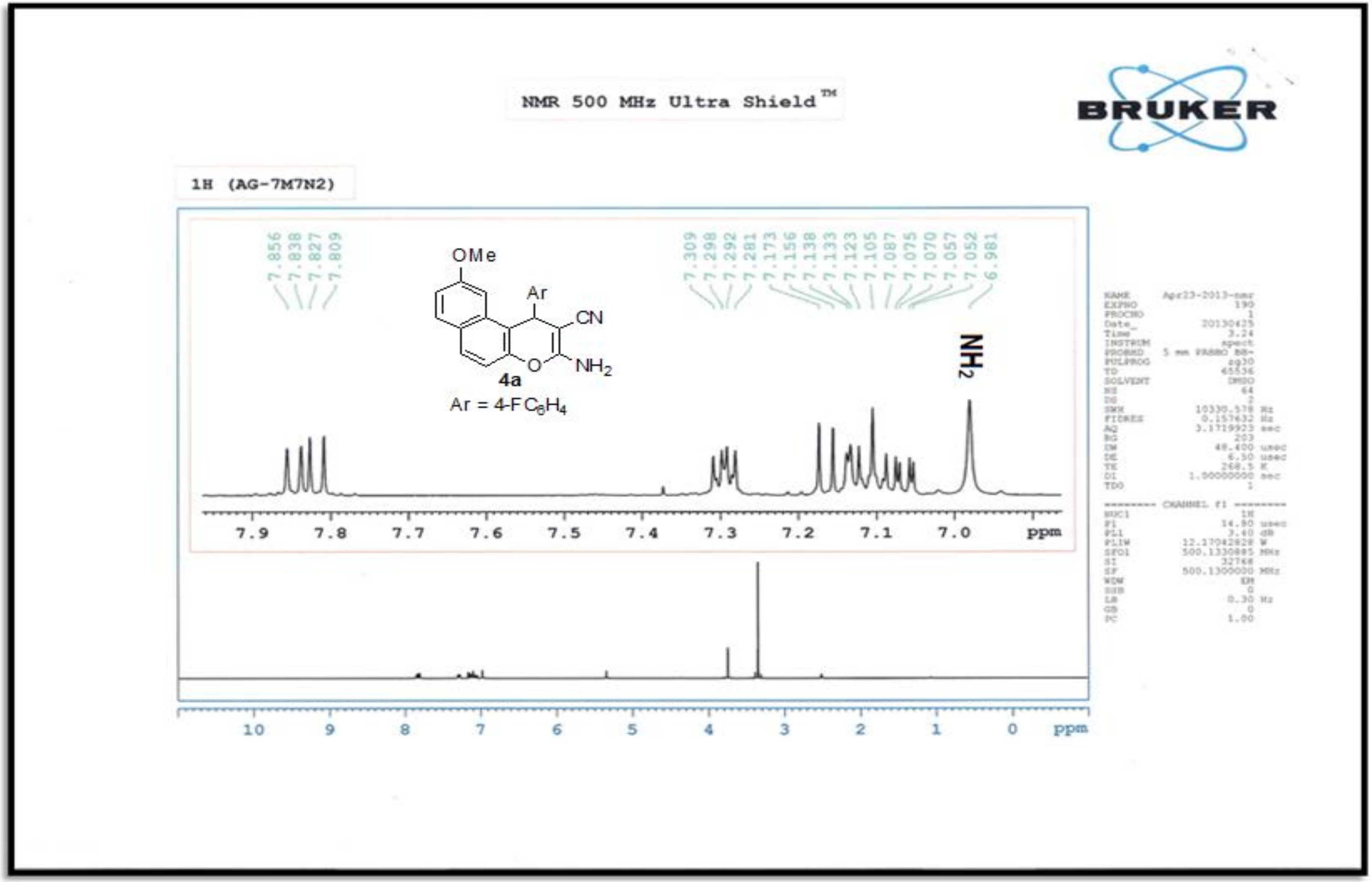

Figure S2: ^{1}H NMR spectrum (DMSO-d_6, 500 MHz) of compound **4a**.

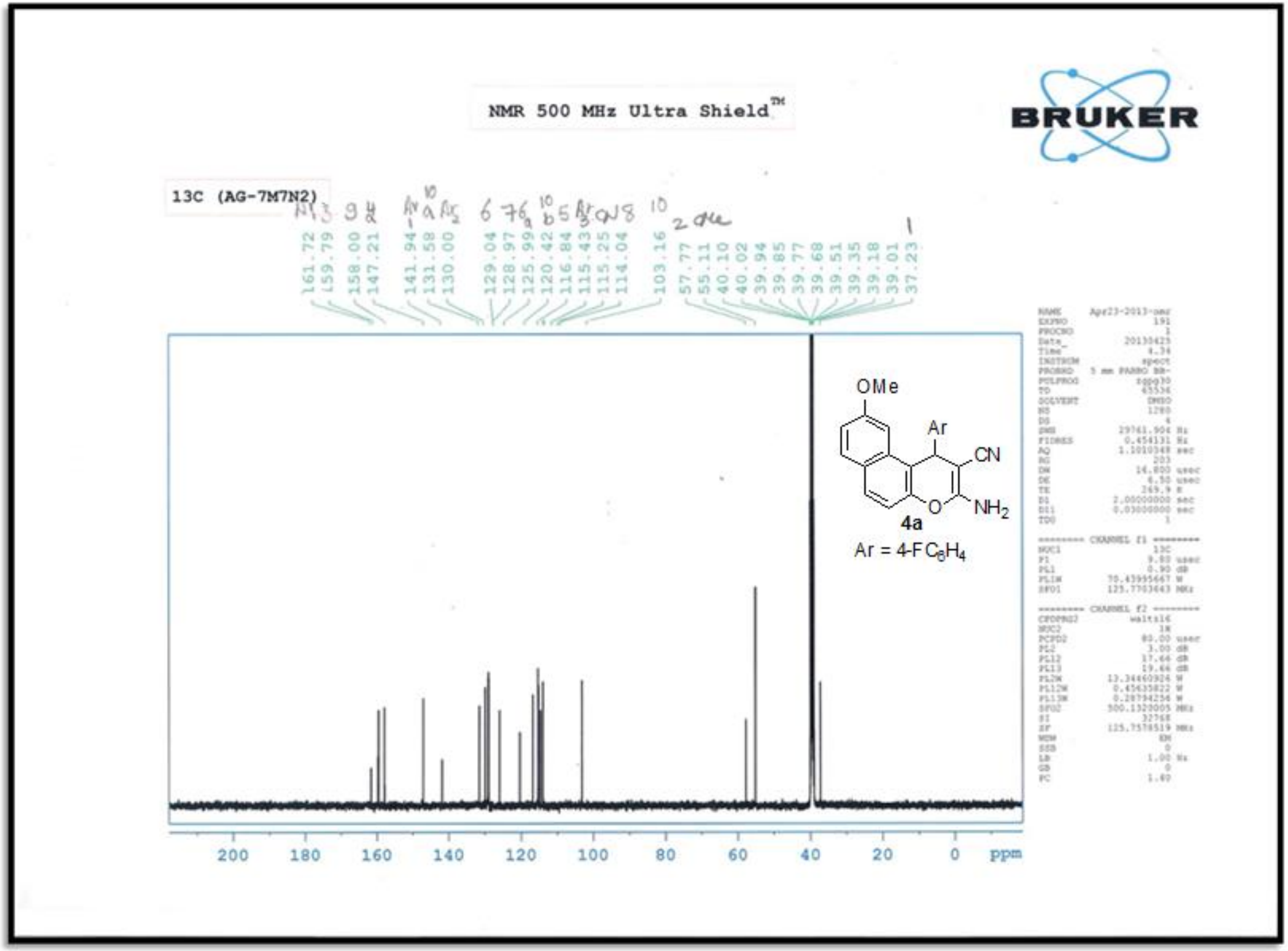

Figure S3: ^{13}C NMR spectrum (DMSO-d_6, 125 MHz) of compound **4a**.

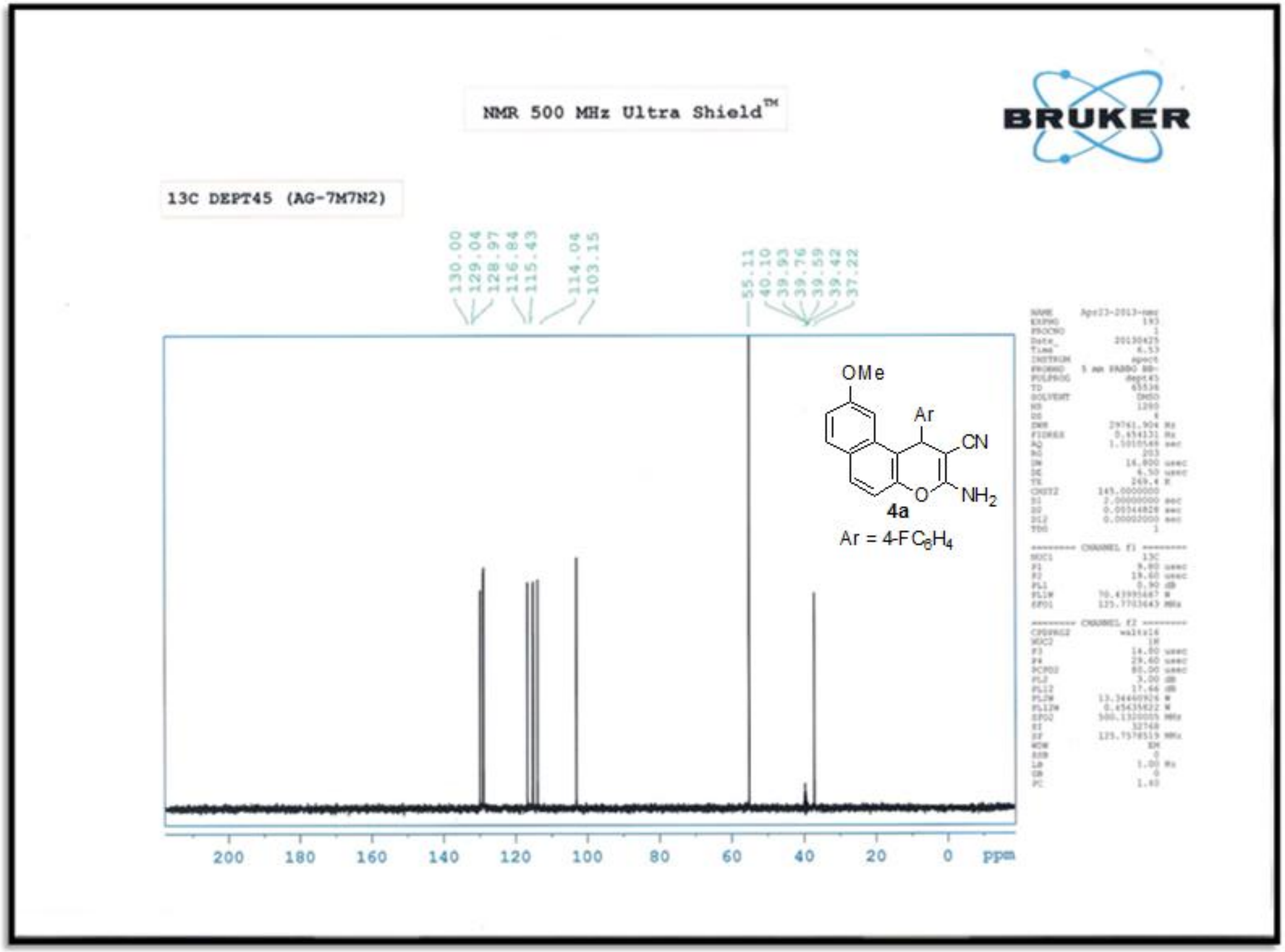

Figure S4: DEPT45 spectrum (DMSO-d_6, 125 MHz) of compound **4a.**

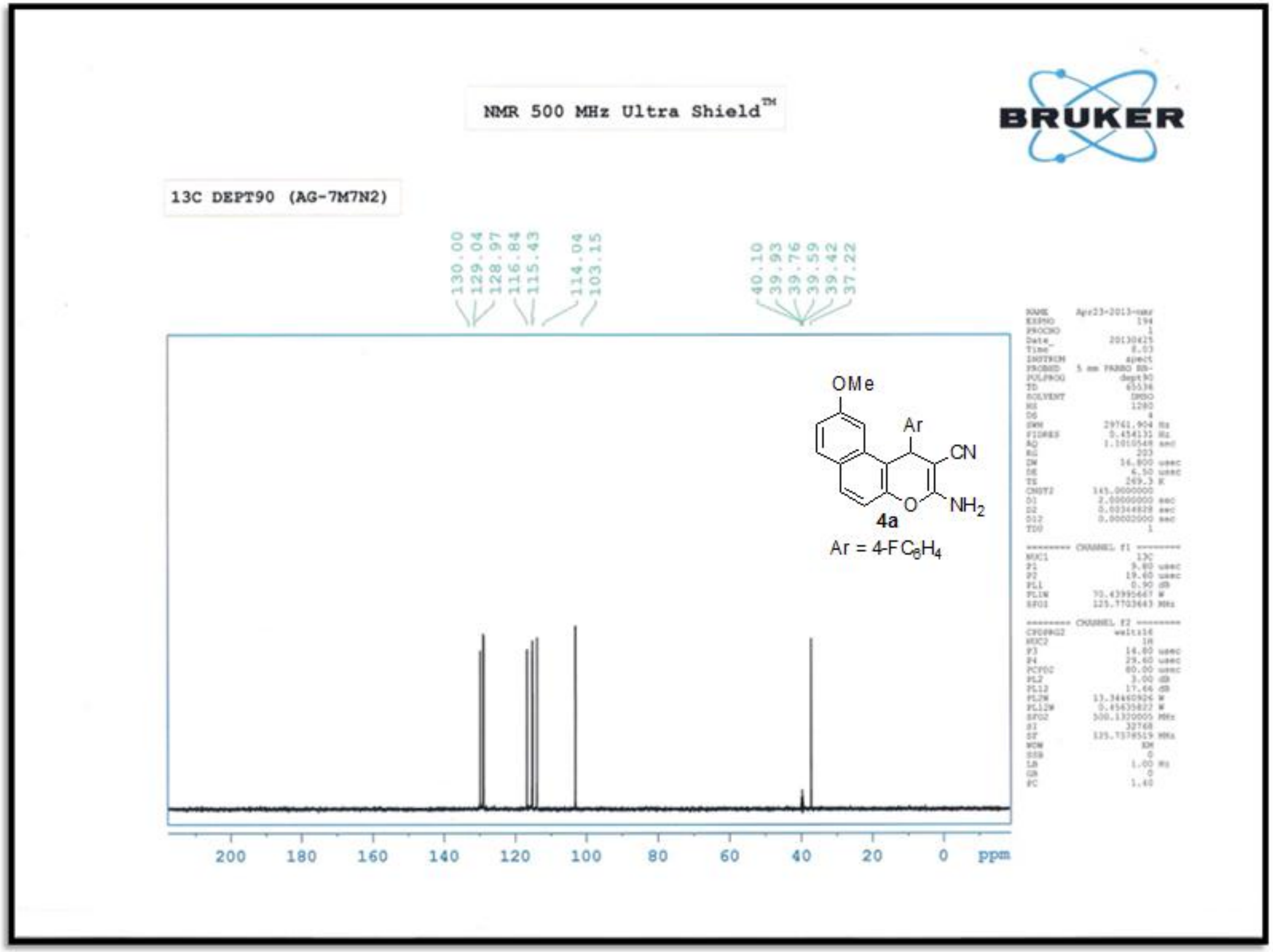

Figure S5: DEPT90 spectrum (DMSO-d_6, 125 MHz) of compound **4a.**

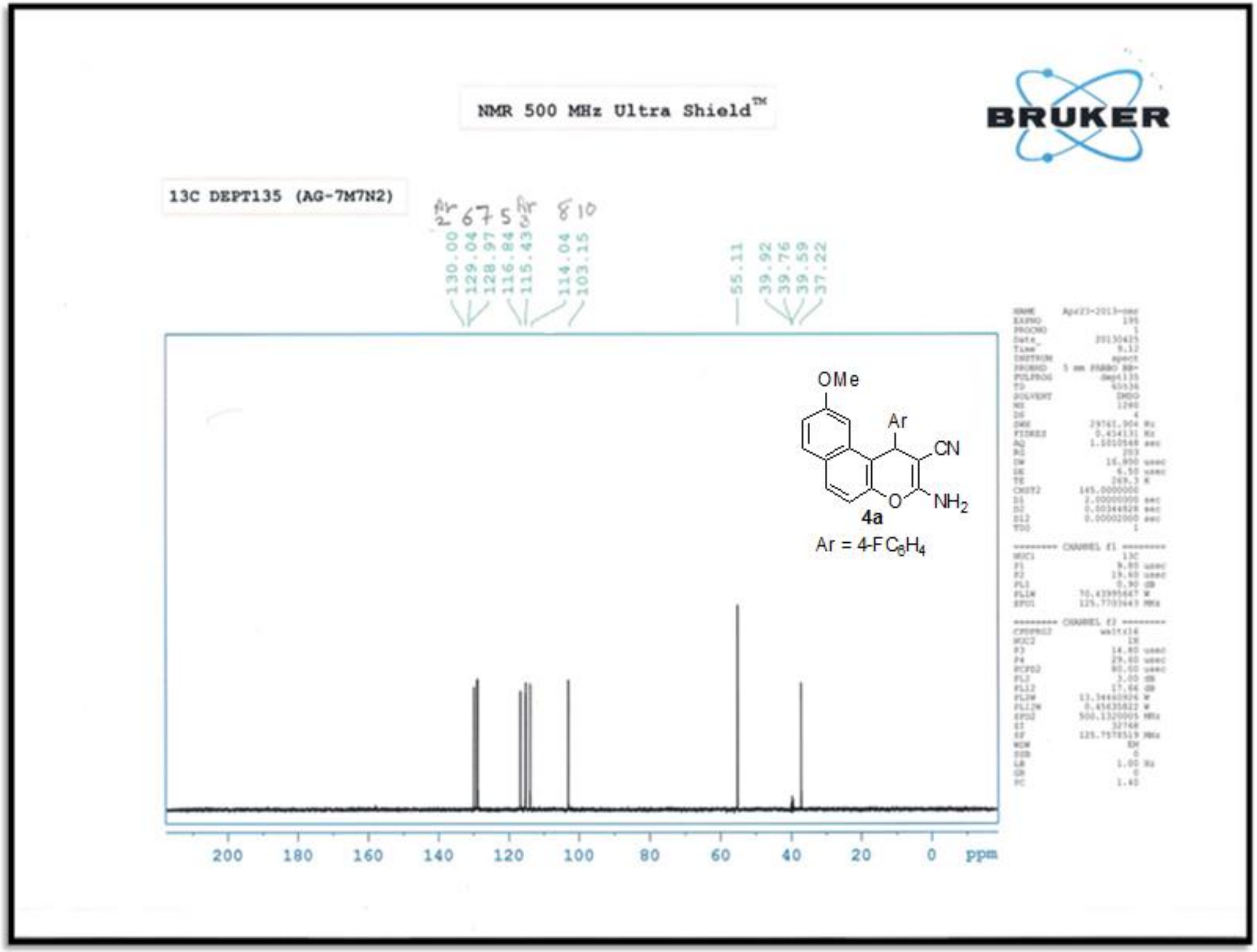

Figure S6: DEPT135 spectrum (DMSO-d_6, 125 MHz) of compound **4a.**

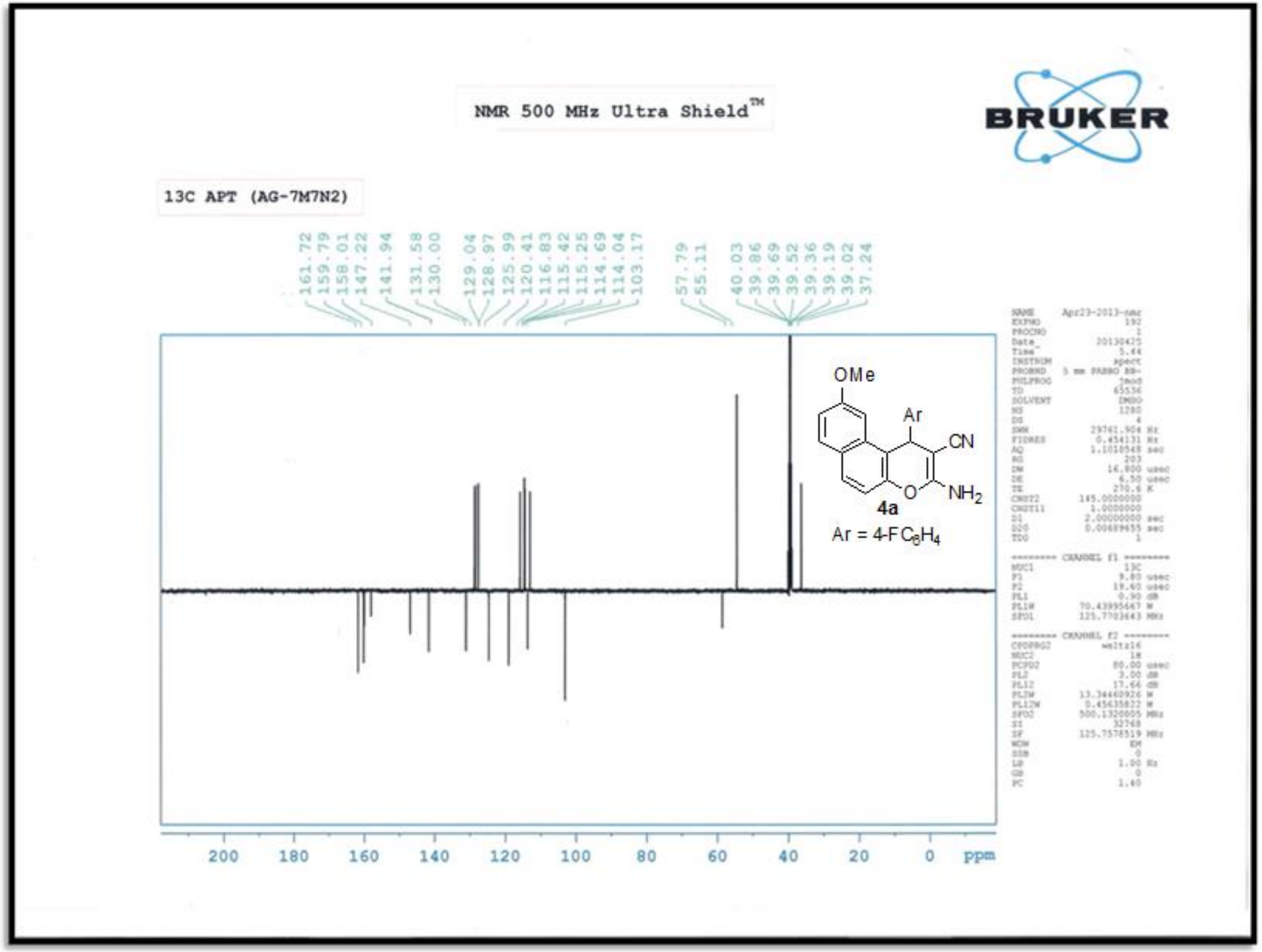

Figure S7: APT spectrum (DMSO-d_6, 125 MHz) of compound **4a**.

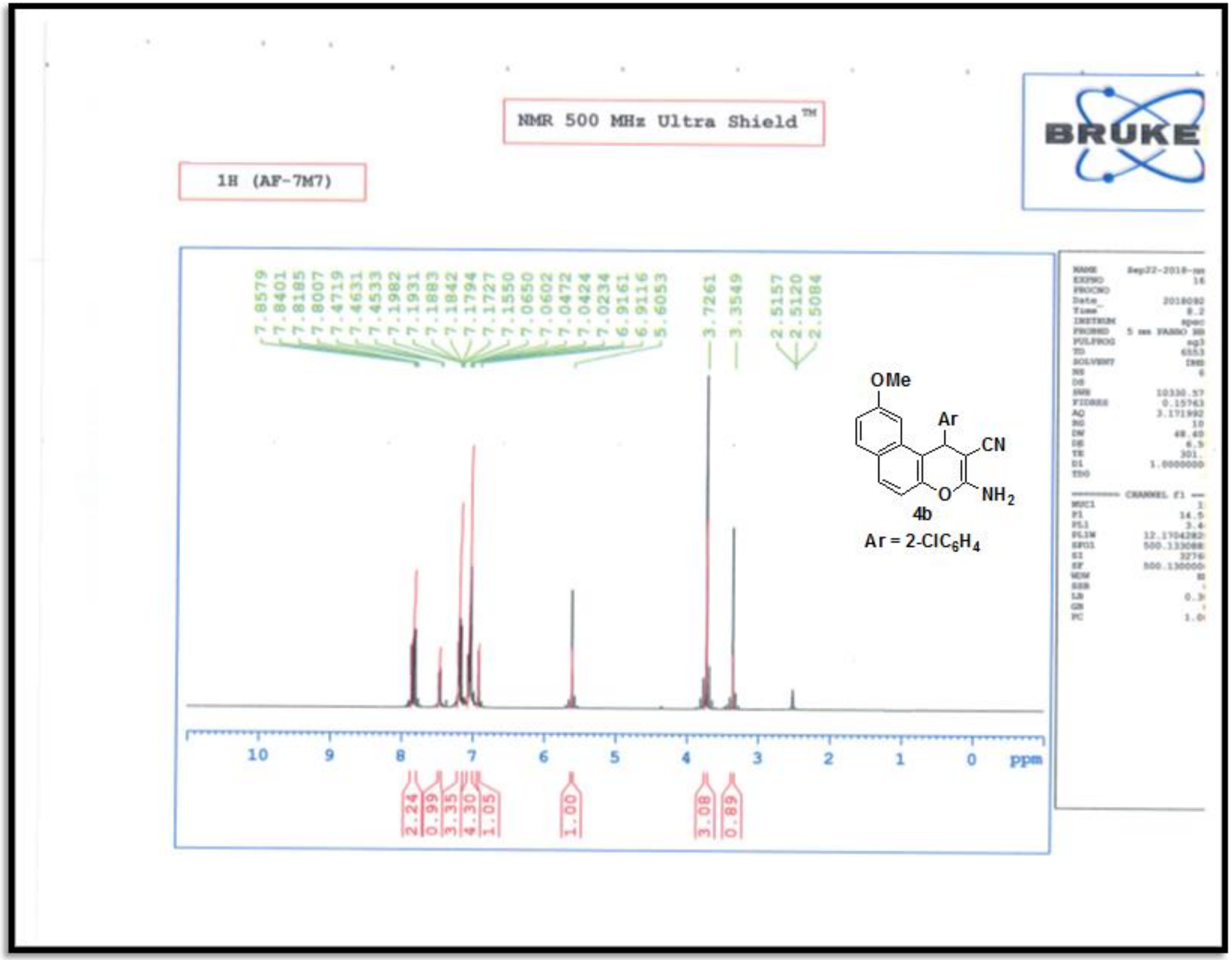

Figure S8: ^{1}H NMR spectrum (DMSO-d_6, 500 MHz) of compound **4b**.

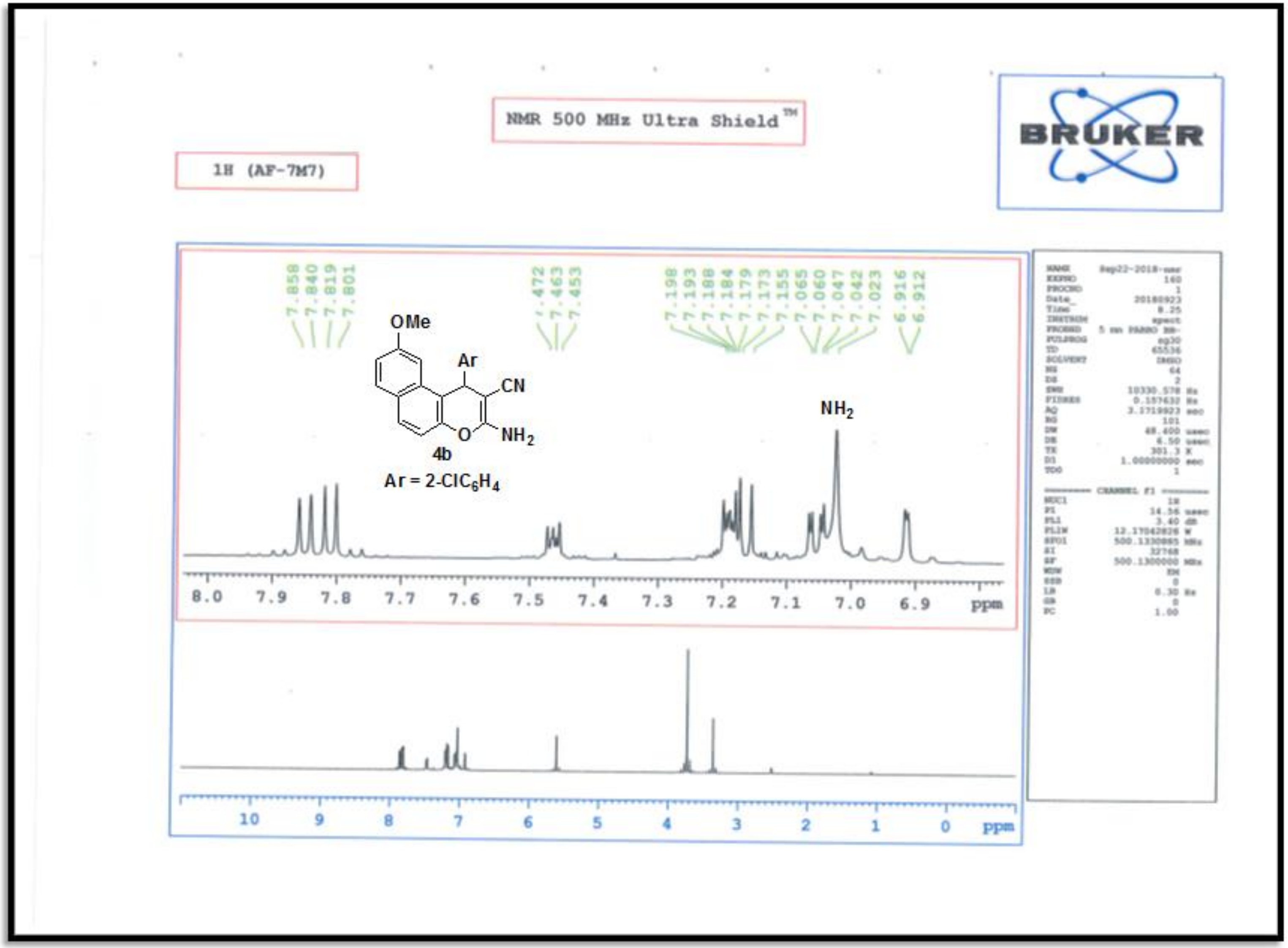

Figure S9: ^{1}H NMR spectrum (DMSO-d_6, 500 MHz) of compound **4b**.

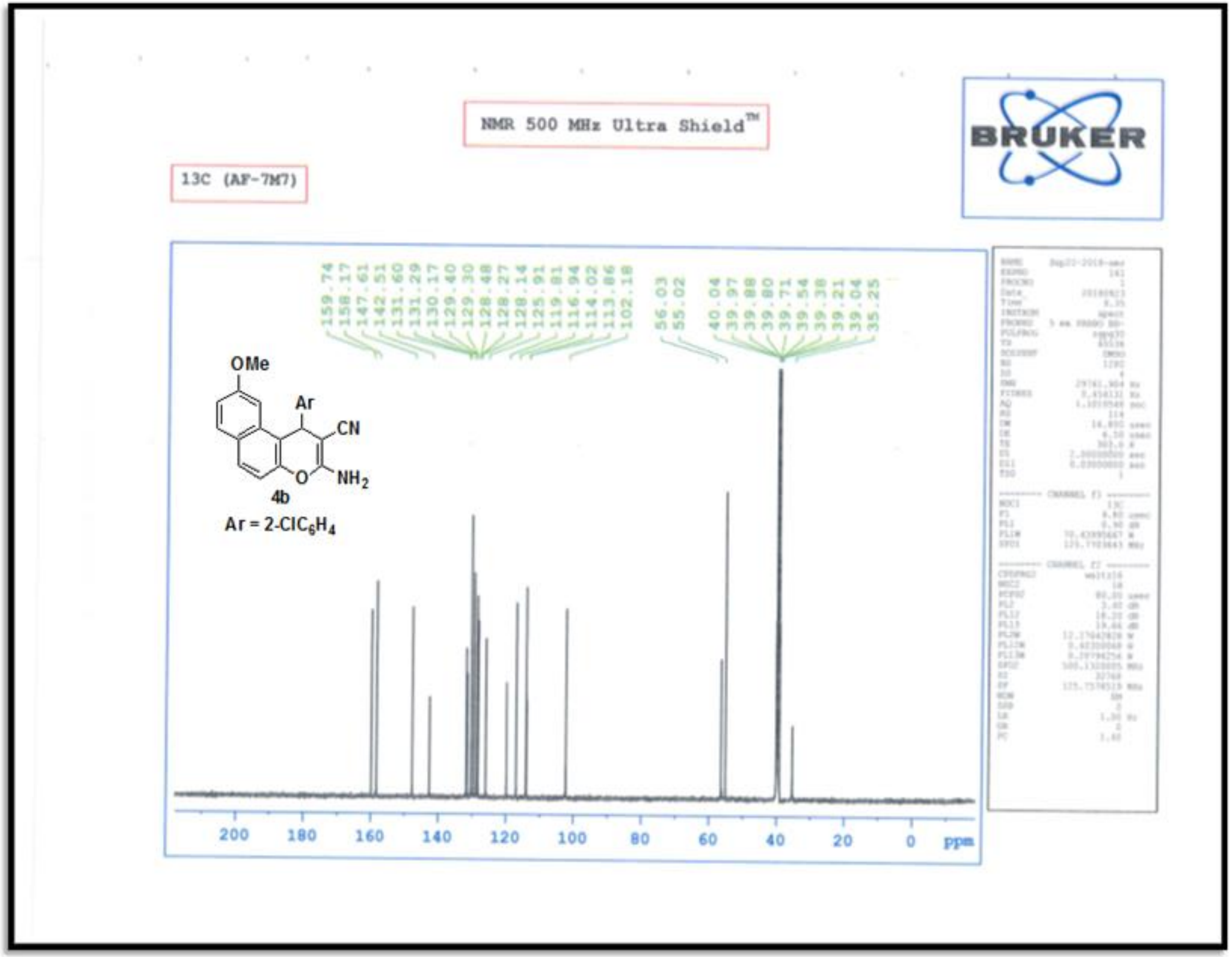

Figure S10: ^{13}C NMR spectrum (DMSO-d_6, 125 MHz) of compound **4b**.

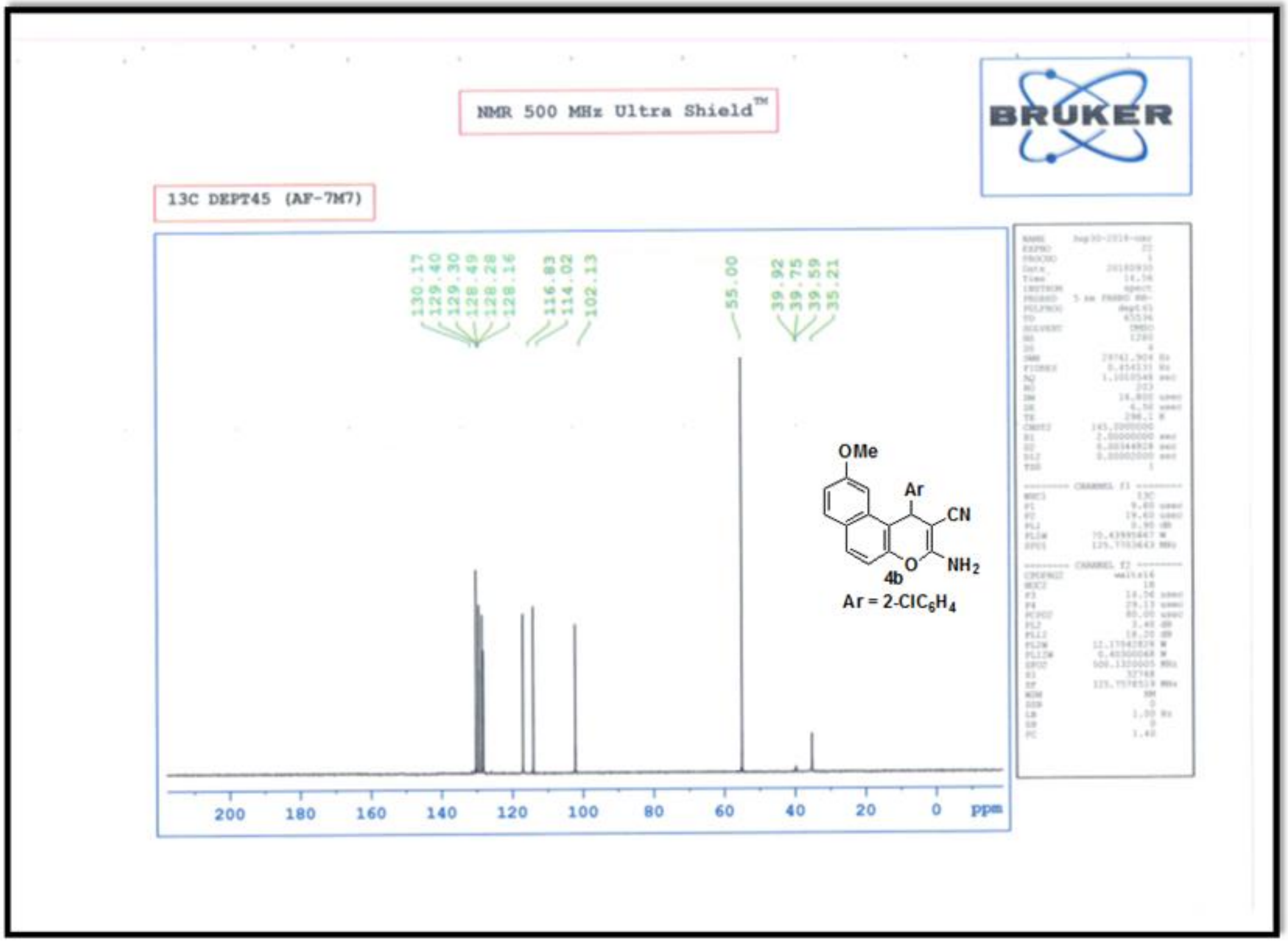

Figure S11: DEPT45 spectrum (DMSO-d_6, 125 MHz) of compound **4b.**

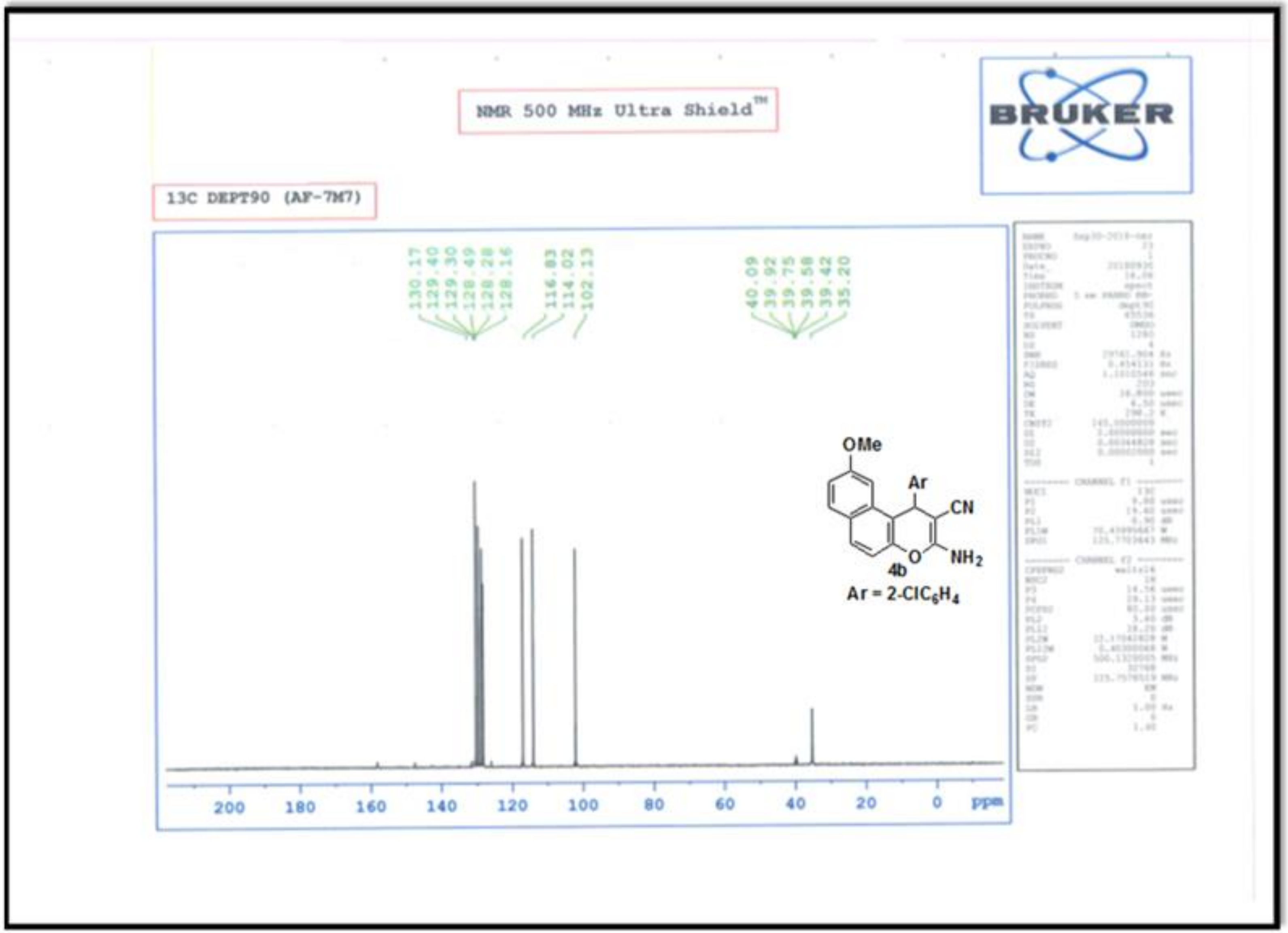

Figure S12: DEPT90 spectrum (DMSO-d_6, 125 MHz) of compound **4b.**

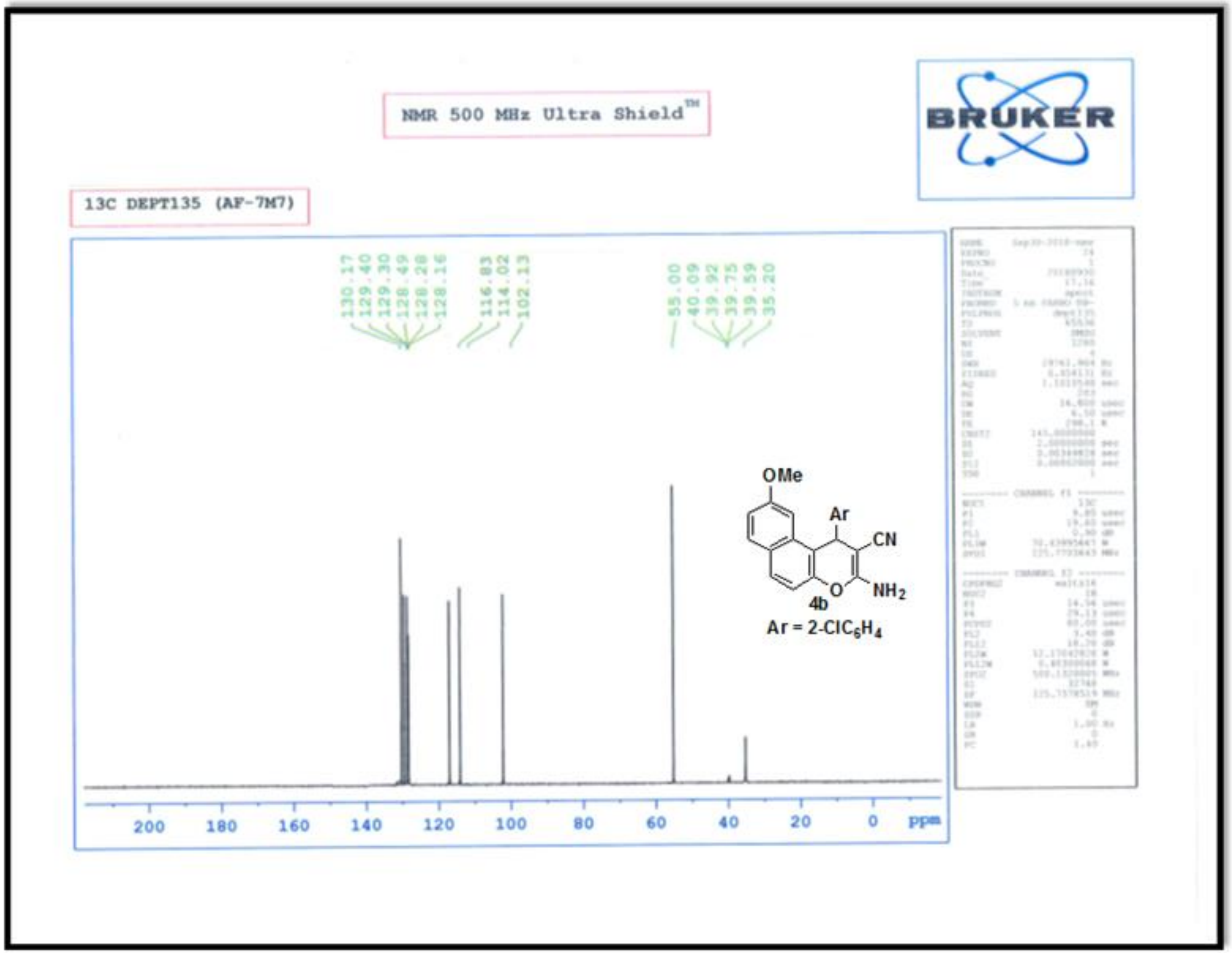

Figure S13: DEPT135 spectrum (DMSO-d_6, 125 MHz) of compound **4b.**

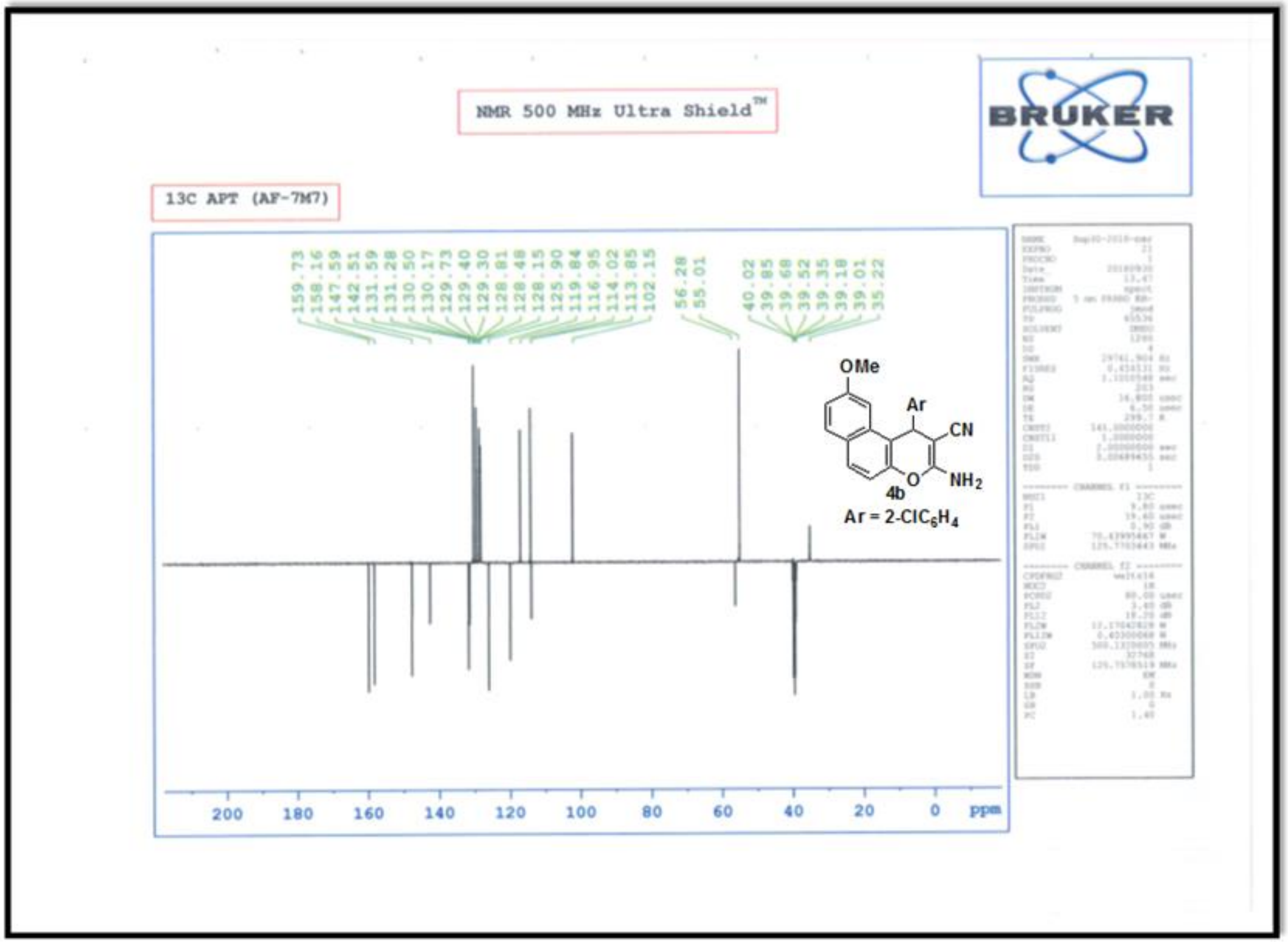

Figure S14: APT spectrum (DMSO-d_6, 125 MHz) of compound **4b**.

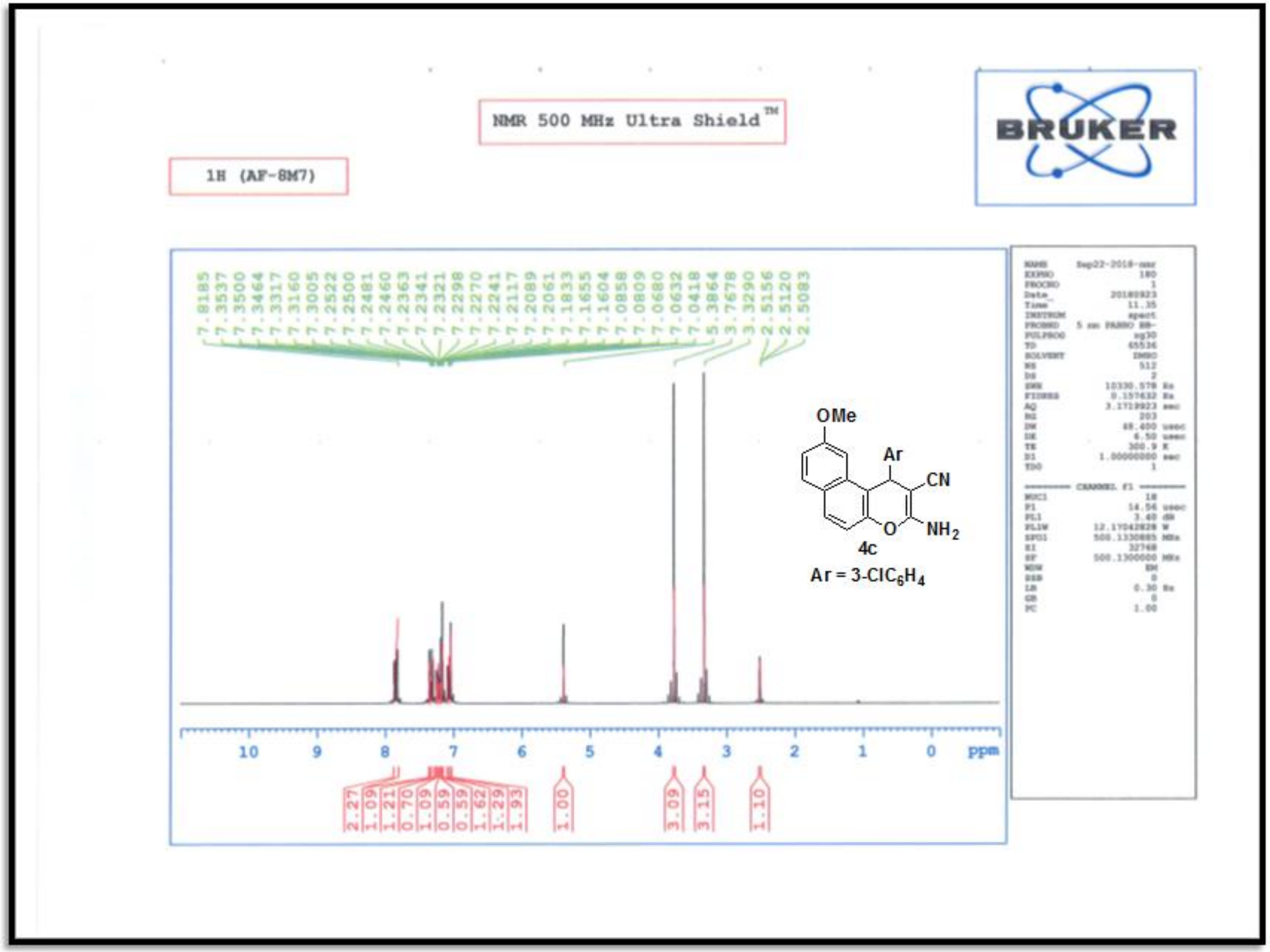

Figure S15: ^{1}H NMR spectrum (DMSO-d_6, 500 MHz) of compound **4c**.

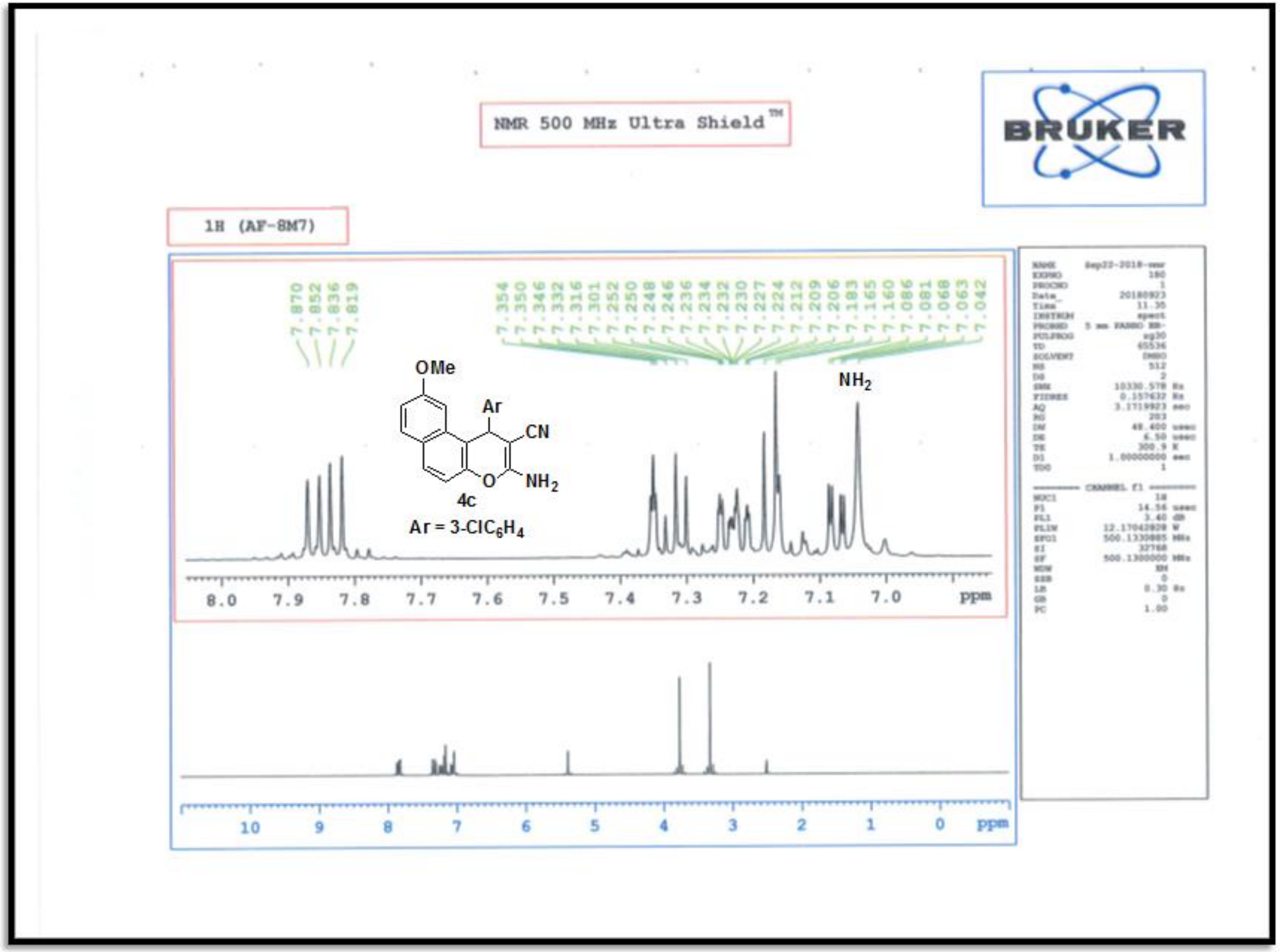

Figure S16: ^{1}H NMR spectrum (DMSO-d_6, 500 MHz) of compound **4c**.

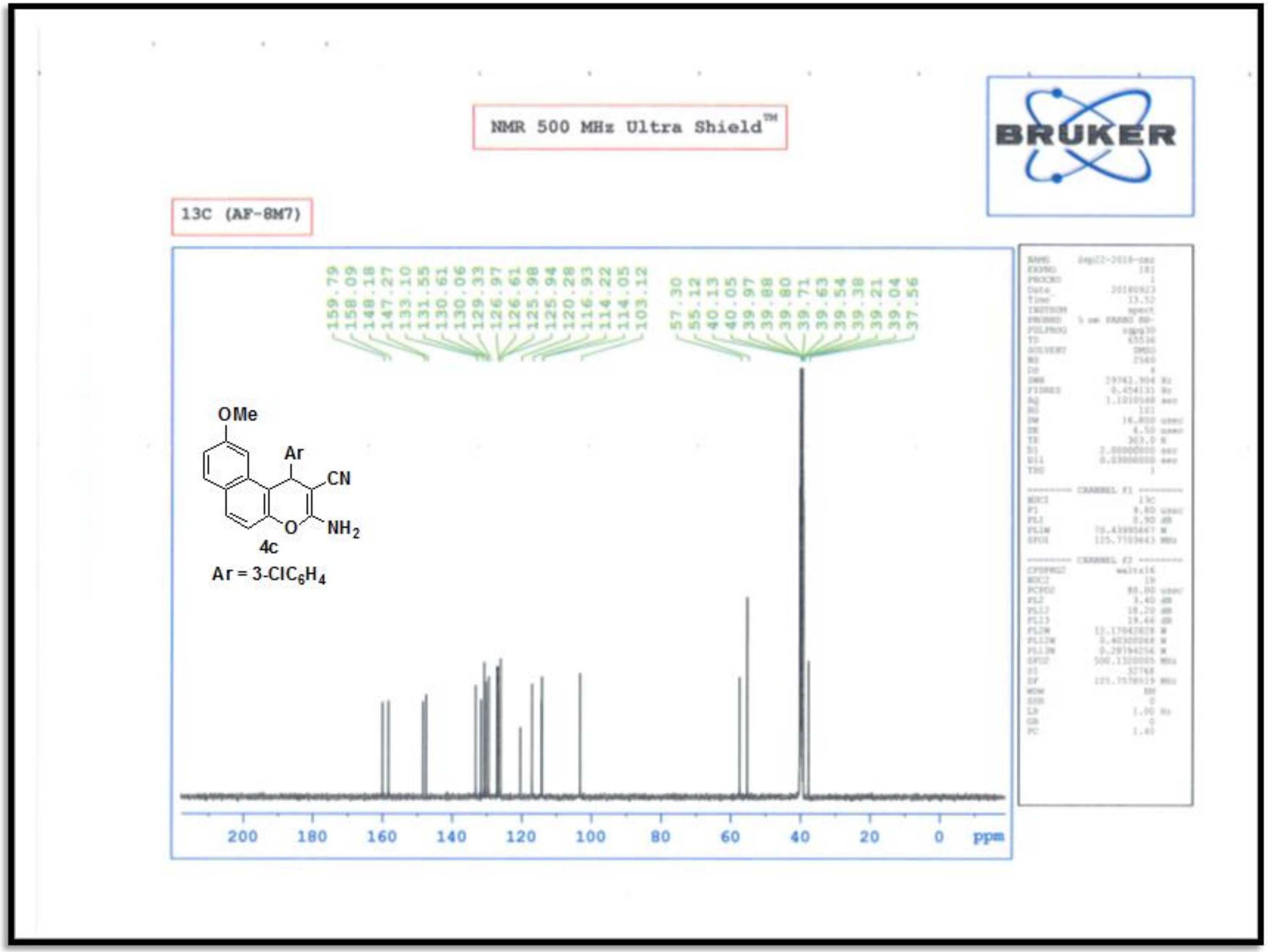

Figure S17: ^{13}C NMR spectrum (DMSO-d_6, 125 MHz) of compound **4c**.

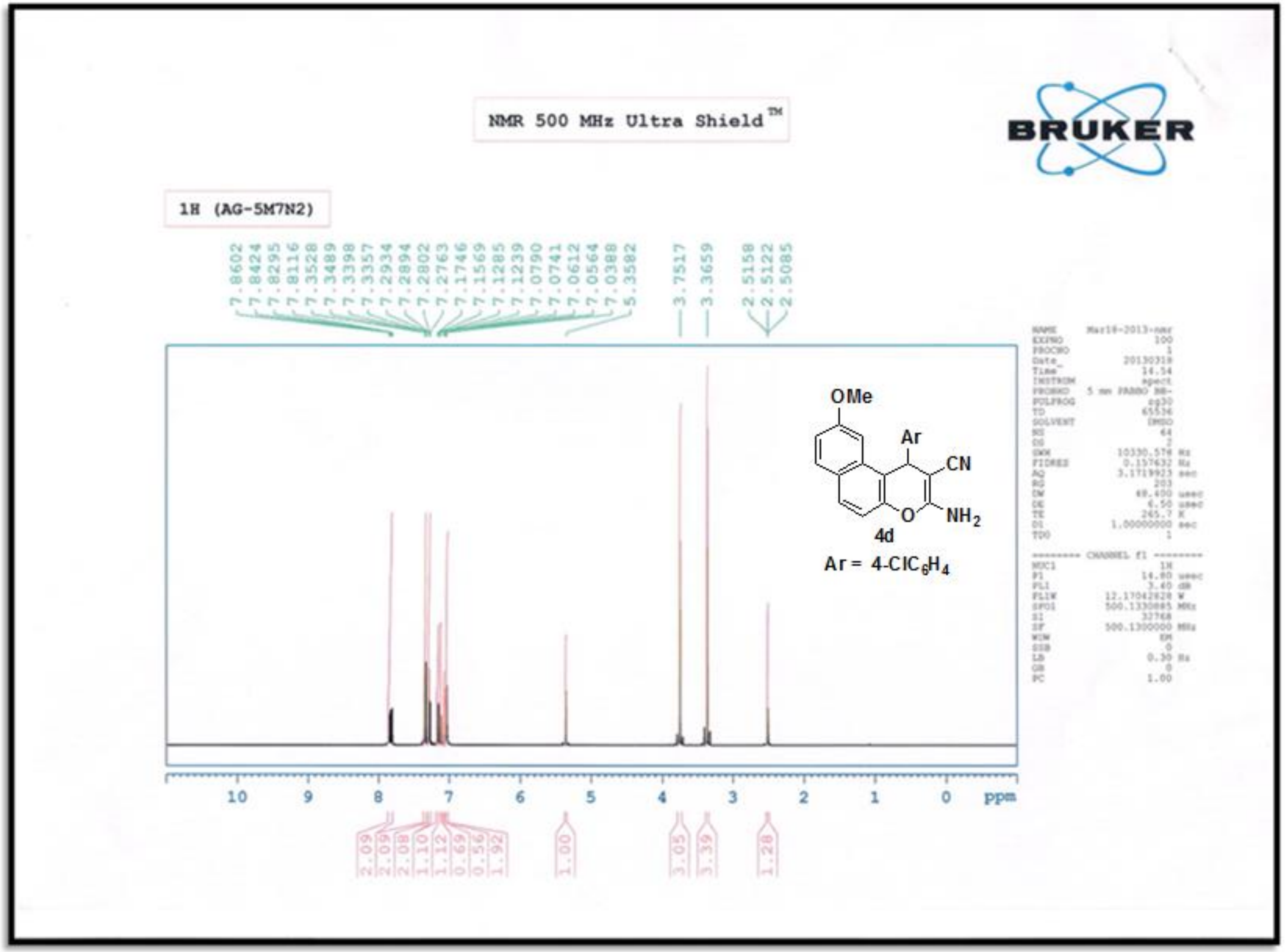

Figure S18: ^{1}H NMR spectrum (DMSO-d_6, 500 MHz) of compound **4d**.

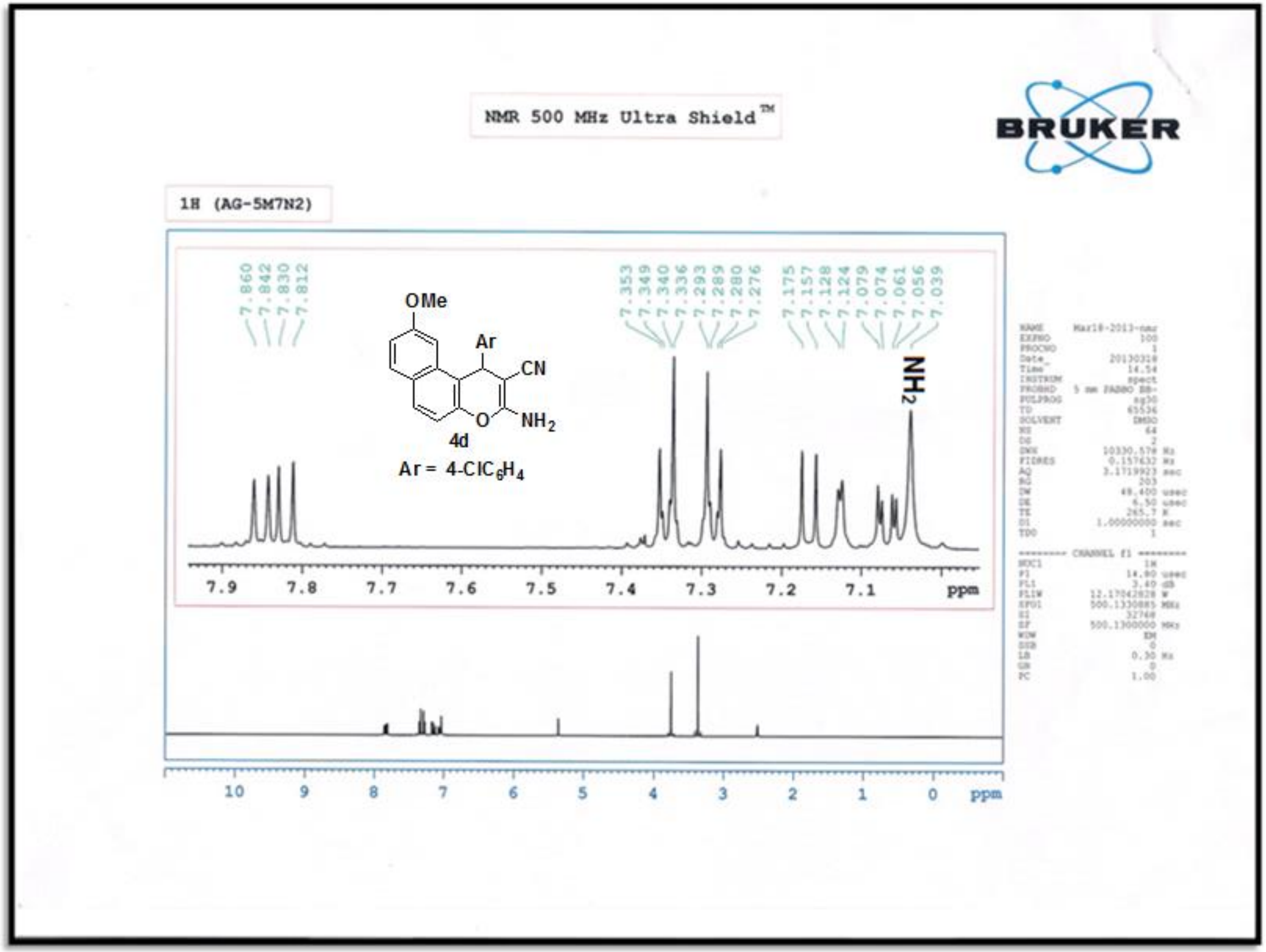

Figure S19: ^{1}H NMR spectrum (DMSO-d_6, 500 MHz) of compound **4d**.

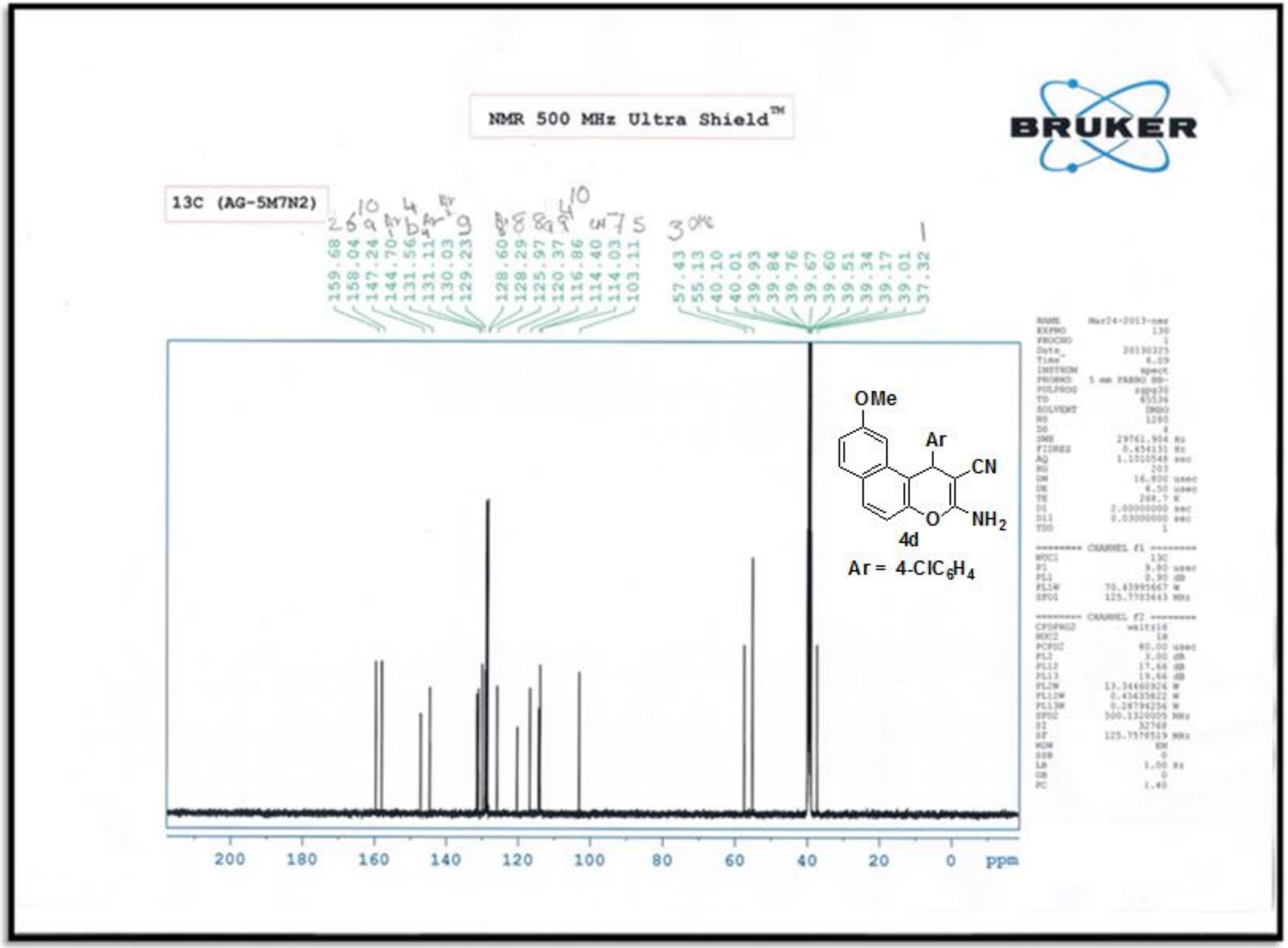

Figure S20: ^{13}C NMR spectrum (DMSO-d_6, 125 MHz) of compound **4d**.

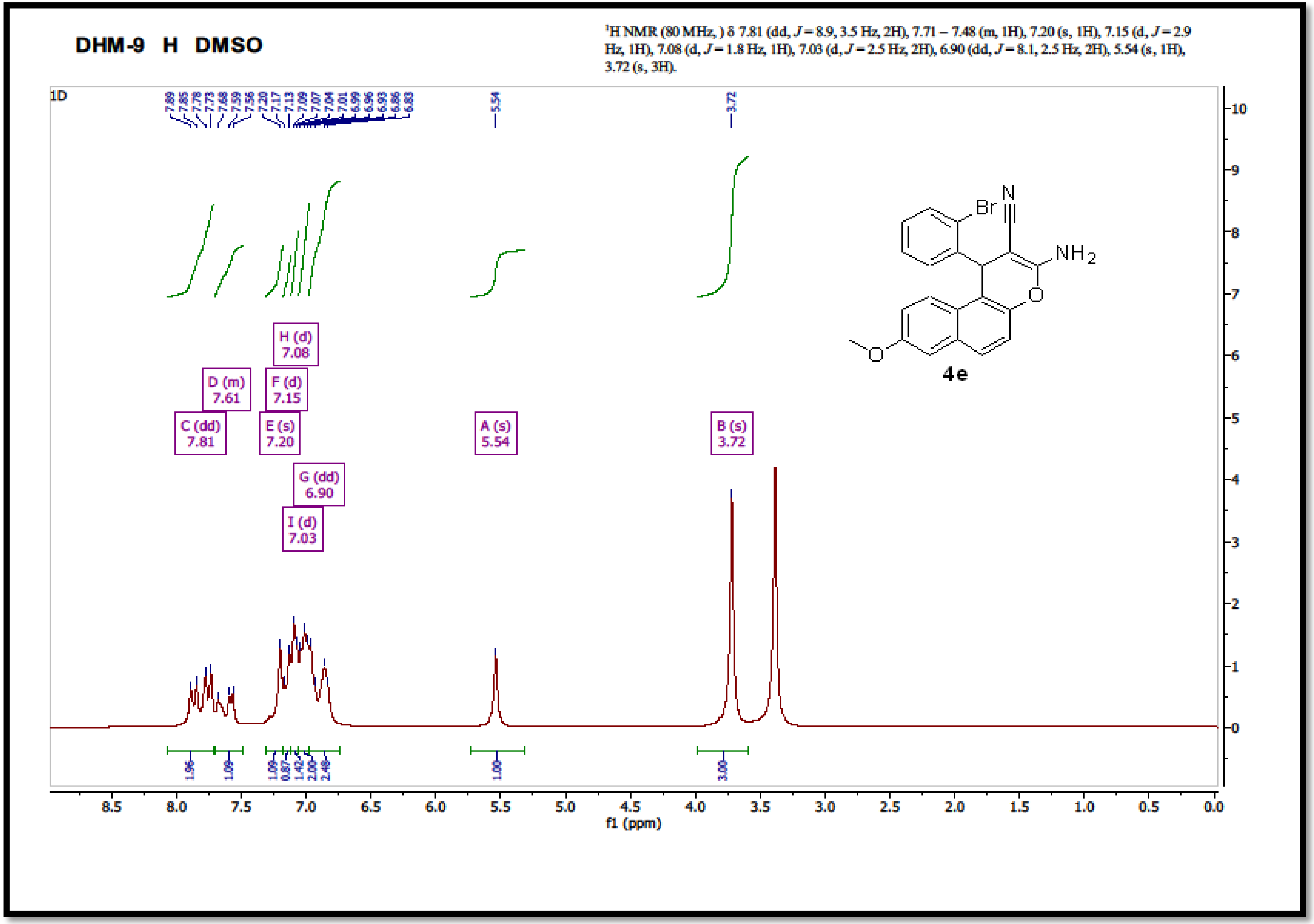

Figure S21: ^{1}H NMR spectrum (DMSO-d_6, 500 MHz) of compound **4e**.

DHM-9 C DMSO

^{13}C NMR (20 MHz,) δ 160.14, 158.69, 148.03, 144.74, 135.99, 134.10, 132.99, 132.10, 130.65, 129.94, 129.26, 126.42, 122.54, 120.19, 117.49, 114.56, 102.79, 56.93, 55.76., 35.76.

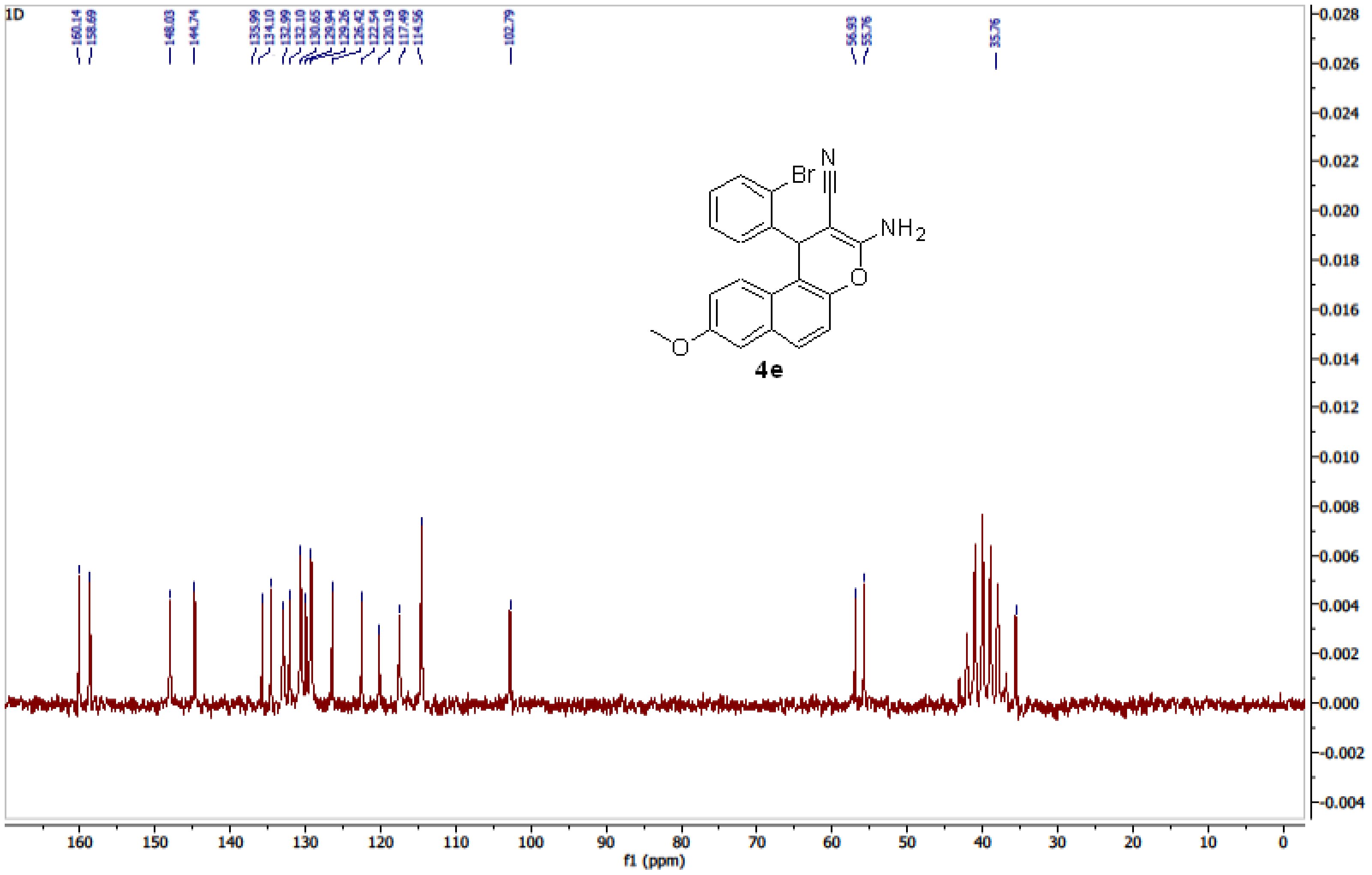

Figure S22: ^{13}C NMR spectrum (DMSO-d_6, 500 MHz) of compound **4e**.

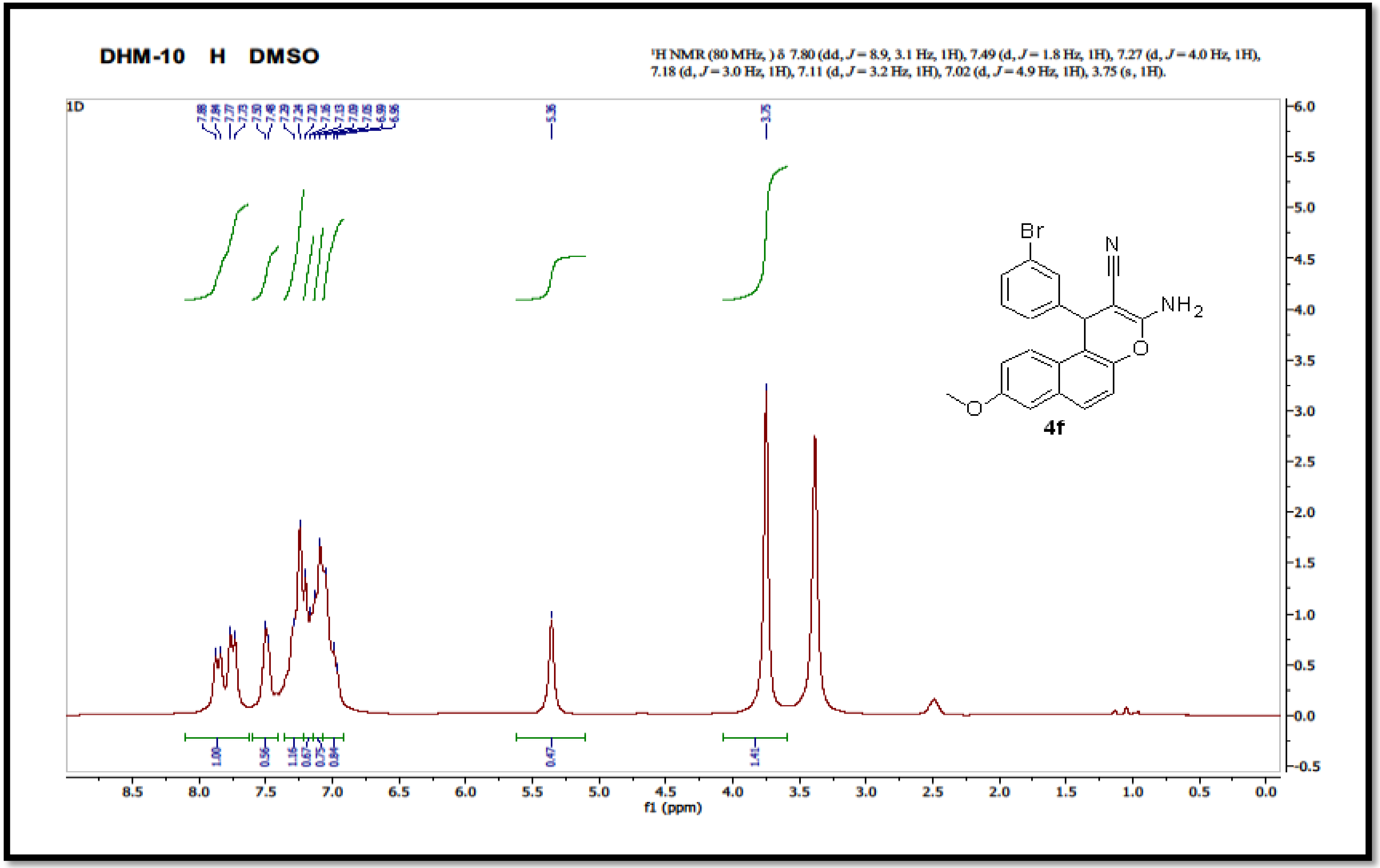

Figure S23: ^{1}H NMR spectrum (DMSO-d_6, 500 MHz) of compound **4f**.

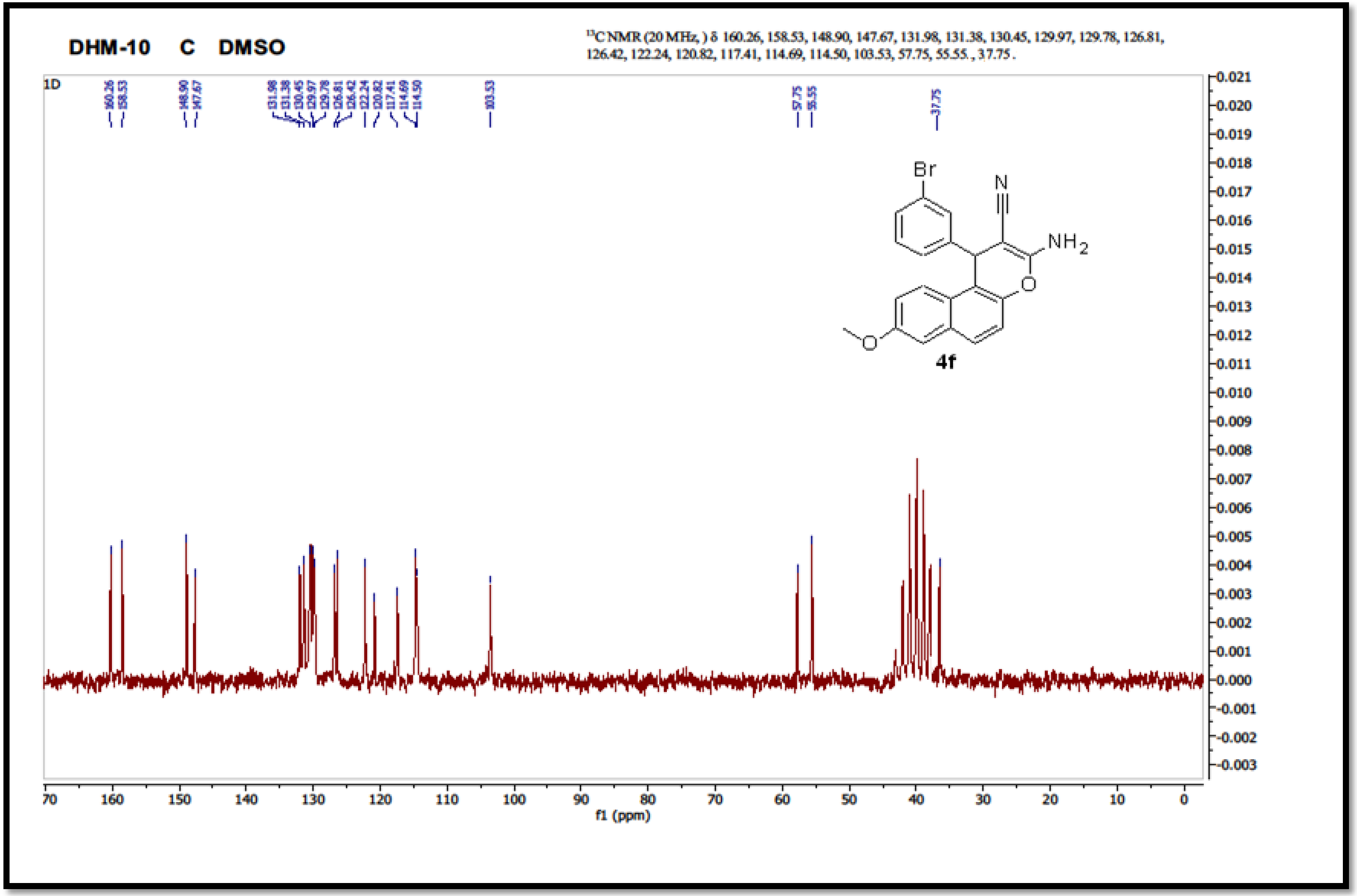

Figure S24: ^{1}H NMR spectrum (DMSO-d_6, 500 MHz) of compound **4f**.

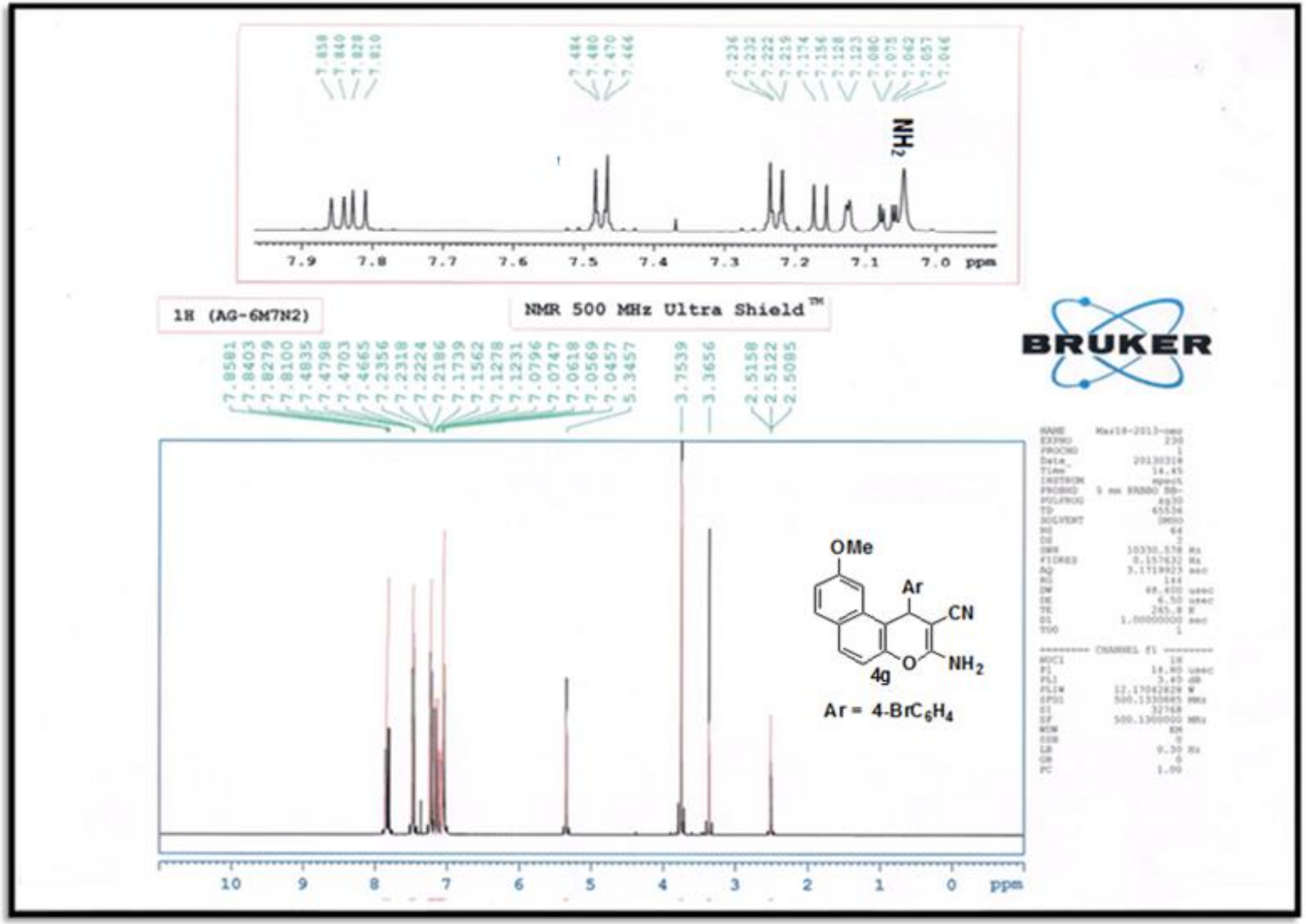

Figure S25: ^{1}H NMR spectrum (DMSO-d_6, 500 MHz) of compound **4g**.

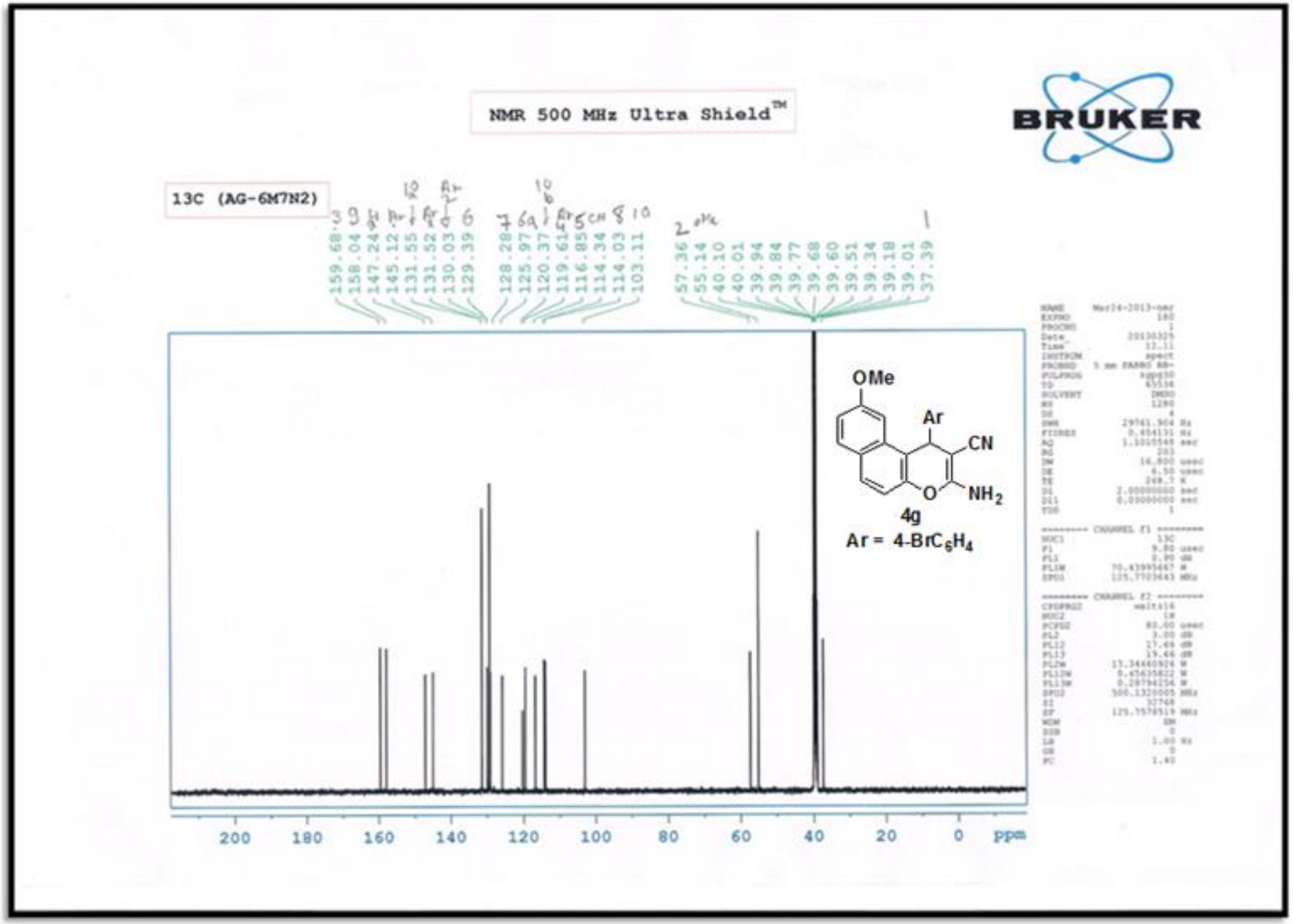

Figure S26: ^{13}C NMR spectrum (DMSO-d_6, 125 MHz) of compound **4g**.

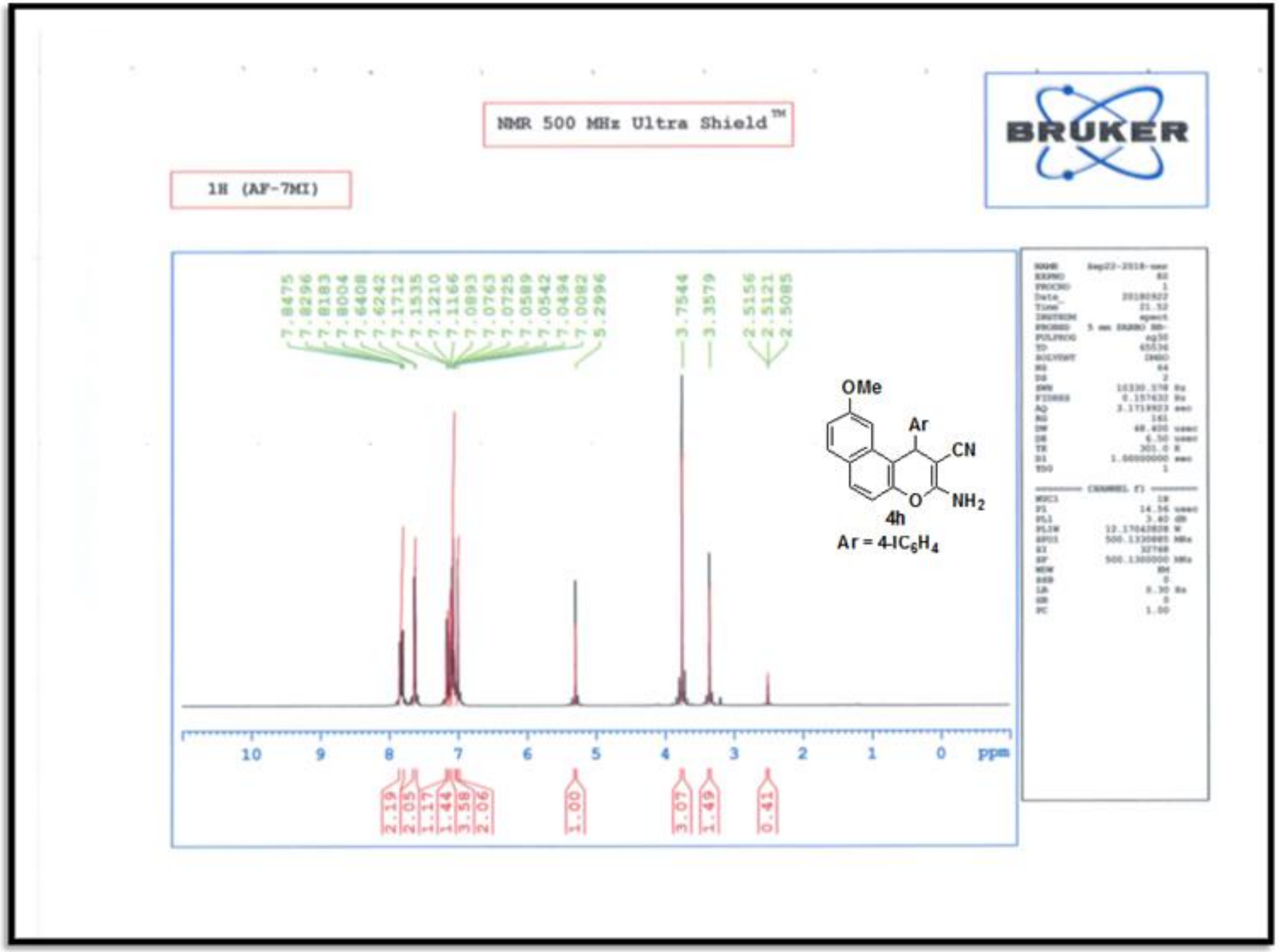

Figure S27: ^{1}H NMR spectrum (DMSO-d_6, 500 MHz) of compound **4h**.

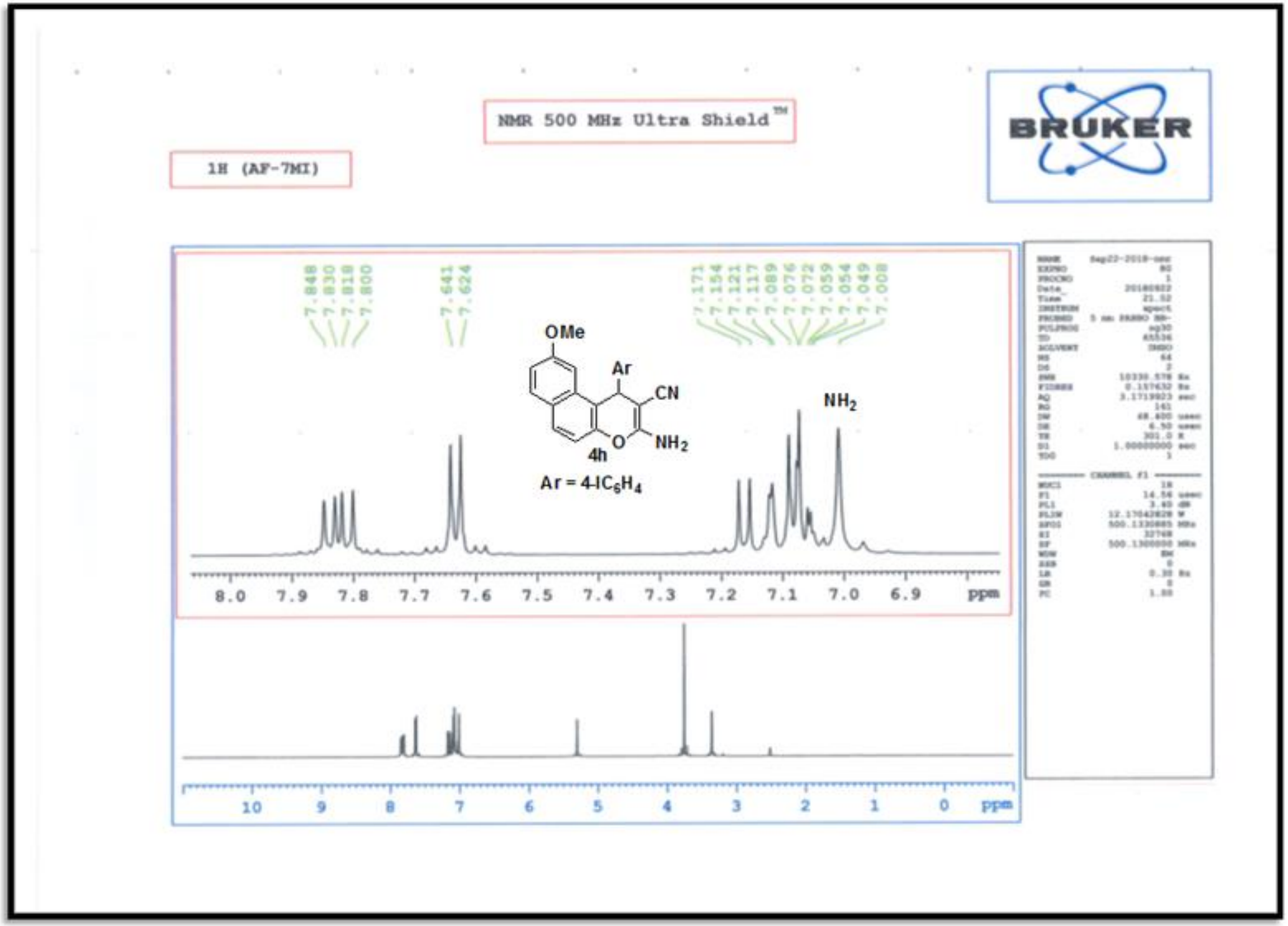

Figure S28: ^{1}H NMR spectrum (DMSO-d_6, 500 MHz) of compound **4h**.

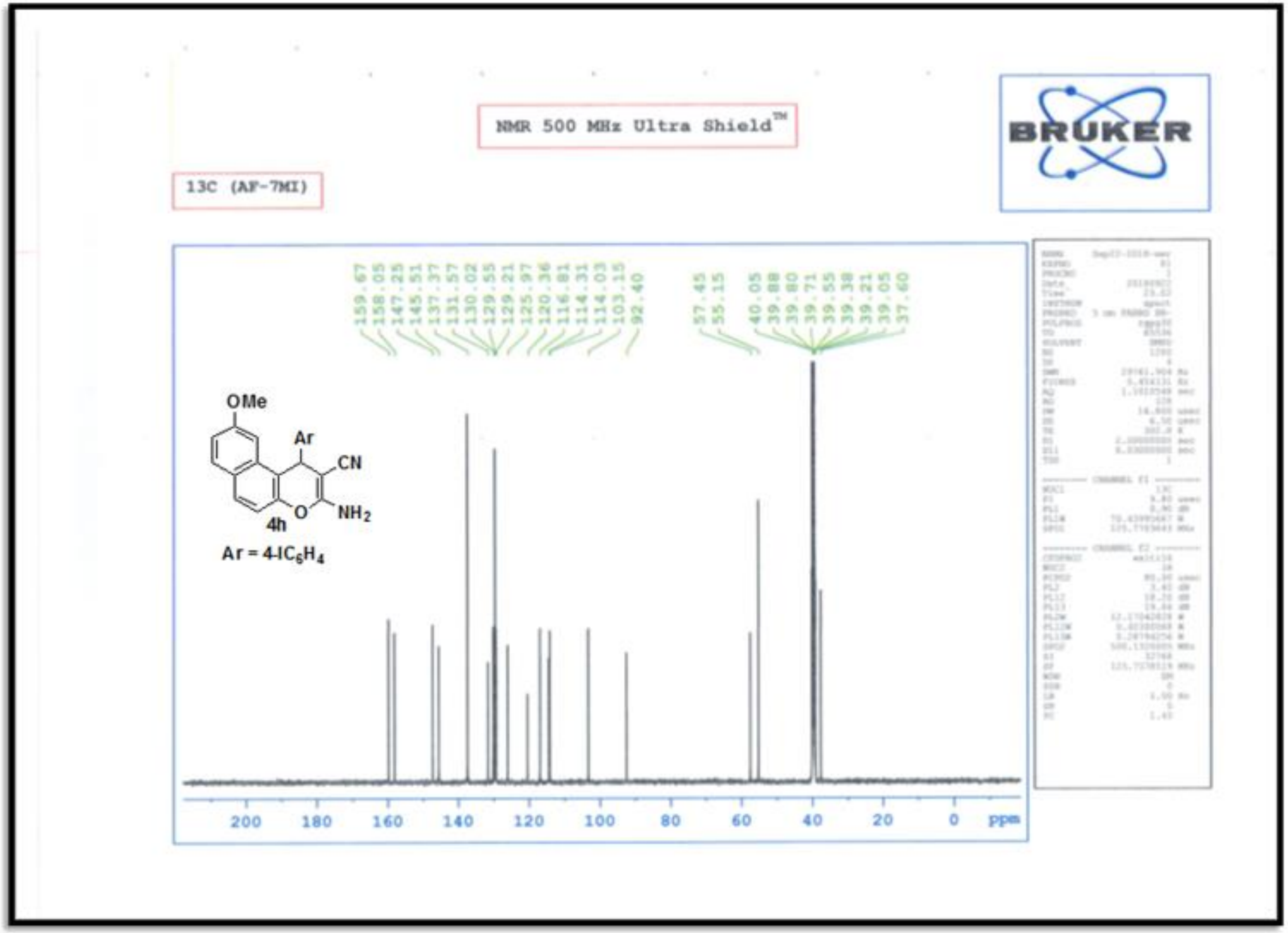

Figure S29: ^{13}C NMR spectrum (DMSO-d_6, 125 MHz) of compound **4h**.

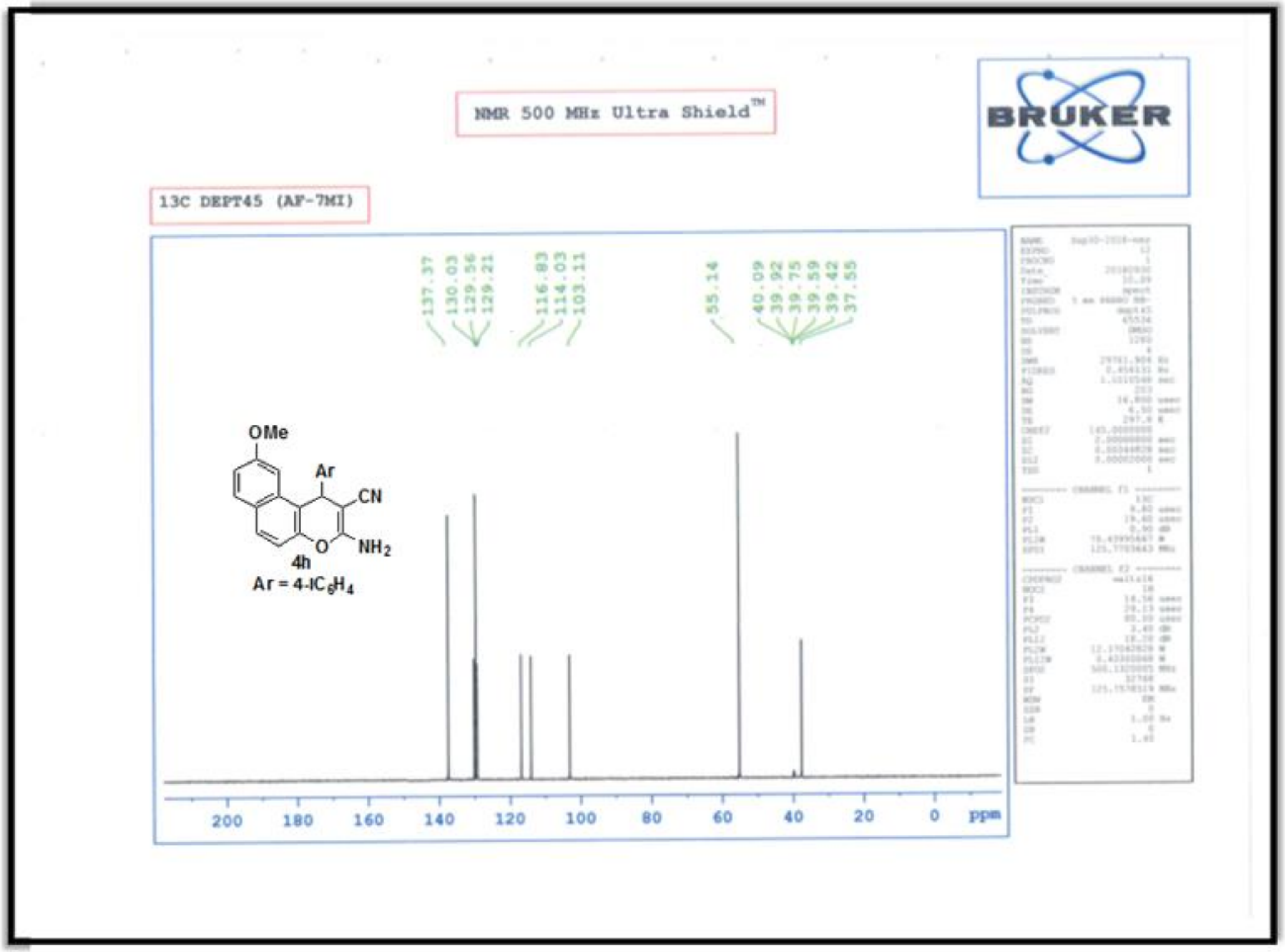

Figure S30: DEPT45 spectrum (DMSO-d_6, 125 MHz) of compound **4h.**

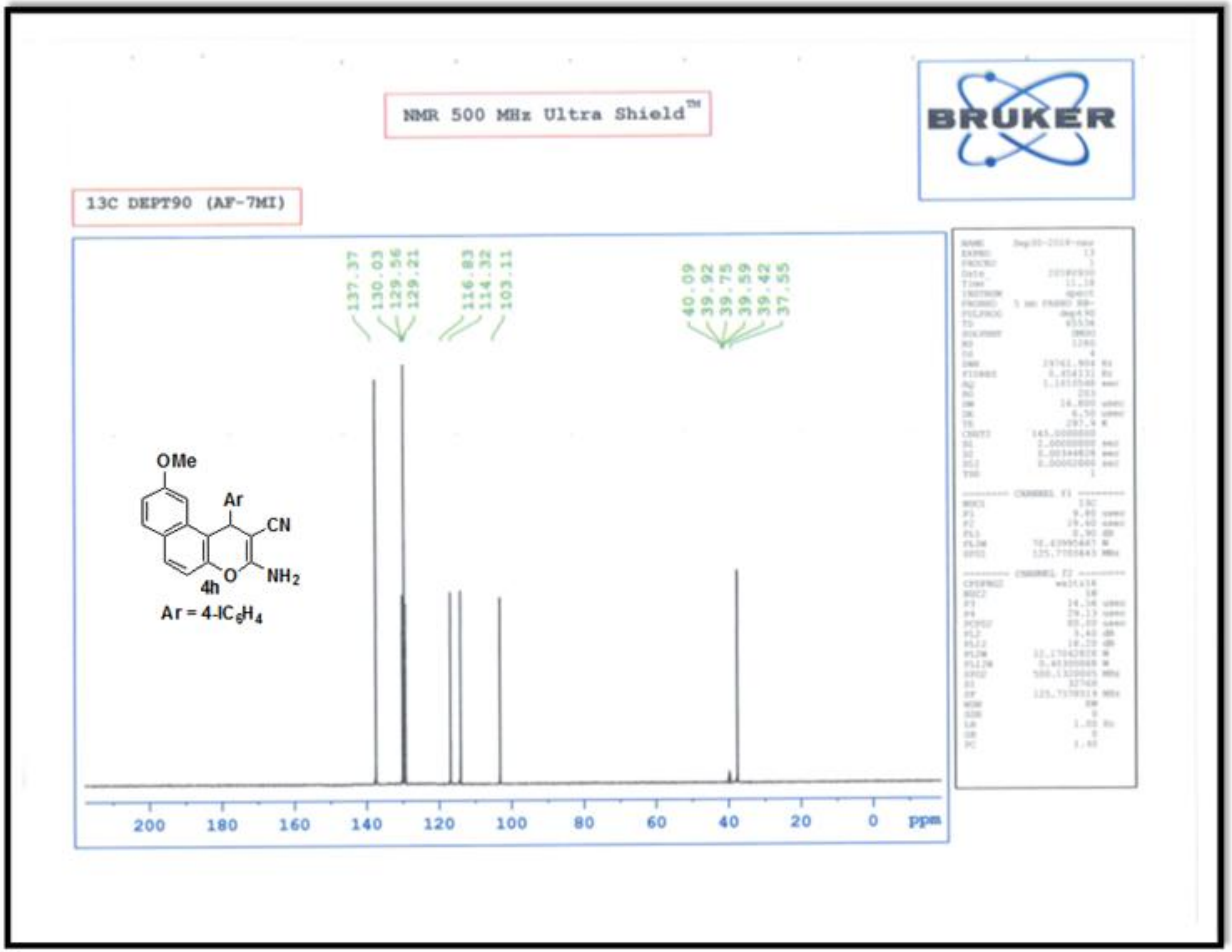

Figure S31: DEPT90 spectrum (DMSO-d_6, 125 MHz) of compound **4h.**

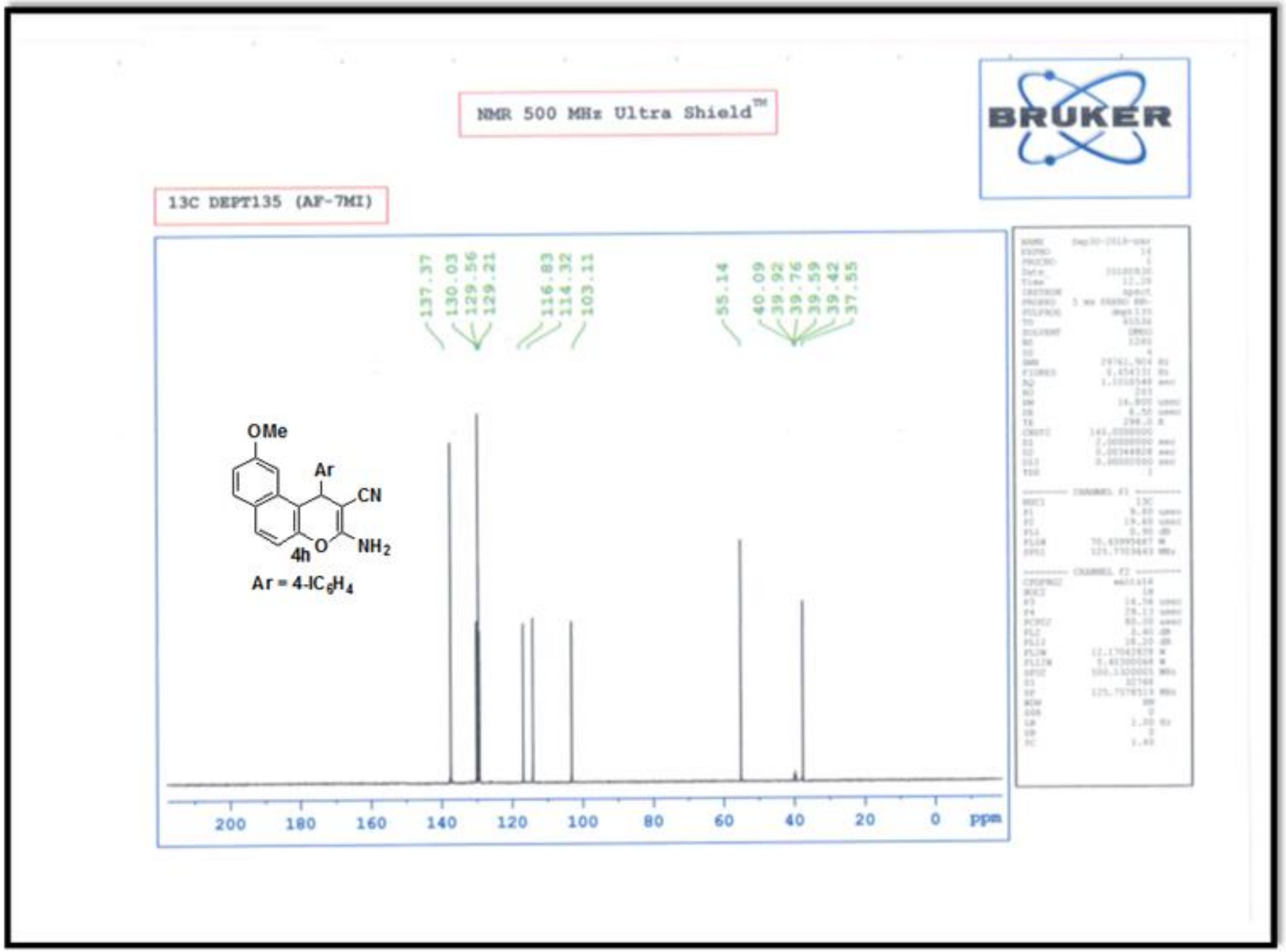

Figure S32: DEPT135 spectrum (DMSO-d_6, 125 MHz) of compound **4h.**

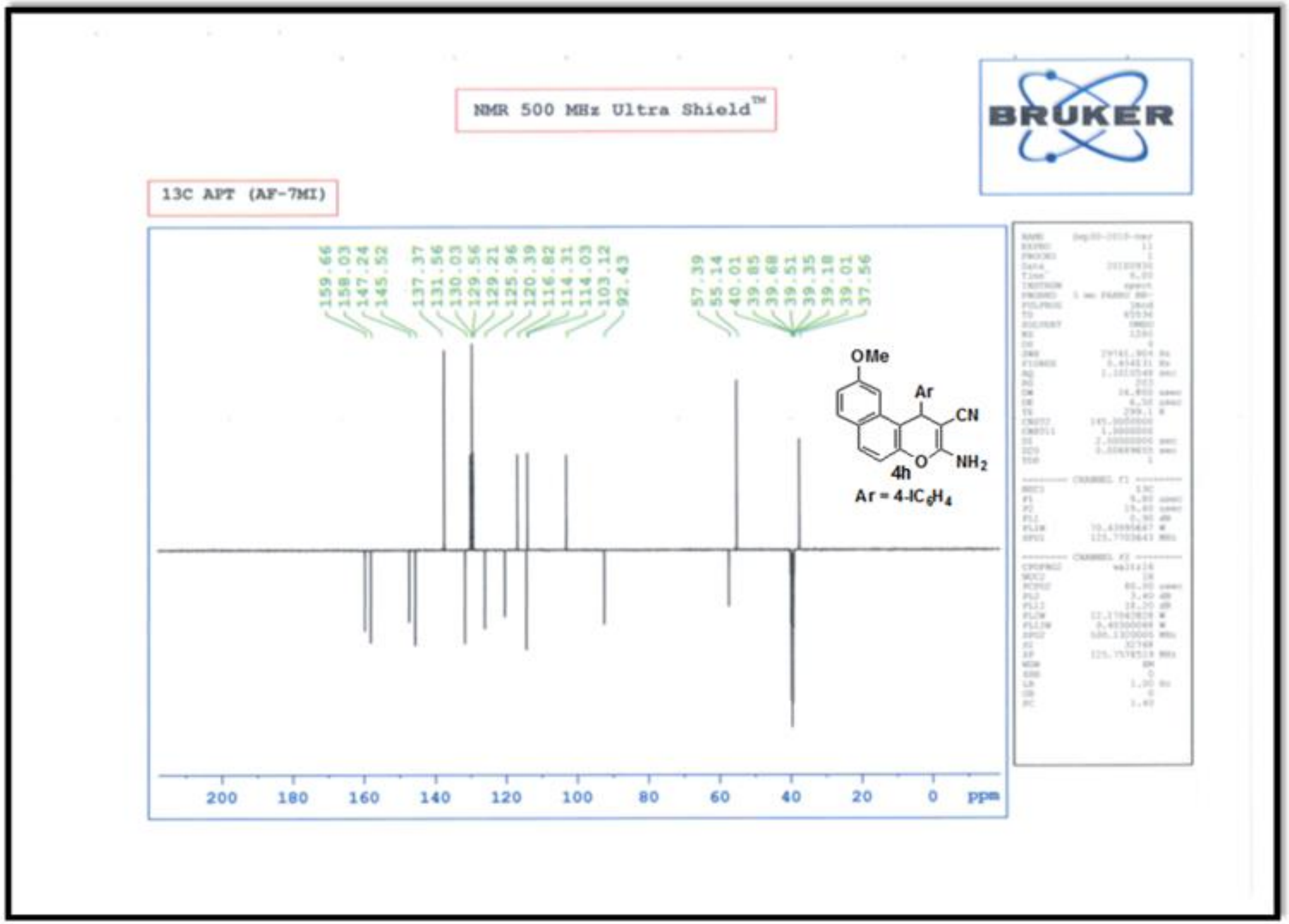

Figure S33: APT spectrum (DMSO-d_6, 125 MHz) of compound **4h.**.

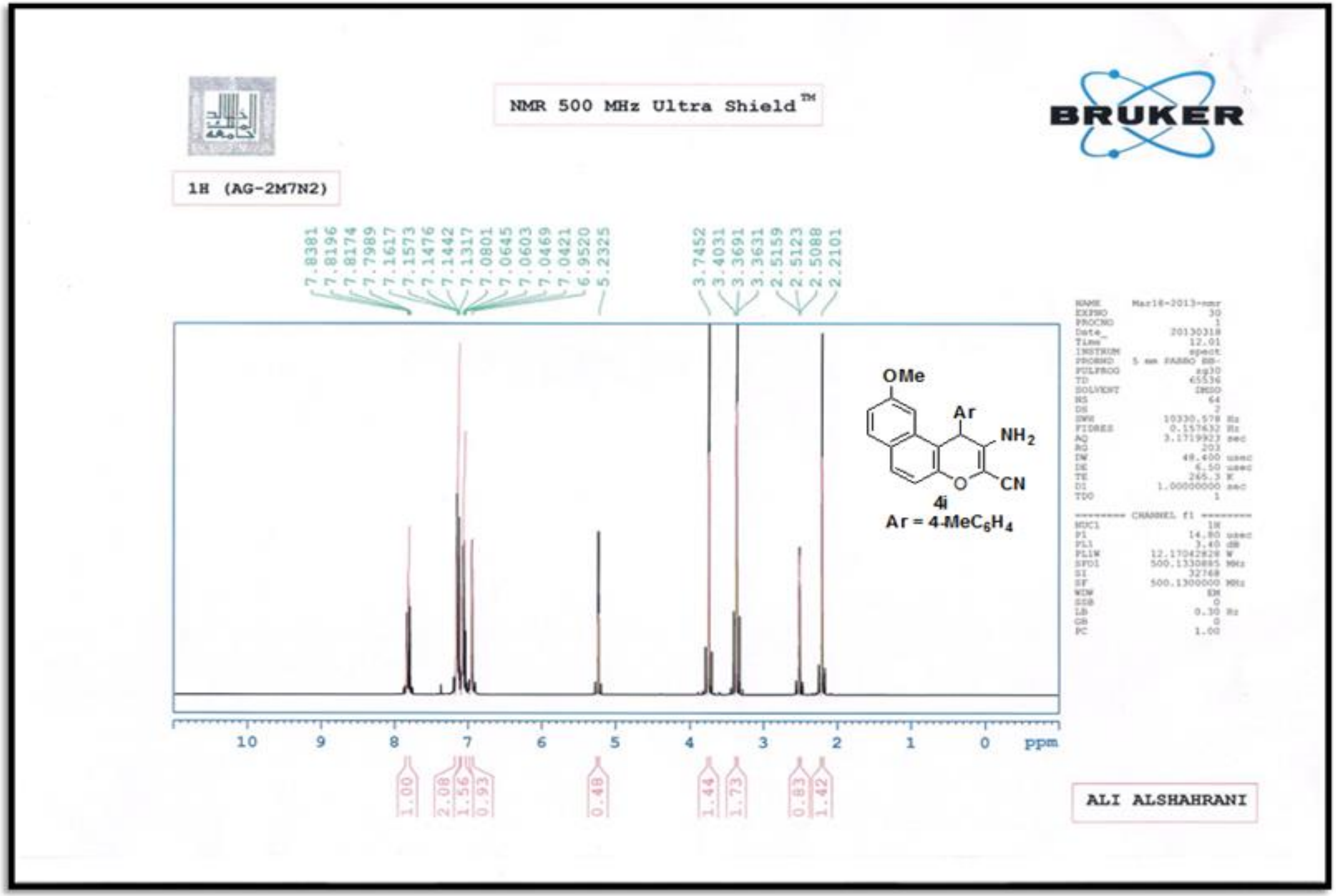

Figure S34: ^{1}H NMR spectrum (DMSO-d_6, 500 MHz) of compound **4i**

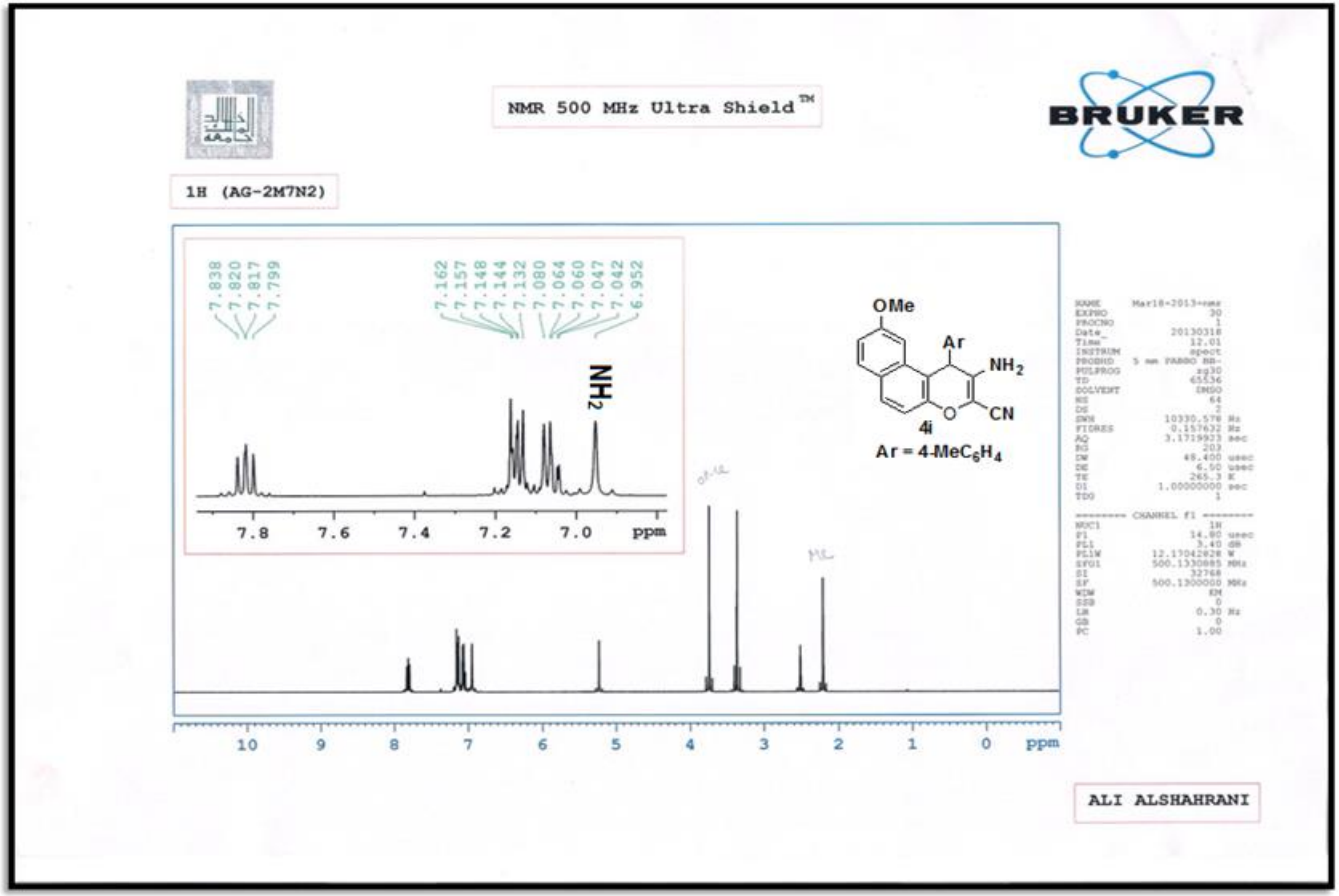

Figure S35: ^{1}H NMR spectrum (DMSO-d_6, 500 MHz) of compound **4i**.

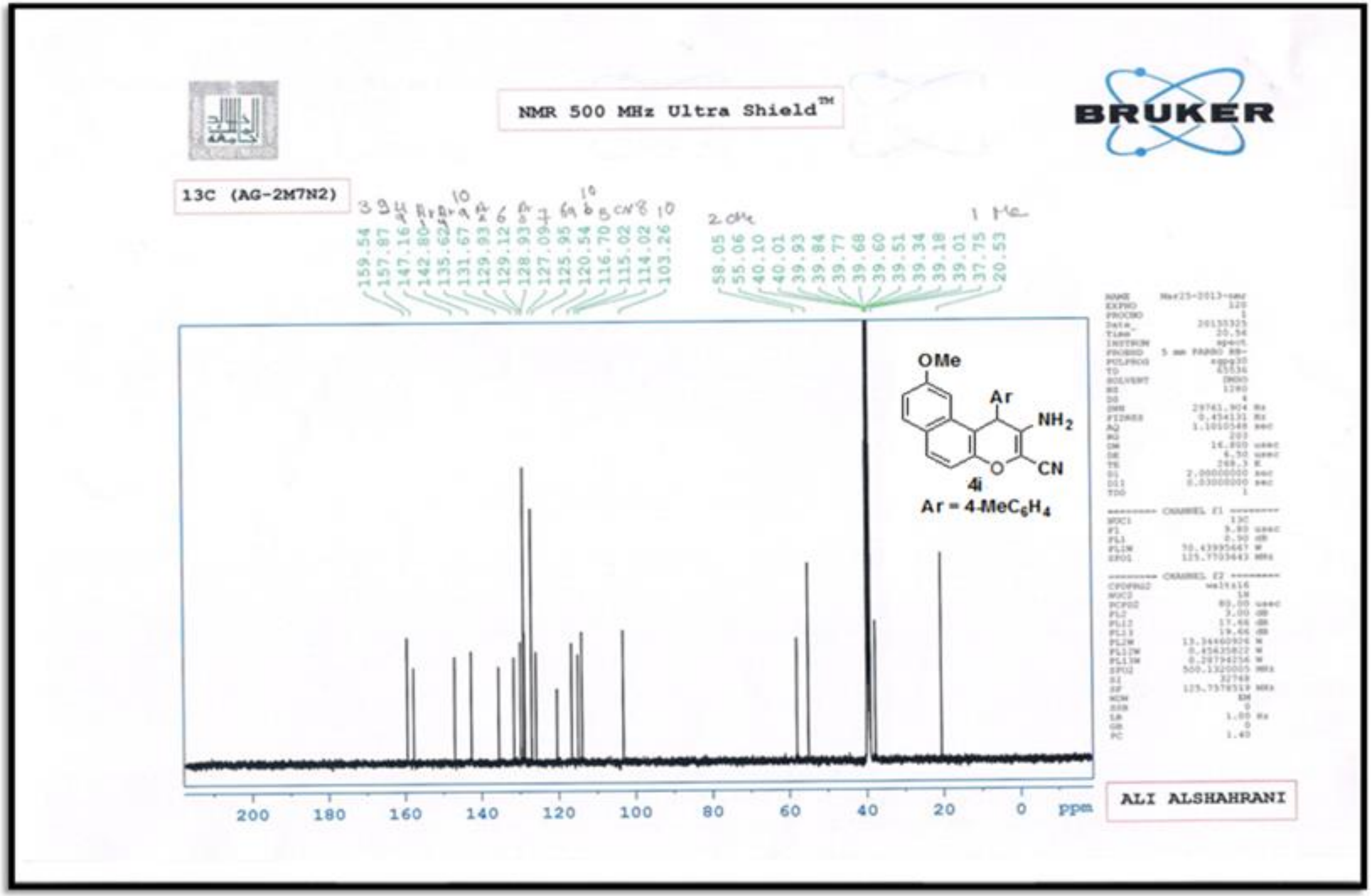

Figure S36: ^{13}C NMR spectrum (DMSO-d_6, 125 MHz) of compound **4i**.

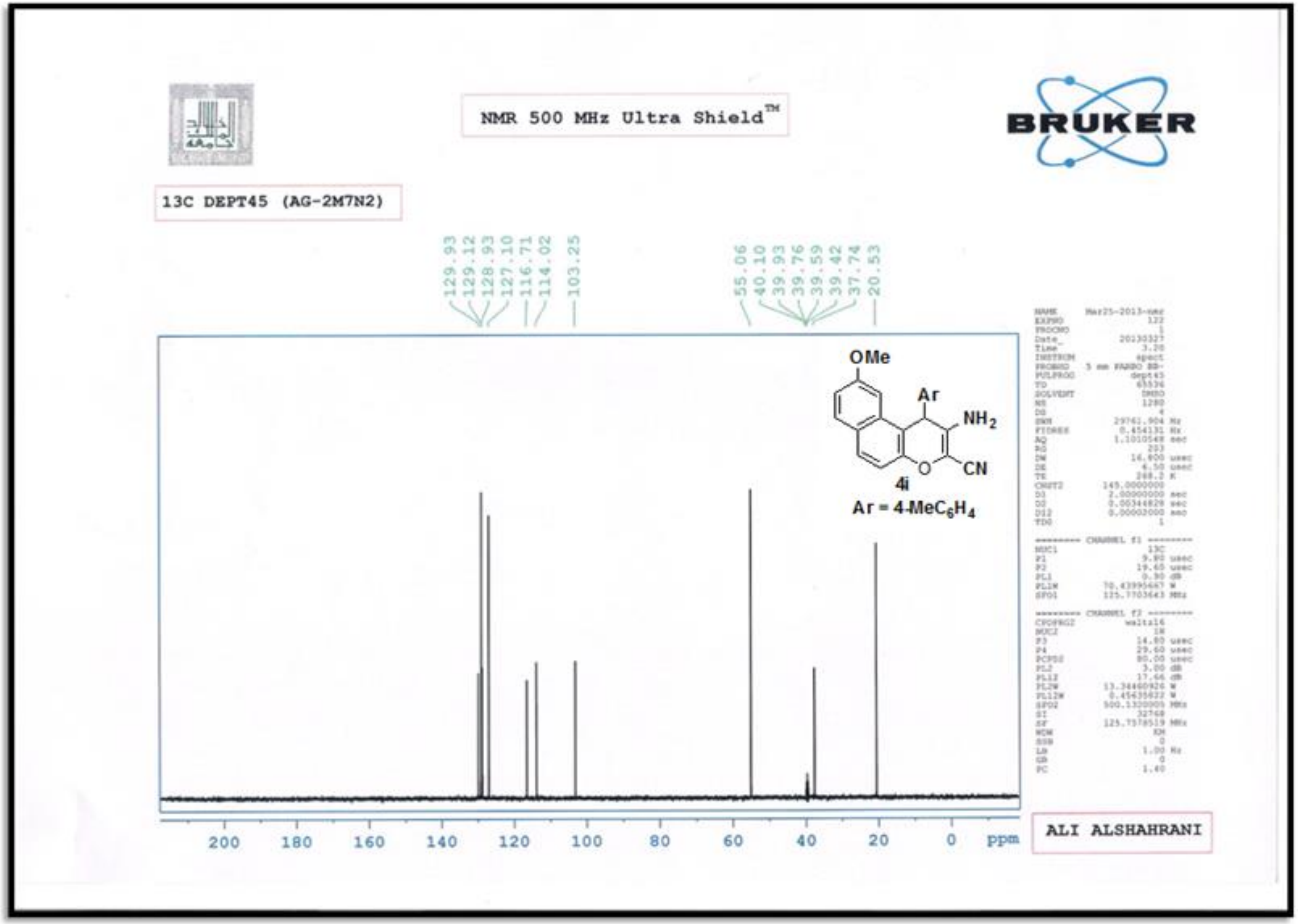

Figure S37: DEPT45 spectrum (DMSO-d_6, 125 MHz) of compound **4i.**

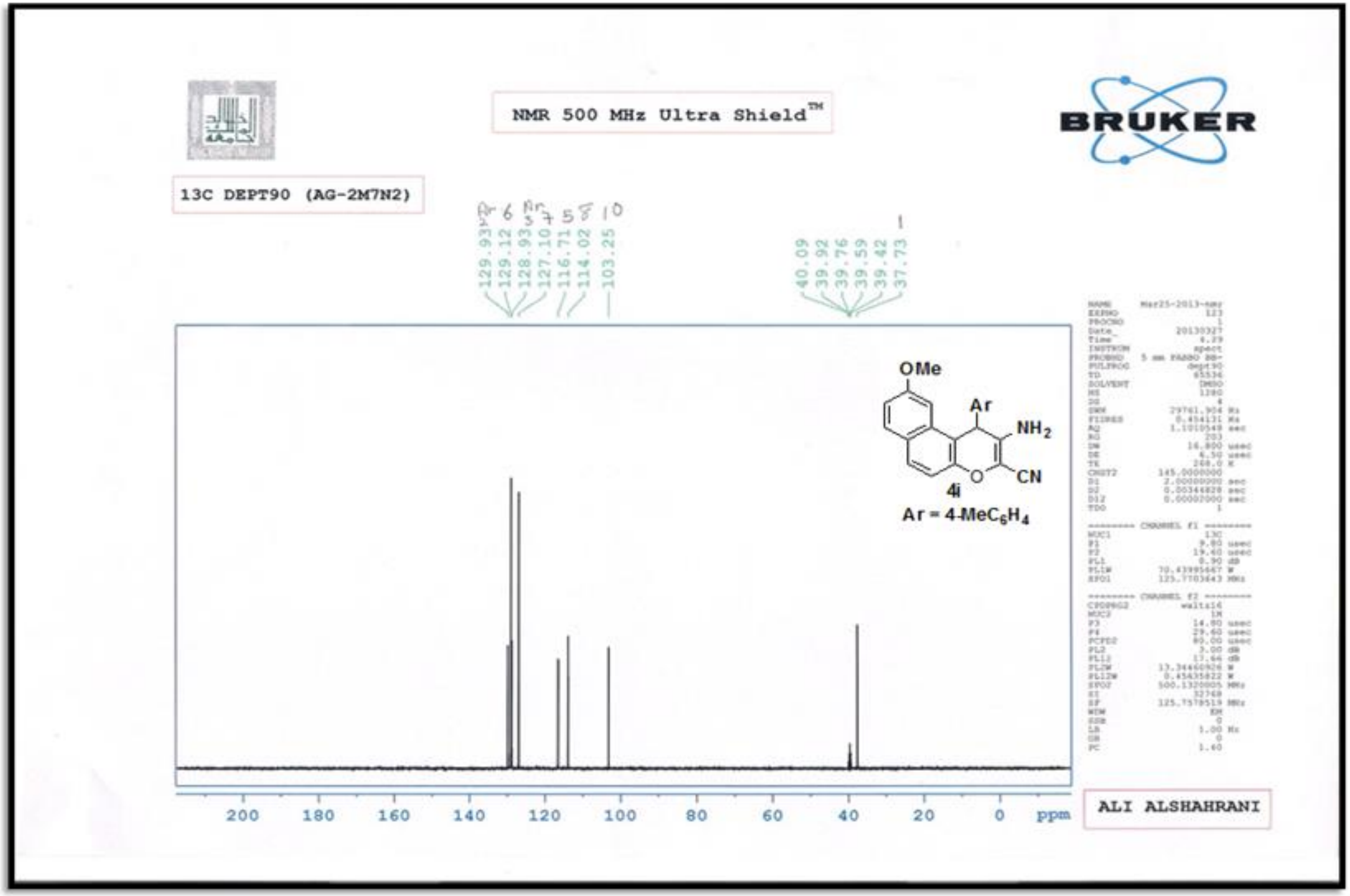

Figure S38: DEPT90 spectrum (DMSO-d_6, 125 MHz) of compound **4i.**

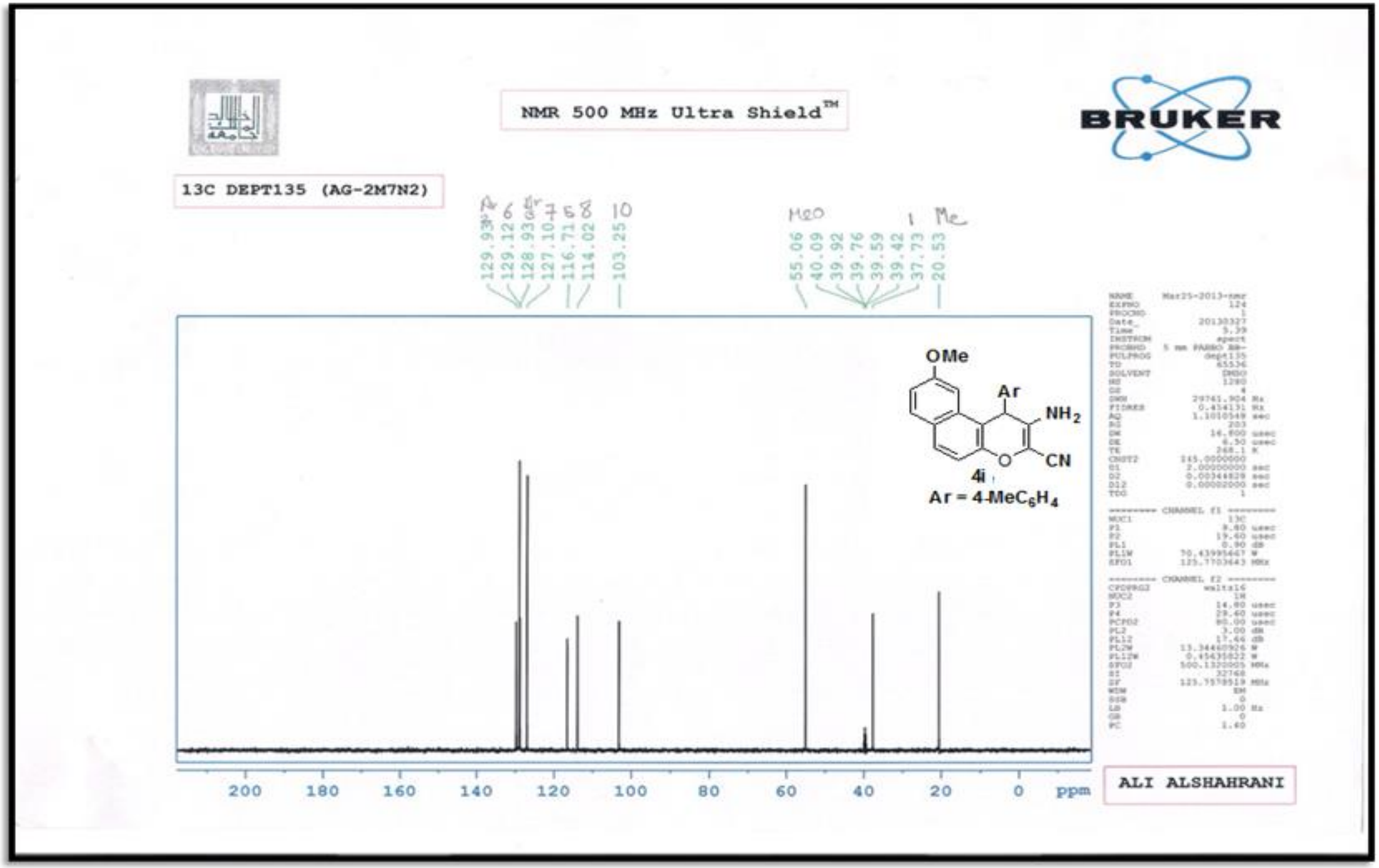

Figure S39: DEPT135 spectrum (DMSO-d_6, 125 MHz) of compound **4i.**

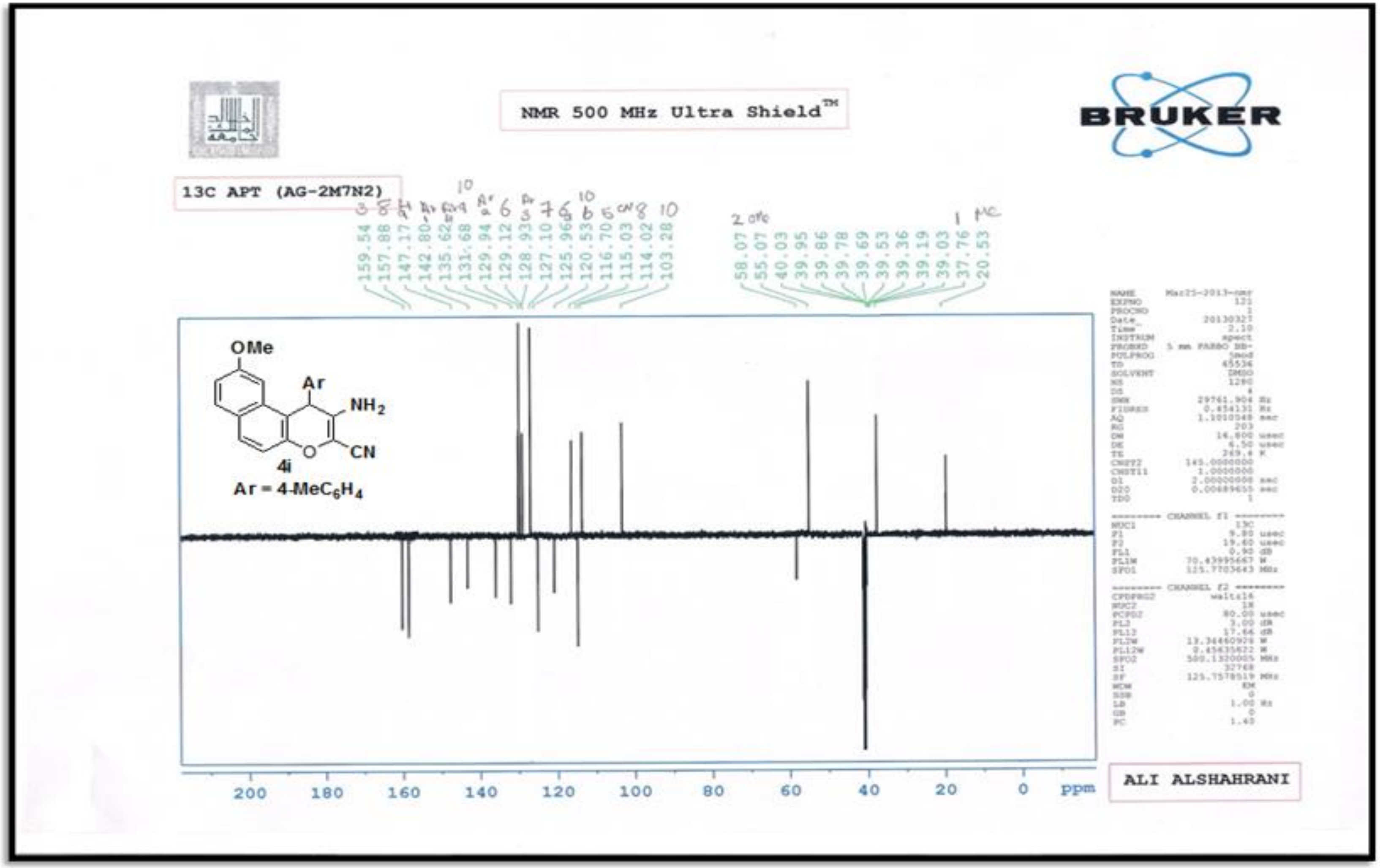

Figure S40: APT spectrum (DMSO-d_6, 125 MHz) of compound **4i**

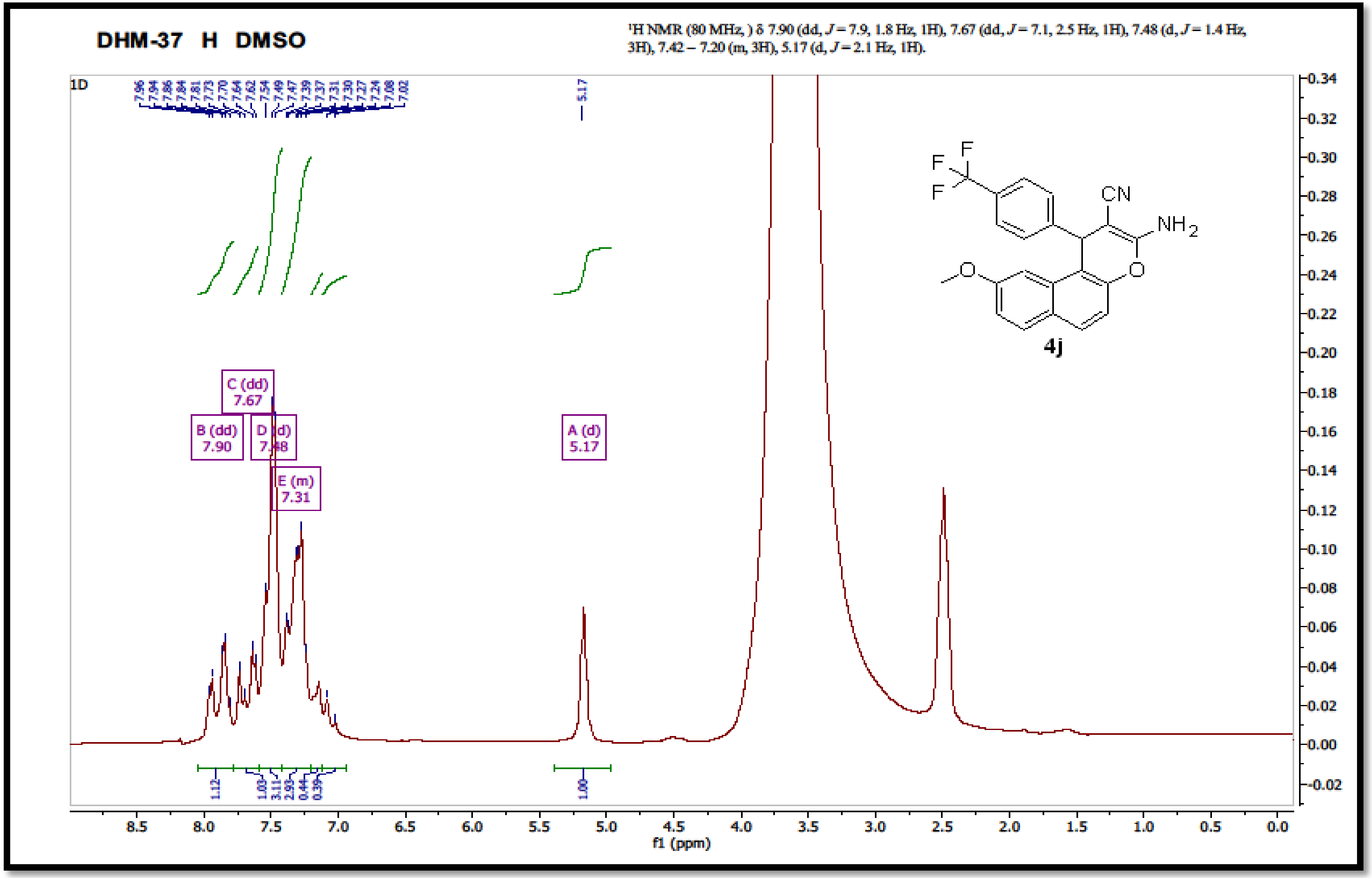

Figure S41: ^{1}H NMR spectrum (DMSO-d_6, 500 MHz) of compound **4j**.

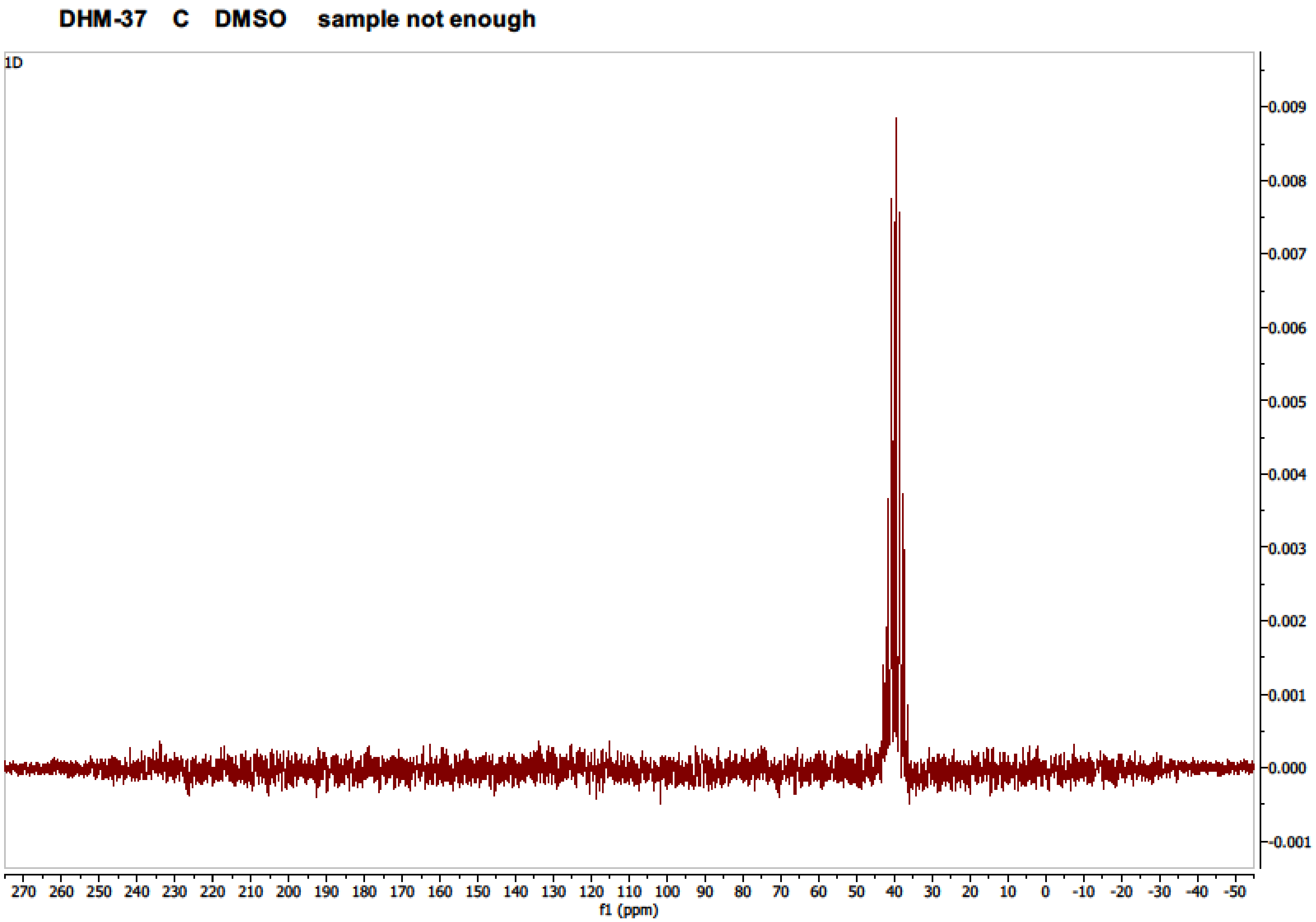

Figure S42: ^{13}C NMR spectrum (DMSO-d_6, 125 MHz) of compound **4j**.

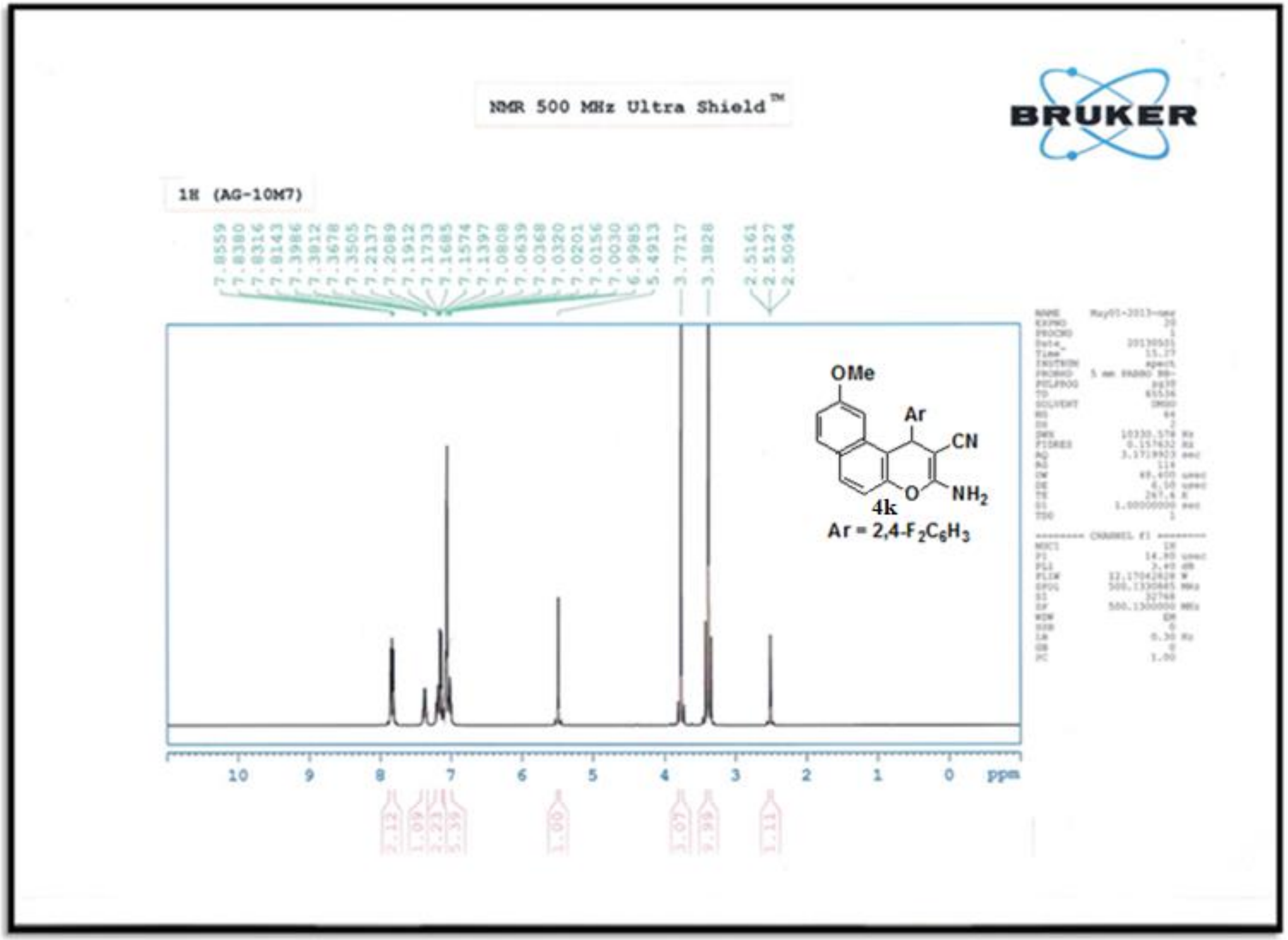

Figure S43: ^{1}H NMR spectrum (DMSO-d_6, 500 MHz) of compound **4k**.

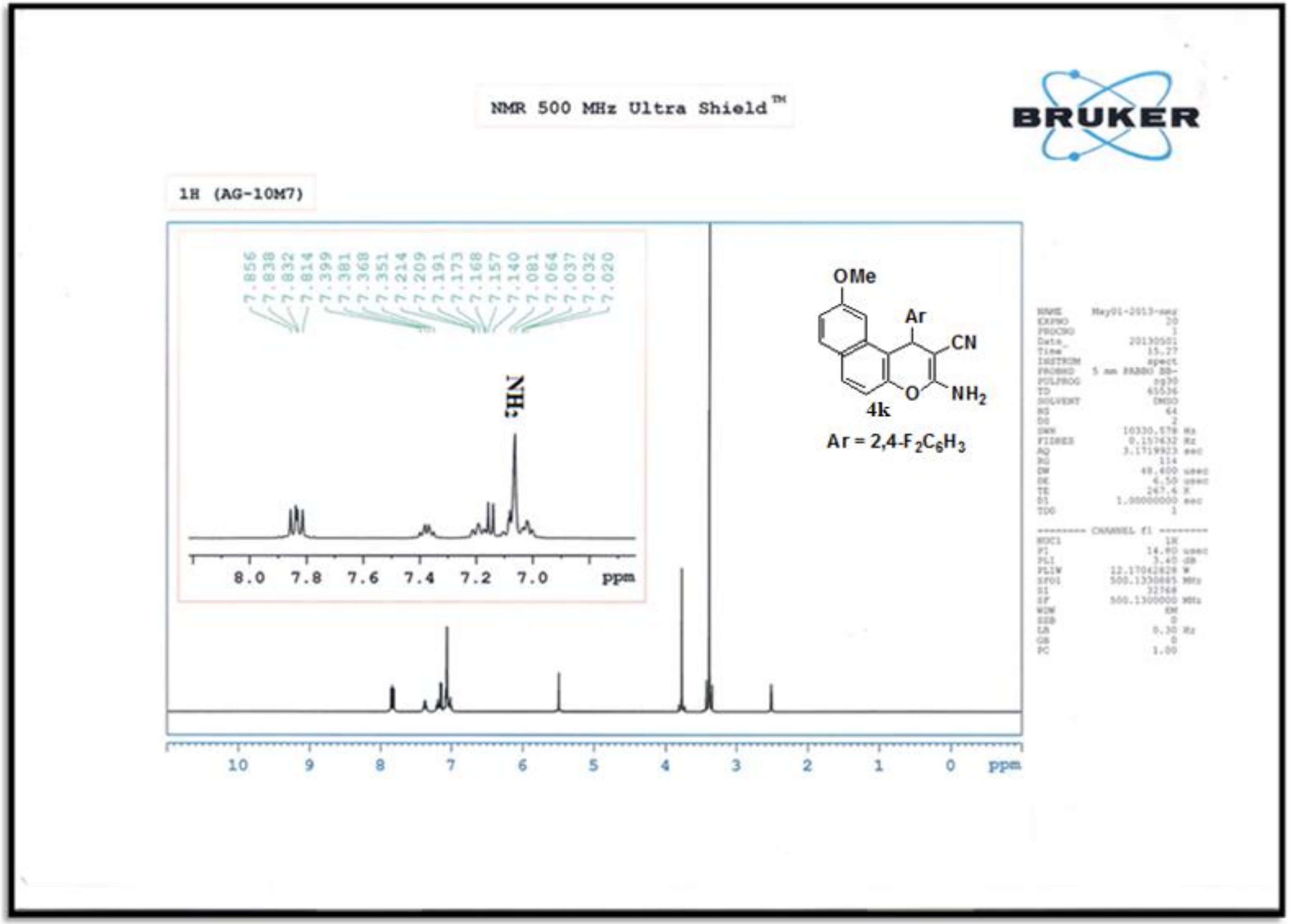

Figure S44: ^{1}H NMR spectrum (DMSO-d_6, 500 MHz) of compound **4k**.

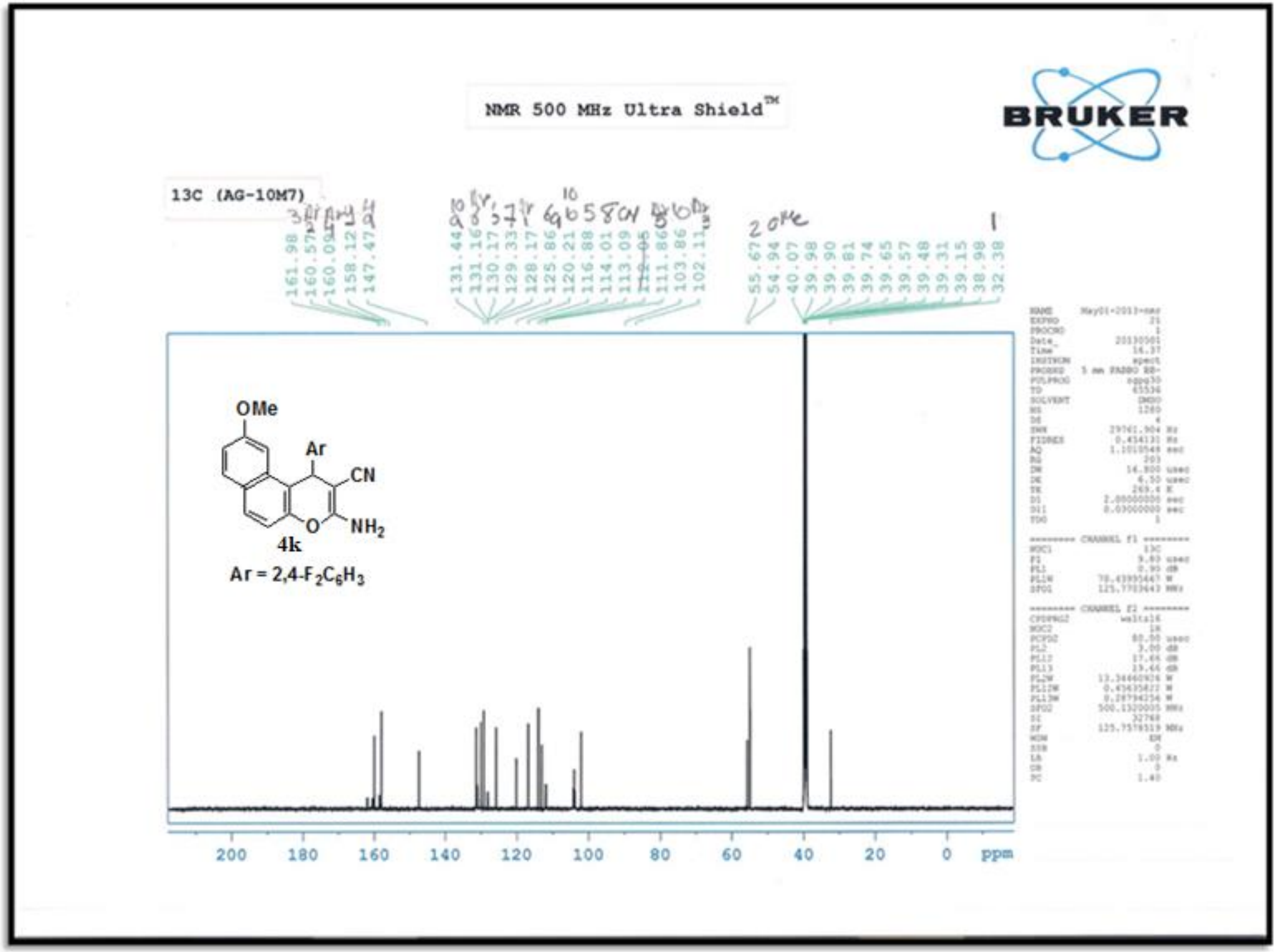

Figure S45: ^{13}C NMR spectrum (DMSO-d_6, 125 MHz) of compound **4k**.

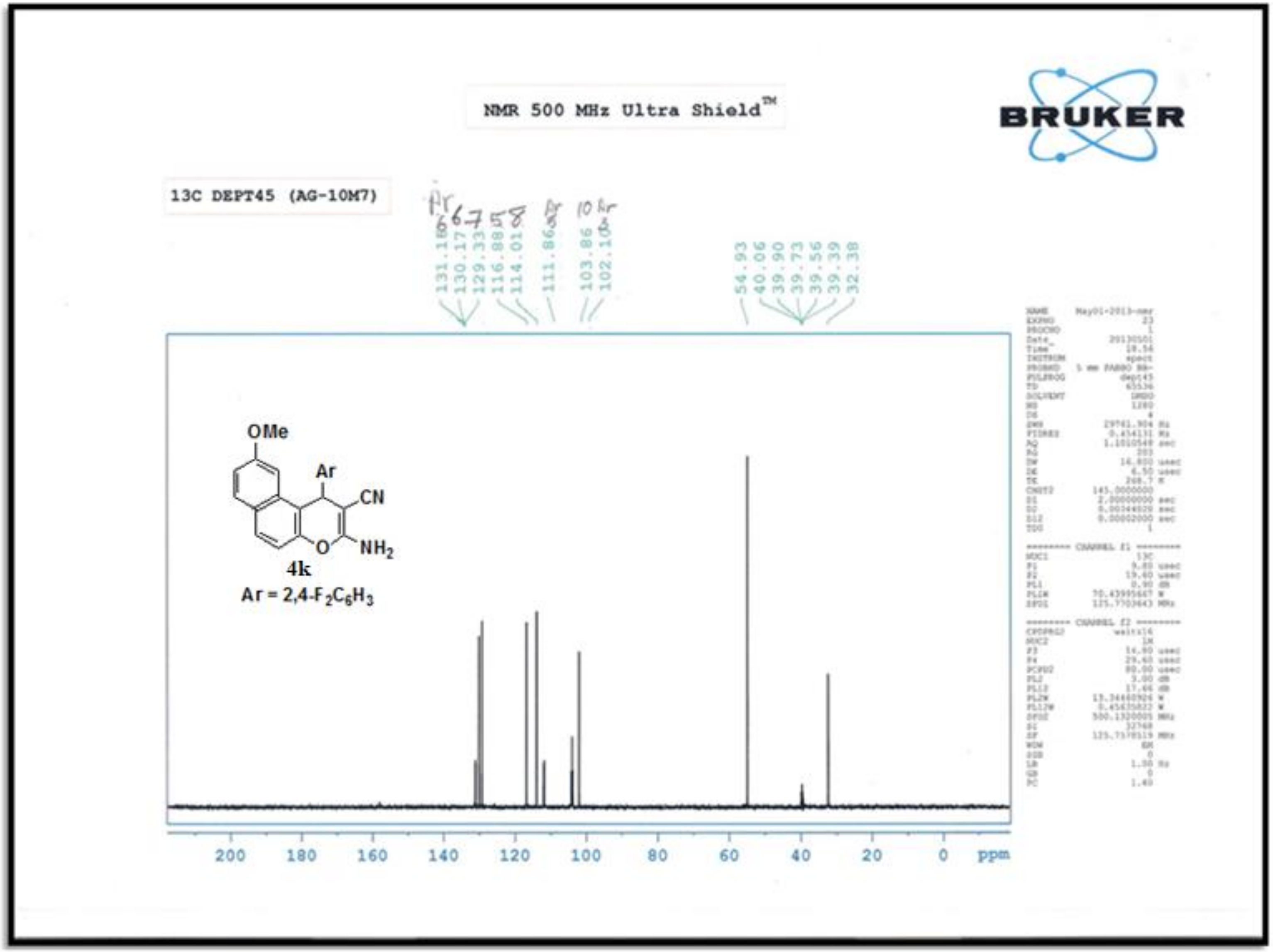

Figure S46: DEPT45 spectrum (DMSO-d_6, 125 MHz) of compound **4k.**

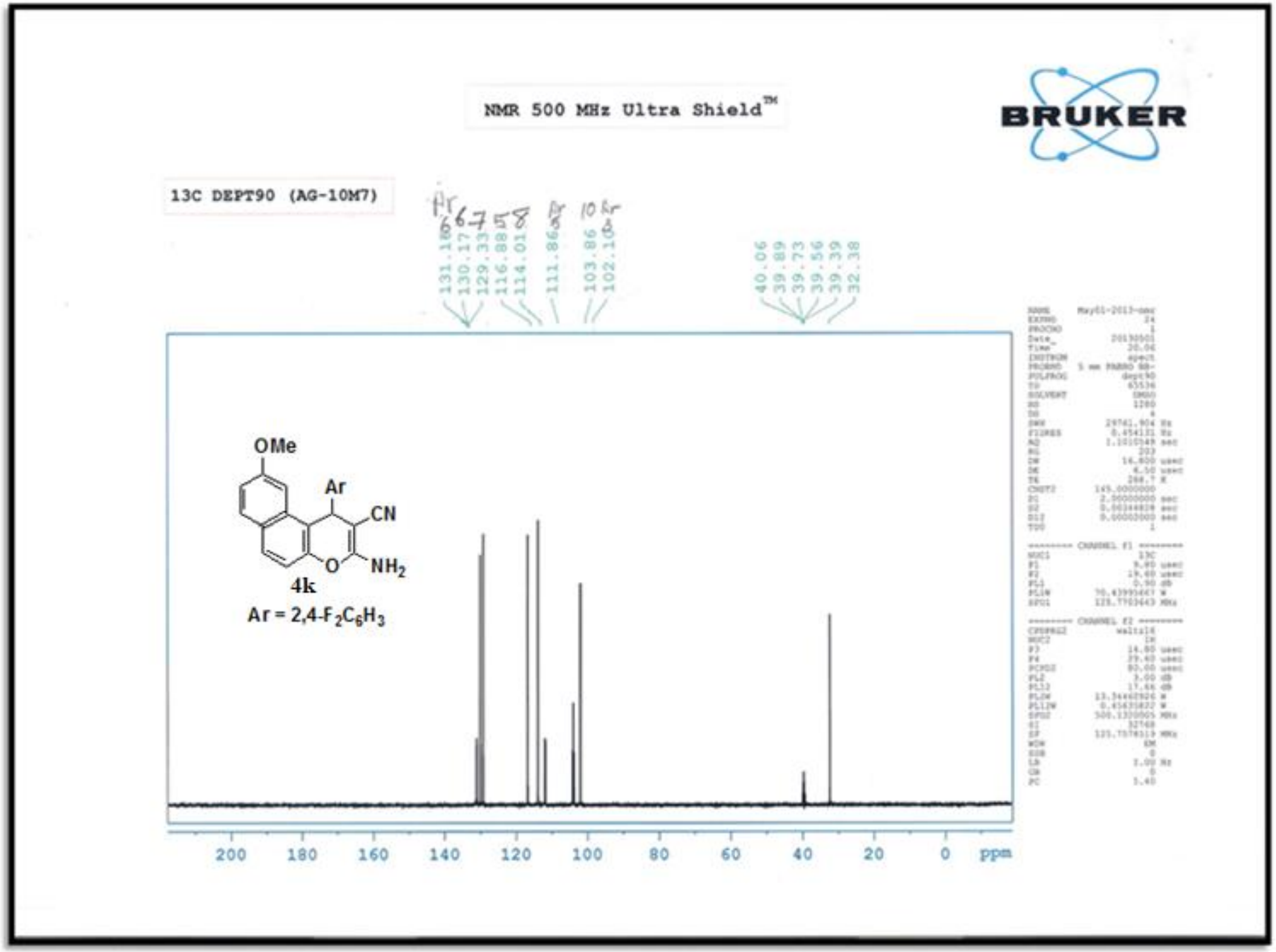

Figure S47: DEPT90 spectrum (DMSO-d_6, 125 MHz) of compound **4k.**

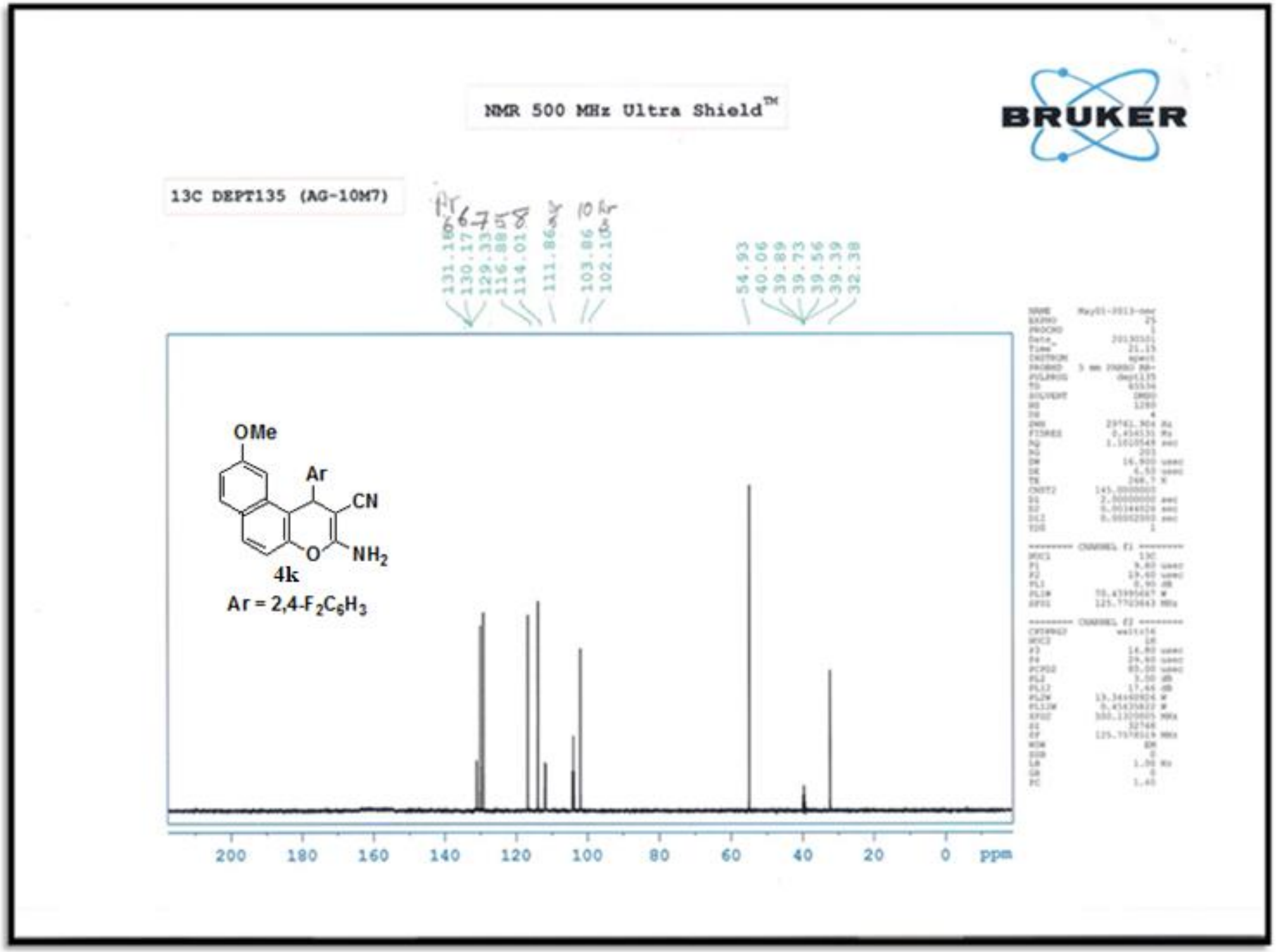

Figure S48: DEPT135 spectrum (DMSO-d_6, 125 MHz) of compound **4k.**

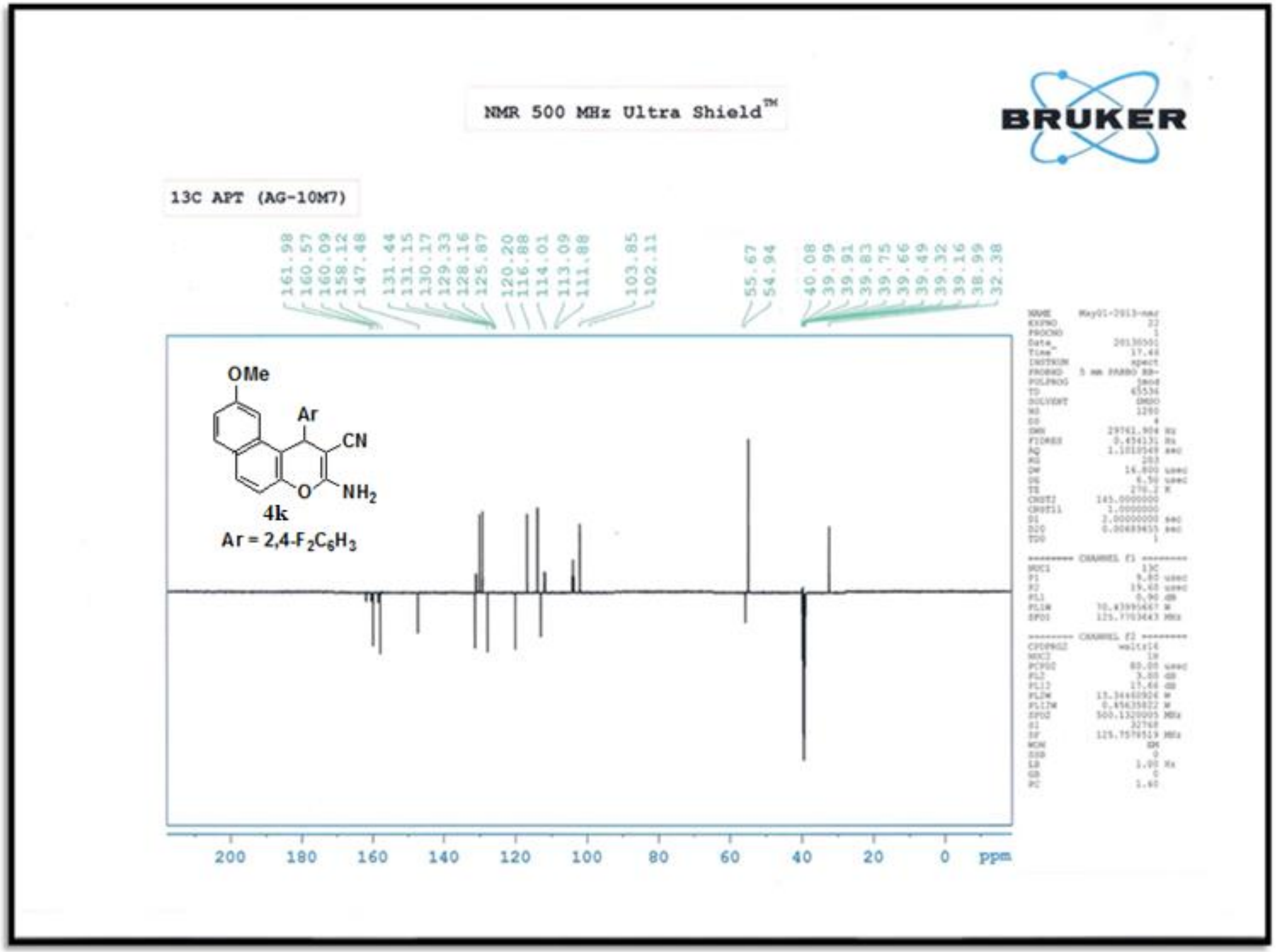

Figure S49: APT spectrum (DMSO-d_6, 125 MHz) of compound **4k**.

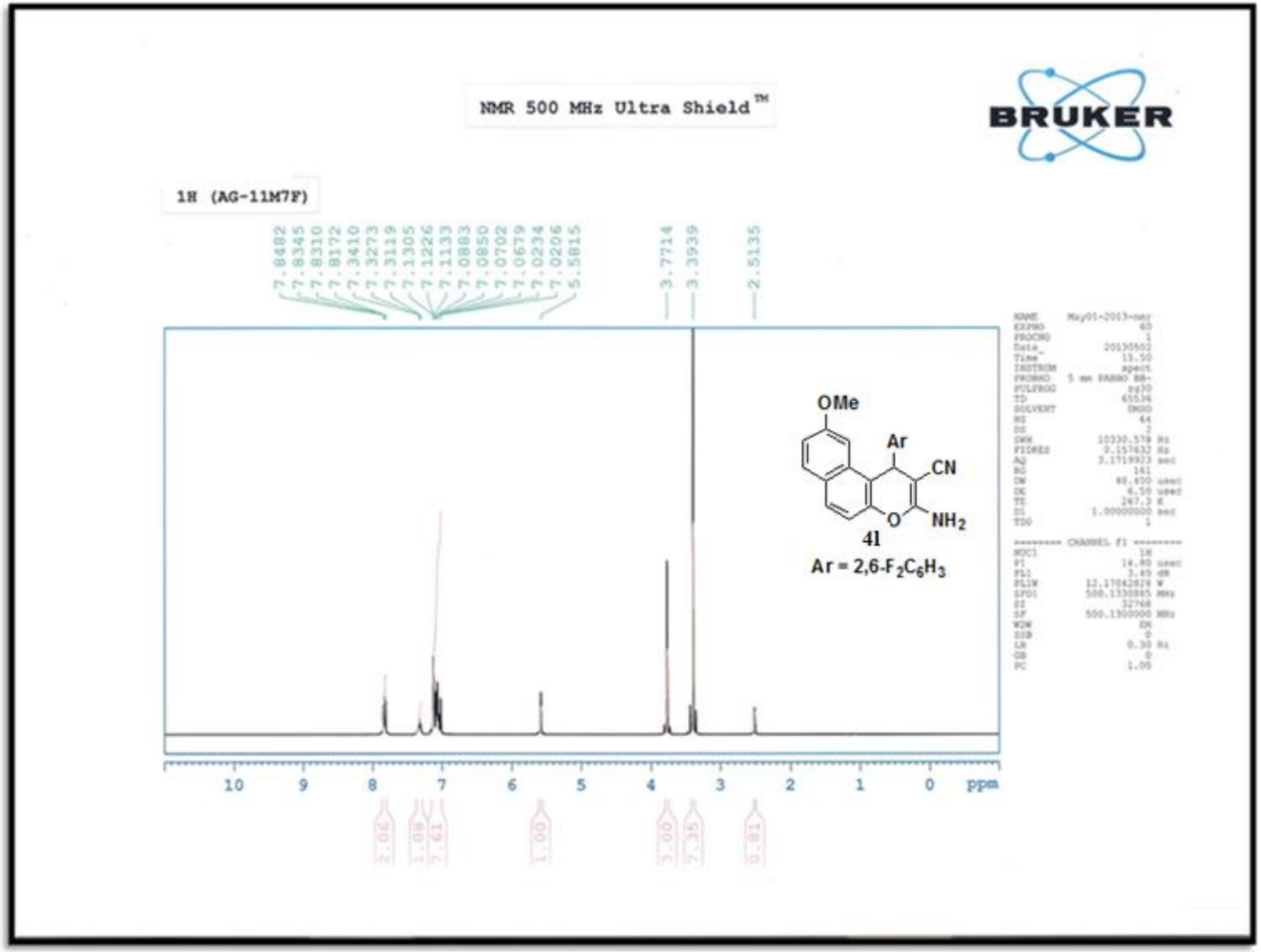

Figure S50: ^{1}H NMR spectrum (DMSO-d_6, 500 MHz) of compound **4l**.

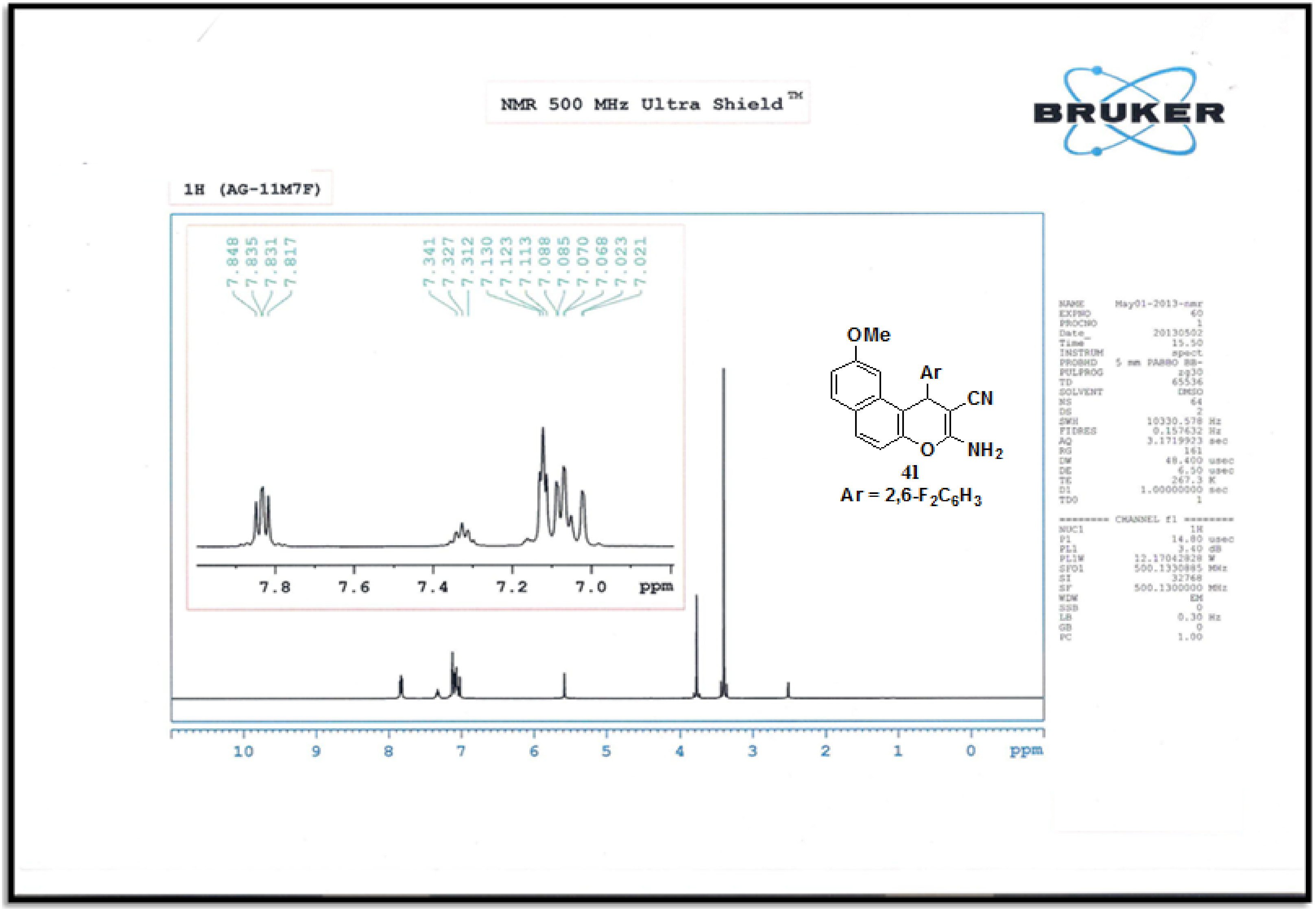

Figure S51: ^{1}H NMR spectrum (DMSO-d_6, 500 MHz) of compound **4l**.

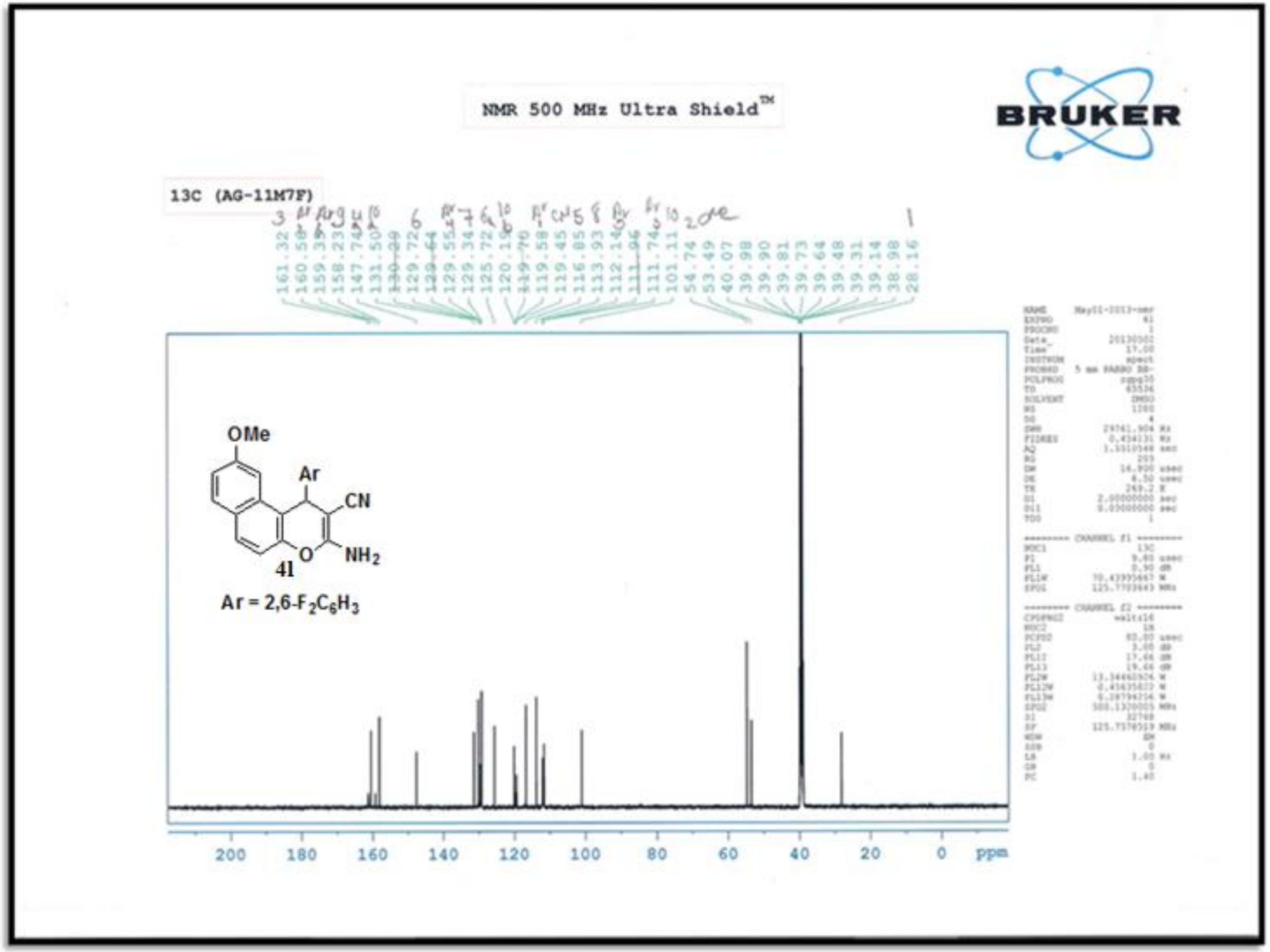

Figure S52: ^{13}C NMR spectrum (DMSO-d_6, 125 MHz) of compound **4l**.

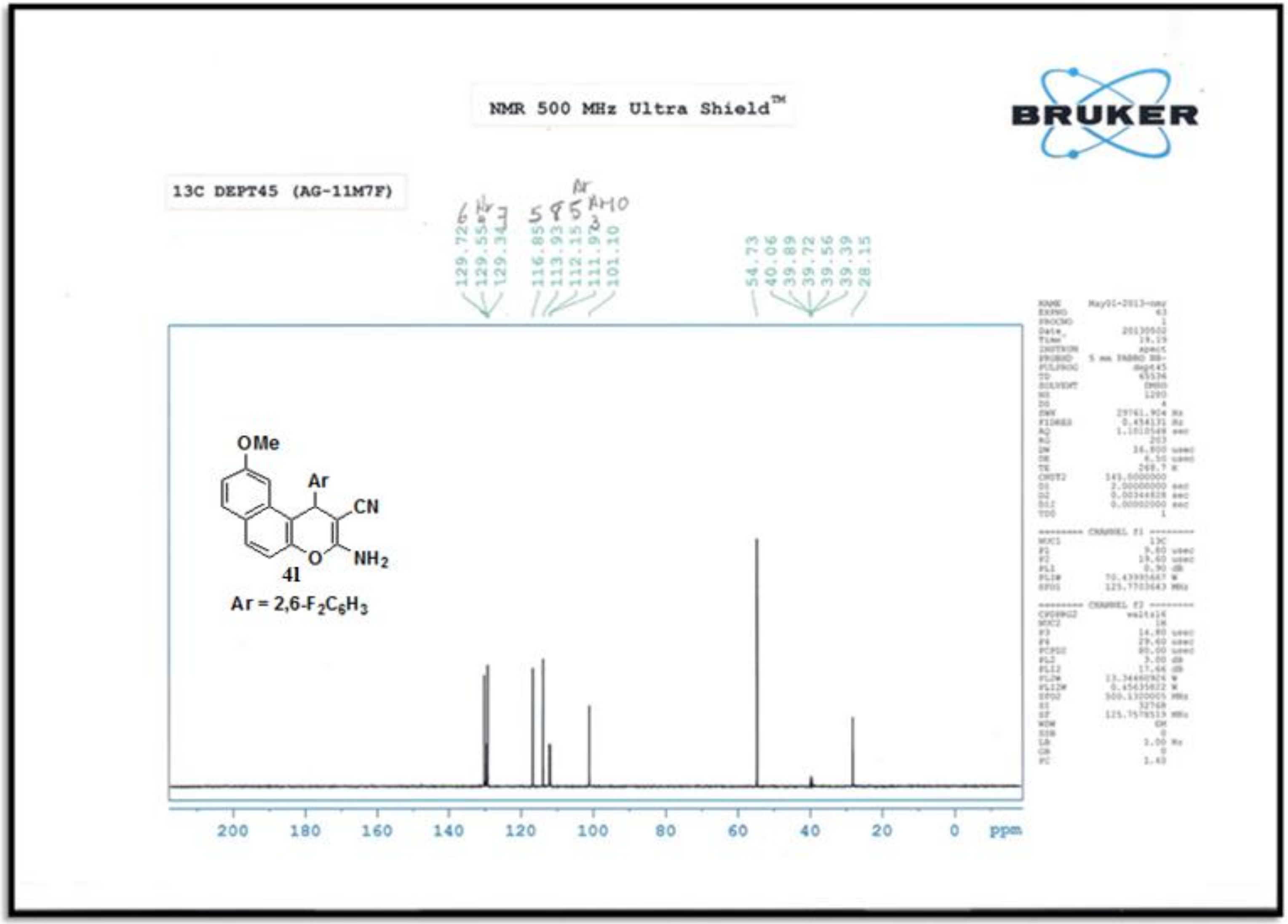

Figure S53: DEPT45 spectrum (DMSO-d_6, 125 MHz) of compound **4l.**

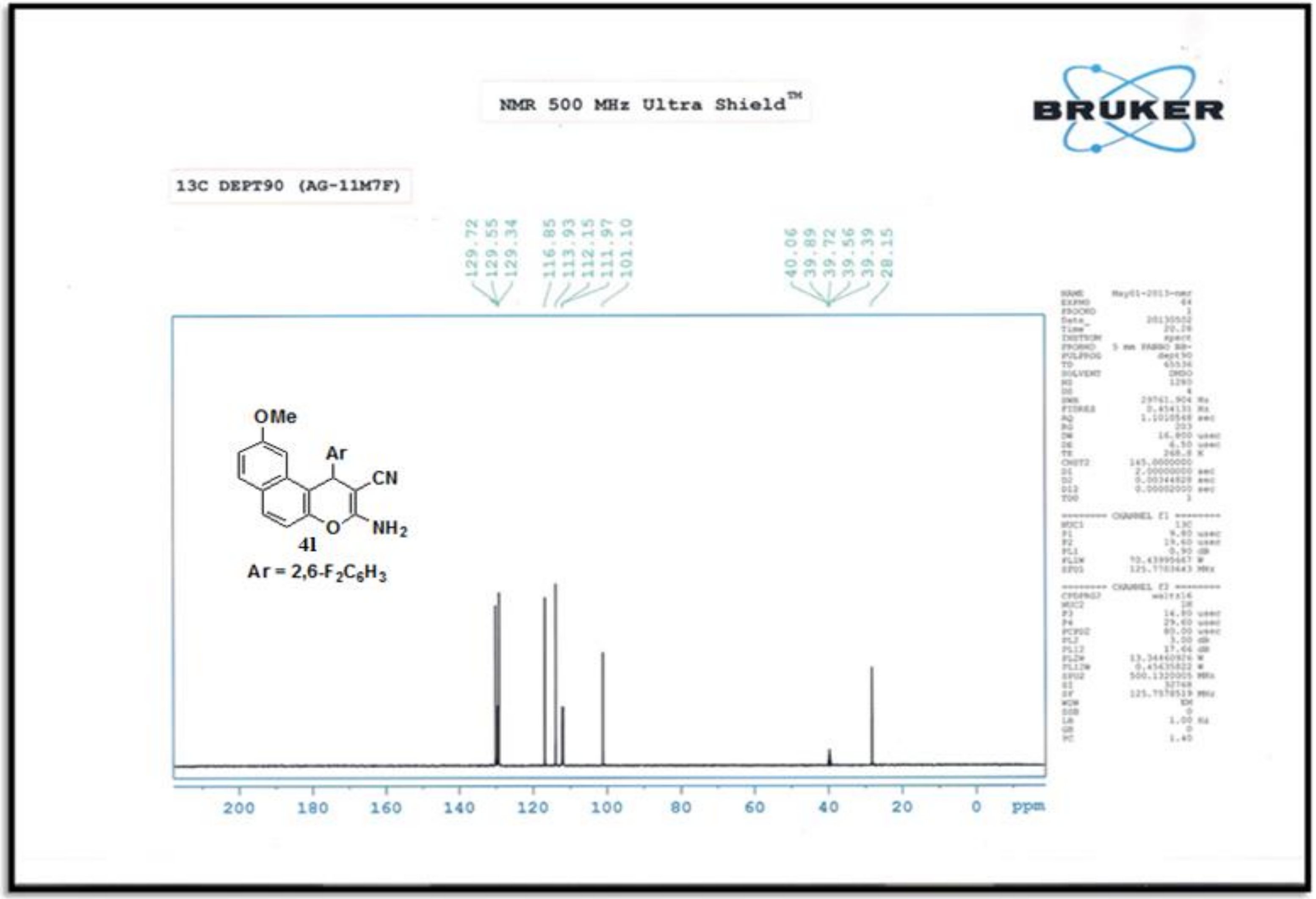

Figure S54: DEPT90 spectrum (DMSO-d_6, 125 MHz) of compound **4l.**

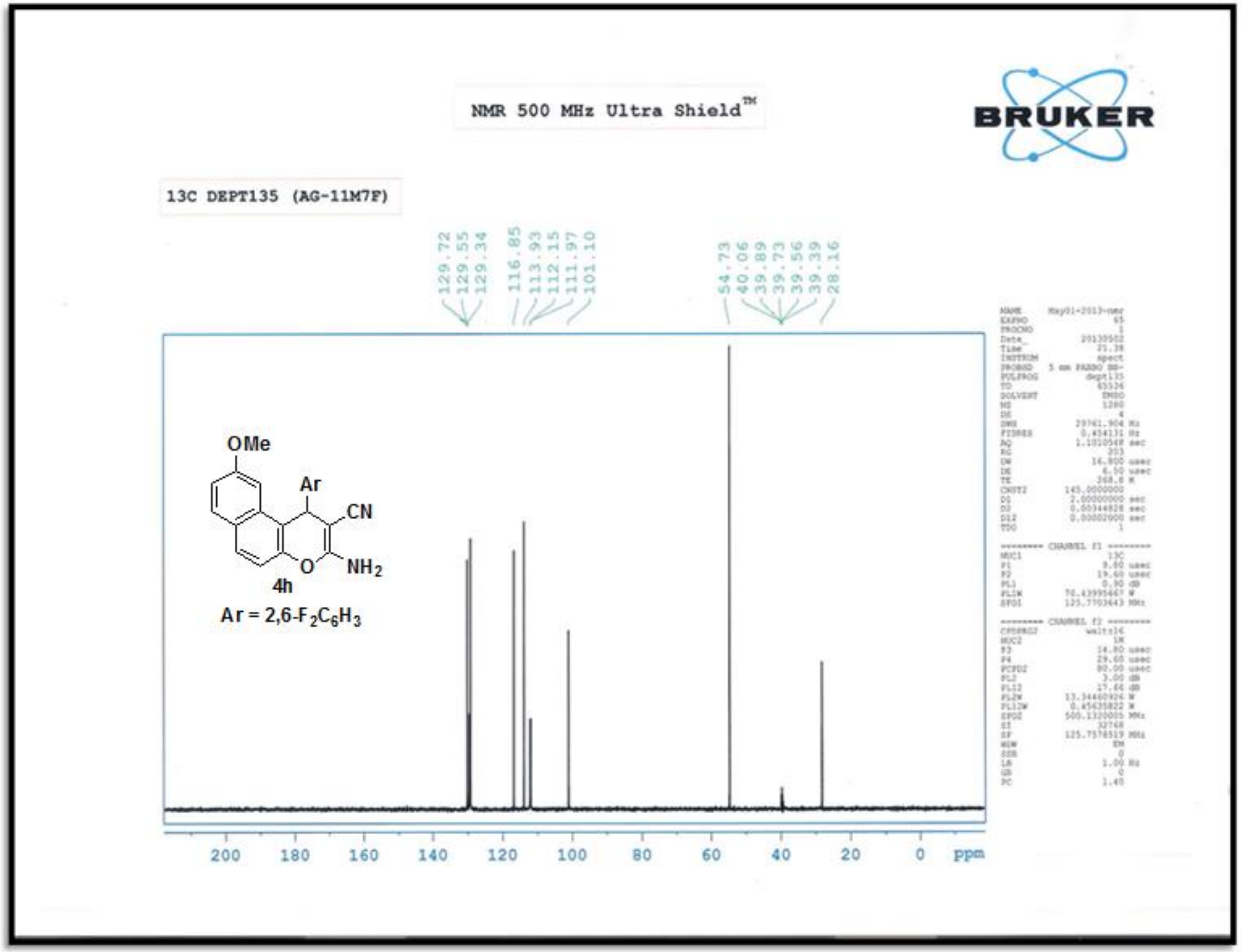

Figure S55: DEPT135 spectrum (DMSO-d_6, 125 MHz) of compound **4l.**

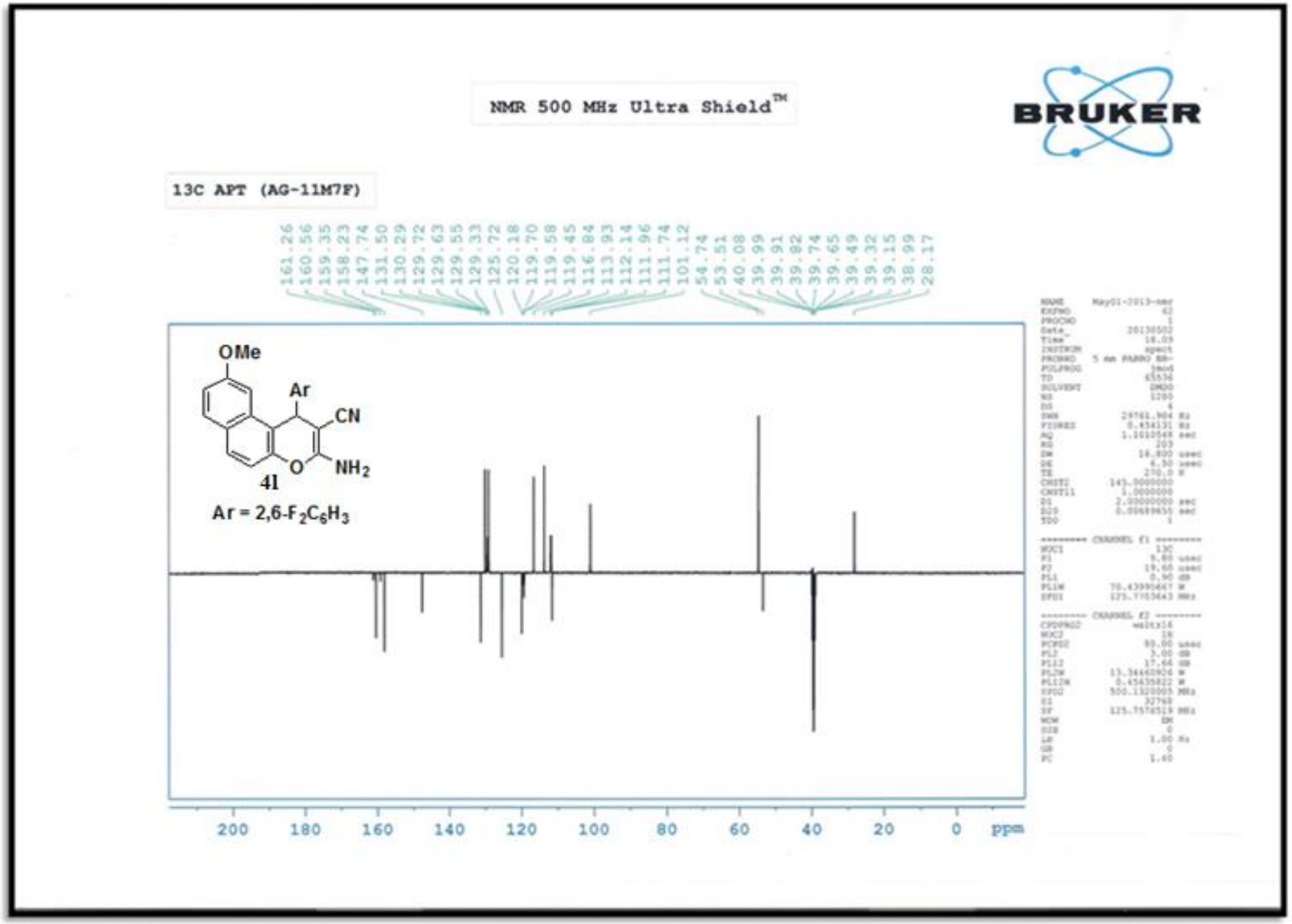

Figure S56: APT spectrum (DMSO-d_6, 125 MHz) of compound **4l**.

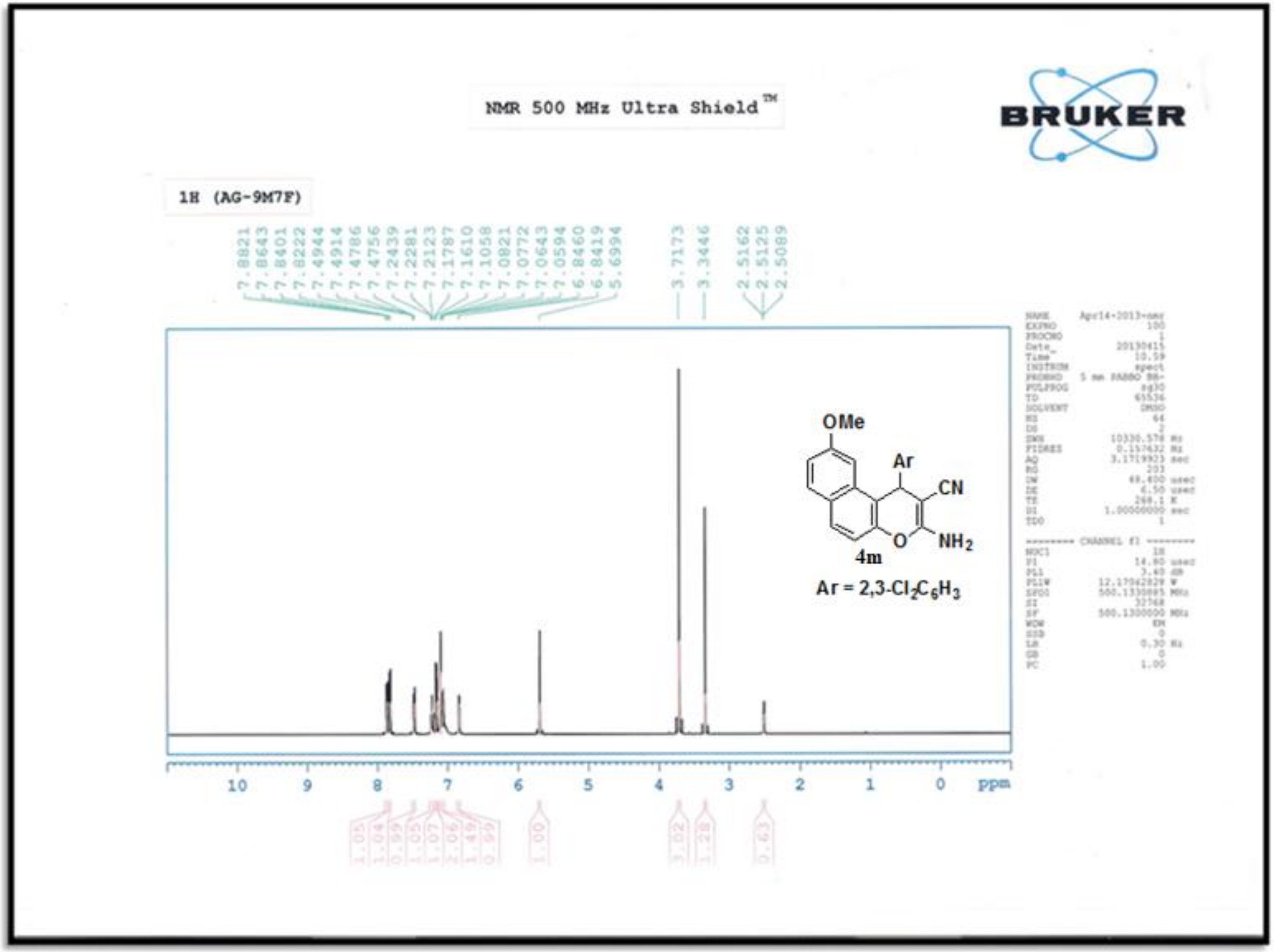

Figure S57: ^{1}H NMR spectrum (DMSO-d_6, 500 MHz) of compound **4m**.

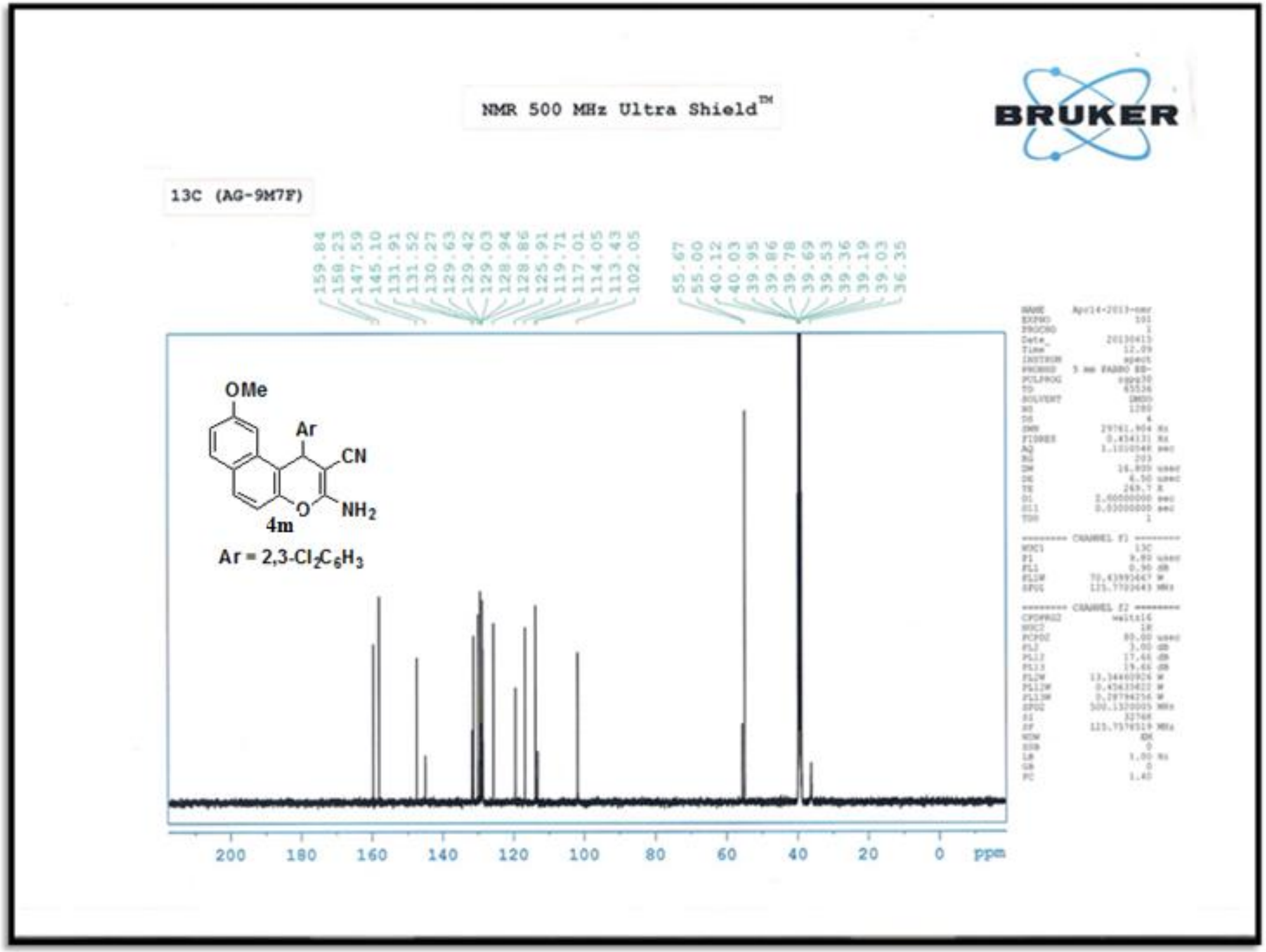

Figure S58: ^{13}C NMR spectrum (DMSO-d_6, 125 MHz) of compound **4m**.

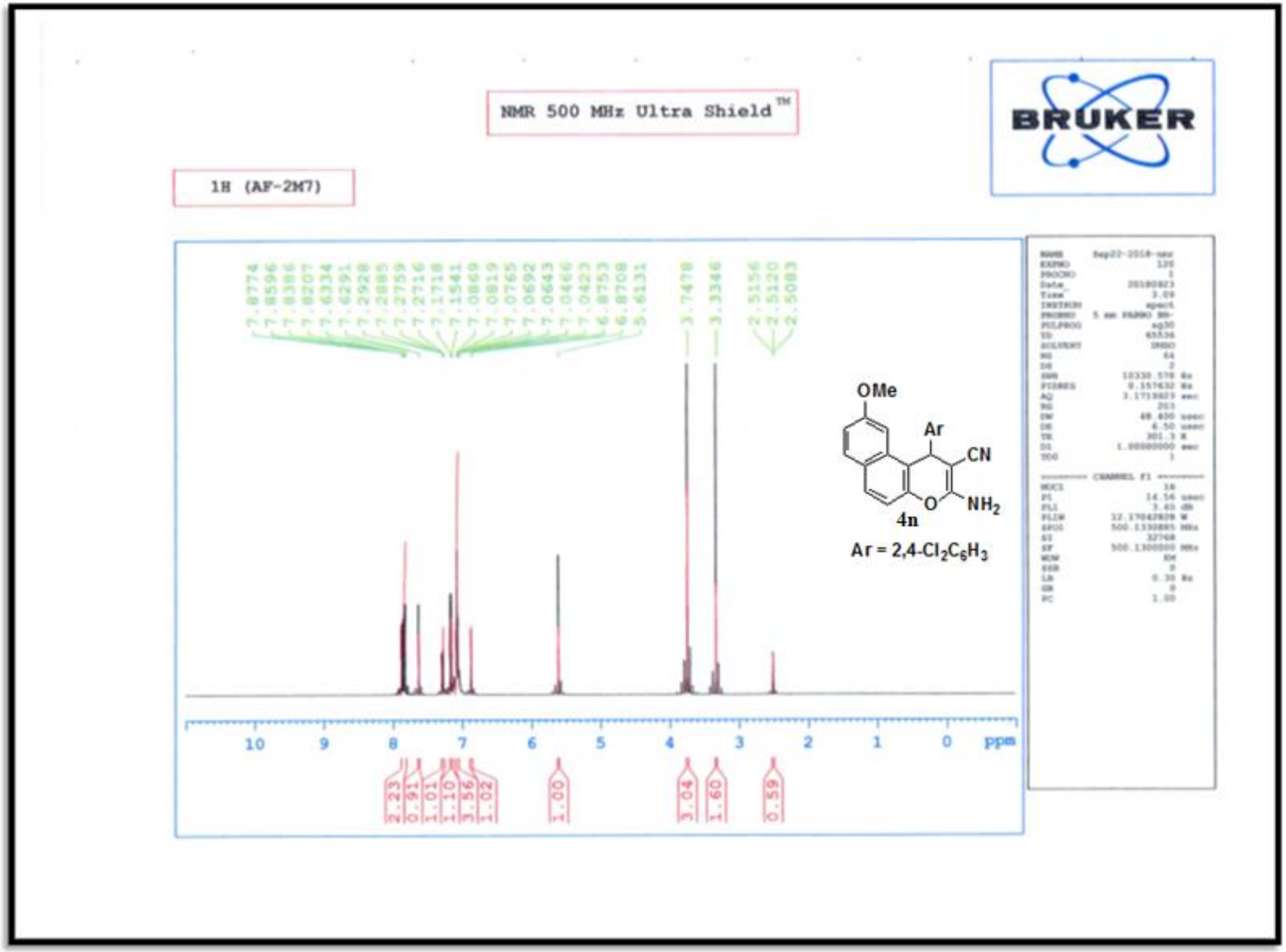

Figure S59: ^{1}H NMR spectrum (DMSO-d_6, 125 MHz) of compound **4n.**

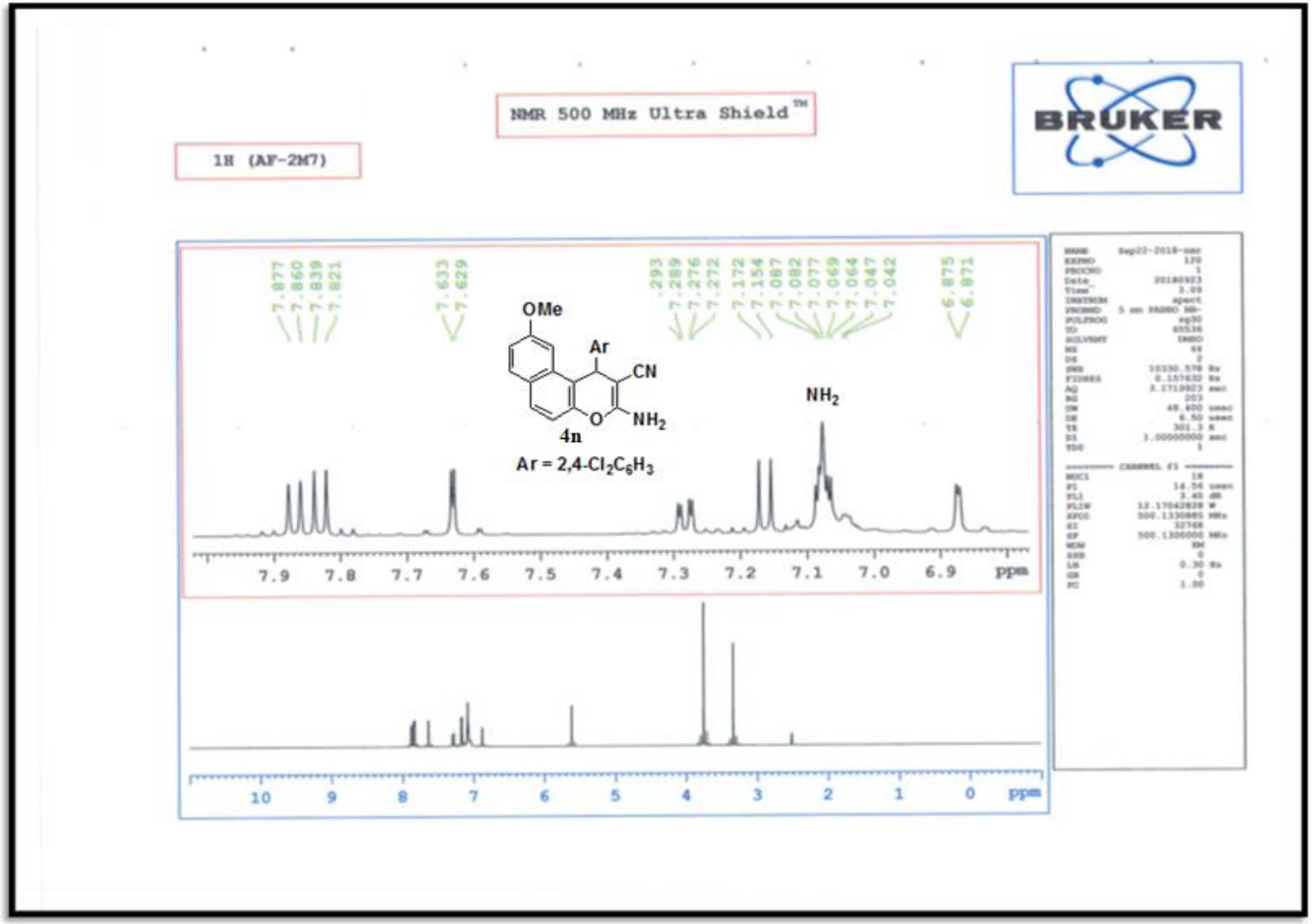

Figure S60: ^{1}H NMR spectrum (DMSO-d_6, 125 MHz) of compound **4n**.

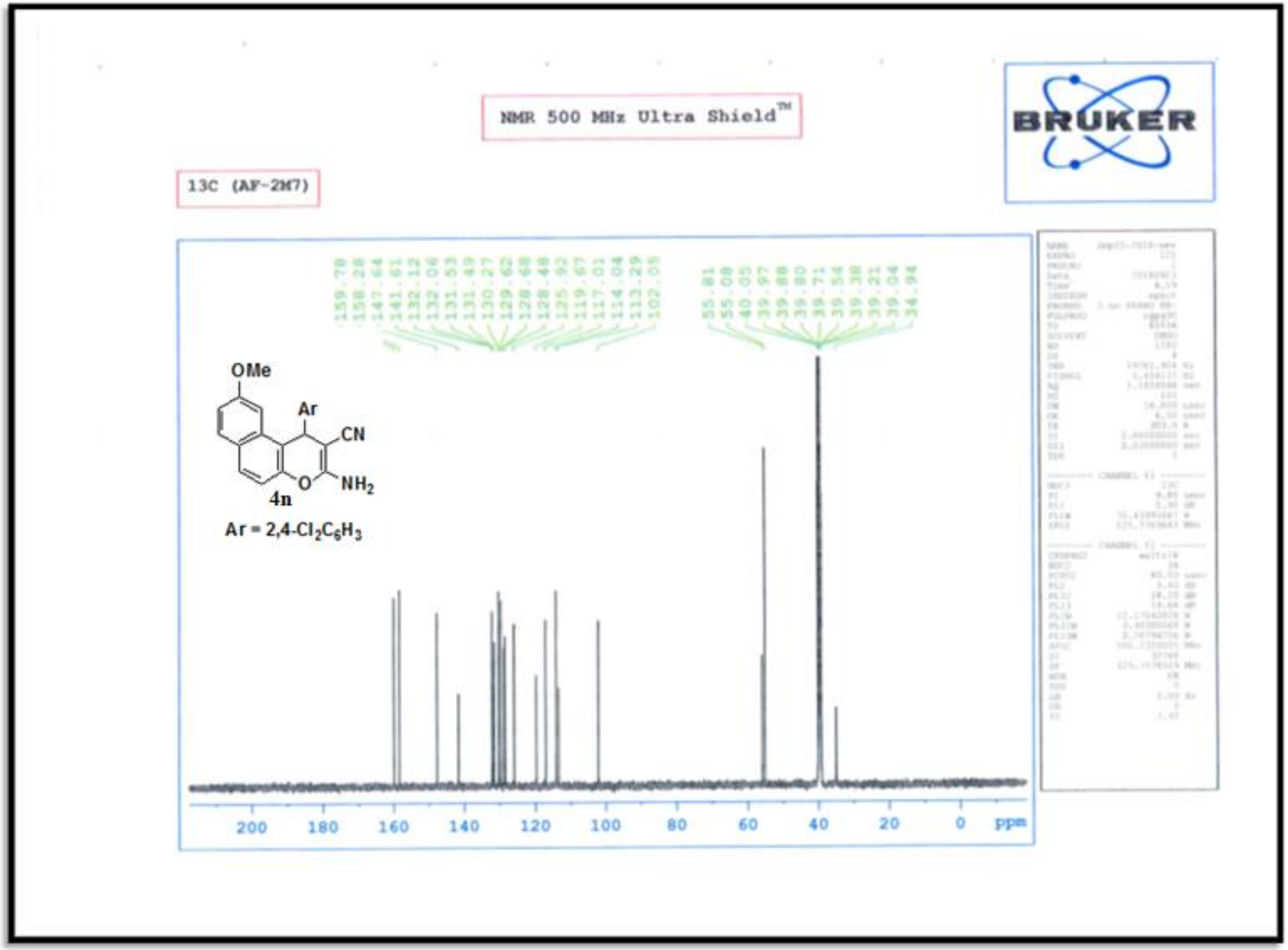

Figure S61: ^{13}C NMR spectrum (DMSO-d_6, 125 MHz) of compound **4n**.

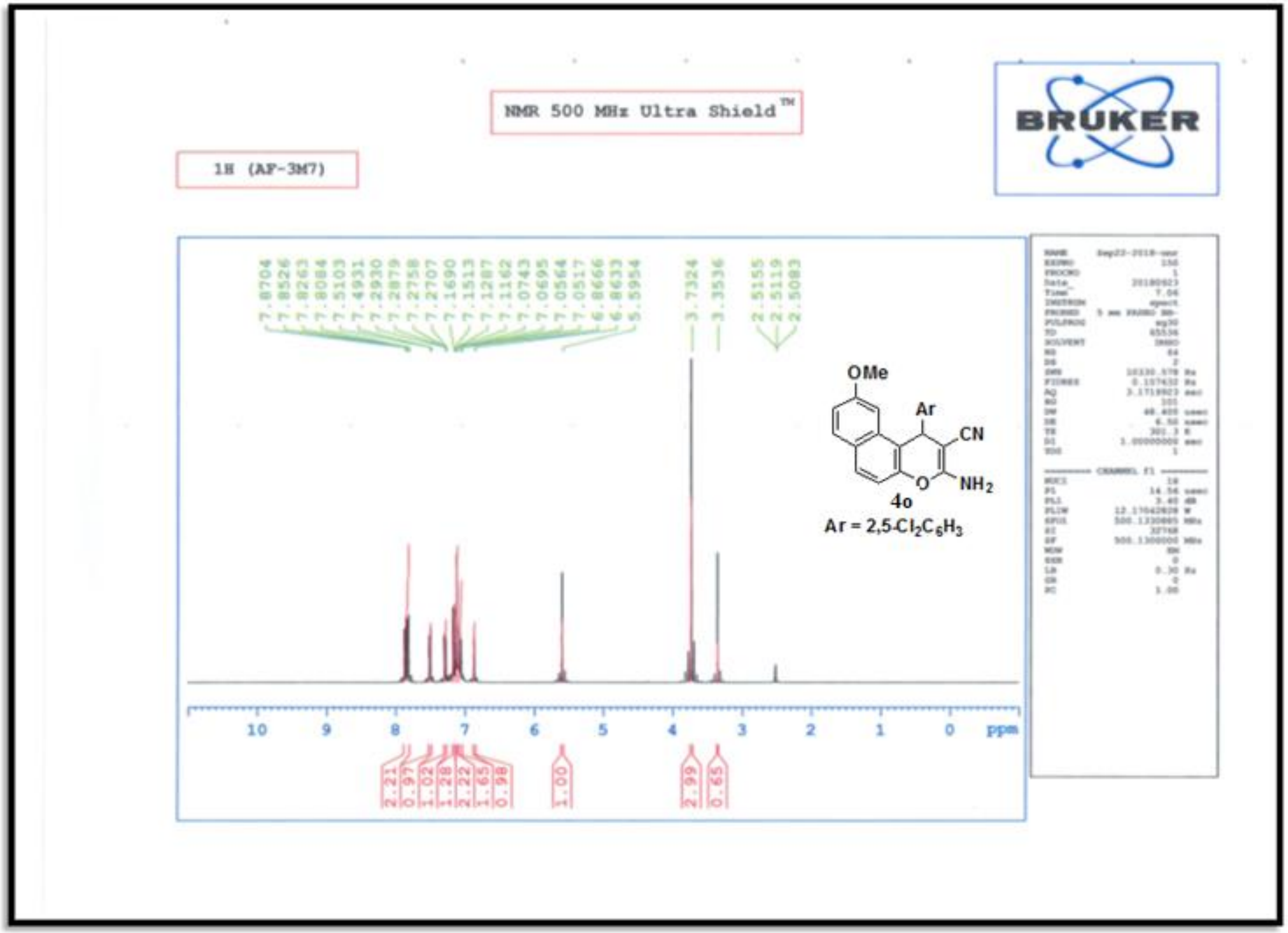

Figure S62: ^{1}H NMR spectrum (DMSO-d_6, 500 MHz) of compound **4o**.

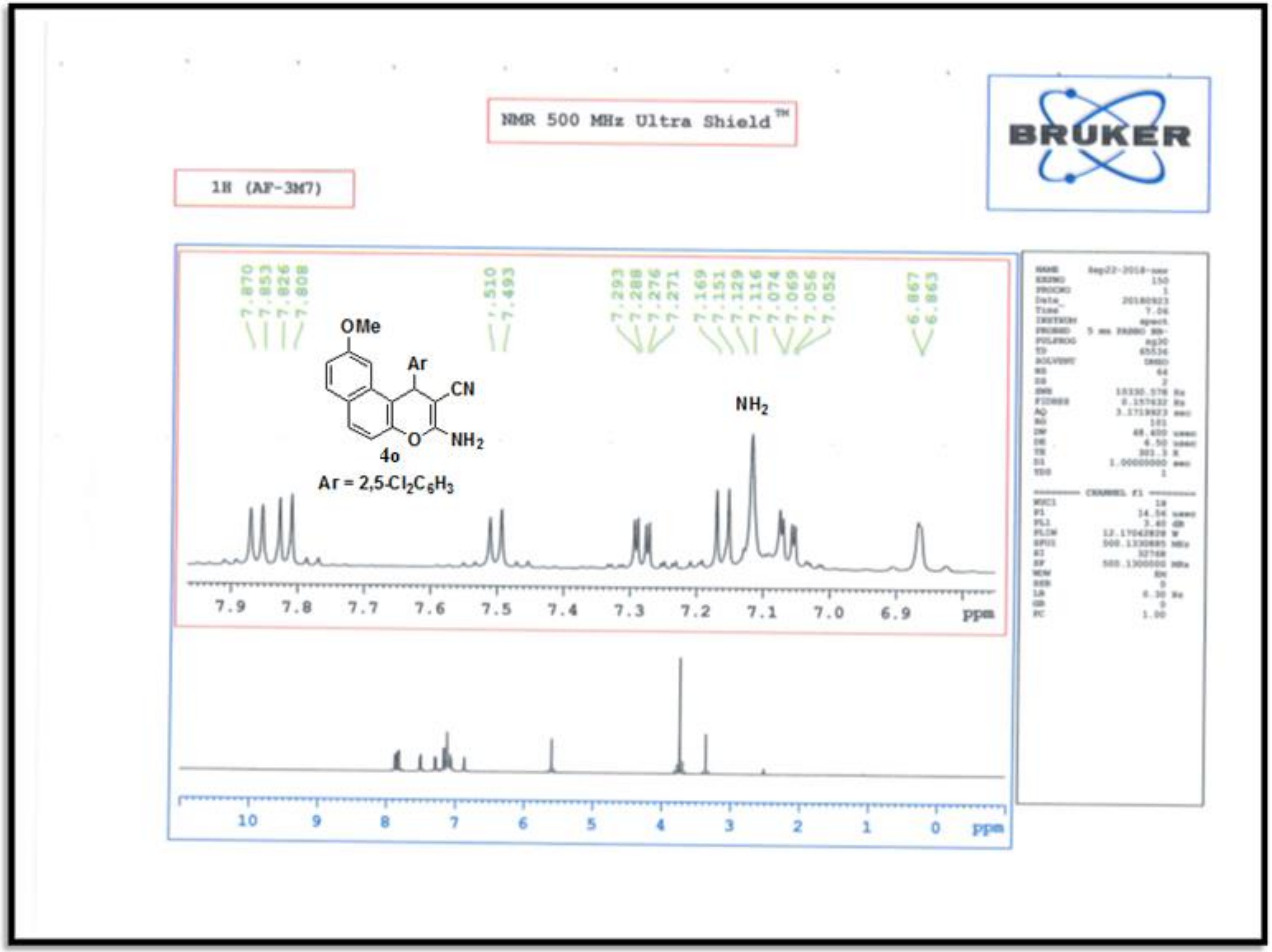

Figure S63: ^{1}H NMR spectrum (DMSO-d_6, 500 MHz) of compound **4o**.

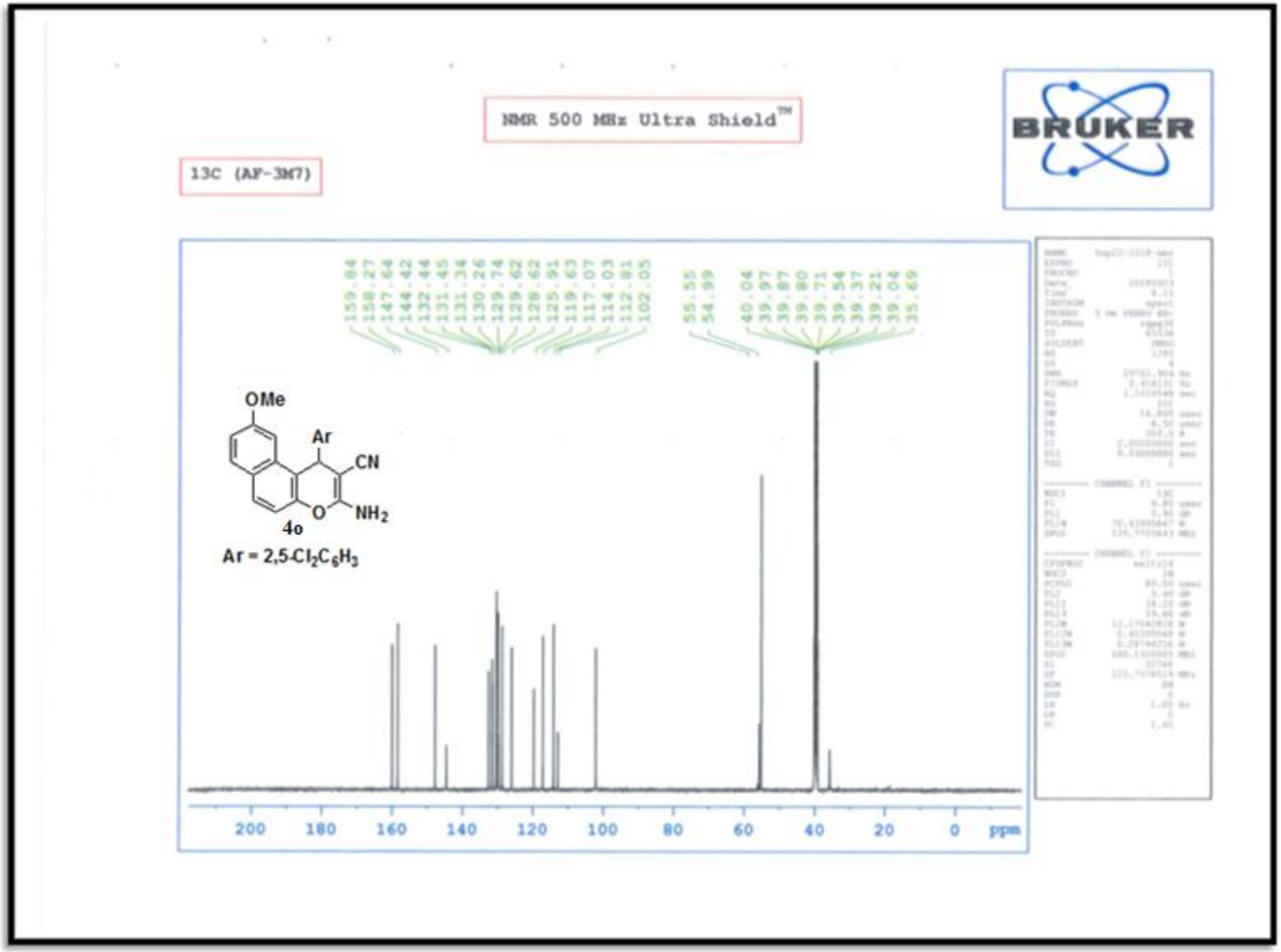

Figure S64: ^{13}C NMR spectrum (DMSO-d_6, 125 MHz) of compound **4o**.

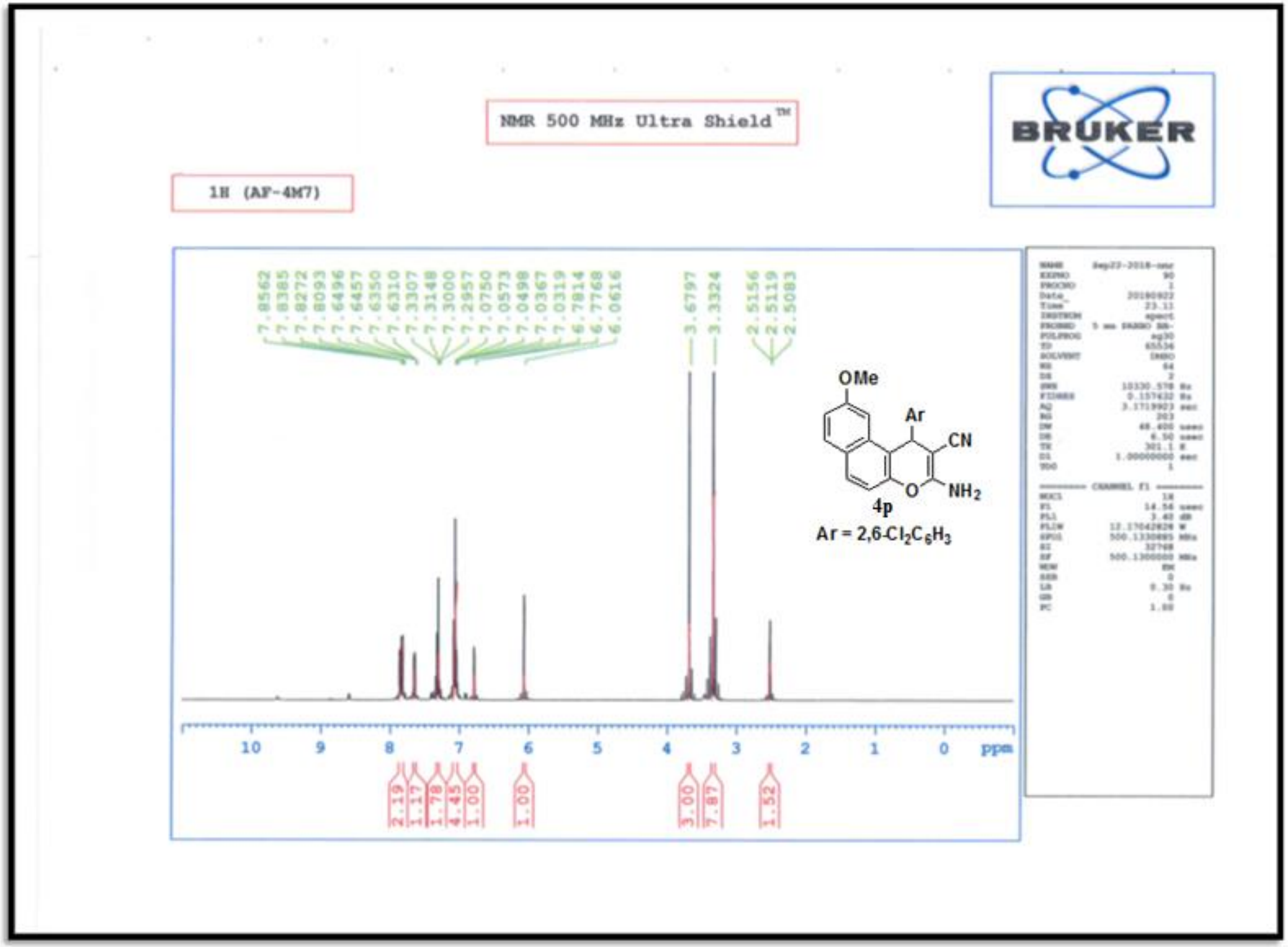

Figure S65: ^{1}H NMR spectrum (DMSO-d_6, 500 MHz) of compound **4p**.

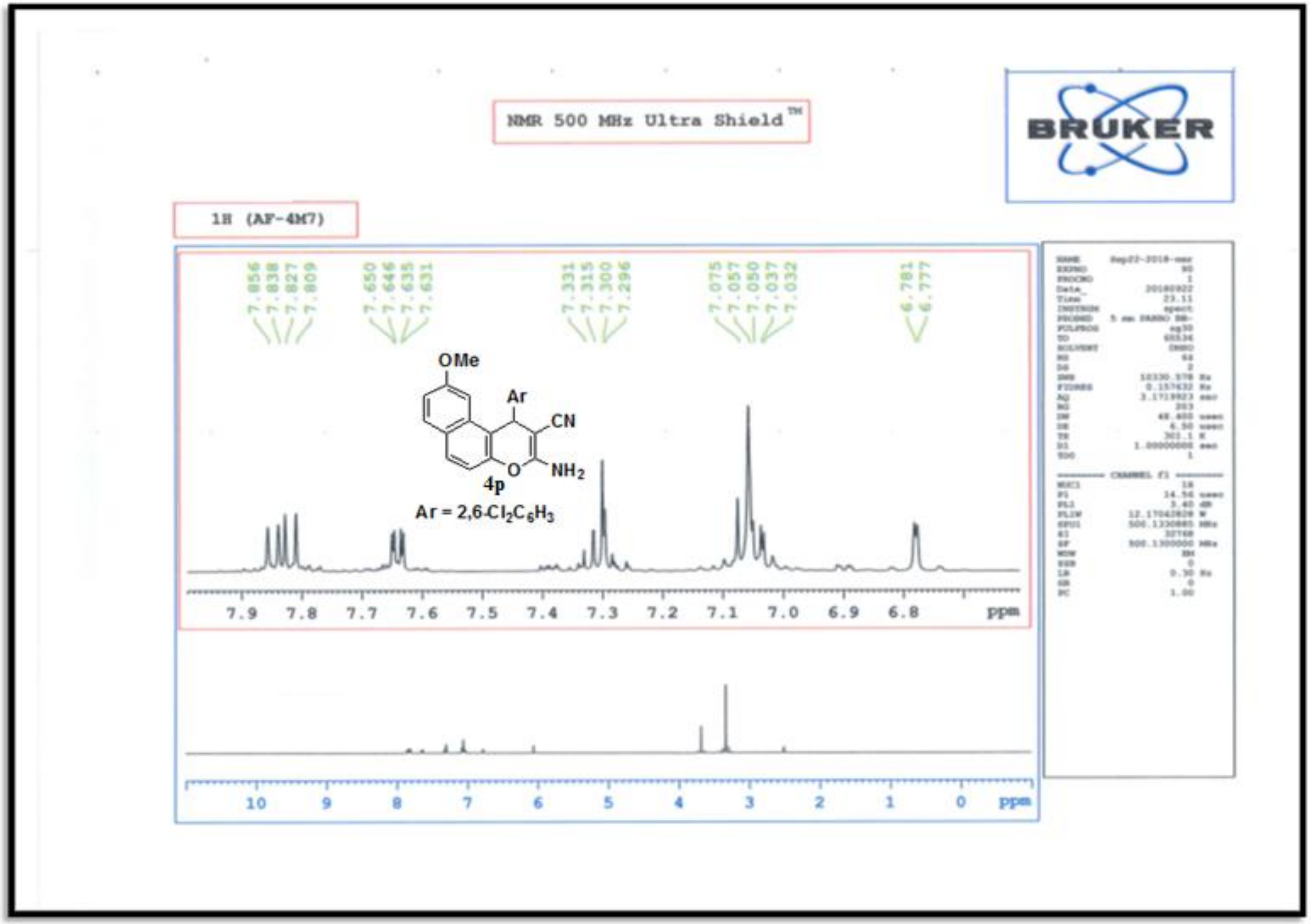

Figure S66: ^{1}H NMR spectrum (DMSO-d_6, 500 MHz) of compound **4p**.

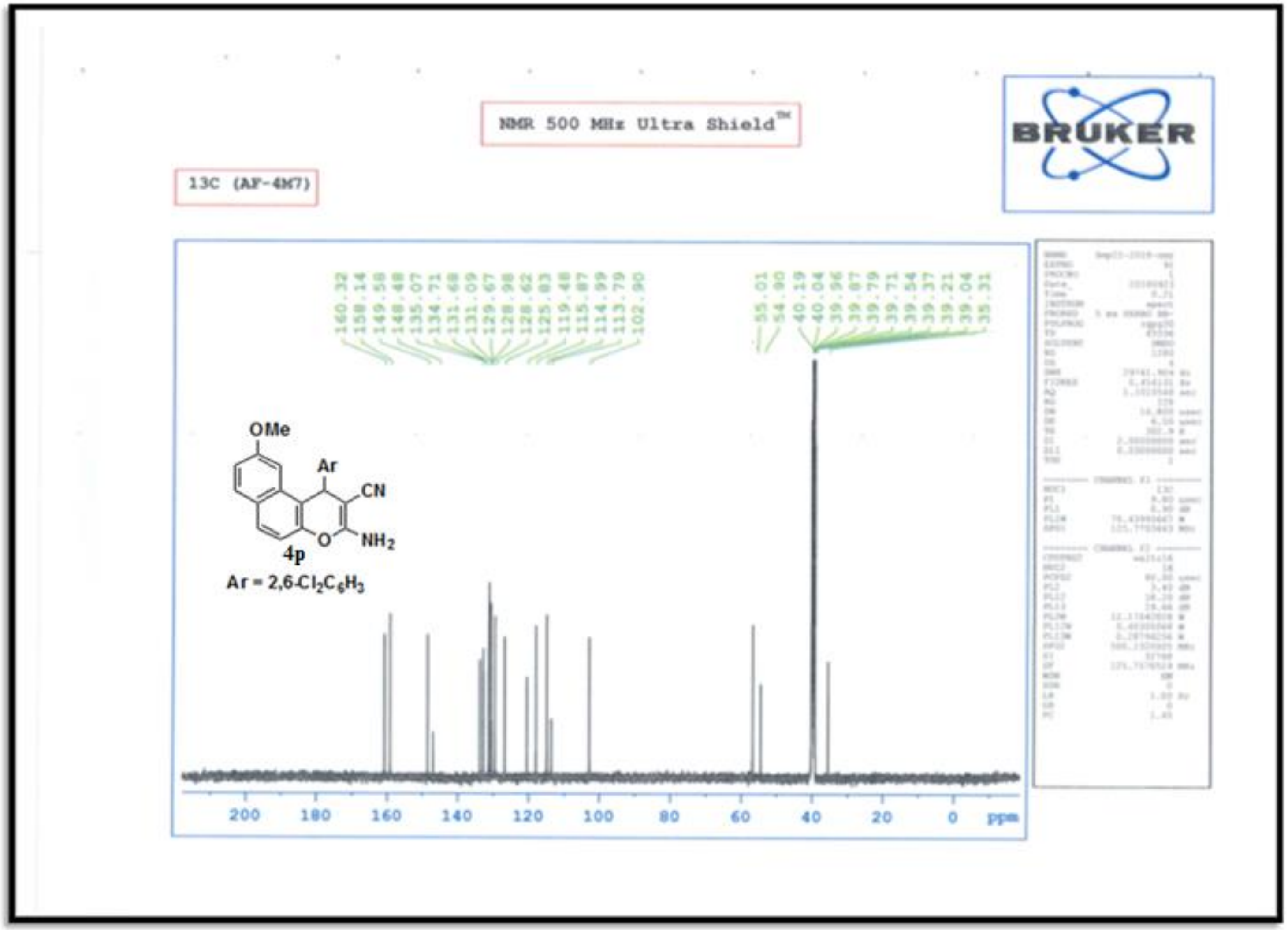

Figure S67: ^{13}C NMR spectrum (DMSO-d_6, 125 MHz) of compound **4p**.

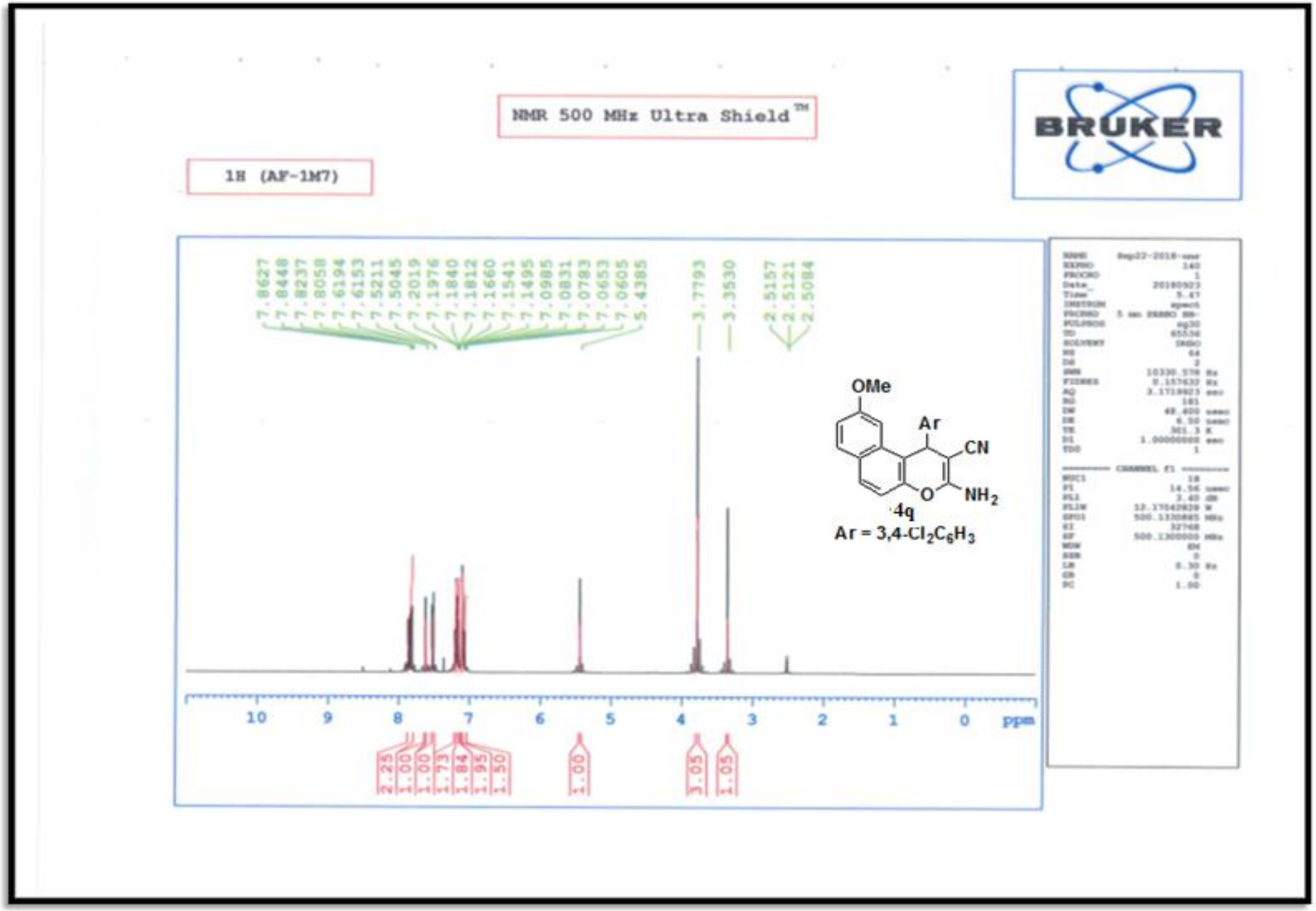

Figure S68: ^{1}H NMR spectrum (DMSO-d_6, 500 MHz) of compound **4q**.

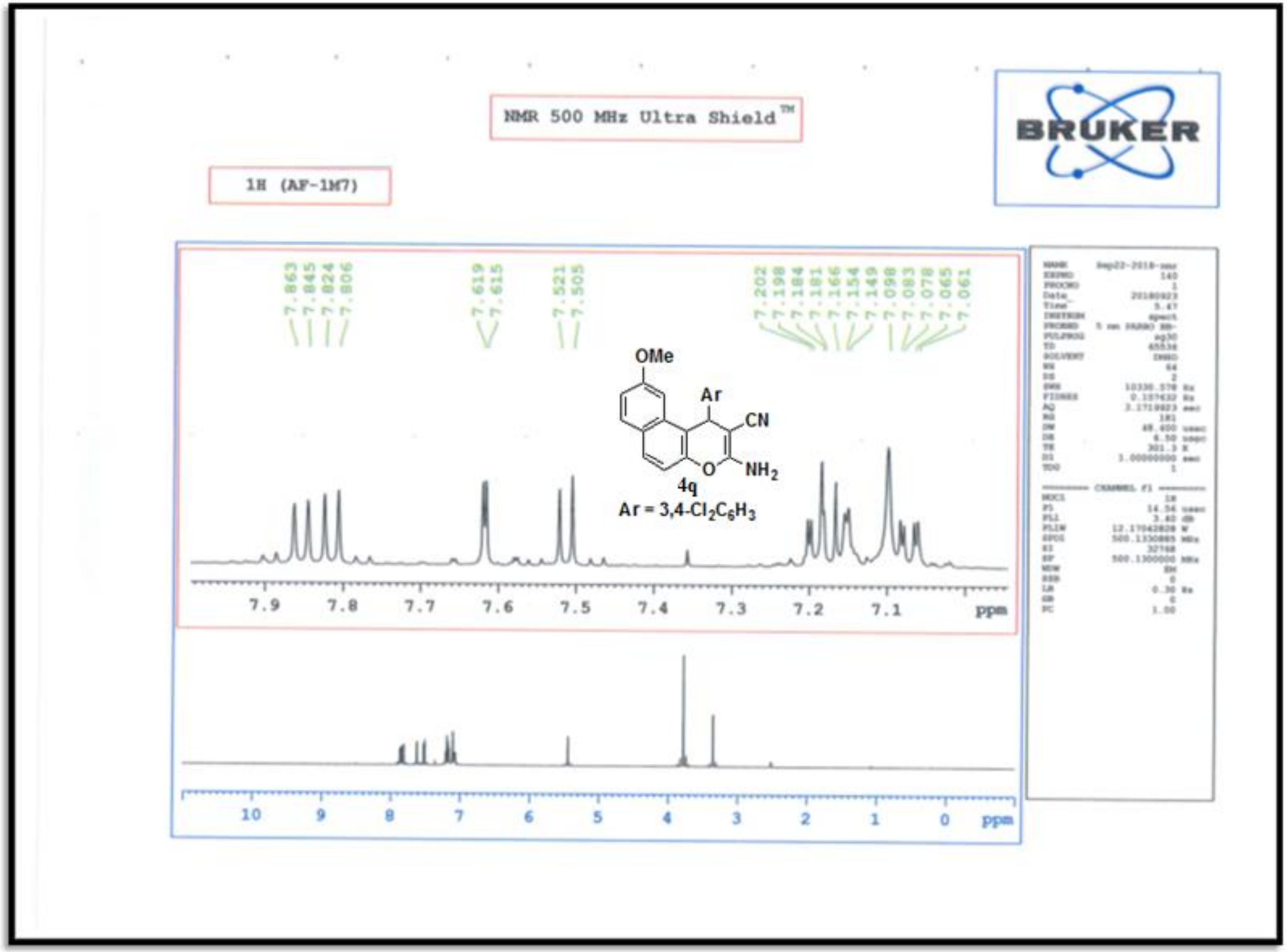

Figure S69: ^{1}H NMR spectrum (DMSO-d_6, 500 MHz) of compound **4q**.

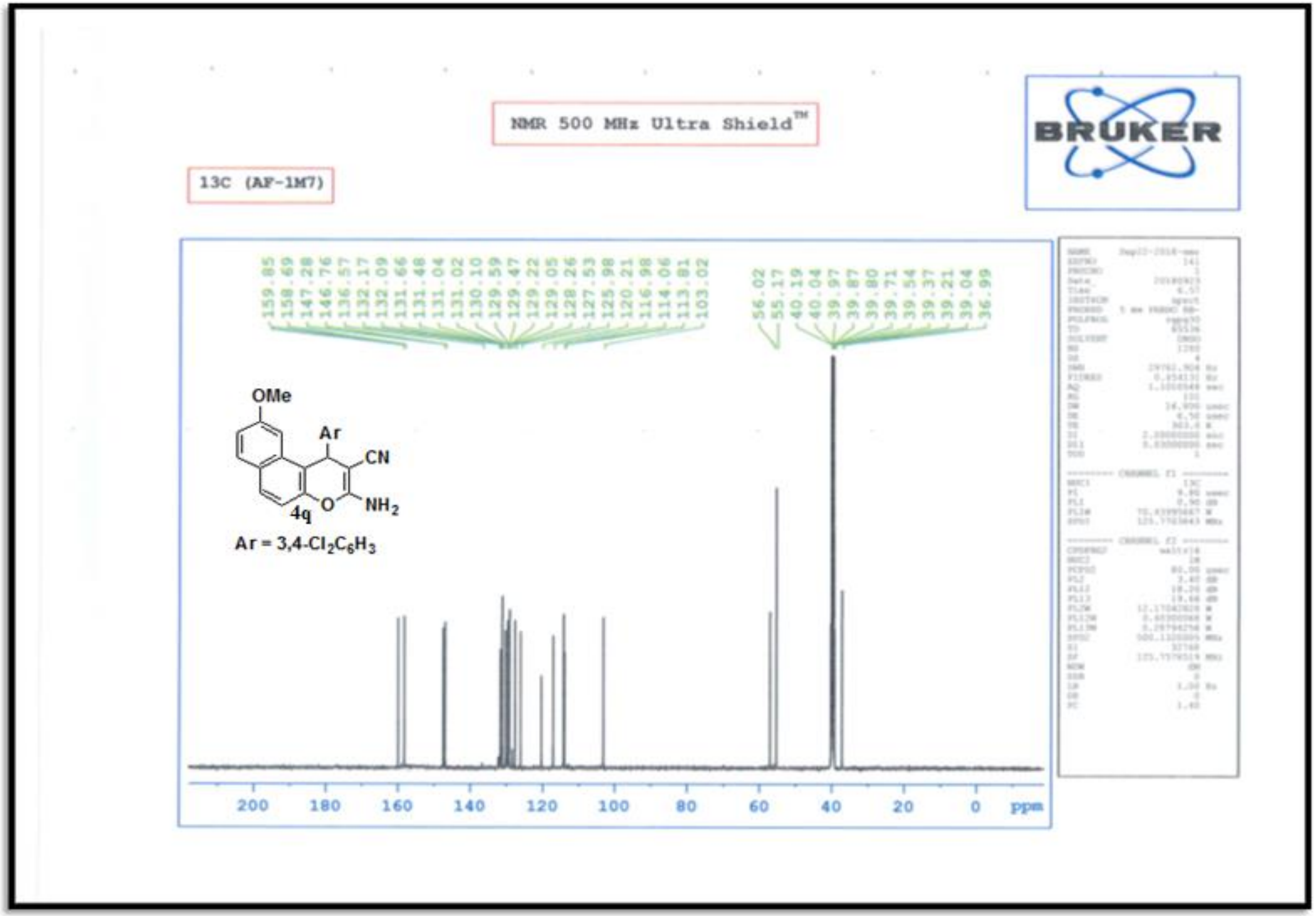

Figure S70: ^{13}C NMR spectrum (DMSO-d_6, 125 MHz) of compound **4q**.

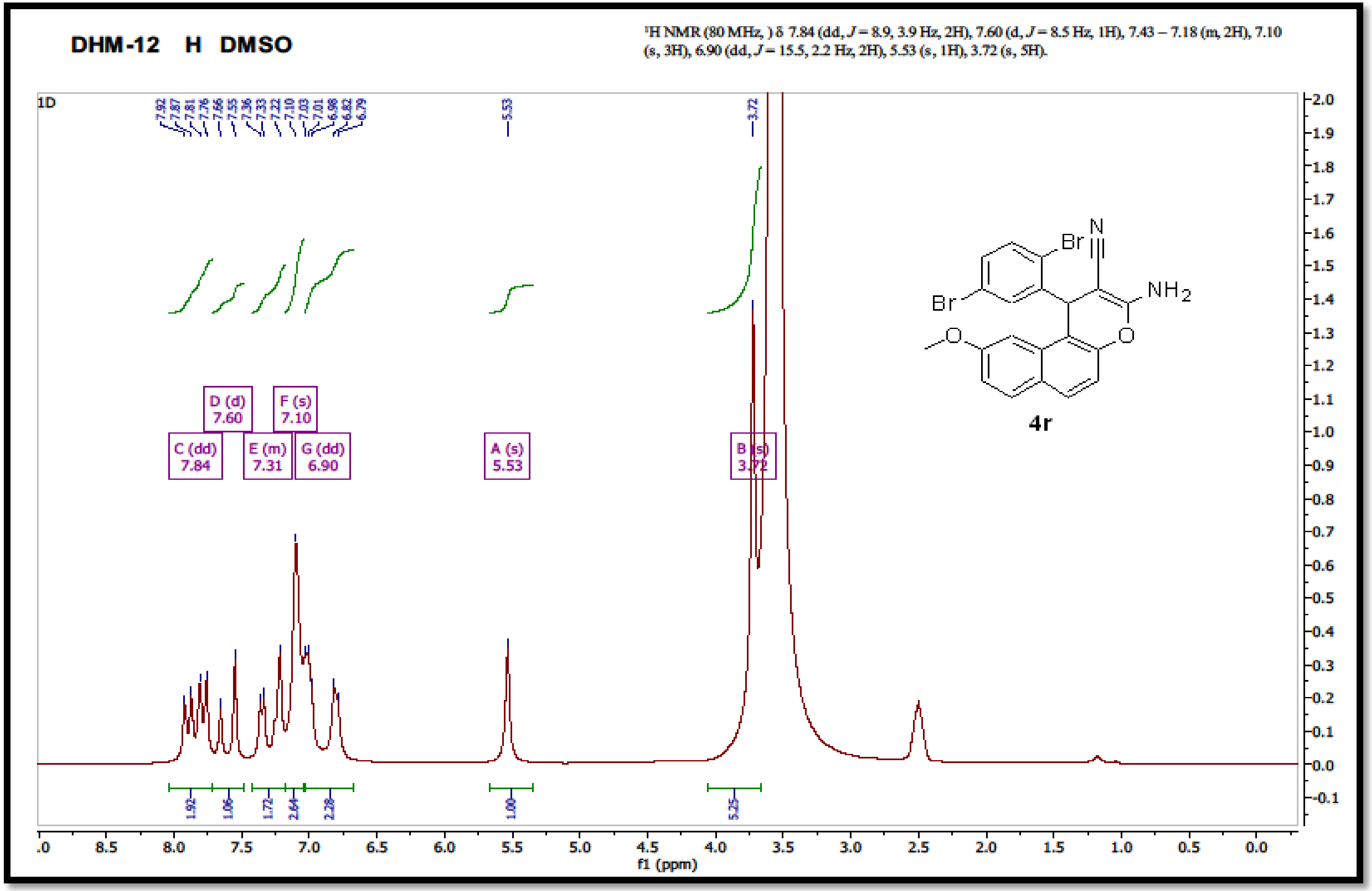

Figure S71: ^{1}H NMR spectrum (DMSO-d_6, 500 MHz) of compound **4r**.

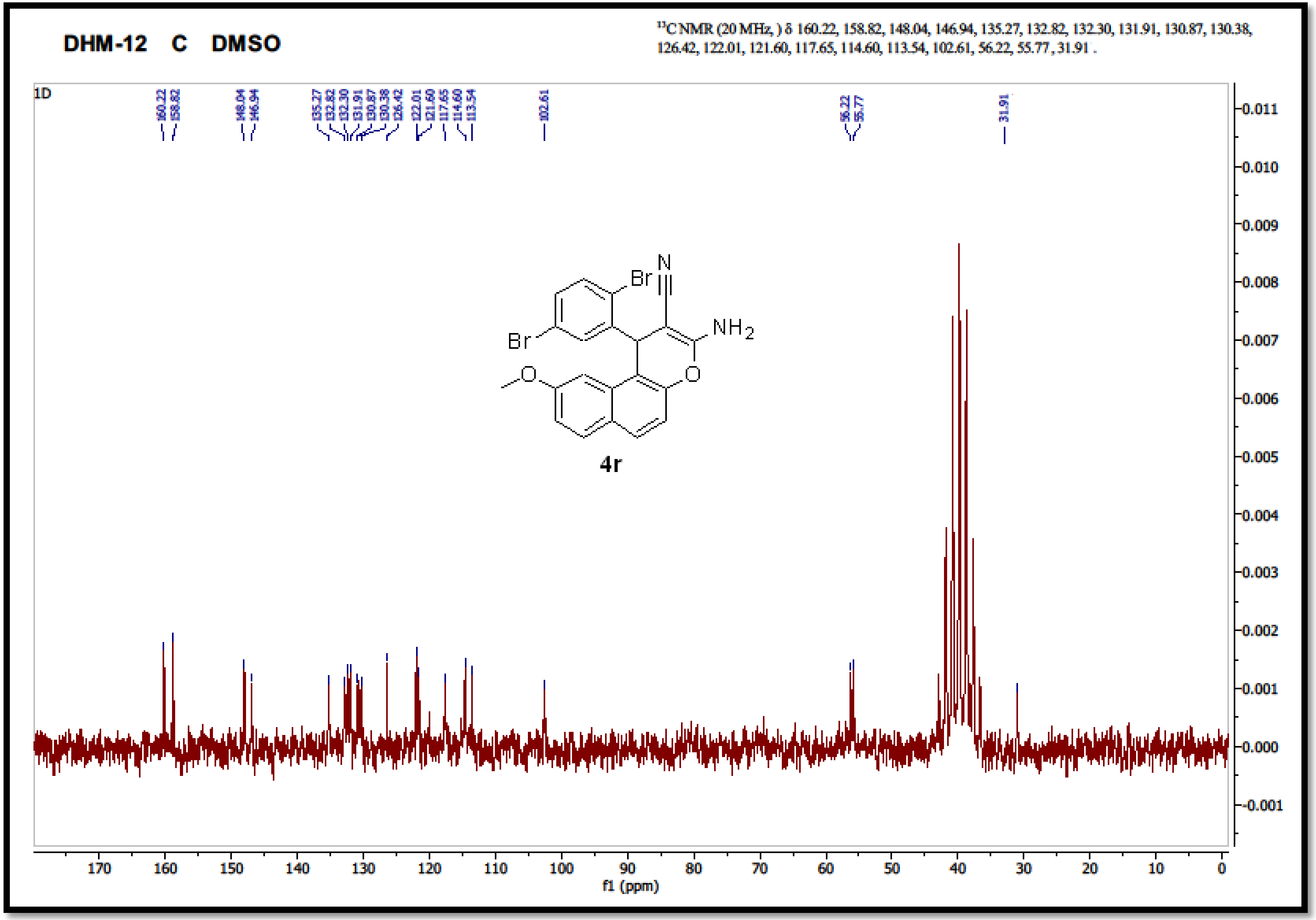

Figure S72: ^{13}C NMR spectrum (DMSO-d_6, 500 MHz) of compound **4r**.

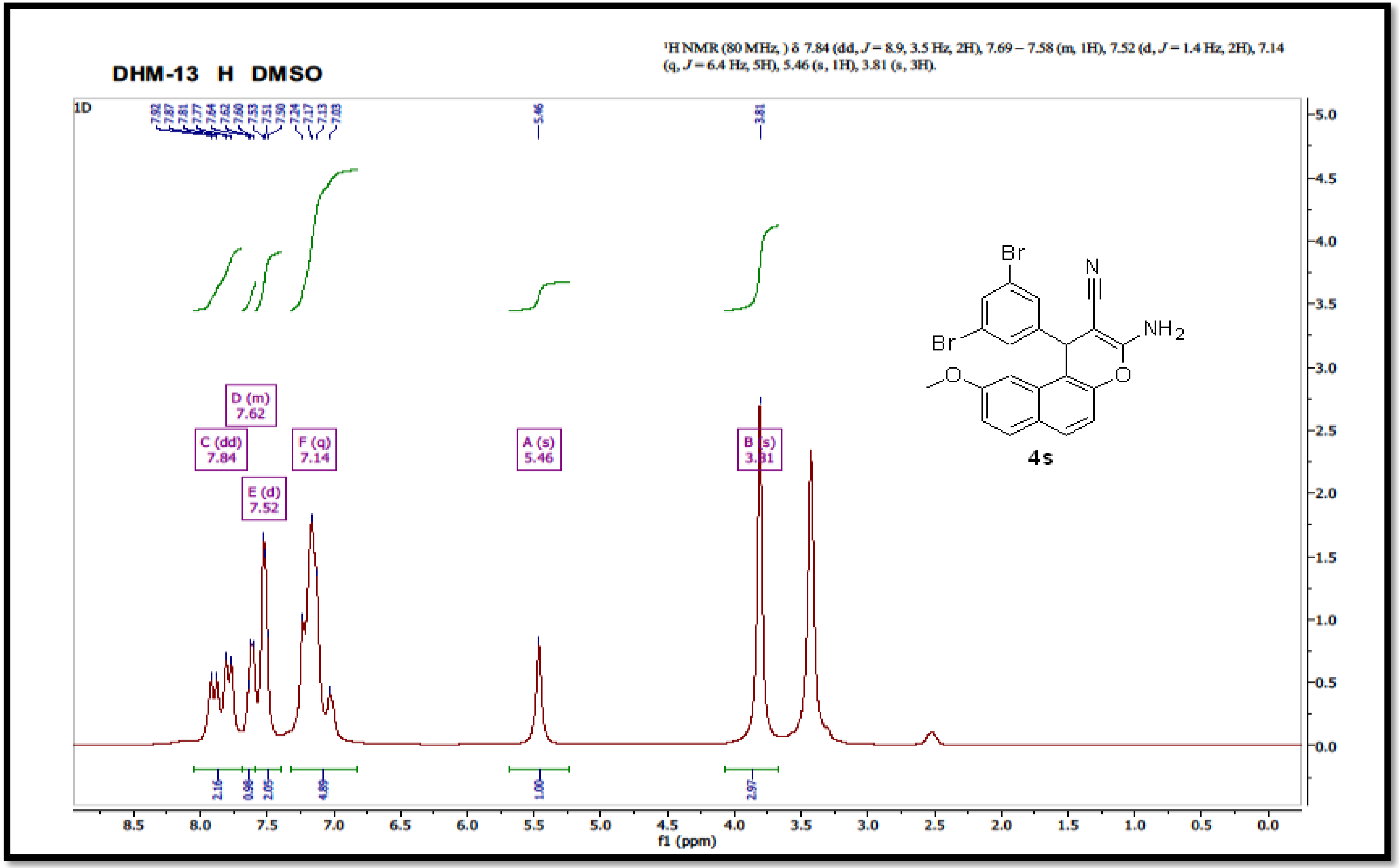

Figure S73: ^{1}H NMR spectrum (DMSO-d_6, 500 MHz) of compound **4s**.

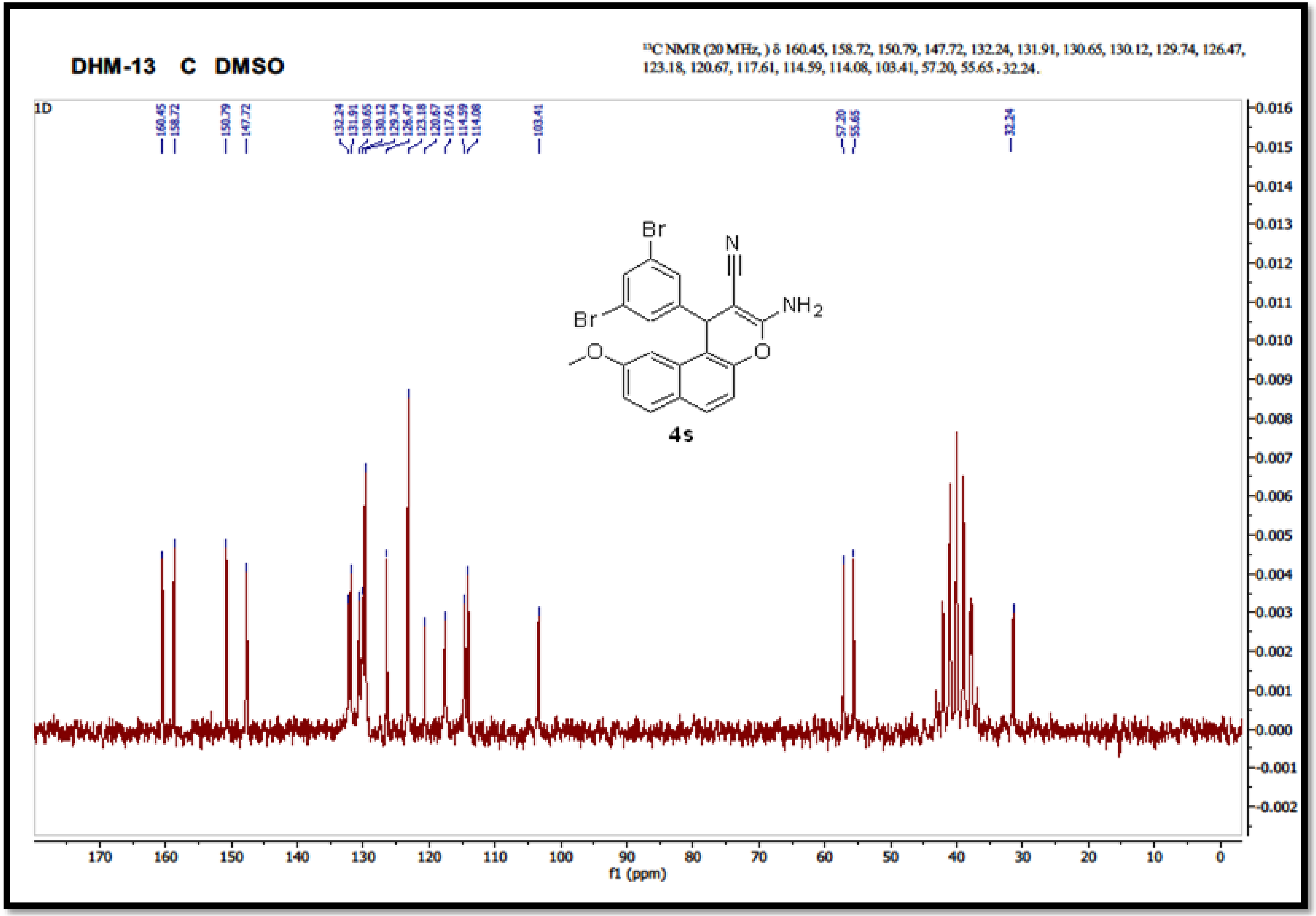

Figure S74: ^{13}C NMR spectrum (DMSO-d_6, 500 MHz) of compound **4s**.

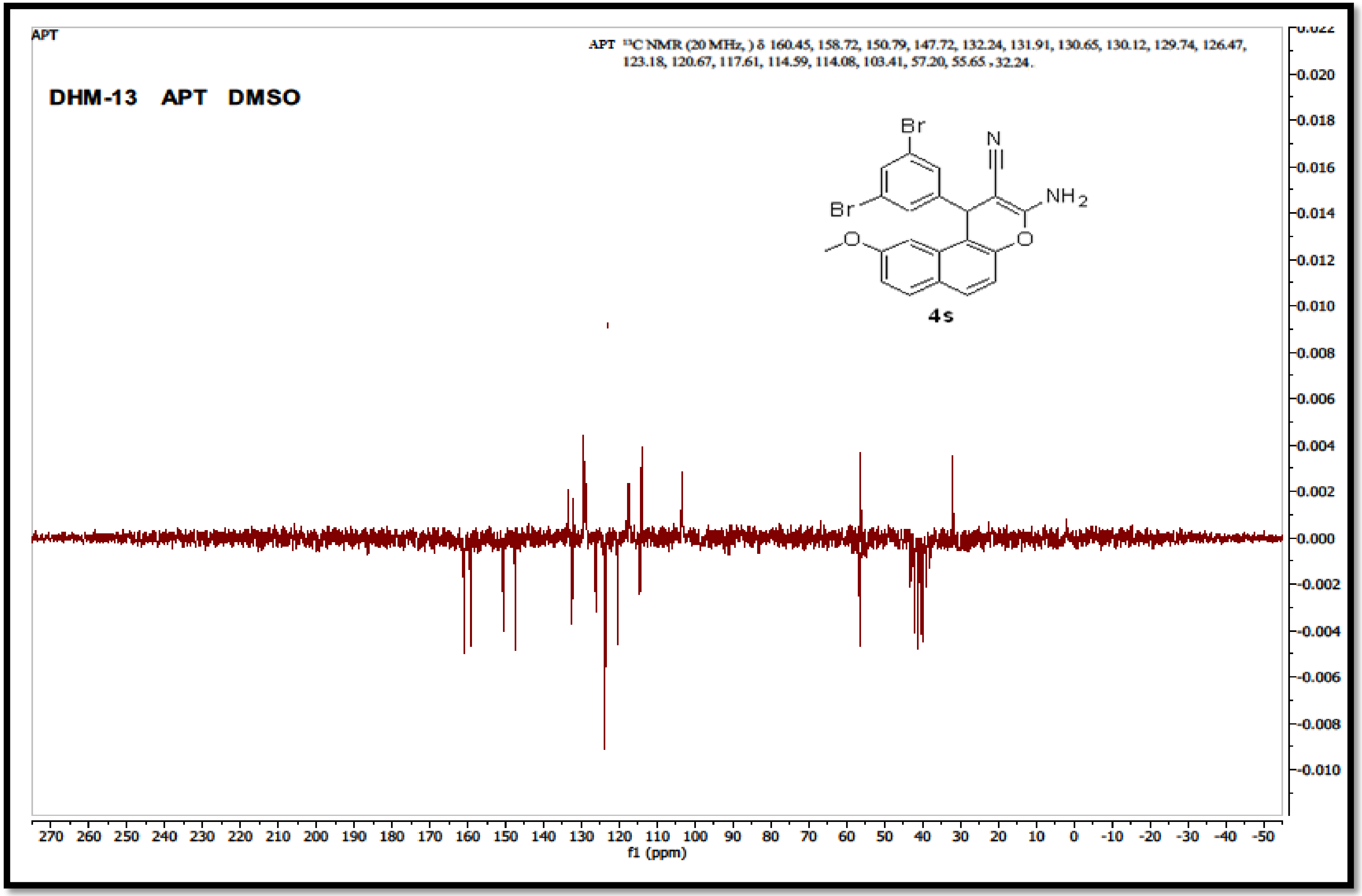

Figure S75: APT spectrum spectrum (DMSO-d_6, 500 MHz) of compound **4s**.

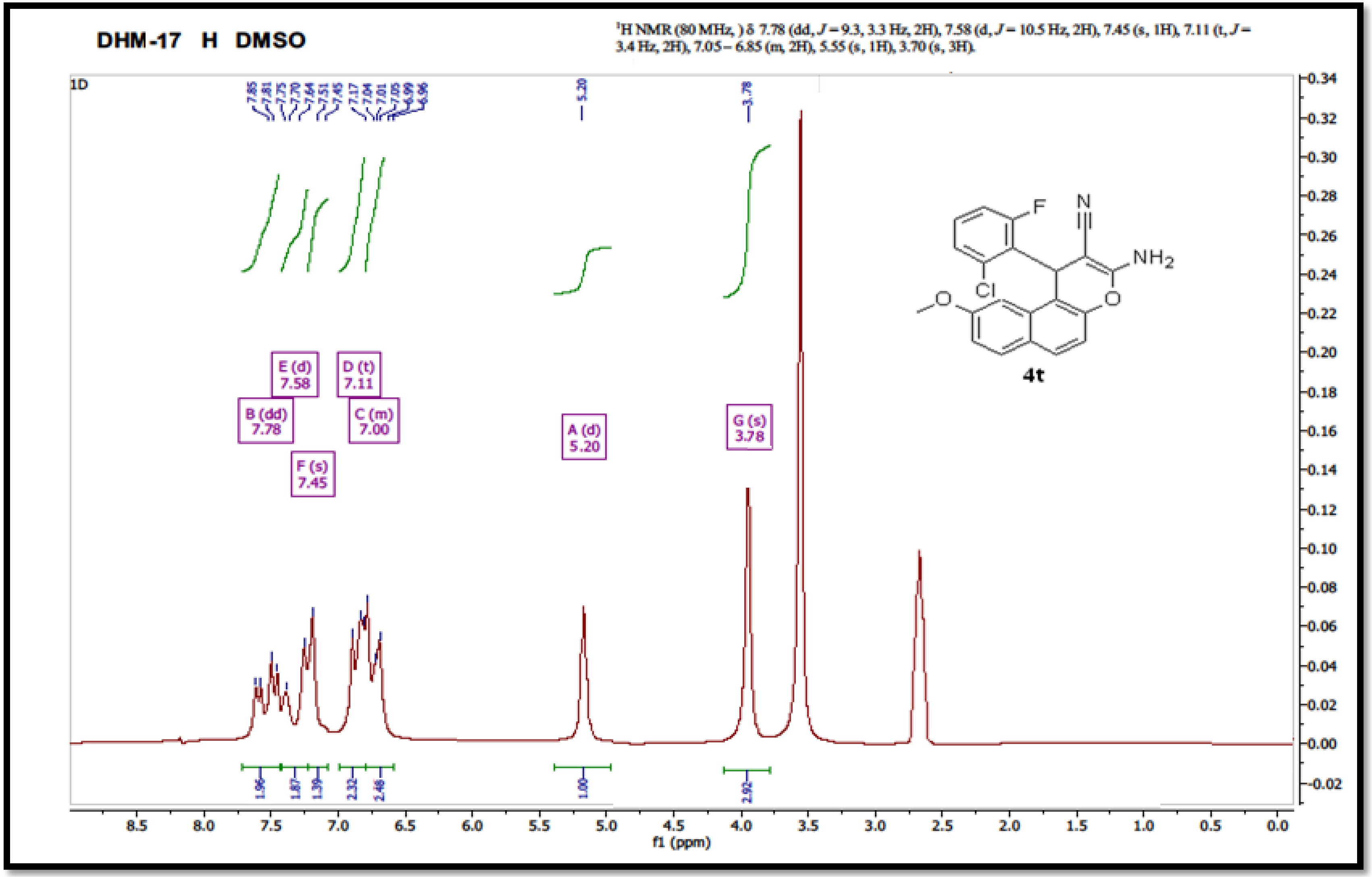

Figure S76: ^{1}H NMR spectrum (DMSO-d_6, 500 MHz) of compound **4t**.

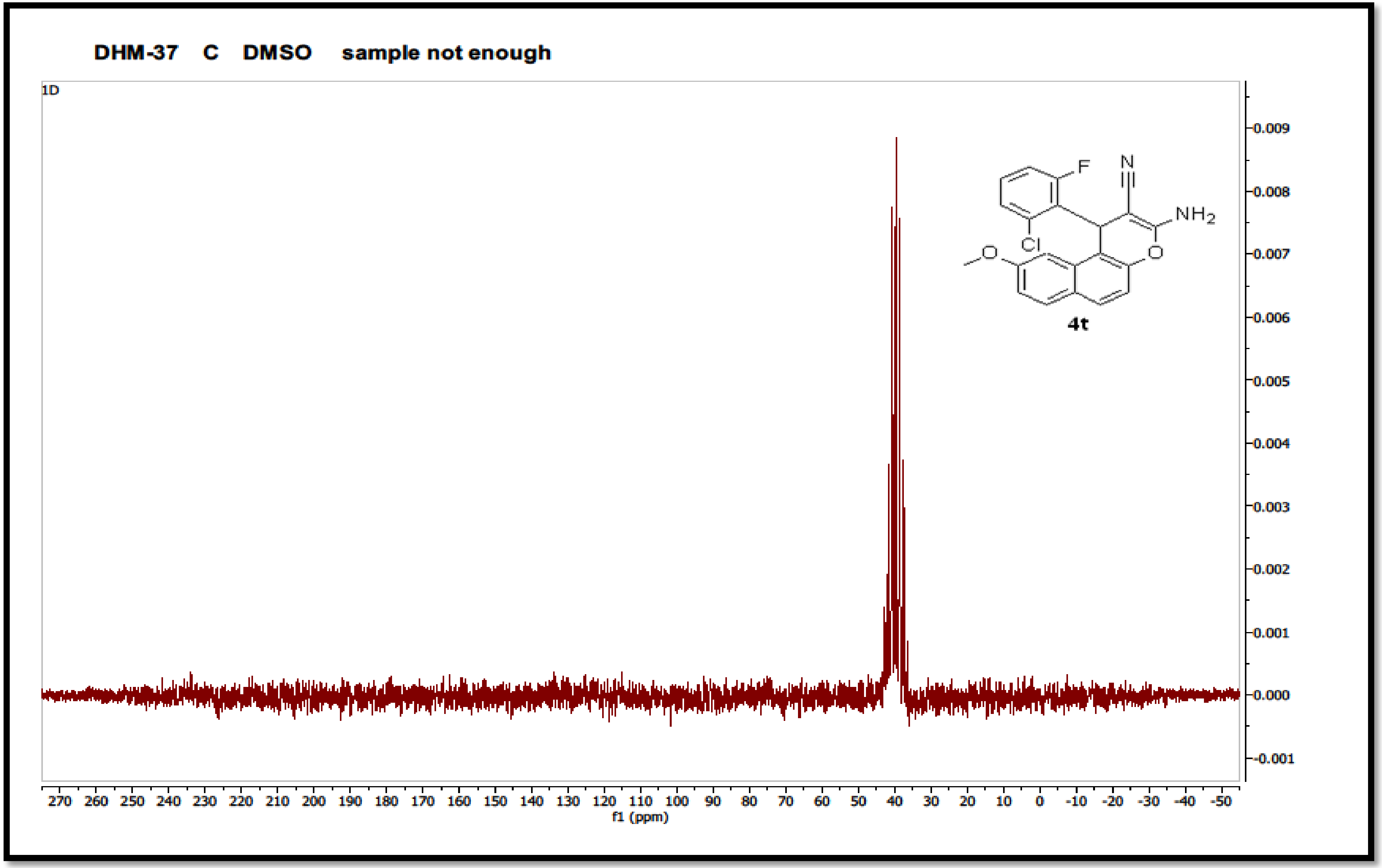

Figure S77: ^{13}C NMR spectrum (DMSO-d_6, 500 MHz) of compound **4t**.

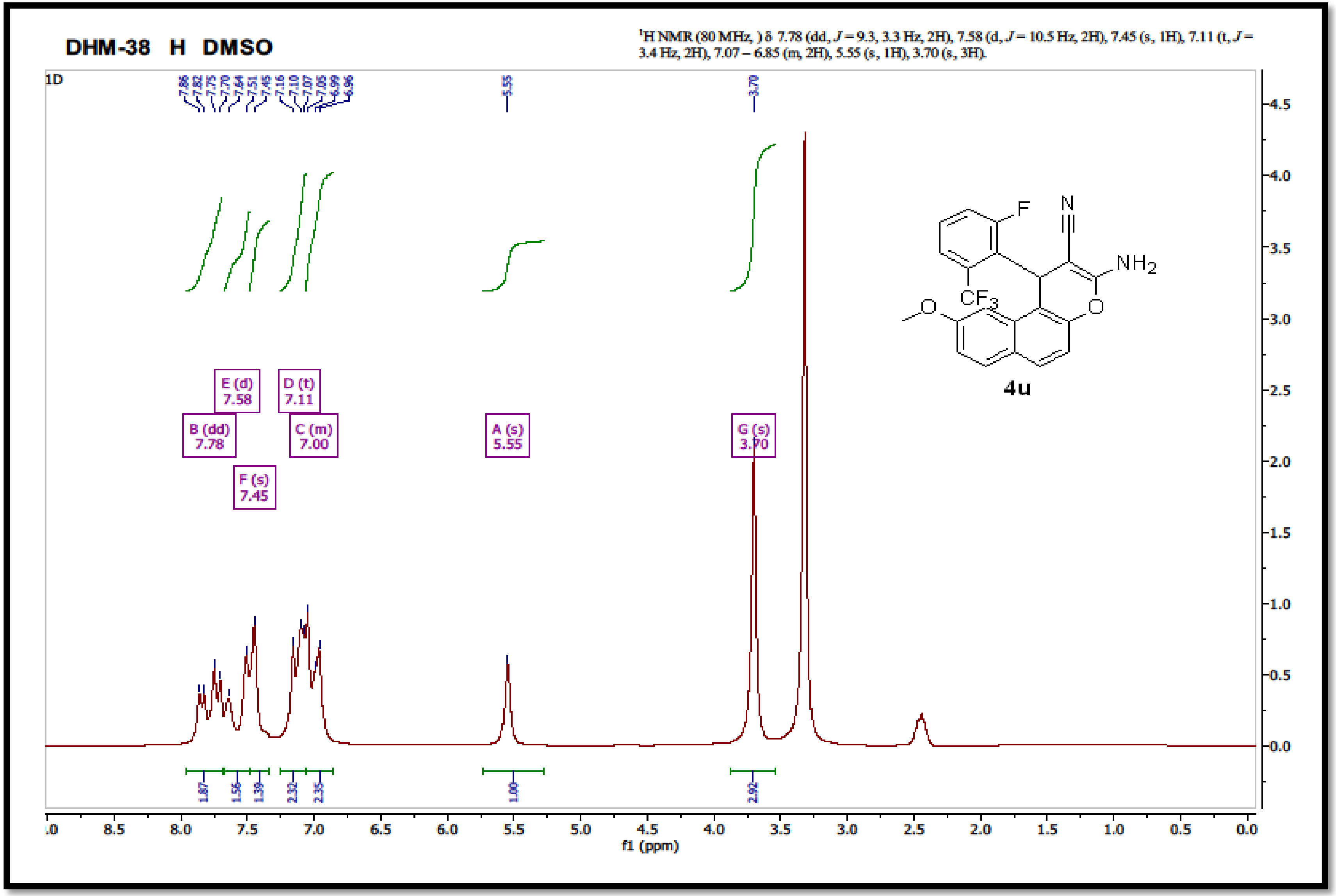

Figure S78: ^{1}H NMR spectrum (DMSO-d_6, 500 MHz) of compound **4u**.

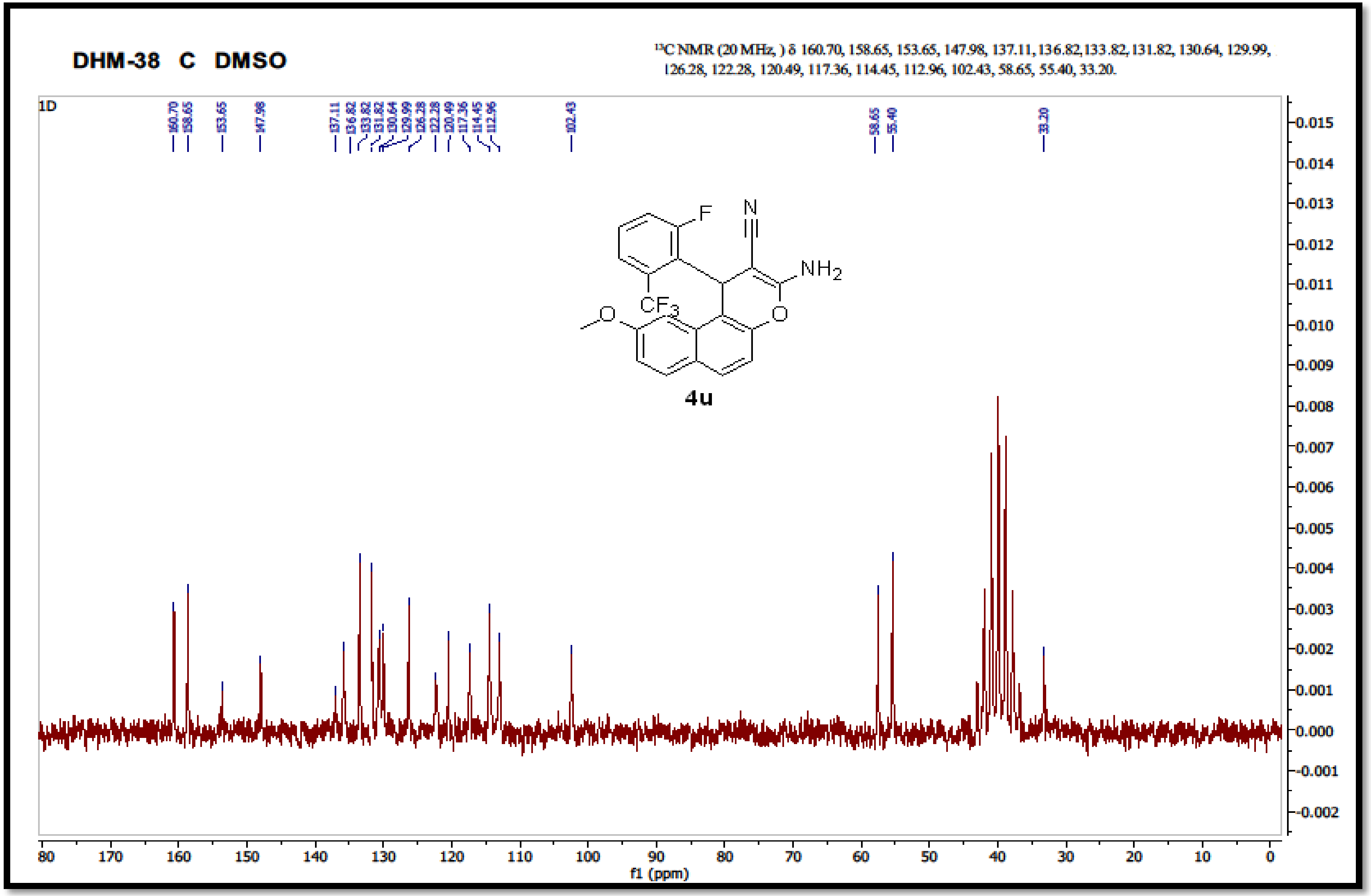

Figure S79: ^{13}C NMR spectrum (DMSO-d_6, 500 MHz) of compound **4u**.

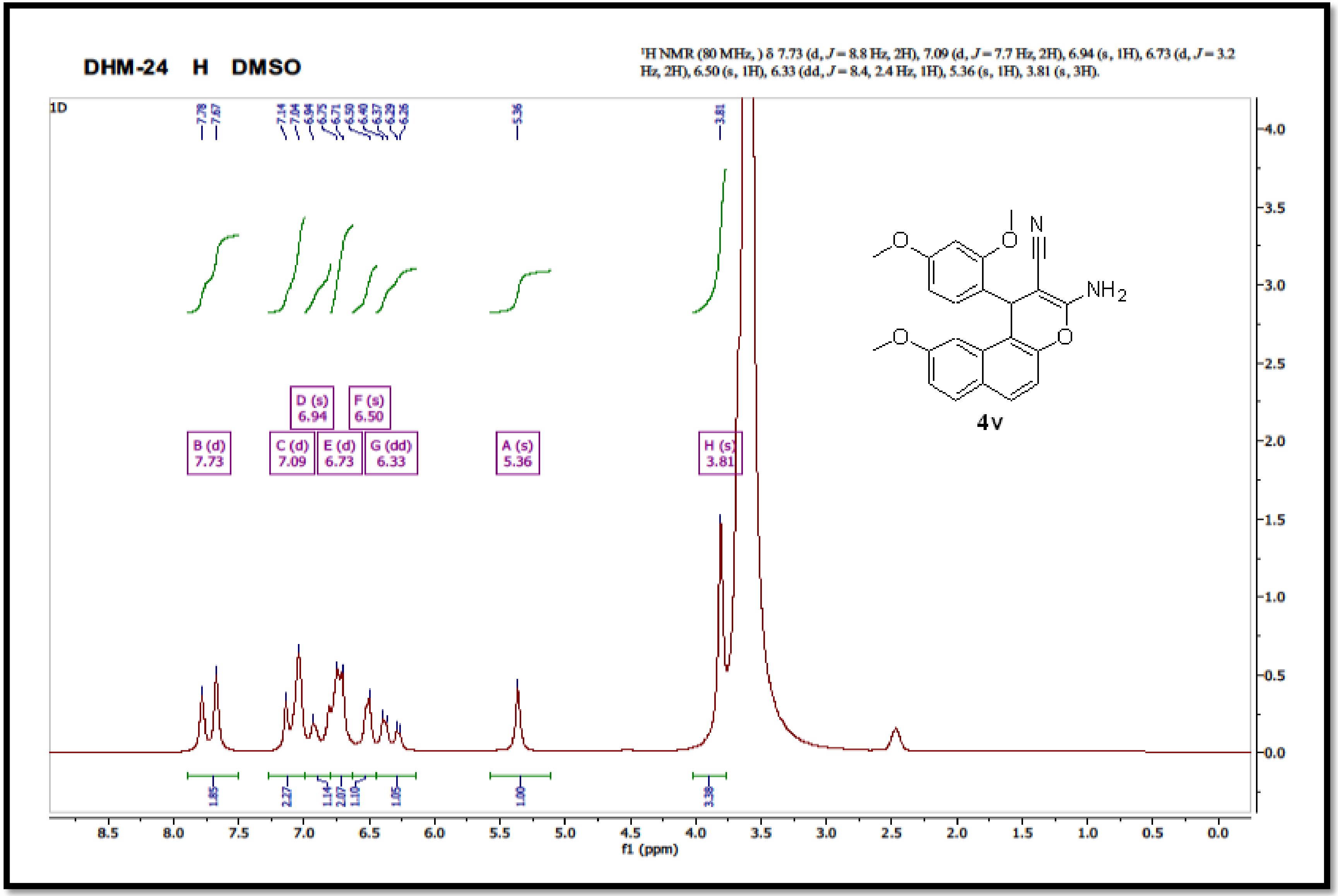

Figure S80: ^{1}H NMR spectrum (DMSO-d_6, 500 MHz) of compound **4v**.

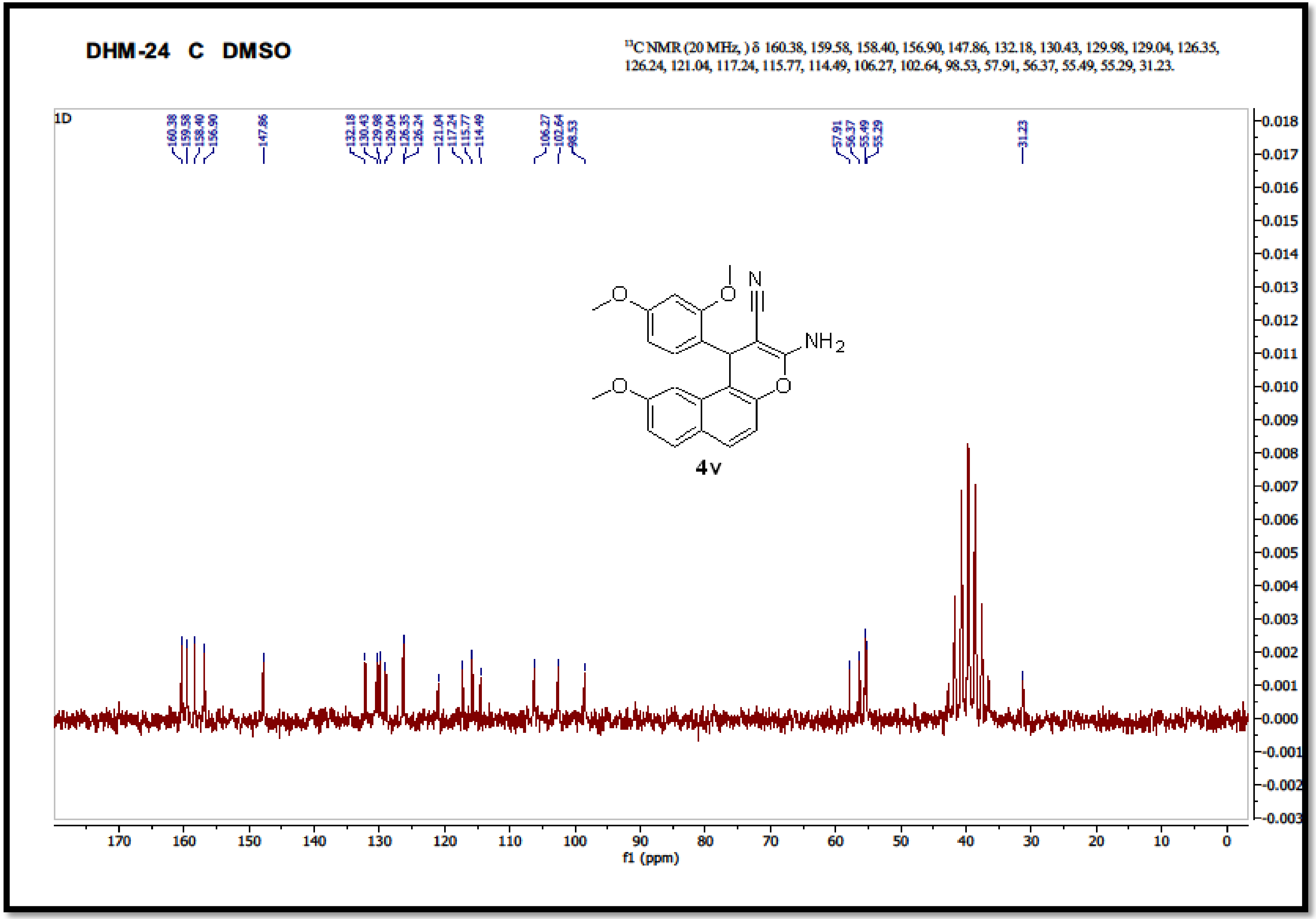

Figure S81: ^{13}C NMR spectrum (DMSO-d_6, 500 MHz) of compound **4v**.

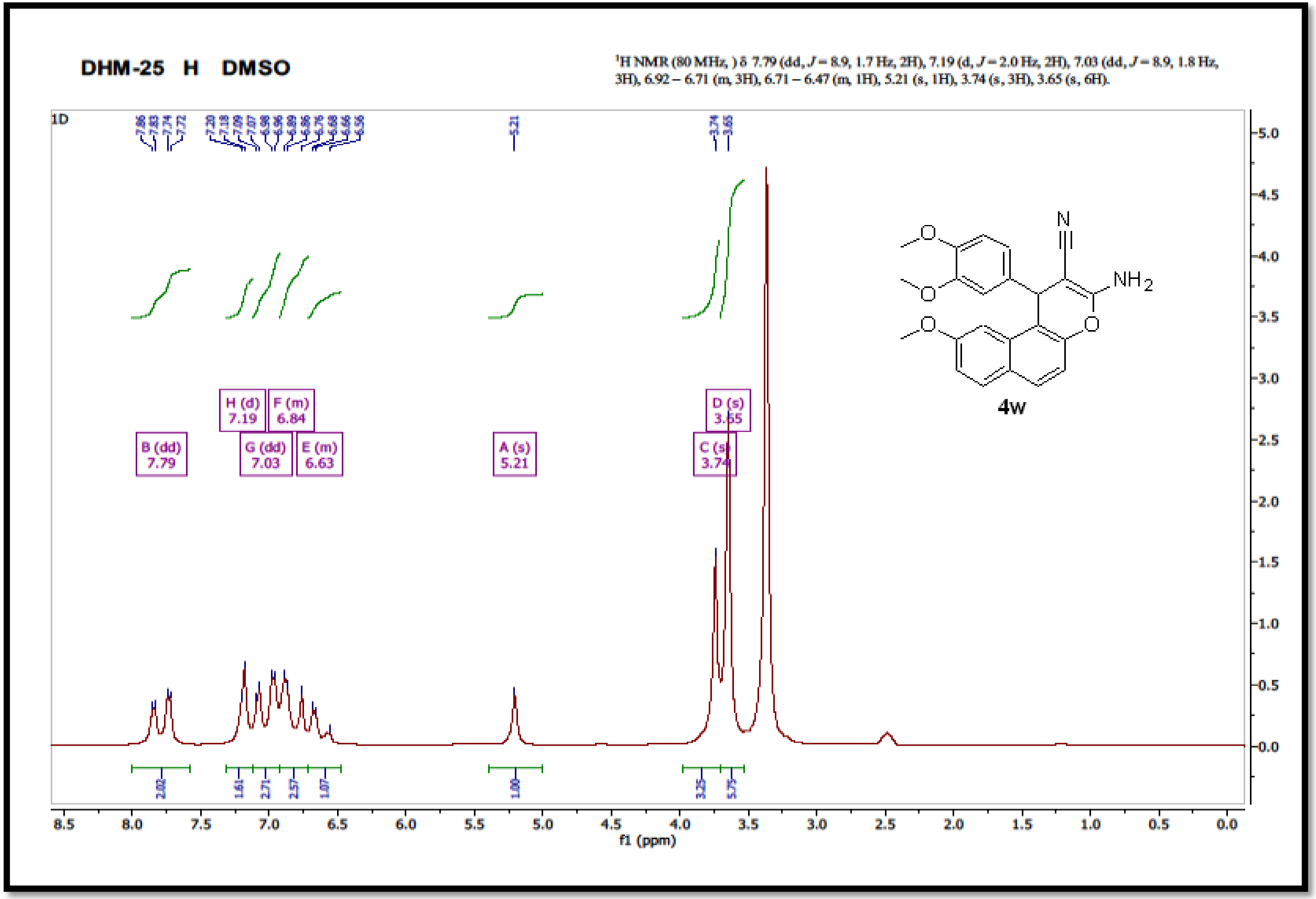

Figure S82: ^{1}H NMR spectrum (DMSO-d_6, 500 MHz) of compound **4w**.

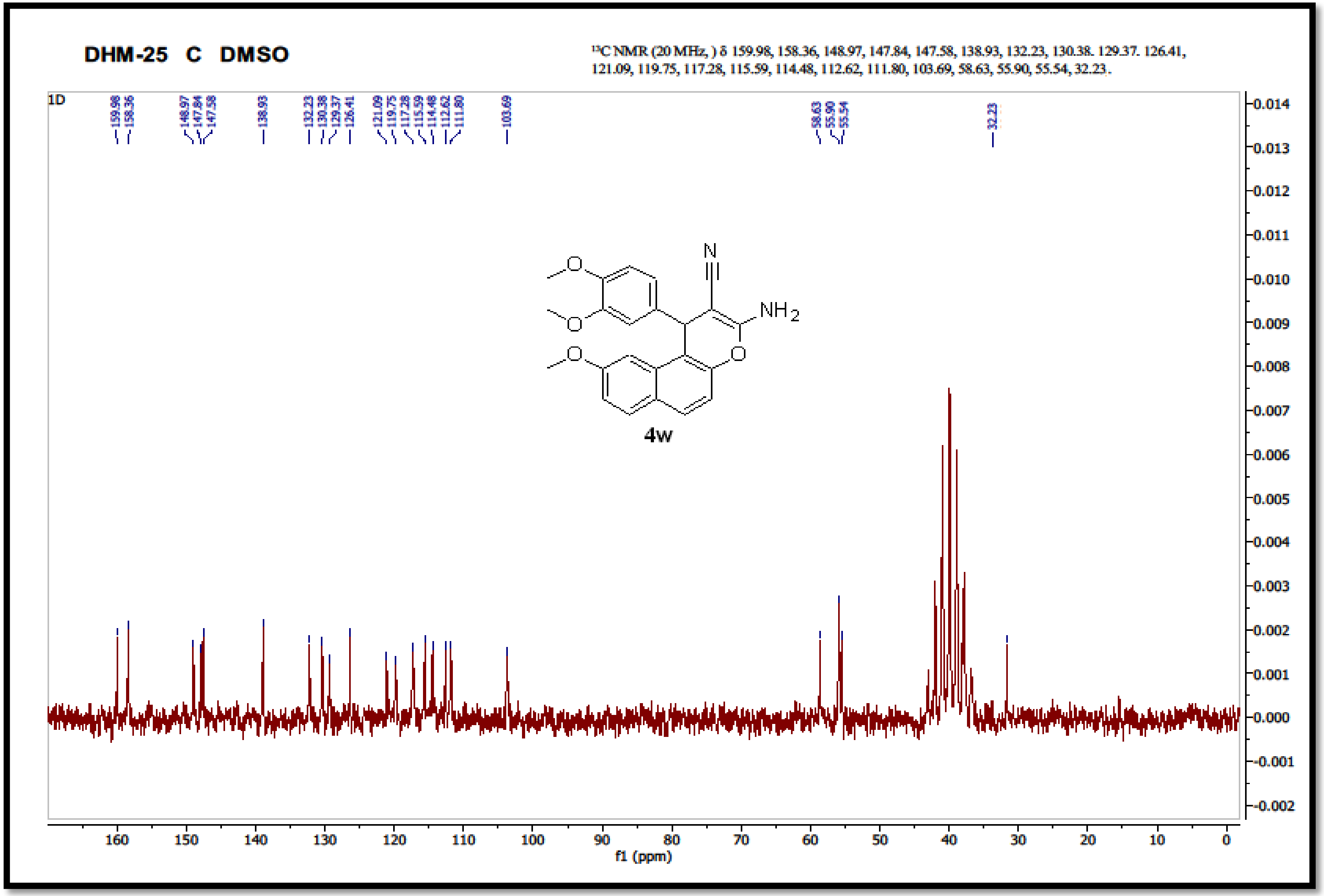

Figure S83: ^{13}C NMR spectrum (DMSO-d_6, 500 MHz) of compound **4w**.

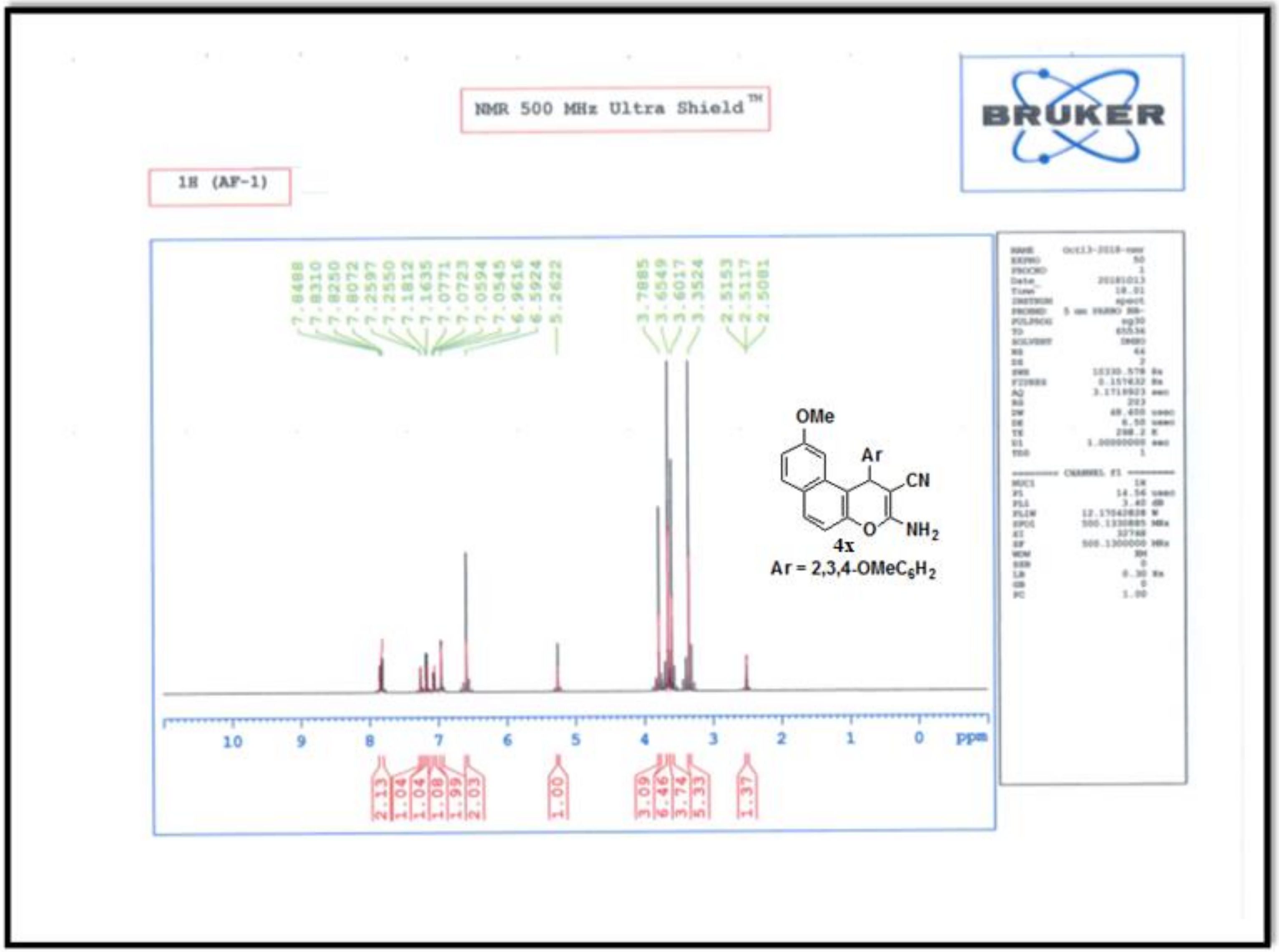

Figure S84: ^{1}H NMR spectrum (DMSO-d_6, 500 MHz) of compound **4x**.

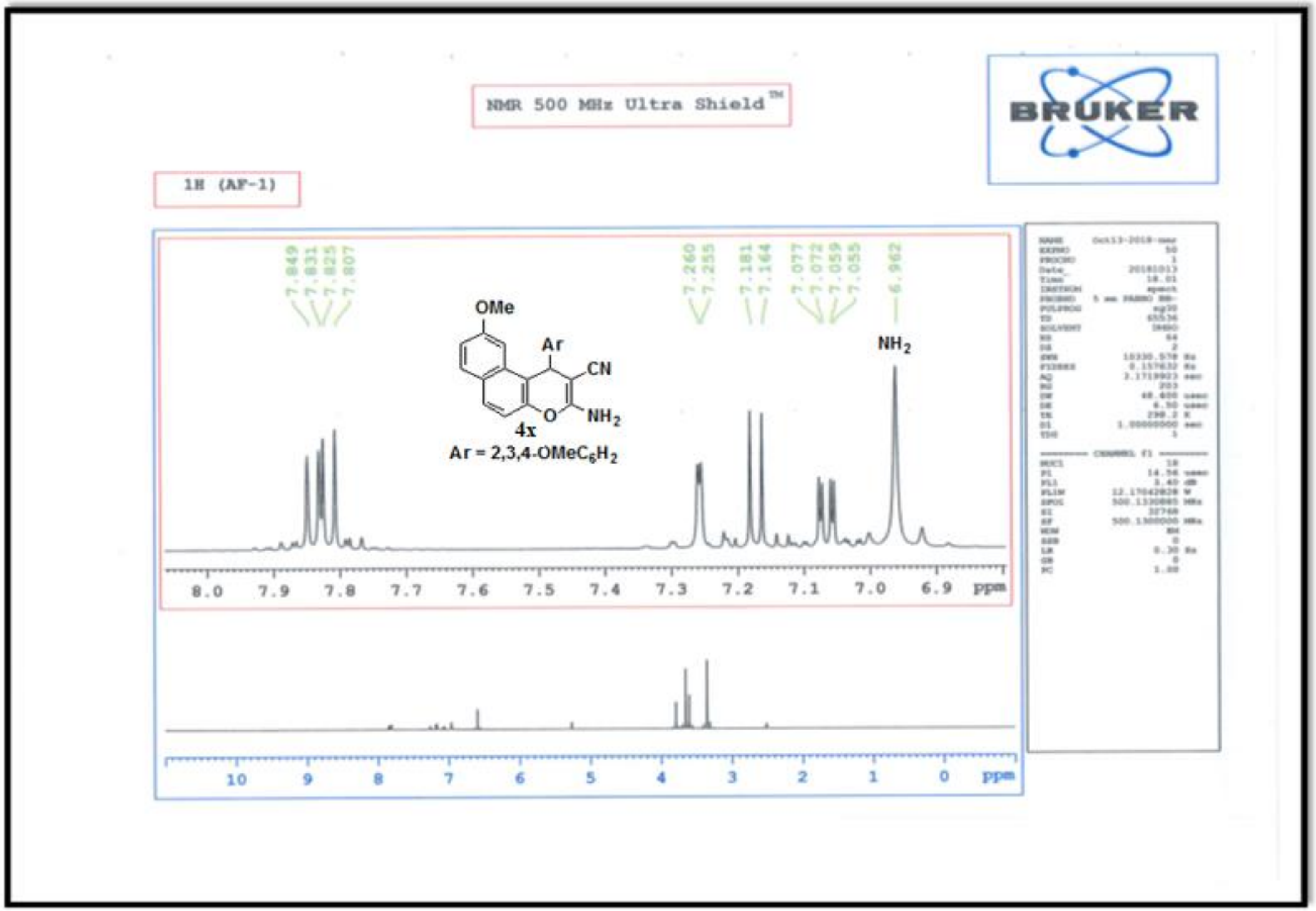

Figure S85: ^{1}H NMR spectrum (DMSO-d_6, 500 MHz) of compound **4x**.

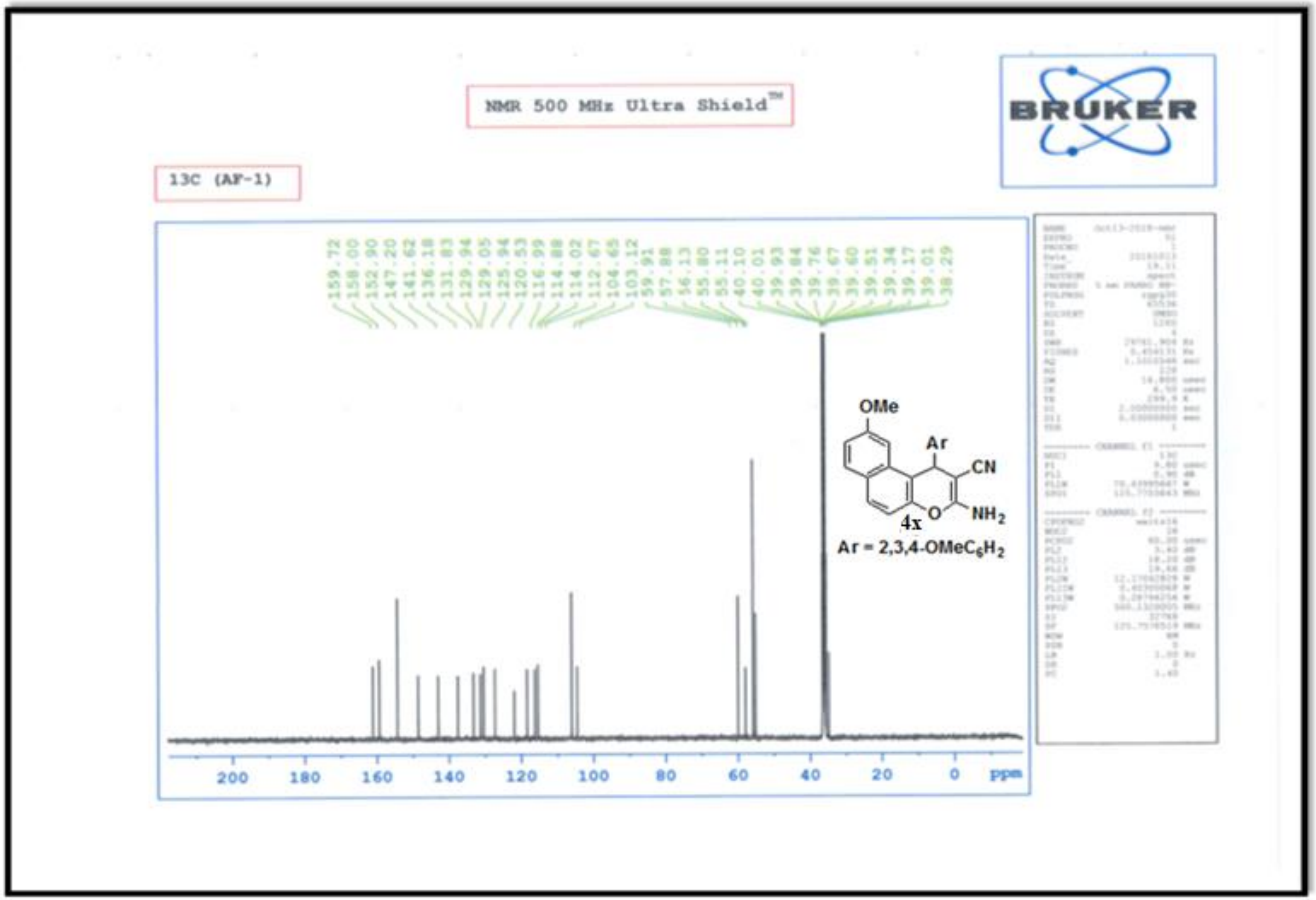

Figure S86: ^{13}C NMR spectrum (DMSO-d_6, 500 MHz) of compound **4x**.

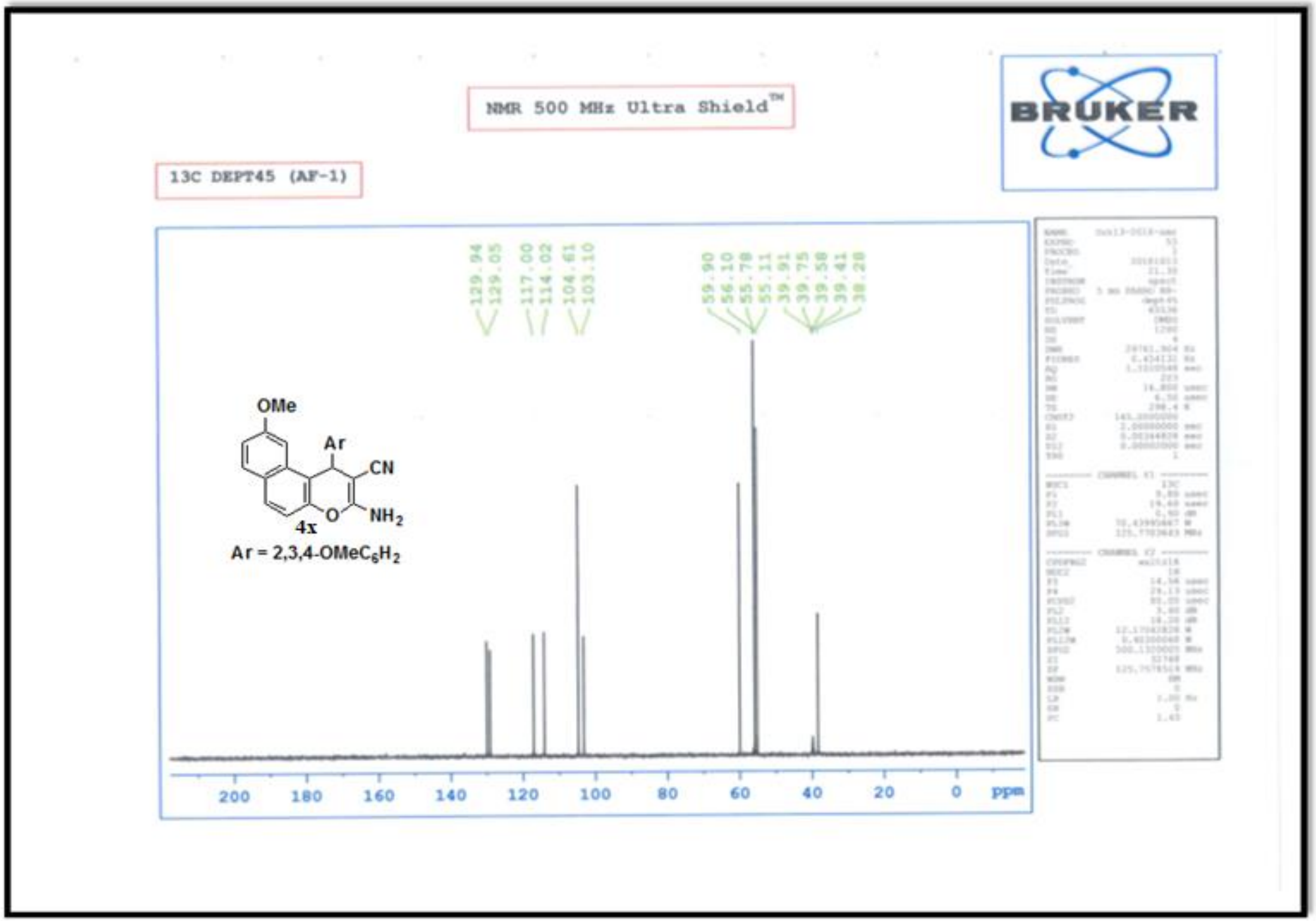

Figure S87: DEPT45 spectrum (DMSO-d_6, 125 MHz) of compound **4x.**

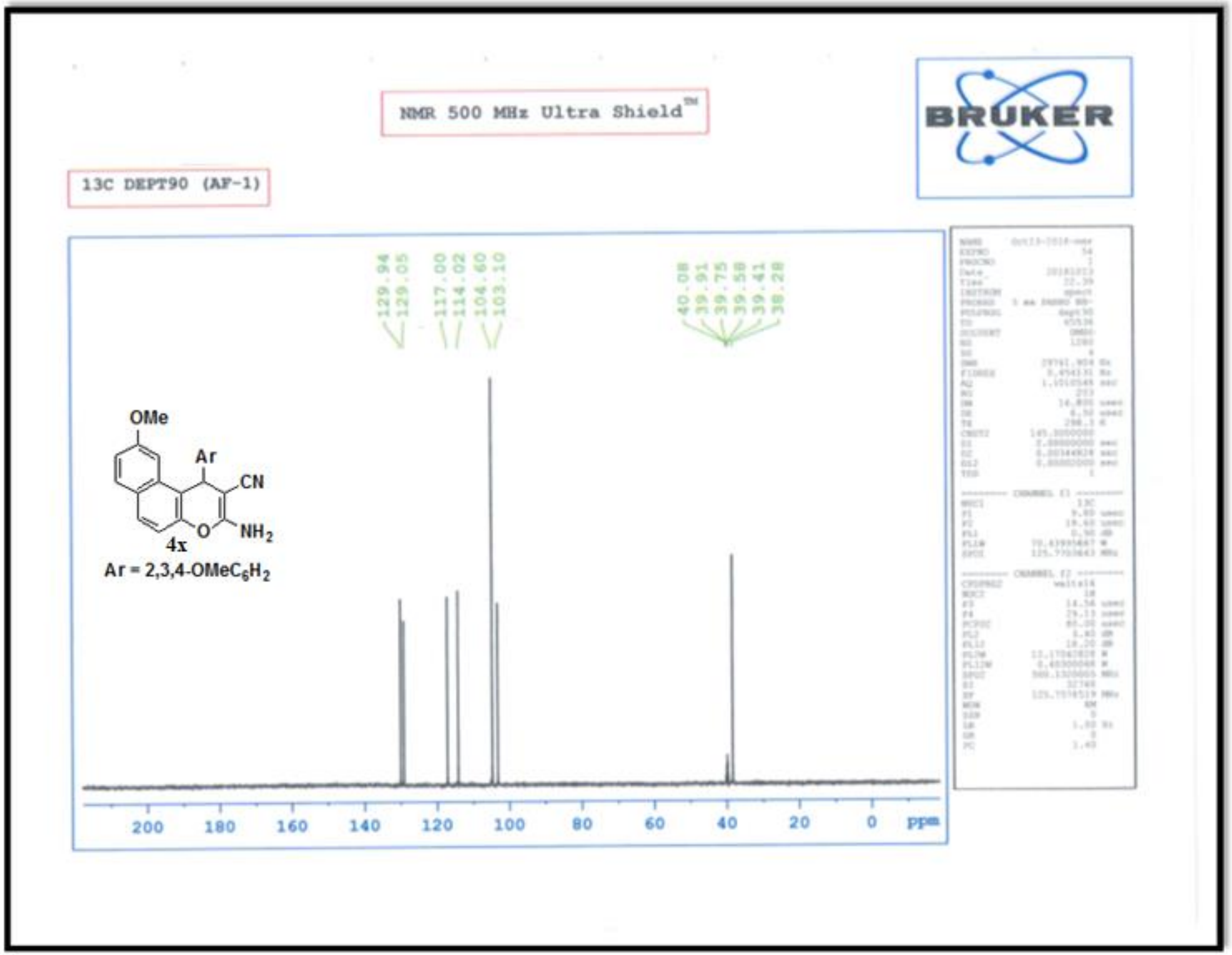

Figure S88: DEPT90 spectrum (DMSO-d_6, 125 MHz) of compound **4x.**

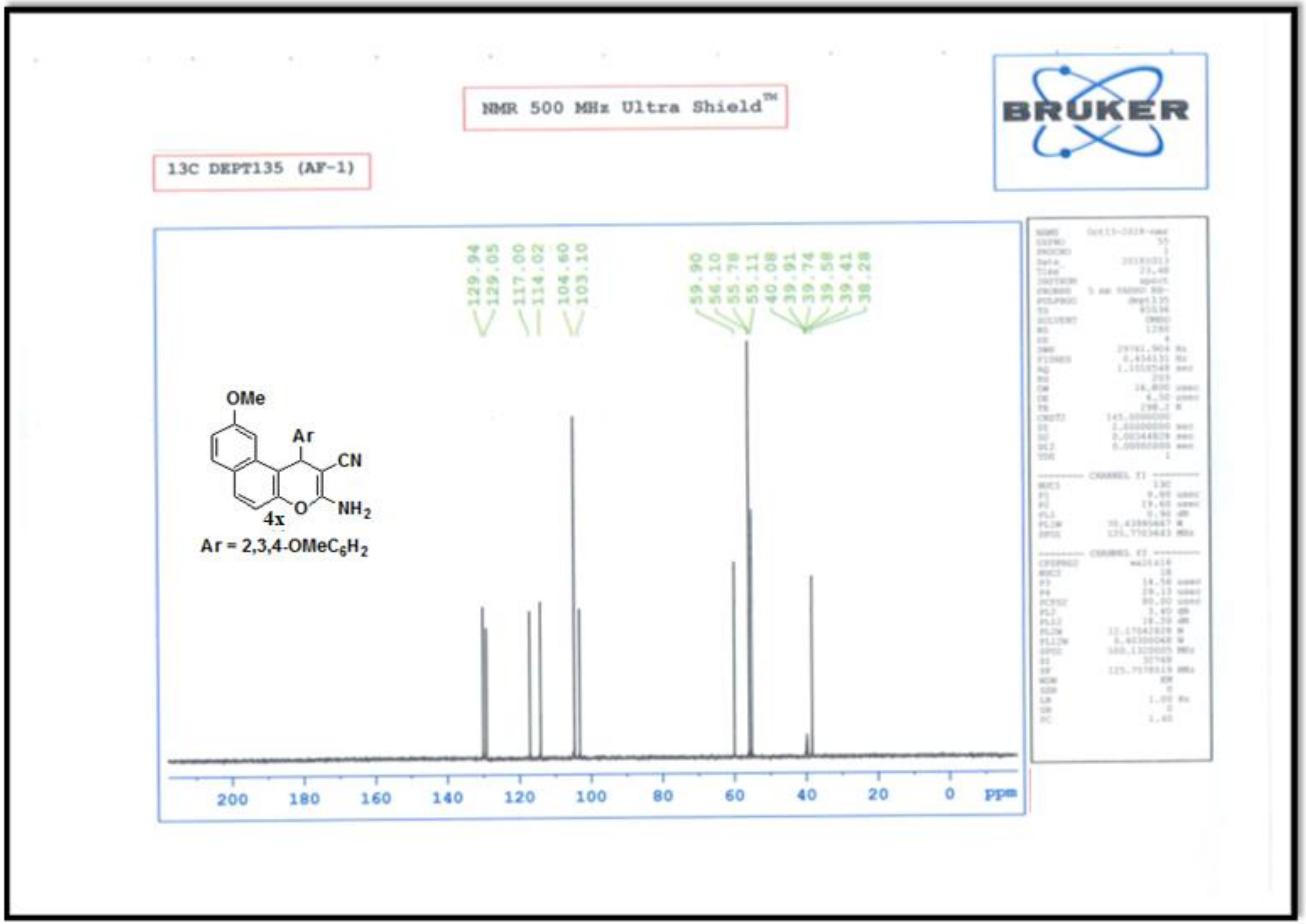

Figure S89: DEPT135 spectrum (DMSO-d_6, 125 MHz) of compound **4x.**

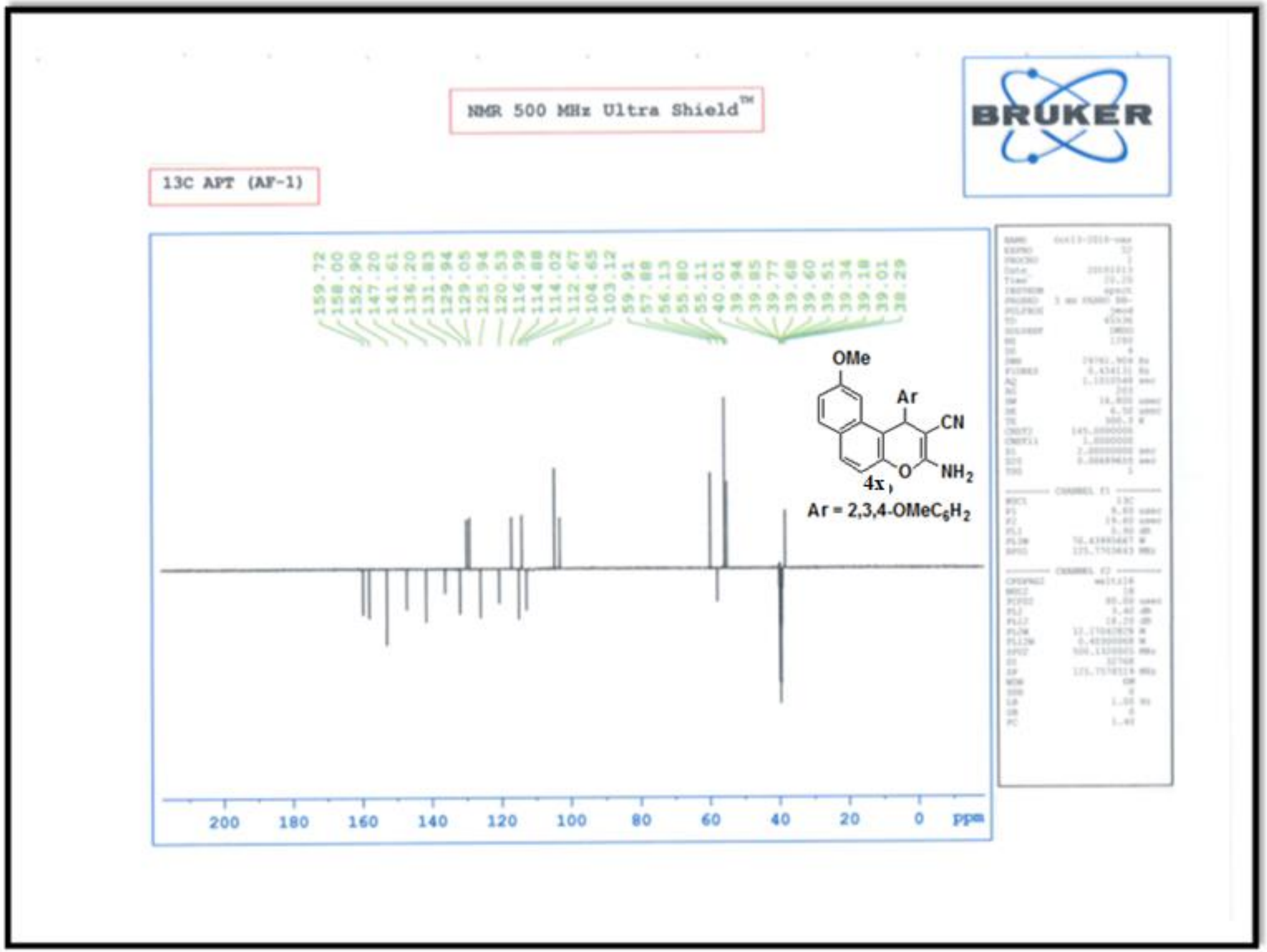

Figure S90: APT spectrum (DMSO-d_6, 125 MHz) of compound **4x**.

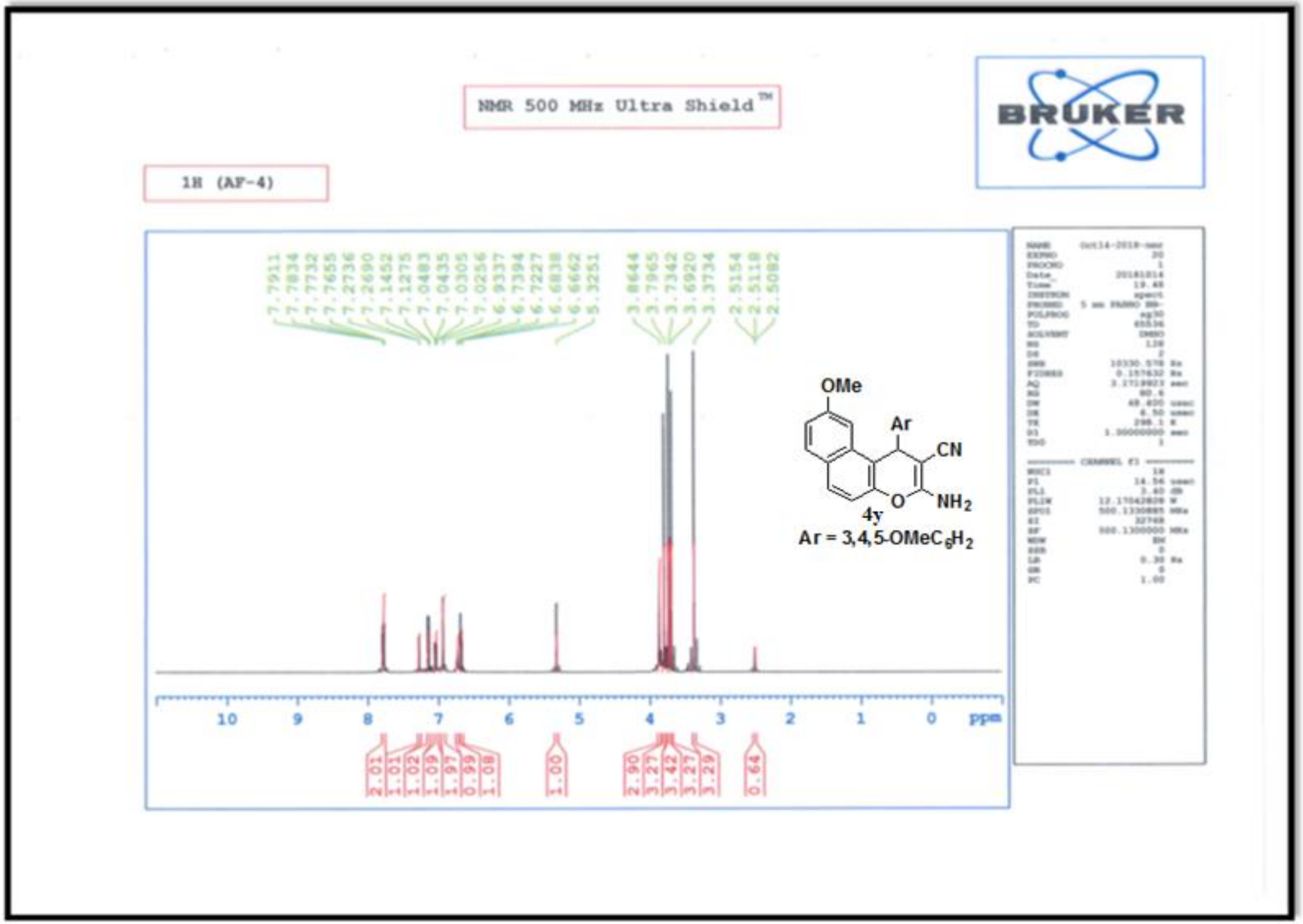

Figure S91: ^{1}H NMR spectrum (DMSO-d_6, 500 MHz) of compound **4y**.

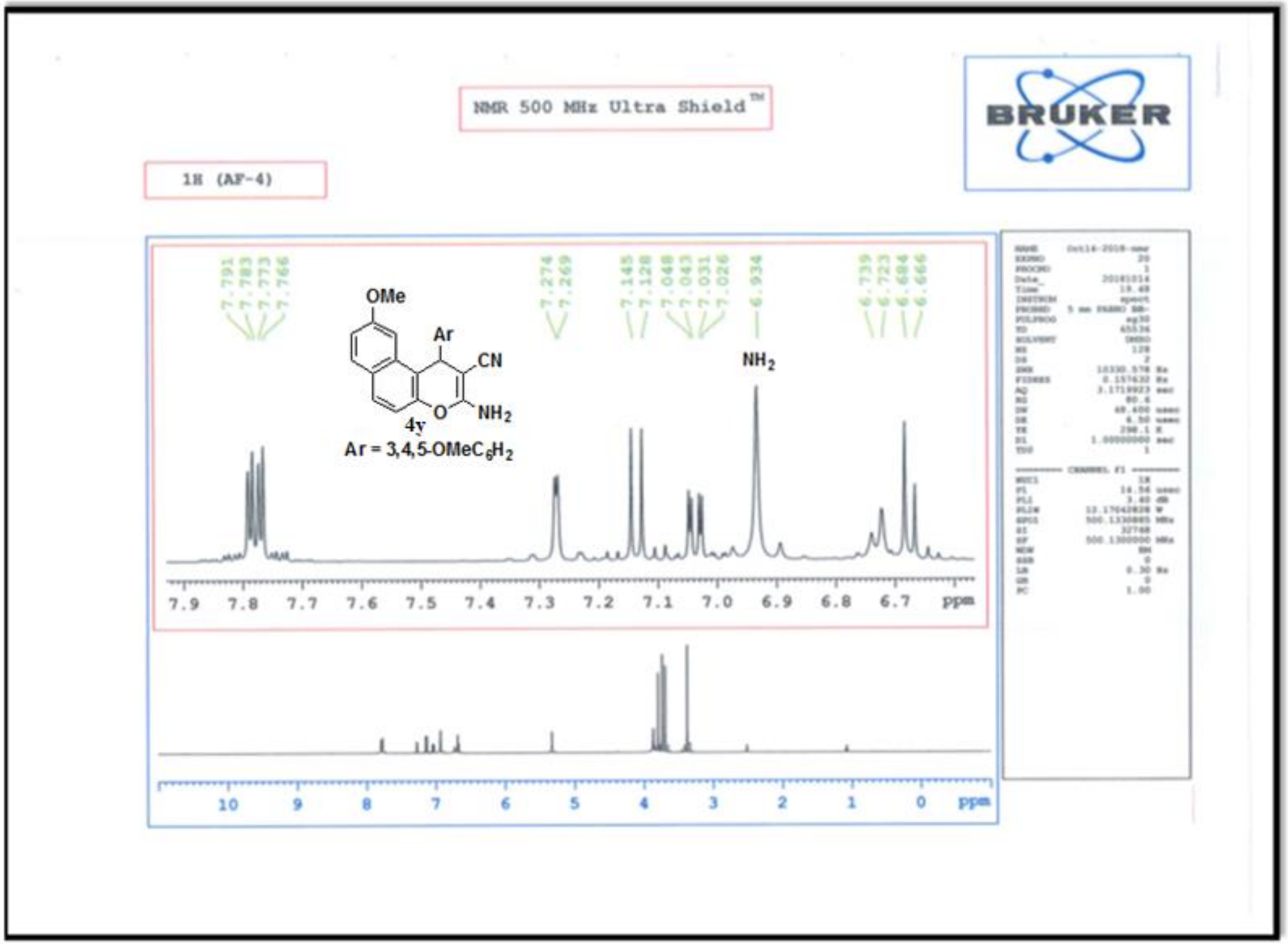

Figure S92: ^{1}H NMR spectrum (DMSO-d_6, 500 MHz) of compound **4y**.

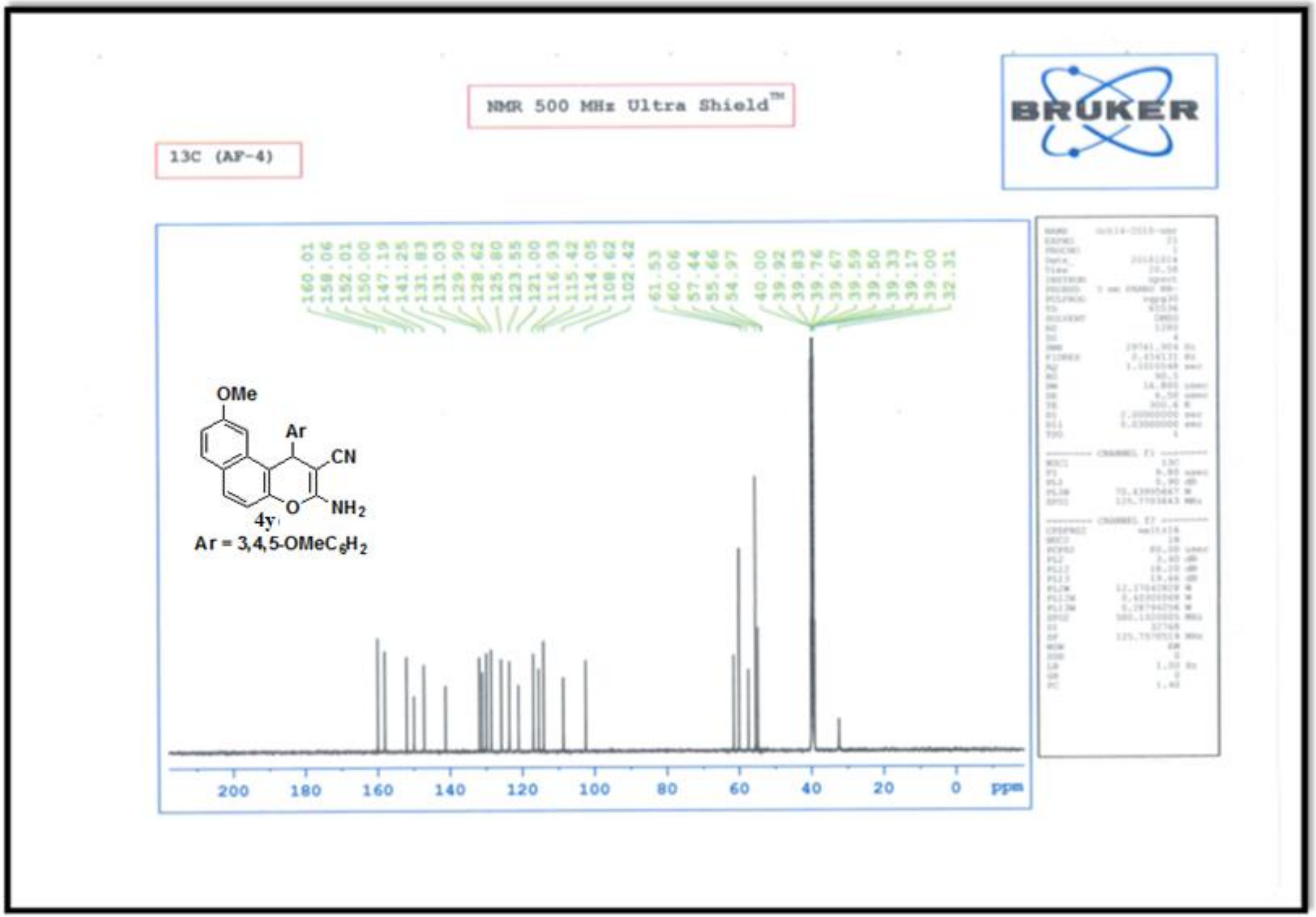

Figure S93: ^{13}C NMR spectrum (DMSO-d_6, 125 MHz) of compound **4y**.

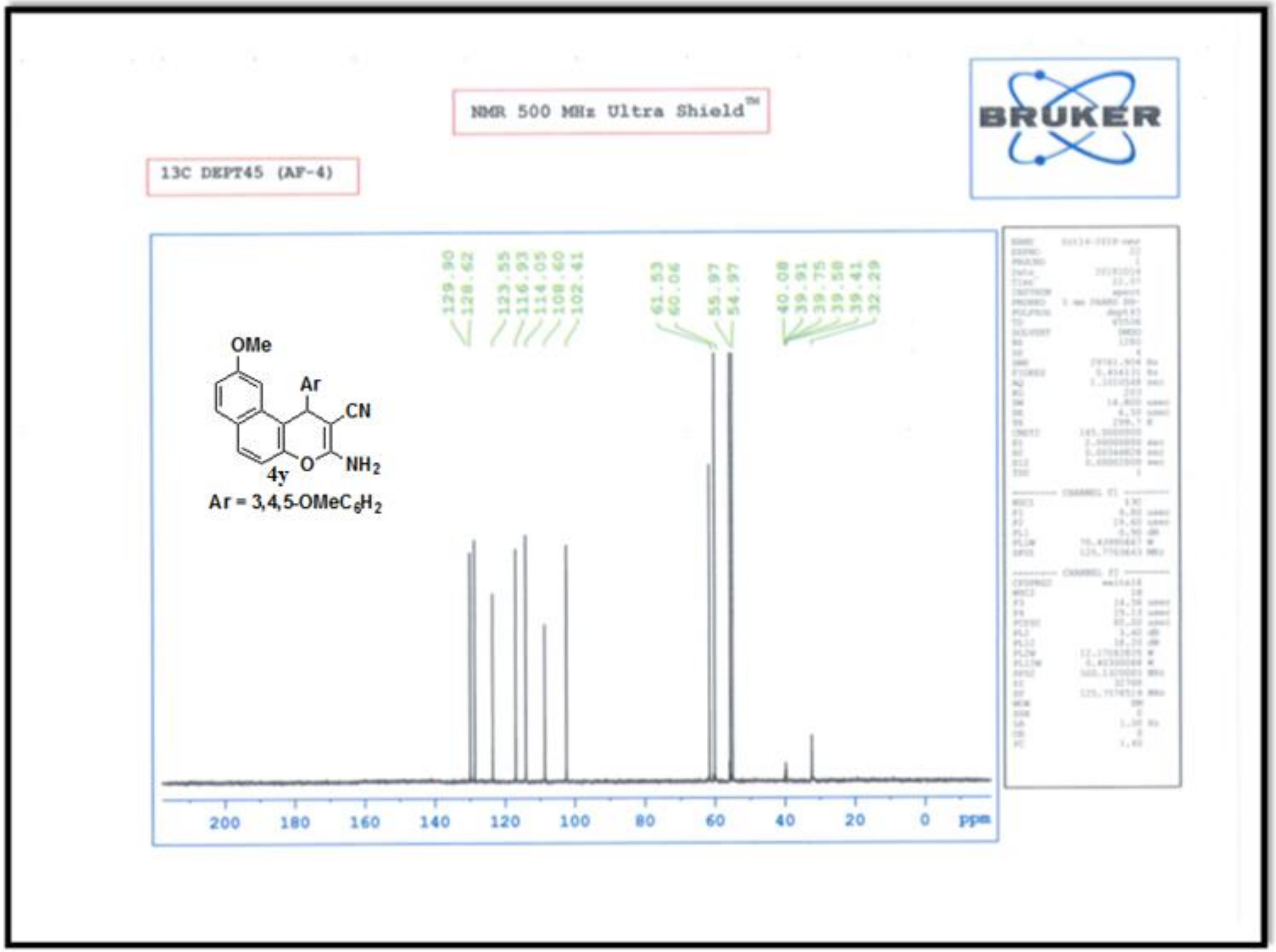

Figure S94: DEPT45 spectrum (DMSO-d_6, 125 MHz) of compound **4y.**

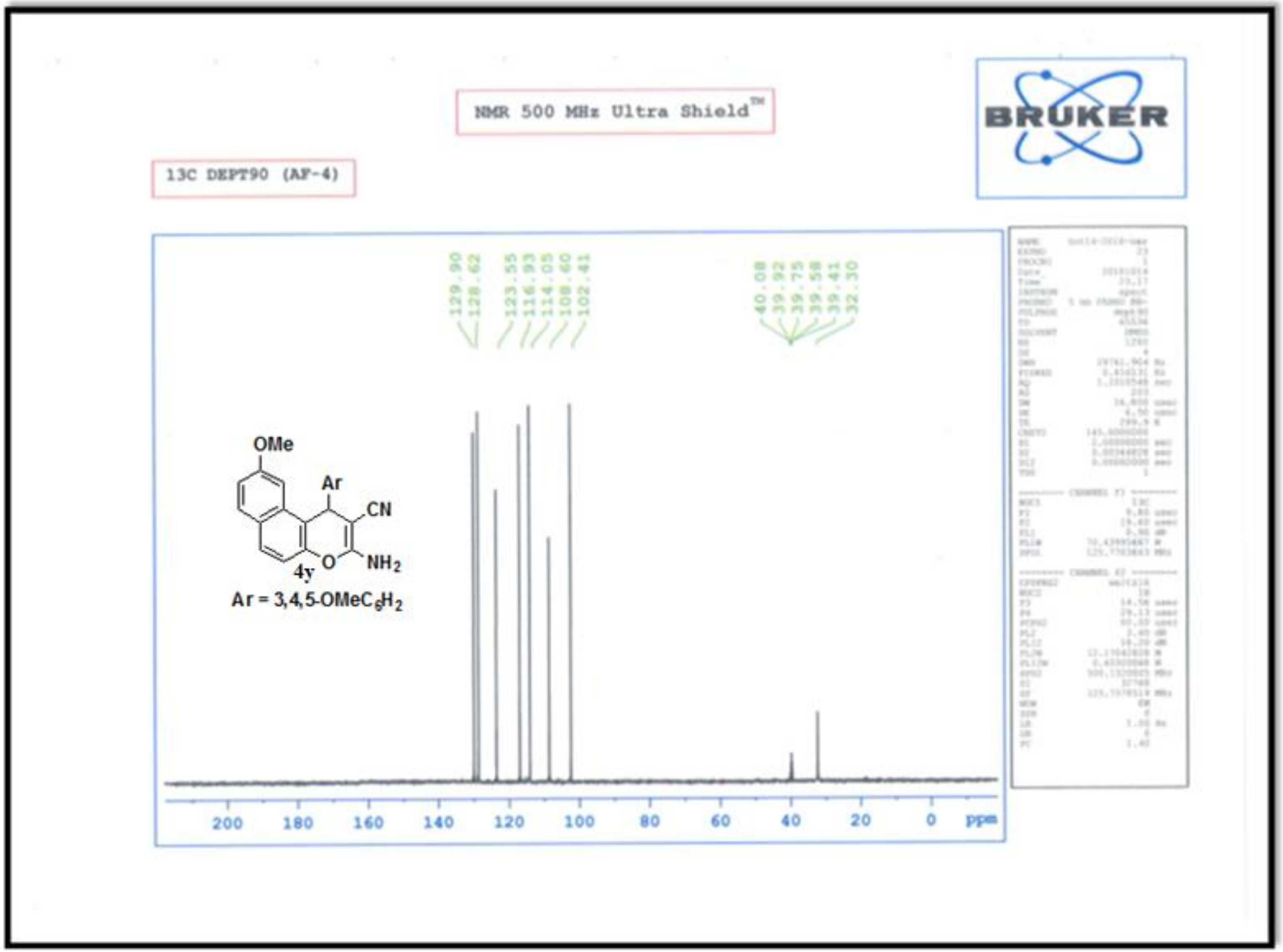

Figure S95: DEPT90 spectrum (DMSO-d_6, 125 MHz) of compound **4y.**

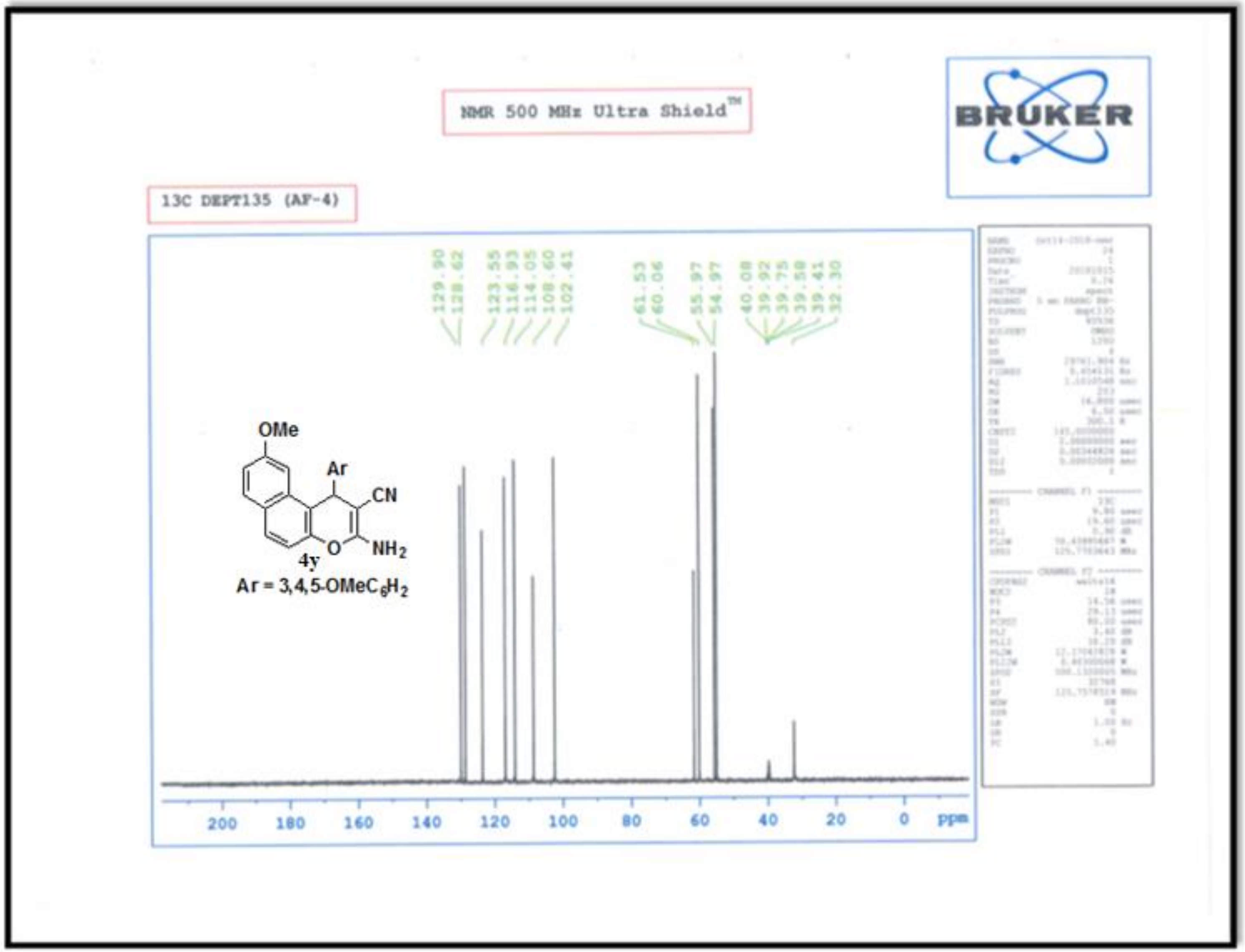

Figure S96: DEPT135 spectrum (DMSO-d_6, 125 MHz) of compound **4y**.

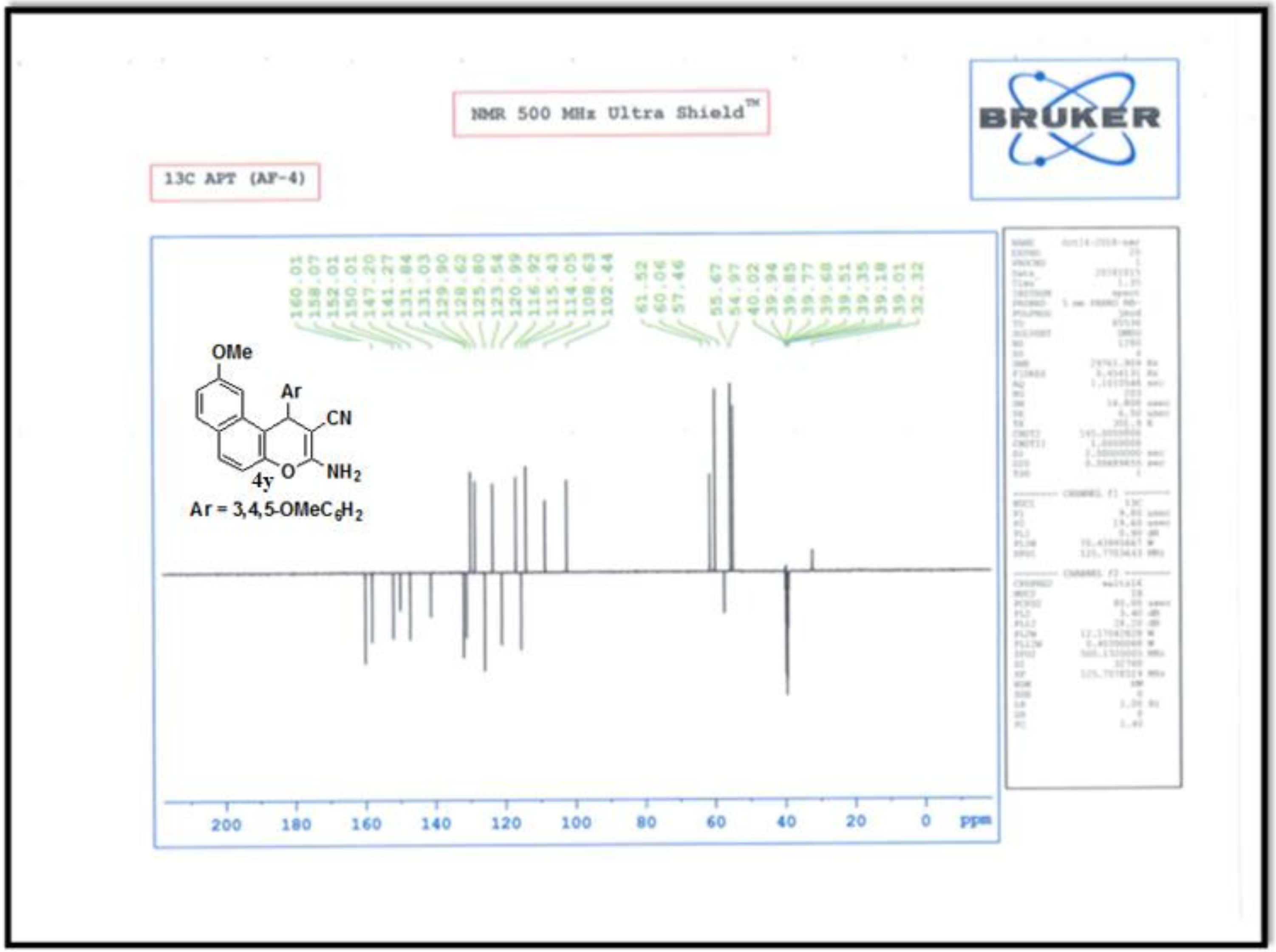

Figure S97: APT spectrum (DMSO-d_6, 125 MHz) of compound **4y**.

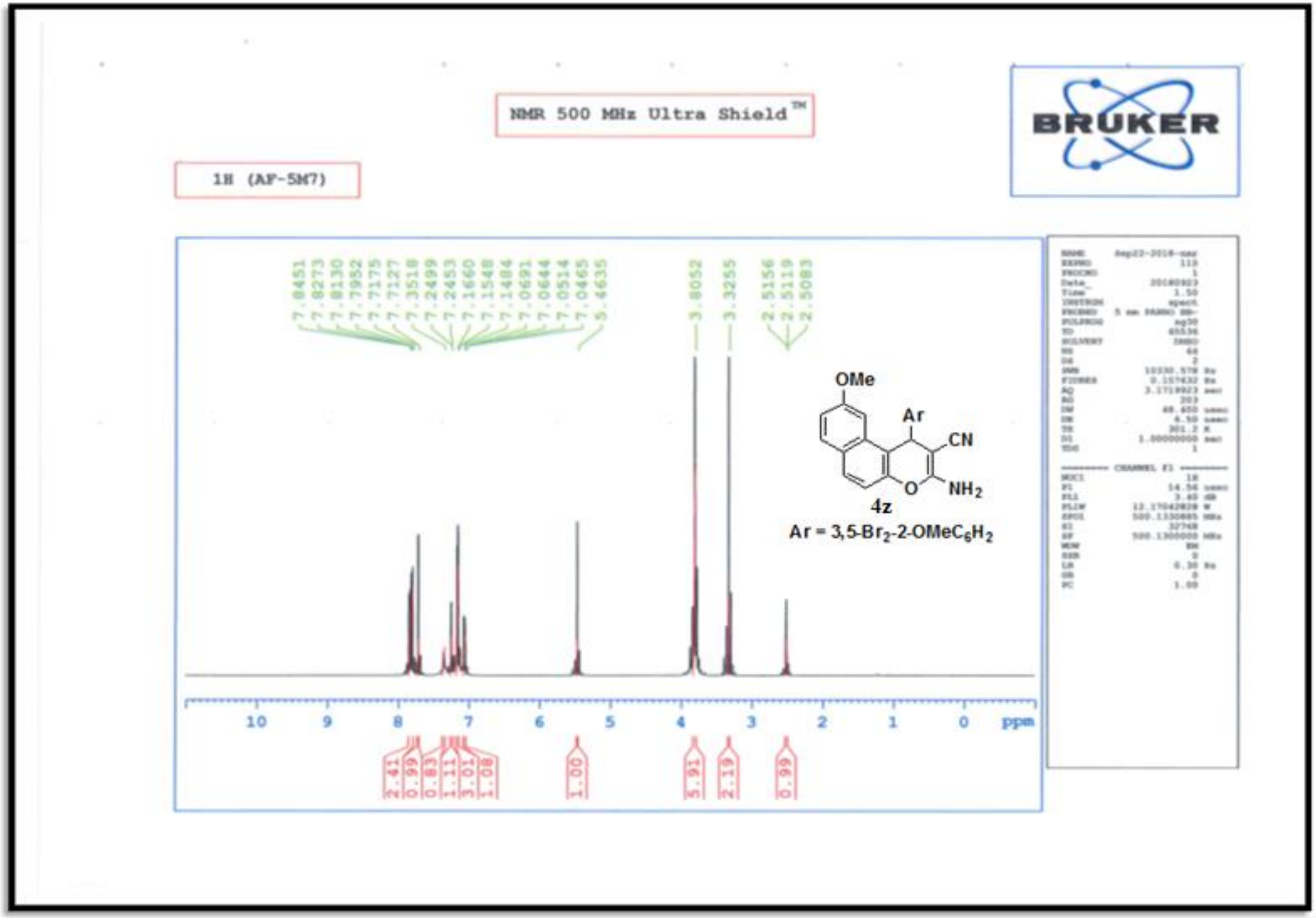

Figure S98: ^{1}H NMR spectrum (DMSO-d_6, 500 MHz) of compound **4z**.

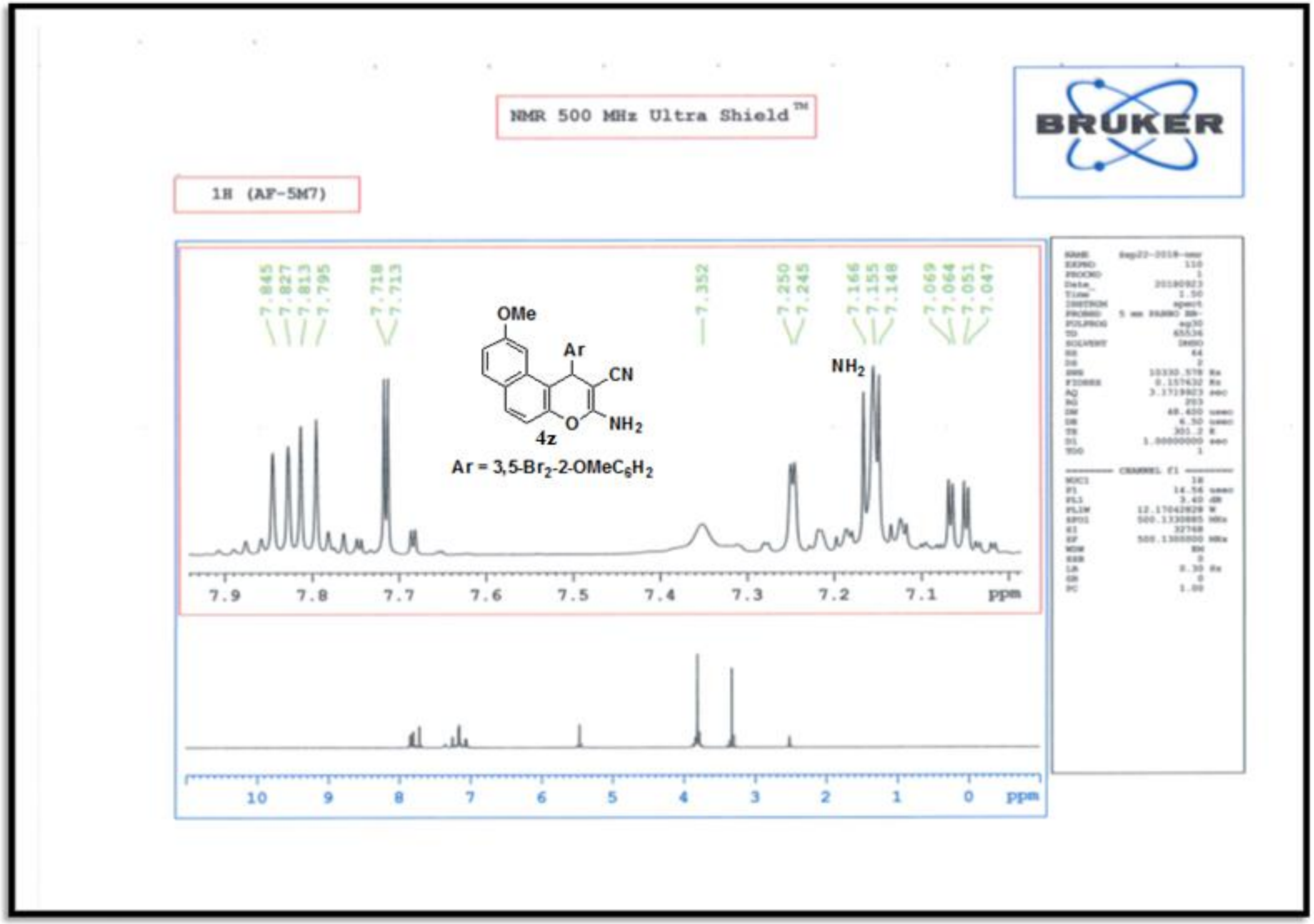

Figure S99: ^{1}H NMR spectrum (DMSO-d_6, 500 MHz) of compound **4z**.

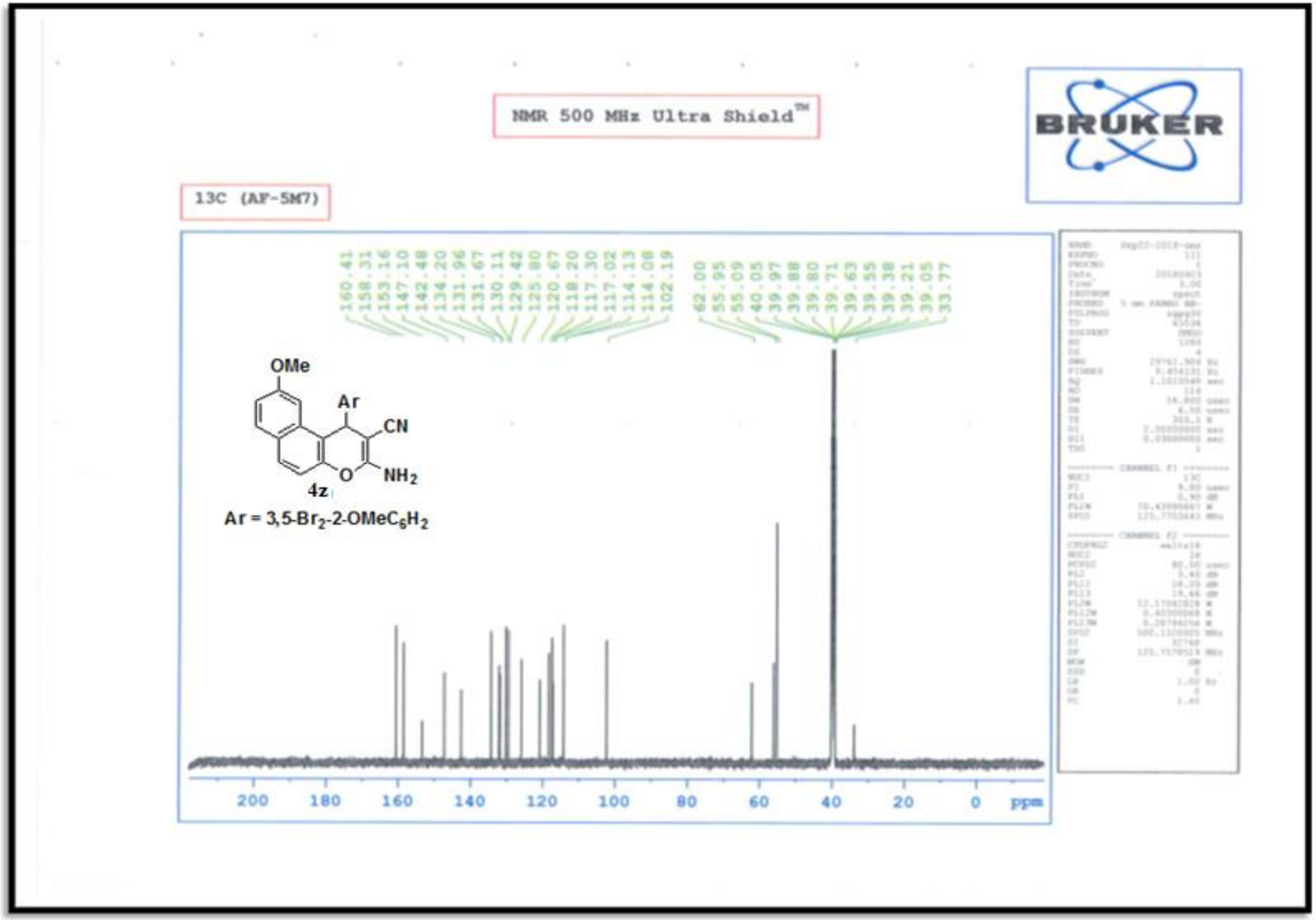

Figure S100: ^{13}C NMR spectrum (DMSO-d_6, 125 MHz) of compound **4z**.
